# Photocatalytic synthesis of homoallylic amines *via* nucleophilic addition of nickel allyl complexes to imines

**DOI:** 10.1039/d5sc06916e

**Published:** 2025-10-09

**Authors:** Christoph Nopper, Niclas Müller, Beloslava Goycheva, Felix Himmelsbach, Felix Bauer, Bernhard Breit

**Affiliations:** a Institut für Organische Chemie, Albert-Ludwigs-Universität Freiburg Albertstraße 21 79104 Freiburg im Breisgau Germany bernhard.breit@chemie.uni-freiburg.de

## Abstract

Homoallylic amines can be found in pharmaceutically interesting molecules and are versatile building blocks for total synthesis. Herein, we present a three-component coupling reaction of an aldehyde, an aniline and an allylic carbonate or allene to yield branched homoallylic amines in good yields and diastereoselectivity. Our straightforward protocol proceeds *via* the addition of an allyl nickel species to an *in situ* formed imine and represents the first photocatalytic realization of the classic approach of allyl metal addition to imines. Next to some follow-up transformations, a detailed reaction mechanism backed by experimental observations is presented.

## Abstract 2

We report the first addition of allyl nickel complexes to imines under photocatalytic conditions. This three-component reaction affords branched homoallylic amines in good yields and diastereoselectivity.

## Graphical abstract

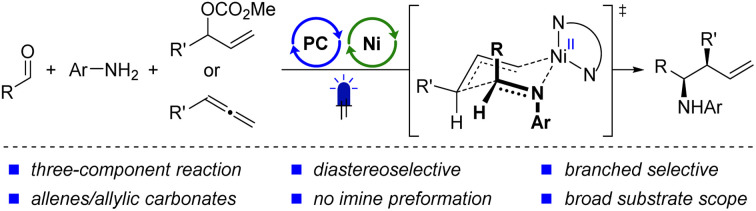

## Introduction

**Scheme 1:**
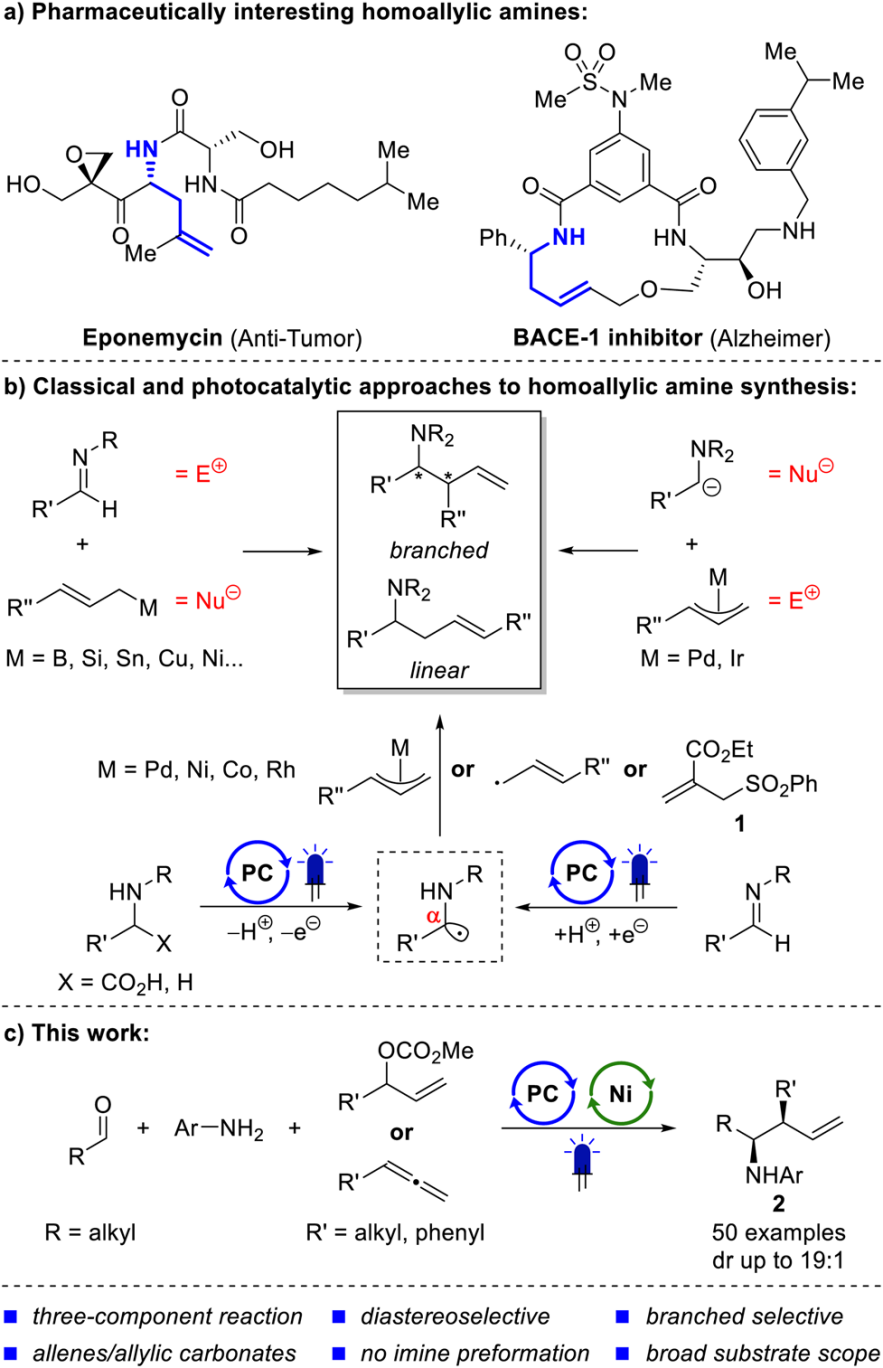
Interesting homoallylic amines (a), previous synthetic approaches (b) and this work (c).

Homoallylic amines are valuable building blocks for the synthesis of nitrogen-containing organic molecules.^1,2^ In addition to their occurrence in natural products^3^ and potential drug molecules^4^ (see Scheme 1a), they also serve as versatile intermediates in the synthesis of *N*-heterocycles and alkaloids.^2^ This highlights their significant role in organic chemistry and has prompted substantial research efforts to develop methodologies for homoallylic amine formation. The investigation of such methods involves challenging selectivity issues. Depending on the regioselectivity of the reaction, either the branched or the linear product can be formed (see Scheme 1b). In the case of the branched homoallylic amine, *syn*/*anti* selectivity must be controlled. Ideally, the products can also be synthesized enantioselectively.

The most common approach for the synthesis of homoallylic amines involves the addition of nucleophilic allyl metal species to imines (Scheme 1b, top left).^1,5,6^ Allylic nucleophiles based on B,^7^ Si,^8–10^ Sn,^11,12^ Cu,^13^ Ni,^14^ and others^1,5,15^ have been successfully added to imines. Reactions with complementary polarity, using α-nitrogen nucleophiles such as nitro-stabilized anions,^16,17^ α-amino acid-derived enolates^18,19^ and fluorenyl imine-derived anions,^20^ together with allylic electrophiles were also realized (Scheme 1b, top right). In addition to ionic pathways, photoredox catalysis has enabled the synthesis of homoallylic amines *via* radical mechanisms (Scheme 1b, bottom). Photocatalytically generated α-amino radicals from amines or α-amino acids can serve as formally “hard” nucleophiles in Pd-catalyzed Tsuji–Trost reactions or hydrofunctionalizations of allenes and 1,3-dienes.^21–26^ Similarly, Ni^27^ and Rh^28^ can catalyze such reactions using either allylic carbonates or allenes and alkynes, as demonstrated by our group. In another study, the Rovis group used 1,3-dienes as allyl precursors in a cobalt catalyzed allylation of α-amino radicals.^29^ α-Amino radicals can also be generated by photocatalytic reduction of imines. In 2016, Chen *et al.* presented a polarity-reversed allylation of imines, in which a Hantzsch ester-activated imine is reduced to an α-amino radical and subsequently undergoes a nucleophilic substitution with allyl phenyl sulfone 1 (Scheme 1b, bottom right).^30^ Imine-derived α-amino radicals were also cross-coupled with allyl radicals generated from butadiene and alkyl halides in a study by Shi *et al.*^4^ Similarly, Guan *et al.* demonstrated the coupling of allyl radicals from allyl bromide with α-amino radicals.^31^

Overall, numerous methods exist to access homoallylic amines, relying on a wide variety of chemical reactivity. However, many of these methods exhibit specific drawbacks. Classical nucleophilic addition reactions to imines typically generate stoichiometric amounts of metal salts.^5^ Previous photochemical methods developed by our group used *N*-aryl α-amino acids, which required preparation over two steps. The *N*-arylation *via* Ullmann chemistry typically suffered from low yields.^25–27^ Another protocol, using amines, is based on an expensive Rh catalyst and is limited in terms of substrate variability.^28^ All photocatalytic methods starting from imines reported so far have exclusively delivered the linear product.^4,30^

To the best of our knowledge, the addition of allyl metal compounds to imines has not yet been realized in a photocatalytic context. This is surprising, given that numerous protocols have already been published for the photochemical addition of allyl metal species to aldehydes. Metals successfully used in dual catalytic syntheses of homoallylic alcohols include Cr,^32–40^ Ni,^41–45^ Ti,^46,47^ Co^38–40,48,49^ and Bi.^50^ Based on this literature precedent and the detection of imines as intermediates in the decarboxylative photocatalytic allylation of α-amino acids,^27^ we began investigating the reductive allylation of imines *via* metallaphotocatalysis.

In this work, we present the first dual photoredox/nickel-catalyzed allylation of imines using allylic carbonates or allenes (Scheme 1c). The developed protocol delivers exclusively branched homoallylic amines (2) in good yields and diastereoselectivity. Aldehyde, aniline, and allylic carbonate or allene are combined in a three-component setup, without the need for imine preformation. Simple mechanistic experiments provide insights into photocatalyst interaction partners and nickel oxidation states. A few of the resulting homoallylic amines were also further functionalized to demonstrate their utility in a synthetic context.

## Results and discussion

We began our optimization of this reaction using 3-phenylpropanal (3), *p*-anisidine (4) and allyl methyl carbonate 5. Stirring NiCl_2_ (10 mol%) and 2,2′-bipyridine (12 mol%) for 30 min at room temperature in THF generated the active nickel catalyst. Subsequently, [Ir(ppy)_2_dtbbpy]PF_6_ (9, 1.0 mol%), *p*-anisidine (4, 1.0 equiv.), Hantzsch ester (HEH, 1.5 equiv.), 3-phenylpropanal (3, 1.5 equiv.), i-Pr_2_NEt (2.0 equiv.) and allylic carbonate (5, 1.2 equiv.) were added, and the mixture was stirred under blue LED irradiation (*λ* = 452 nm) overnight. The branched homoallylic amine 7 was obtained in 93% NMR yield (80% isolated), with a 4 : 1 diastereomeric ratio (entry 1, Table 1). Notably, the linear homoallylic amine was not observed. Using 1,4-dioxane gave a similar yield of 91% (entry 2). However, deviations from these conditions generally led to lower yields (see Table 1). A few experiments using chromium or cobalt as transition metal catalyst were also performed. In these cases, no product was obtained (see SI for details).

**Table 1:** Optimization of the photocatalytic homoallylic amine synthesis^a^

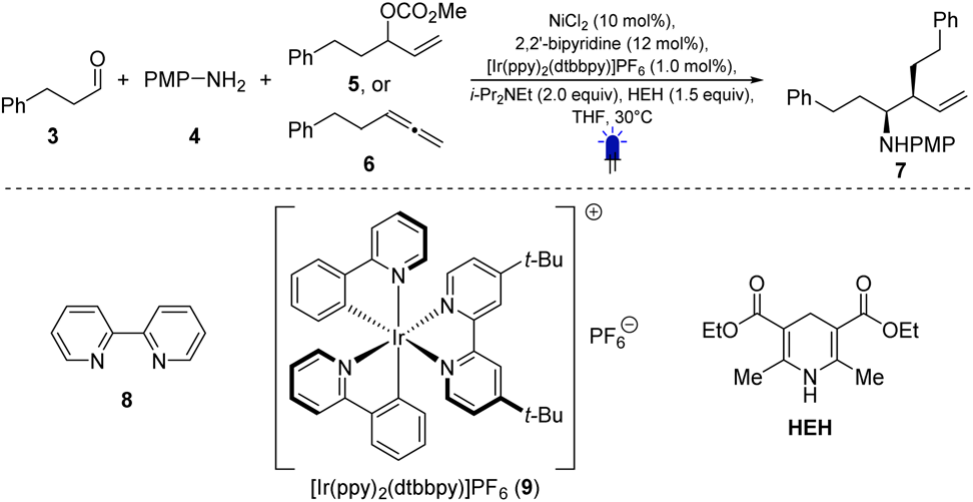			
Entry	Deviations from standard conditions	Yield [%]	dr
**1**	**None**	**93(80)**	**4 : 1**
2	1,4-Dioxane instead of THF (0.1 M)	91	4 : 1
3	Toluene instead of THF (0.1 M)	63	4 : 1
4	Ni(COD)_2_ instead of NiCl_2_ (0.1 M)	79	4 : 1
5	NiCl_2_(glyme) instead of NiCl_2_ (0.1 M)	46	4 : 1
6	4,4′-(MeO)_2_-2,2′-bipyridine as ligand (0.1 M)	25	4 : 1
7	4,4′-(CF_3_)_2_-2,2′-bipyridine as ligand (0.1 M)	89	4 : 1
8	Allene 6 (1.2 equiv., 0.1 M)	75	4 : 1
9	**Allene 6 (1.4 equiv., 0.1 M)**	**76(68)**	**4:1**
10	No NiCl_2_	0	—
11	No [Ir(ppy)_2_(dtbbpy)]PF_6_	0	—
12	No i-Pr_2_NEt	0	—
13	No HEH	0	—
14	No light	0	—

a Standard conditions: aldehyde (3, 1.5 equiv.), *p*-anisidine (4, 200 μmol, 1.0 equiv.), allylic carbonate (5, 1.2 equiv.) or allene (6, 1.4 equiv.), NiCl_2_ (10 mol%), 2,2′-bipyridine (12 mol%), [Ir(ppy)_2_(dtbbpy)]PF_6_ (1.0 mol%), i-Pr_2_NEt (2.0 equiv.), Hantzsch ester (HEH, 1.5 equiv.), THF [0.2 M (allyl carbonate) or 0.1 M (allene)], 30 °C, blue LEDs, 18–24 h. Yields and diastereoselectivities were calculated from the ^1^H-NMR spectrum of the crude product. 1,3,5-Trimethoxybenzene was used as an internal standard. Isolated yields in brackets.

We found that allylic carbonates could be replaced with allenes, resulting in higher atom economy for the transformation. Nevertheless, allene hydrofunctionalizations typically gave lower yields (entries 8 and 9). Interestingly, the diastereomeric ratio (dr) remained largely unaffected by changes in reaction conditions. A more detailed description of the optimization process is provided in the SI.

**Scheme 2:**
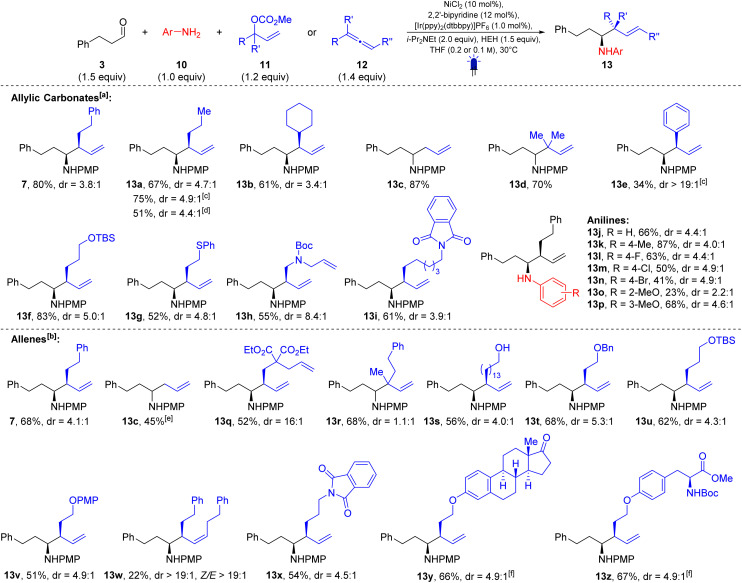
Scope I – allylic carbonates, allenes and anilines. ^[a]^*c* = 0.2 M; ^[b]^*c* = 0.1 M; ^[c]^ linear allylic carbonate was used; ^[d]^ branched allylic acetate was used; ^[e]^ 2.0 equiv. propa-1,2-diene were used; ^[f]^ dr referring to the newly formed stereocenters.

**Scheme 3:**
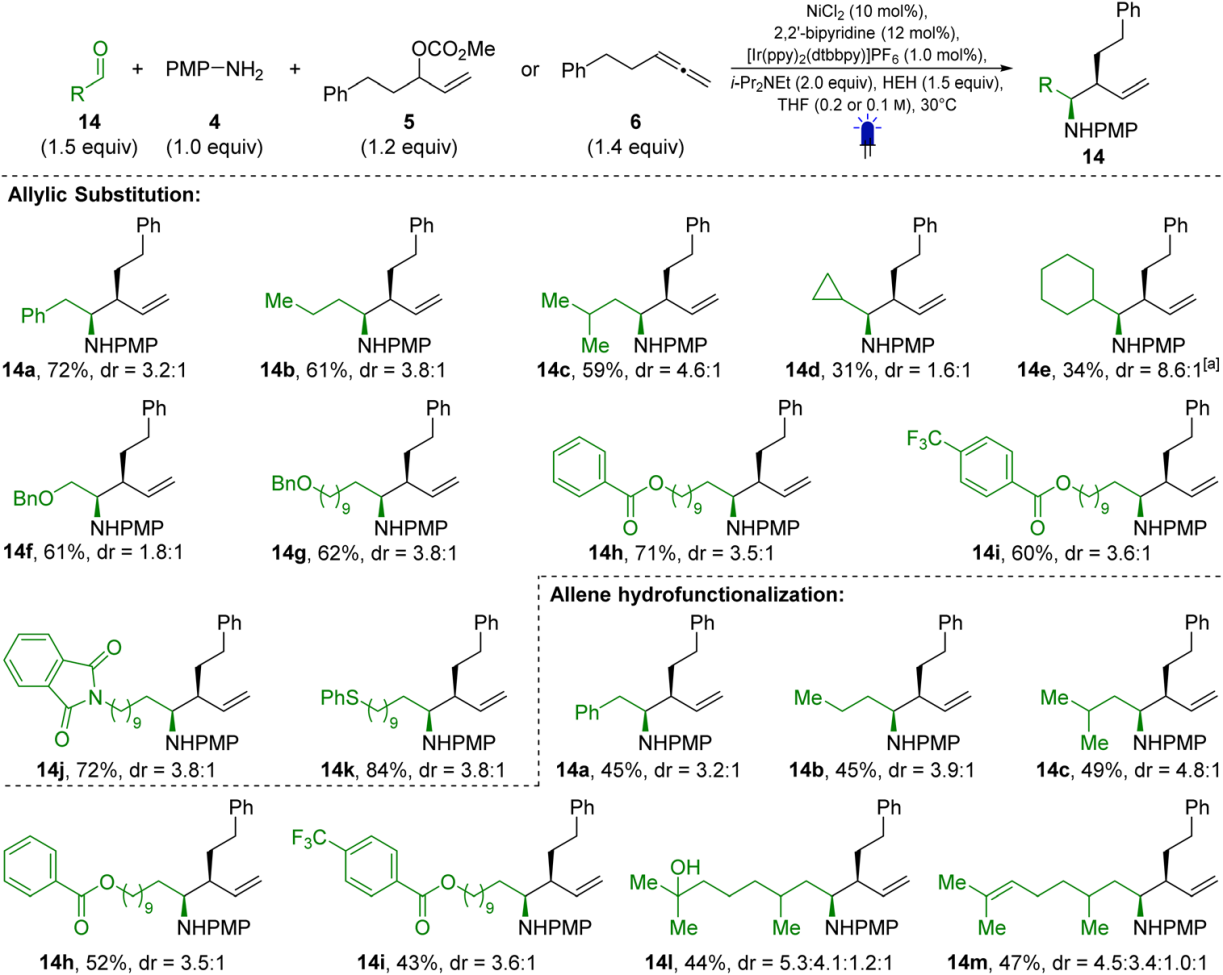
Scope II – aldehydes; ^[a]^ 2.0 equiv. of allylic carbonate were used.

With the optimized conditions at hand, we began investigating the substrate scope (Schemes 2 and 3). The reaction proved tolerant to a wide range of substrate modifications. Allylic carbonates bearing various side chains such as *n*-propyl (13a, 67%, dr = 4.7 : 1 from branched allylic carbonate and 75%, dr = 4.9 : 1 from linear allylic carbonate) or cyclohexyl (13b, 61%, dr = 3.4 : 1), were well tolerated. The parent compound allyl methyl carbonate (R=R′=H) and the tertiary allylic carbonate (R=R′=Me) provided high yields of 87% (13c) and 70% (13d), respectively. Cinnamyl methyl carbonate afforded the corresponding homoallylic amine (13e) in 34% yield with excellent diastereoselectivity (>19 : 1). Moreover, allylic carbonates with silyloxy groups (13f, 83%, dr = 5.0 : 1) and thioether (13g, 52%, dr = 4.8 : 1) gave the corresponding products in good yields. Nitrogen functionalities protected as carbamate (13h, 55%, dr = 8.4 : 1) or phthalimide (13i, 61%, dr = 3.9 : 1) were also compatible with the reaction conditions. Using substrates 13j–13p, we demonstrated that the reaction does not depend on the presence of a methoxy substituent on the aromatic ring. Aniline (13j) as well as derivatives bearing Me (13k), F (13l), Cl (13m) or Br (13n) substituents were found to be suitable reaction partners. *Ortho*-Anisidine (13o) gave a low yield of 23% and a reduced dr of 2.2 : 1. In contrast, *meta*-anisidine (13p) delivered the product in 68% yield with a 4.6 : 1 diastereoselectivity.

Next, we investigated the allene scope (Scheme 2, bottom). Substrate 7, which was previously synthesized from the corresponding allylic carbonate (5) in 80% yield with a dr of 3.8 : 1, was obtained in 68% yield and a dr of 4.1 : 1 when the equivalent allene was used. Propa-1,2-diene can also be used as an allylating agent, delivering the corresponding homoallylic amine (13c) in 45% yield. The diester 13q was obtained in 52% yield with a dr of 16 : 1. The homoallylic amine 13r derived from a 1,1-disubstituted allene was obtained in 68% yield, although without any diastereoselectivity. Free alcohols (13s, 56%, dr = 4.0 : 1), as well as alcohols protected with Bn (13t, 68%, dr = 5.3 : 1), TBS (13u, 62%, dr = 4.3 : 1) and PMP (13v, 51%, dr = 4.9 : 1) groups, were well tolerated under the reaction conditions. An internal allene was also tested and delivered the corresponding homoallylic amine 13w in 22% yield, notably with excellent *syn*/*anti* (>19 : 1) and *E*/*Z* (>19 : 1) selectivity. An allene bearing a phthalimide group in the side chain delivered the 1,5-diamine 13x in 54% yield (dr = 4.5 : 1). The allenes derived from estrone and tyrosine afforded the corresponding products in 66% (13y, dr = 4.9 : 1) and 67% (13z, dr = 4.9 : 1) yield, respectively. Interestingly, the ketone functionality of estrone, despite its potential to form an imine, did not negatively affect the reaction outcome.

The developed protocol also enables the coupling of a broad variety of aldehydes with both allylic carbonates (Scheme 2, top) and allenes (Scheme 3, bottom). Phenylacetaldehyde performed well, affording the allylation product 14a in 72% yield and a dr of 3.2 : 1 when reacted with allylic carbonate 5. Other alkyl substituted aldehydes, such as butyraldehyde (14b, 61%, dr = 3.8 : 1) and isovaleraldehyde (14c, 59%, dr = 4.6 : 1), were also effective substrates. α-Branched aldehydes, including cyclopropyl (14d, 31%, dr = 1.6 : 1) and cyclohexyl aldehyde (14e, 34%, dr = 8.6 : 1) gave lower yields, though the latter showed good diastereoselectivity. The reaction also tolerates the presence of different functional groups on the aldehyde side chain. Benzyl ethers (14f, 61%, dr = 1.8 : 1 and 14g, 62%, dr = 3.8 : 1), esters (14h, 71%, dr = 3.5 : 1 and 14i, 60%, dr = 3.6 : 1), phthalimides (14j, 72%, dr = 3.8 : 1), and thioethers (14k, 84%, dr = 3.8 : 1) were all compatible under the reaction conditions. Unfortunately, benzaldehydes and α,β-unsaturated aldehydes were unreactive (see SI). Using allene 6 as the allylating agent, the corresponding homoallylic amines were also obtained (examples 14a–14c, 14h, 14i). The naturally occurring aldehydes 7-hydroxycitronellal (14l, 44%, dr = 5.3 : 4.1 : 1.2 : 1) and citronellal (14m, 47%, dr = 4.5 : 3.4 : 1.0 : 1) gave comparable results.

**Scheme 4:**
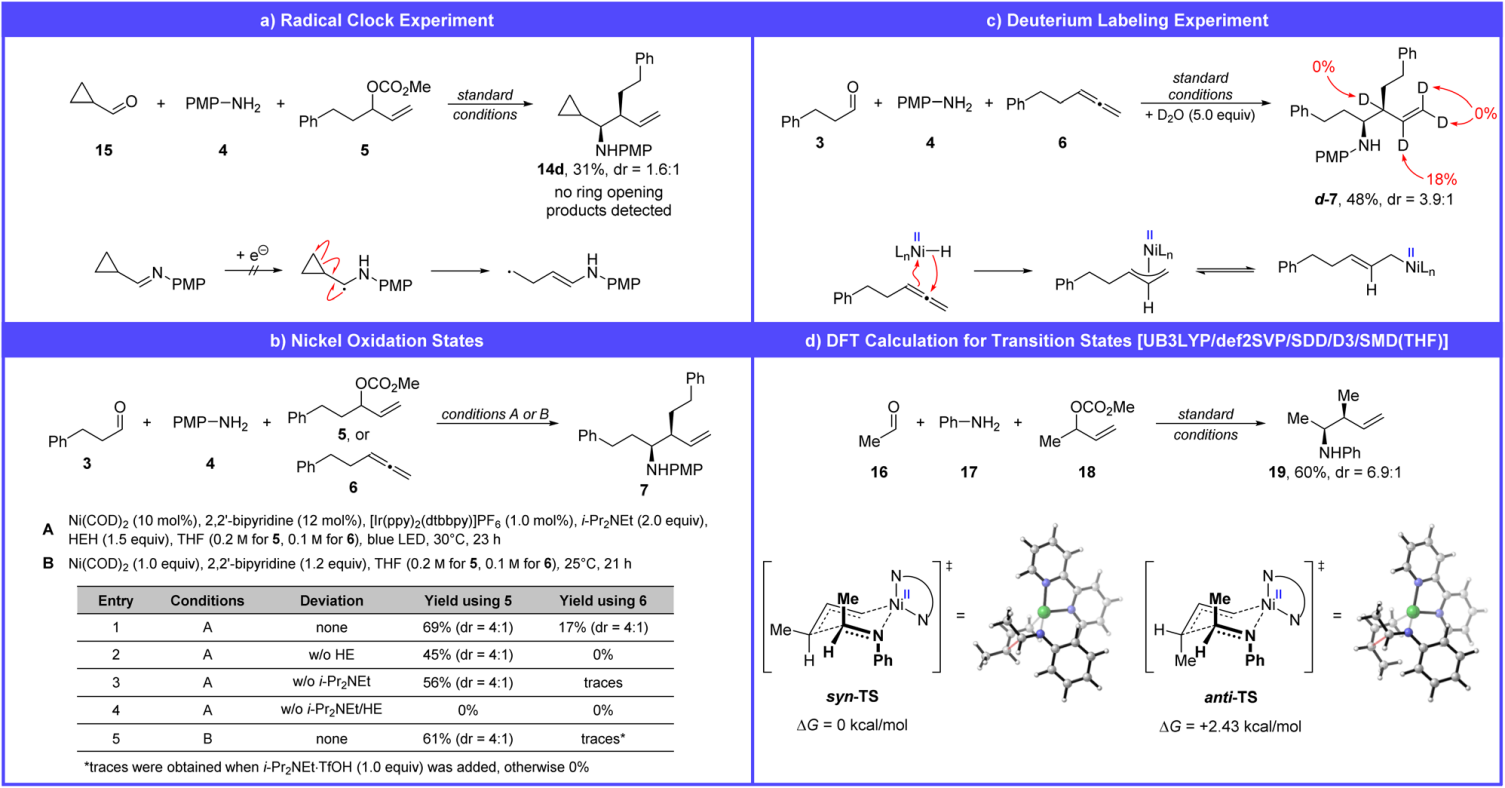
Mechanistic experiments on radical formation (a), nickel oxidation states (b), nickel hydride formation (c), and DFT calculations on transition-state energy (d).

Finally, a series of experiments was conducted to gain insight into the reaction mechanism. Our working hypothesis proposes the formation of an imine as a key intermediate, which is subsequently allylated by an allyl nickel complex. To rule out the involvement of imine reduction to the corresponding α-amino radical, as previously reported,^30^ a radical clock experiment was performed using cyclopropyl carbaldehyde (Scheme 4a). The desired homoallylic amine was obtained in 31% yield, and no ring-opening products were detected.

As a working model for the nickel catalysis, we propose that an oxidative addition (in the case of allylic carbonates) and the formation of a nickel hydride complex (in the case of allenes) are key steps in the reaction mechanism. Nickel catalysts are known for their versatile and complex redox chemistry, which complicates the identification of the oxidation states involved in the product-forming catalytic cycle.^45,51^ To gain insight into this aspect, we conducted a series of experiments using the nickel(0) precatalyst Ni(COD)_2_ (Scheme 4b).

Replacing NiCl_2_ with Ni(COD)_2_ results in 69% of the product for allylic carbonate 5 and 17% for the allene 6 (entry 1). These results support the involvement of nickel(0) as an intermediate in the catalytic cycle of the allylic substitution reaction, likely undergoing oxidative addition with the allylic carbonate (5) to form a π- or σ-allyl nickel(ii) complex. This mechanistic hypothesis is supported by numerous literature reports.^44,45,52,53^ In contrast, the poor performance of the allene under these conditions suggests that nickel(0) may not be a suitable catalyst for this transformation.

Further control experiments in absence of Hantzsch ester (entry 2: 45% for 5, 0% for 6), i-Pr_2_NEt (entry 3: 56% for 5, traces for 6) and both reagents (entry 4: 0% for 5, 0% for 6) highlighted the crucial role of these reagents as electron donors in the catalytic cycle. In case of the allene hydrofunctionalization, both reagents could additionally serve as hydrogen donors.

To further probe the mechanism, we conducted an experiment using 1.0 equiv. of Ni(COD)_2_ and 1.2 equiv. of 2,2′-bipyridine, in the absence of any reducing agents, photocatalyst, or light. Under these conditions, the homoallylic amine (7) was obtained in 61% yield from allylic carbonate 5. This result demonstrates that, once the allyl nickel(ii) complex is formed, it can react with the imine to give the product without requiring further reduction to a potentially more nucleophilic allyl nickel(i) species.

**Scheme 5:**
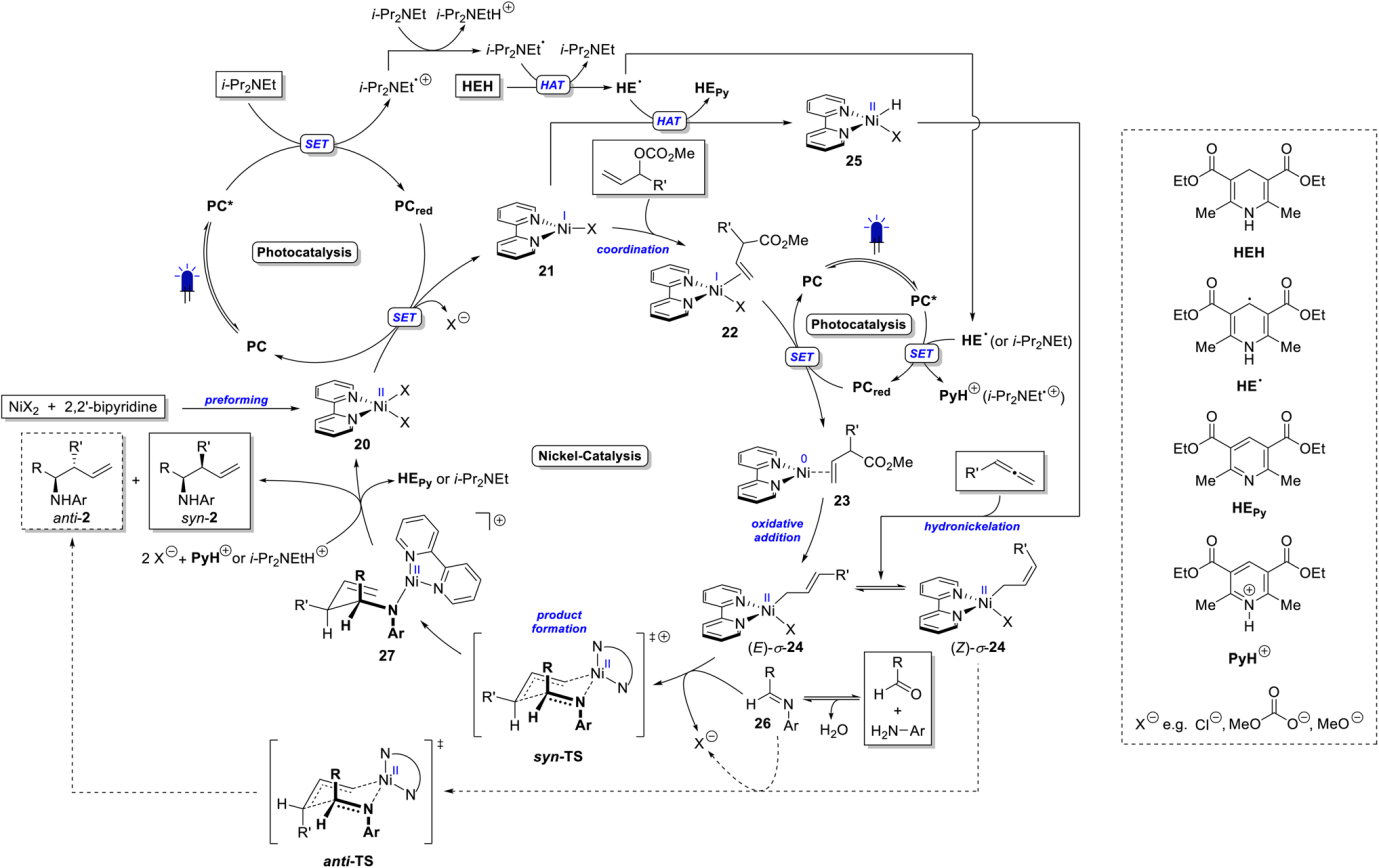
Mechanistic proposal for the dual photoredox/nickel catalyzed synthesis of homoallylic amines from imines and allylic carbonates or allenes.

Under the same conditions, no product was detected when using allene 6. However, upon addition of i-Pr_2_NEt·TfOH (1.0 equiv.) to the allene reaction, traces of product were observed (see SI for details). This suggests that Ni(0) can, in principle, be protonated by i-Pr_2_NEtH^+^ to form a nickel hydride species, although this process appears to be inefficient. As a result, an alternative mechanistic pathway, which is discussed later (Scheme 5), was considered.

To investigate nickel hydride formation, a deuterium-labeling experiment was performed by adding 5.0 equiv. of D_2_O to the reaction mixture (Scheme 4c). Regioselective deuterium incorporation at the formerly sp-hybridized carbon atom of the allene supports the formation of a nickel hydride complex.

The allyl nickel complexes formed from either the allylic carbonate or the allene reductively allylate the imine to form the homoallylic amine. Since the diastereoselectivity of the reaction appears to be largely independent of the chosen allylating agent (Schemes 2 and 3), we propose that both reactions proceed *via* the same transition state. A Zimmerman–Traxler-type transition state was assumed to explain the observed *syn*-selectivity (Scheme 4d). In the proposed *syn*-transition state, one methyl group occupies the disfavored pseudo-axial position, whereas in the *anti*-transition state, two methyl groups are positioned pseudo-axial.

A comparison of the two geometries using DFT calculations revealed a ΔΔ*G* of 2.43 kcal mol^−1^ in favor of the *syn*-transition state. Although this value overestimates the theoretical diastereomeric ratio (98 : 2 according to the Eyring equation) compared to the experimentally observed dr of 6.9 : 1 for 19, the energy difference supports the proposed model and the preferred formation of the *syn*-product.

Based on the made experimental observations, the mechanism proposed in Scheme 5 is postulated. The excited photocatalyst ([Ir(ppy)_2_(dtbbpy)]PF_6_, PC) oxidizes i-Pr_2_NEt, as supported by Stern–Volmer quenching studies described in the SI. This is also consistent with the redox potentials of the photocatalyst [*E*_1/2_(PC*/PC_red_) = +0.66 V *vs.* SCE in MeCN]^54^ and i-Pr_2_NEt [*E*_1/2_(i-Pr_2_NEt˙^+^/i-Pr_2_NEt) = +0.65 V *vs.* SCE in MeCN].^55^

A direct oxidation of Hantzsch ester (HEH) by the photocatalyst seems unlikely since the corresponding Stern–Volmer experiment shows only a weak interaction between PC and HEH [*E*_1/2_(HEH˙^+^/HEH) = +0.89 V *vs.* SCE in MeCN].^56^ Instead, i-Pr_2_NEt˙^+^ formed upon photocatalytic oxidation is deprotonated by a second equivalent of i-Pr_2_NEt to generate the α-amino radical (i-Pr_2_NEt˙). A subsequent hydrogen atom transfer (HAT) reaction with HEH regenerates i-Pr_2_NEt and forms Hantzsch ester radical (HE˙). This step is expected to exhibit a significant driving force, as the unpaired electron in HE˙ is strongly delocalized. HE˙ can undergo a second HAT to form Hantzsch ester pyridine (HE_Py_, isolated as a by-product) or react with the photocatalyst to the protonated Hantzsch ester pyridine [*E*_1/2_(PyH^+^/HE˙) = −0.76 V *vs.* SCE in MeCN].^56^ The electrons collected by the photocatalyst can subsequently be used in the nickel catalytic cycle.

Upon solvation of NiCl_2_ and 2,2′-bipyridine in THF, the bipyridine nickel(ii) complex 20 is formed. The reduced photocatalyst [*E*_1/2_(PC/PC_red_) = −1.51 V *vs.* SCE in MeCN]^54^ can then reduce 20 to the nickel(i) species 21. According to Martin *et al.*, the redox potential of [(bpy)NiBr_2_] is *E*_1/2_(Ni^II^/Ni^I^) = −0.88 V *vs.* SCE in MeCN.^57^ A similar potential is assumed for [(bpy)NiCl_2_] (20), which supports its reduction by PC_red_. From this nickel(i) species, the catalytic cycle takes different pathways depending on the allylating agent. In the case of allylic carbonates, the nickel(i) complex coordinates the carbonate (22), facilitating a subsequent photocatalytic reduction to the nickel(0) species 23.^45^ The reduction of a bipyridine nickel(i) complex to nickel(0) is associated with a redox potential of *E*_1/2_(Ni^I^/Ni^0^) = −1.18 V *vs.* SCE in MeCN [(bpy)Ni(i)Br],^57^ which is accessible by the reduced photocatalyst (PC_red_). The resulting nickel(0) complex 23 can undergo oxidative addition with the allylic carbonate to form the allyl nickel(ii) complex 24.^44,45^24 exists in an equilibrium of the (*E*)-*σ*-allyl and the (*Z*)-*σ*-allyl complex.^42^

For the allene pathway, the nickel(i) complex (21) undergoes hydrogen atom transfer (HAT) with HE˙ to form a nickel(ii) hydride complex (25). Subsequent hydronickelation of the allene yields the same allyl nickel(ii) complex (24) as formed *via* oxidative addition in the allylic carbonate pathway. The fact that Ni(COD)_2_ is only a poor catalyst for the allene hydrofunctionalization suggests that, in this case, the mechanism avoids a nickel(0) intermediate.

In the next step, complex 24 coordinates the imine (26), which is generated *in situ* from the aldehyde and aniline, and undergoes allylation *via* a Zimmerman–Traxler-type transition state. The (*E*)-σ-allyl intermediate leads to the *syn*-diastereoisomer (*syn*-2), while the (*Z*)-σ-allyl gives the *anti*-isomer (*anti*-2). Finally, the resulting product-nickel(ii) complex (27) is protonated by PyH^+^ or i-Pr_2_NEtH^+^, releasing the homoallylic amine (2) and regenerating the nickel(ii) complex 20.

Previous DFT calculations by other groups on the reductive allylation of aldehydes suggest that the allylation step may also proceed *via* a more nucleophilic nickel(i) complex.^44,45^ However, since homoallylic amines can be formed from allyl carbonates in the absence of any reducing agents when a stoichiometric amount of Ni(COD)_2_ is used, we believe that nucleophilic attack by an allyl nickel(ii) species is more likely in our reaction. Notably, the study by Xi *et al.* demonstrates that the oxidation state of the nickel catalyst can vary depending on the substrate.^45^

**Scheme 6:**
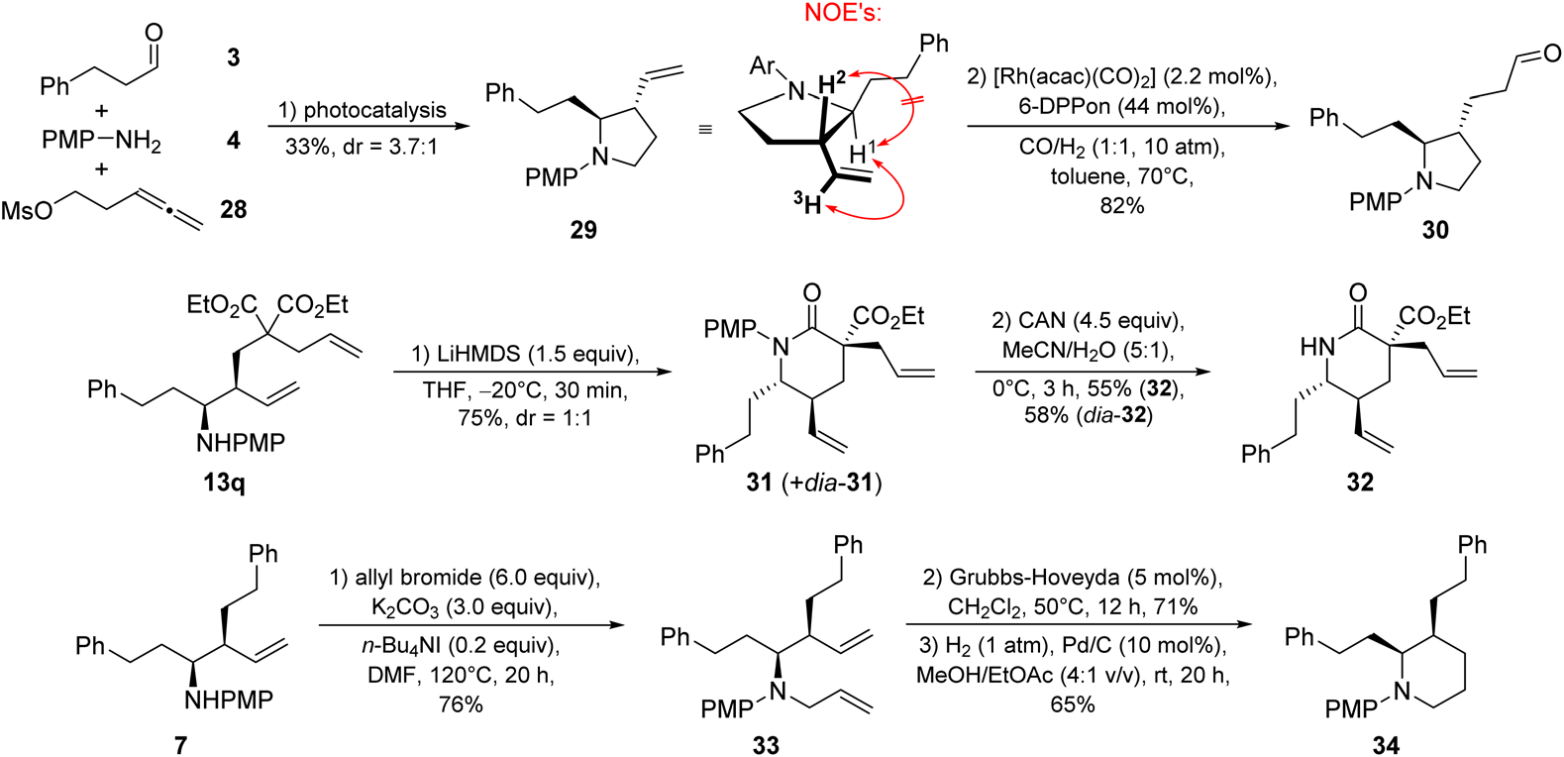
Follow-up chemistry.

A few follow-up transformations were performed to demonstrate the synthetic utility of our method (Scheme 6). When using the leaving group-substituted allene 28, pyrrolidine 29 was obtained directly after the photocatalysis. *Via* NOESY NMR spectroscopy we determined the *syn*-homoallylic amine as the major diastereoisomer. A subsequent hydroformylation of 29 afforded the aldehyde 30 in 82% yield. Furthermore, the lactam 31 was synthesized from the homoallylic amine 13q. The diastereoisomers 31 and *dia*-31 were separated by flash column chromatography and both treated with ceric ammonium nitrate (CAN) to remove the PMP group. Homoallylic amine 7 was *N*-allylated using allyl bromide, yielding the bis-olefin 33 in 76%. A subsequent ring-closing metathesis using the Grubbs–Hoveyda catalyst followed by catalytic hydrogenation furnished piperidine 34.

## Conclusions

In summary, we have developed a diastereoselective three-component coupling protocol for the synthesis of branched *syn*-homoallylic amines from aldehydes, anilines, and allylic carbonates or allenes. This transformation represents the first example of the addition of a photochemically generated nucleophilic allyl metal species to imines. The reaction conditions enable the synthesis of homoallylic amines bearing a wide range of functional groups, including olefins, alcohols, ethers (OBn, OPMP, OTBS), thioethers, ketones, esters, carbamates, phthalimides, and aryl halides (F, Cl, Br). Mechanistic investigations allowed us to propose a detailed catalytic cycle. In the photocatalytic cycle, the photocatalyst transfers electrons from Hantzsch ester and i-Pr_2_NEt to the nickel catalytic cycle. The nickel(ii) precatalyst is reduced to nickel(0), which undergoes oxidative addition with the allylic carbonate to generate an allyl nickel(ii) complex. The same intermediate is accessed from allenes *via* a nickel hydride species. This allyl–nickel complex then reacts with the imine, formed *in situ*, through a six-membered Zimmerman–Traxler-type transition state to yield the homoallylic amine. The preference for the *syn*-diastereomer is supported by DFT calculations.

Finally, follow-up transformations were conducted to showcase the synthetic utility of the products. These included hydroformylation, amide coupling, PMP deprotection, *N*-allylation, ring-closing metathesis, and catalytic hydrogenation, yielding valuable building blocks for further applications.

## Author contributions

C. N. conceptualized the project, carried out the synthesis of starting materials, reaction optimization, substrate scope studies, mechanistic investigations, and follow-up chemistry. C. N. also performed data analysis and wrote the manuscript. N. M., B. G., and F. H. assisted with starting material synthesis and reaction optimization. Furthermore, N. M. contributed to manuscript revision. F. B. performed the DFT calculations. B. B. supervised all aspects of the project and revised the manuscript.

## Conflicts of interest

There are no conflicts to declare.

## Supplementary Material

SC-016-D5SC06916E-s001

## Data Availability

All obtained data are described either in the manuscript or the supplementary information (SI). Supplementary information: further information, including general experimental information, optimization studies, detailed experimental procedures, compound characterization data and NMR spectra of new compounds. See DOI: https://doi.org/10.1039/d5sc06916e.
