# Supplementary material for: Photocatalytic synthesis of homoallylic amines via nucleophilic addition of nickel allyl complexes to imines
Source: Chem Sci. 2025 Oct 9;16(44):21047–55. doi: 10.1039/d5sc06916e (PMC12517050; doi:10.1039/d5sc06916e)
Supplement: SC-016-D5SC06916E-s001 [file SC-016-D5SC06916E-s001.pdf]

Supplementary Information (SI) for Chemical Science.
This journal is © The Royal Society of Chemistry 2025

Photocatalytic Synthesis of Homoallylic Amines via Nucleophilic Addition of Nickel Allyl Complexes to Imines

Christoph Nopper,[a] Niclas Müller,[a] Beloslava Goycheva,[a] Felix Himmelsbach,[a] Felix Bauer,[a] and Bernhard Breit*[a]

[a] Institut für Organische Chemie, Albert-Ludwigs-Universität Freiburg, Albertstraße 21,

79104 Freiburg im Breisgau, Germany

E-mail: bernhard.breit@chemie.uni-freiburg.de

Table of Contents

1. General Methods and Materials 2

2. Substrate Synthesis 4

2.1 Anilines 4

2.2 Allylic Carbonates 4

2.3 Allenes 7

2.4 Aldehydes 8

3. Photocatalytic Reactions 16

3.1 Experimental Setup 16

3.2 Reaction Optimization 17

3.3 Allylic Carbonate Scope 25

3.4 Aniline Scope 32

3.5 Allene Scope 36

3.6 Aldehyde Scope for Allylic Carbonates 43

3.7 Aldehyde Scope for Allenes 51

3.8 Failed Substrates 54

4. Scale-Up 54

5. Mechanistic Experiments 55

5.1 Control experiments 55

5.2 Detection of Radical Intermediates 56

5.3 Nickel oxidation states 57

5.4 Deuterium labeling 60

5.5 Stern Volmer Quenching 61

6. Follow-Up Chemistry 65

7. Determination of Main Diastereoisomer 73

8. DFT Calculations 74

9. Literature 78

10. NMR-Spectra 80

1. General Methods and Materials

Reagents were purchased from commercial suppliers and used as received unless noted otherwise.

Reactions were carried out in dry glassware under an argon atmosphere (Argon 5.0, Sauerstoffwerk Friedrichshafen). For this, the glassware was dried by heat gun under high vacuum (oil pump, 0.1 mbar), cooled to room temperature and backfilled with argon. An argon atmosphere in the reaction vessel was maintained throughout the reaction, unless noted otherwise. For the addition of solvents and reagents, syringes and cannula were flushed with argon three times prior to use. All yields are isolated yields, unless noted otherwise. For optimization studies in catalytic reactions, yields were determined from the noted ^{1}H-NMR spectrum of the crude product using 1,3,5-trimethoxybezene (TMB) as an internal standard.

Solvents were bought in p.a. quality and used without further purification. Dry solvents for air/moisture sensitive reactions were bought from commercial suppliers and used as received. Solvents were evaporated at 40°C under reduced pressure using a HEIDOLPH LABORATA 4001 rotatory evaporator system and a rotary vane pump ($\geq$ 8 mbar) from VACCUBRAND GMBH & CO. KG.

Thin layer chromatography (TLC) was performed on MACHERY-NAGEL silica gel 60 F254 aluminium plates (0.24 mm layer thickness). Compounds were visualized using UV lights (λ = 254 nm) or by applying common staining solution and heating:

$KMnO_4$ stain: $KMnO_4$ (3.00 g), Na_2CO_3 (20.0 g), NaOH (5% w/v, 5.00 mL) in H_2O (300 mL).

phosphomolybdic acid stain: PMA (5.0 g) and AcOH (5.0 mL) in EtOH (250 mL).

Flash column chromatography was carried out using standard glass columns packed with a plug of cotton wool, sea sand (1-2 cm), silica gel 60 (MACHERY-NAGEL, 0.04-0.063 mm, 230-240 mesh) and sea sand (1-2 cm).

Nuclear magnetic resonance (NMR) spectra were measured on BRUKER Avance III HD 300 MHz, BRUKER Avance Neo 400 MHz, BRUKER Avance 500 MHz and BRUKER Avance Neo 700 MHz spectrometers. ^{1}H-NMR spectra were measured at 300, 400, 500 or 700 MHz, ^{13}C-NMR spectra were measured at 101, 126 or 176 MHz, ^{19}F spectra were measured at 282 MHz, 271 or 659 MHz. All signals are referenced to the signal of the deuterated solvent (^{1}H-NMR: CH_3Cl_3, $\delta = 7.26$ ppm; ^{13}C-NMR: $CDCl_3$, $\delta = 77.1$ ppm). ^{13}C-NMR spectra are ^{1}H broad band decoupled. Measurements on the BRUKER Avance Neo 400 MHz, BRUKER Avance 500 MHz and BRUKER Avance Neo 700 MHz were performed in the institute´s analytical department by DR. M. KELLER, DR. S. BRAUKMÜLLER or M. SCHONHARD. NMR data are reported as follows:

chemical shift (δ/ppm), multiplicity (s: singlet; d: doublet; t: triplet; q: quartet; p: pentet; m: multiplet; br. s: broad signal), coupling constants (J/Hz), integration).

High resolution mass spectrometry (HRMS) experiments were performed on an Thermo Fisher Scientific Exactive mass spectrometer (Orbitrap instrument). Samples were infused directly or via a LC/MS setup. Ionization was achieved by electrospray ionization (ESI, needle voltage 2.5-5.0 kV, ion transfer tube 250°C, sheath/auxiliary gas N_2) or atmospheric pressure chemical ionization (APCI, corona needle current 5-10 μA, vaporizer temperature 50-400°C, sheath gas N_2, auxiliary gas N_2/NH_3). HRMS data are reported as follows: chemical formula, $[ion]^{charge}$, ionization method (ESI or APCI in positive or negative mode): calculated value, found value, deviation (Δ/ppm).

Gas chromatography-mass spectrometry (GC-MS) experiments were performed on a Thermo Scientific ISW/Trace 1300 Quadruple GC-MS. CI/EI ionization and ammonia as reagent gas were used. For better resolution also HRMS of the samples were measured.

2. Substrate Synthesis

2.1 Anilines

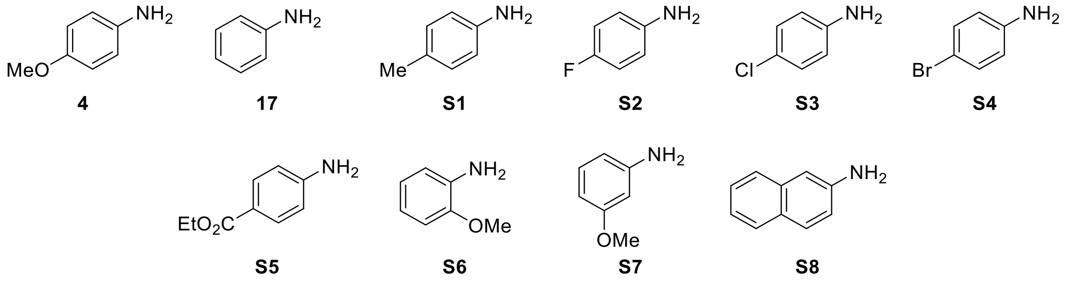

All Anilines (**4, 17, S1**-**S8**) were purchased from commercial suppliers. Solid anilines were recrystallized before use, liquid anilines were filtered over a plug of silica.

2.2 Allylic Carbonates

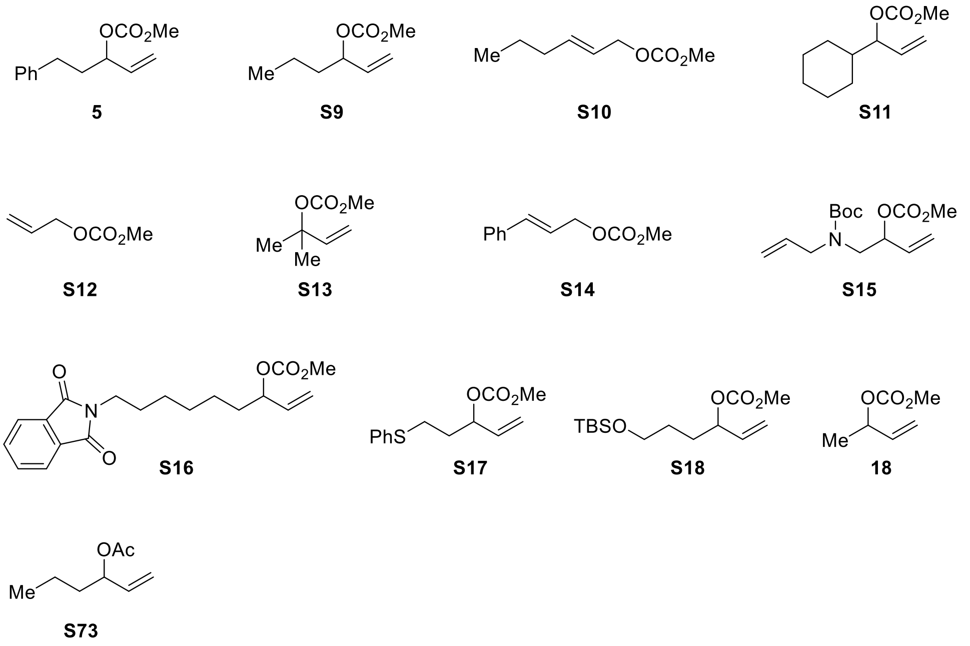

Allyl methyl carbonate (**S12**) was purchased from Acros. All other compounds were similarly synthesized by addition of vinyl magnesium bromide to the respective aldehyde and subsequent esterification with methyl chloroformate. A general procedure was published before.[1] Where commercially available, the allylic alcohol was bought. Analytical date were in accordance with literature: **5**[2], **S9**[3], **S10**[4], **S11**[5], **S13**[6], **S14**[4], **S15**[1], **S17**[1], **S18**[7], **18**[8]

Synthesis and analytical data for **S73** were described previously.[9]

Synthesis of S16:

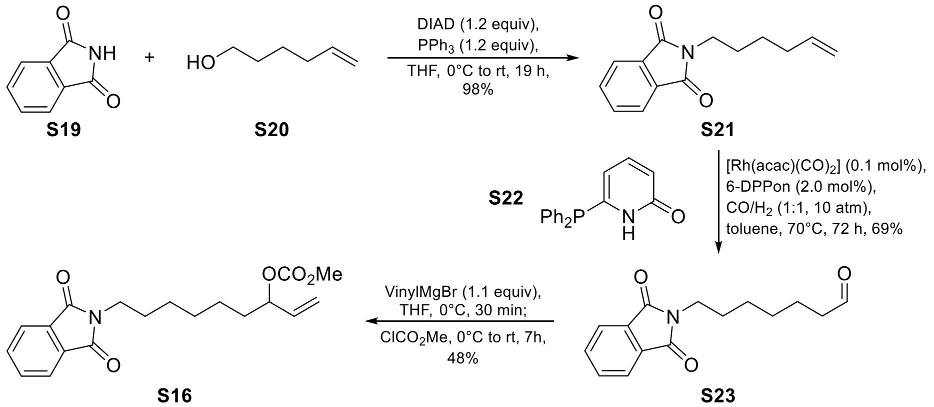

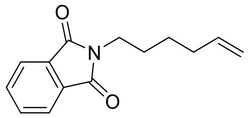

S21

A stirred solution of PPh_3 (99%, 9.54 g, 36.0 mmol, 1.2 equiv), 5-hexenol (**S20**, 99%, 3.66 mL, 3.04 g, 36.0 mmol, 1.2 equiv) and phthalimide (**S19**, 5.30 g, 36.0 mmol, 1.2 equiv) in dry THF (60 mL) was cooled to 0°C. DIAD (98%, 7.21 mL, 7.43 g, 36.0 mmol, 1.2 equiv) was added and the reaction mixture was stirred at rt for 19 h. Then, the solvent was removed under reduced pressure and the residue was purified by flash column chromatography [silica gel, *n*-pentane/EtOAc 19:1 to 9:1 v/v, R_f = 0.28 (*n*-pentane/EtOAc 9:1 v/v)]. The product (**S21**) was obtained as a colorless oil (6.75 g, 29.5 mmol, 98%). Analytical data were in accordance with literature.[10]

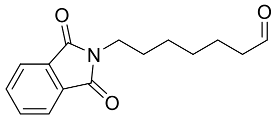

S23

[Rh(acac)(CO)$_2$] (1.55 mg, 6.00 µmol, 0.1 mol%) and 6-DPPon (**S22**, 33.5 mg, 120 µmol, 2.0 mol%) were added to a previously dried 20 mL glass inlet equipped with a cross-shaped magnetic stirring bar. The glass vial was closed with an aluminum crimp seal with septum and alternately put on vacuum and argon three times before adding dry toluene (8.6 mL, 0.7 M) and olefin **S21** (1.38 g, 6.00 mmol, 1.0 equiv). Afterwards, the glass inlet fitted with a small cannula was inserted into a steel autoclave, which was then sealed. The closed autoclave was then purged three times with CO/H_2 gas (1:1, 5 bar), followed by adjustment of the reaction pressure to 10 bar. A detailed description of the reaction setup is provided in chapter 2.4. The reaction mixture was stirred in the autoclave for 72 h at 70°C. Afterwards, the autoclave was allowed to cool to rt and depressurized. The solvent was removed *in*

vacuo and the residue was purified by flash column chromatography [silica gel, *n*-pentane/Et_2O 3:1 to 1:1 v/v, R_f = 0.28 (*n*-pentane/Et_2O 1:1 v/v)]. Aldehyde **S23** (1.07 g, 4.13 mmol, 69%) was obtained as an oily liquid.

^{1}H-NMR (500 MHz, $CDCl_3$): δ = 1.37 (m, 4H), 1.66 (m, 4H), 2.42 (td, *J* = 7.3, 1.8 Hz, 2H), 3.68 (m, 2H), 7.71 (m, 2H), 7.84 (m, 2H), 9.75 (t, *J* = 1.8 Hz, 1H) ppm.

^{13}C-NMR (126 MHz, $CDCl_3$): δ = 22.0, 26.6, 28.5, 28.7, 37.9, 43.8, 123.3, 132.2, 134.0, 168.5, 202.6 ppm.

HRMS ($C_{15}H_{17}NO_3Na$, $[M + Na]^+$, pos. ESI): *m/z*: calcd 282.1101, found 282.1095, Δ = –1.9 ppm.

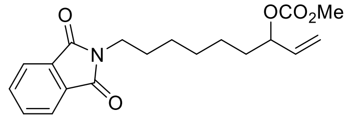

S16

Aldehyde **S23** (1.11 g, 4.71 mmol, 1.0 equiv) was dissolved in dry THF (10 mL) and cooled to 0°C under an argon atmosphere. Vinyl magnesium bromide (0.7 M in THF, 6.73 mL, 4.71 mmol, 1.1 equiv) was added dropwise via syringe pump over 15 min. Subsequently, the mixture was stirred at 0°C for 45 min. Then, methyl chloroformate (502 µL, 613 mg, 6.42 mmol, 1.5 equiv) was added dropwise by syringe. The resulting mixture was warmed to room temperature and stirred for 7 hours. Afterwards, sat. aq. NH_4Cl solution (10 mL) was added carefully and the mixture was extracted with Et_2O (3 × 20 mL). The combined organic layers were dried over Na_2SO_4 and concentrated under vacuum. The crude product was purified by flash column chromatography [silica gel, *n*-pentane/Et_2O 3:1 to 2:1 v/v, R_f = 0.11 (*n*-pentane/Et_2O 3:1 v/v)]. The product (**S16**, 712 mg, 2.06 mmol, 48%) was obtained as a pale yellow oil.

^{1}H-NMR (500 MHz, $CDCl_3$): δ = 1.34 (m, 6H), 1.64 (m, 4H), 3.67 (m, 2H), 3.76 (s, 3H), 5.02 (m, 1H), 5.19 (ddd, *J* = 10.5, 1.2 Hz, 1H), 5.28 (ddd, *J* = 17.2, 1.2 Hz, 1H), 5.77 (ddd, *J* = 17.2, 10.5, 6.8 Hz, 1H), 7.70 (m, 2H), 7.83 (m, 2H) ppm.

^{13}C-NMR (126 MHz, $CDCl_3$): δ = 24.9, 26.8, 28.6, 29.0, 34.2, 38.0, 54.7, 79.2, 117.5, 123.2, 132.2, 133.9, 136.1, 155.3, 168.5 ppm.

HRMS ($C_{19}H_{23}NO_5Na$, $[M + Na]^+$, pos. ESI): *m/z*: calcd 368.1468, found 368.1476, Δ = 2.2 ppm.

2.3 Allenes

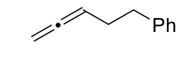

6

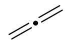

S24

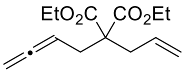

S25

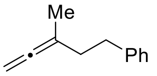

S26

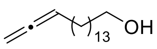

S27

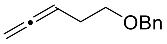

S28

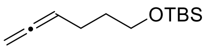

S29

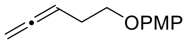

S30

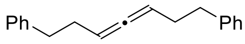

S31

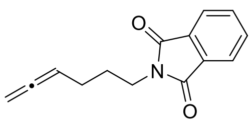

S32

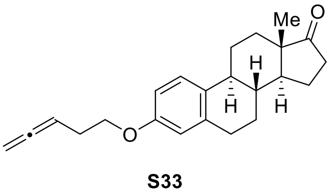

S33

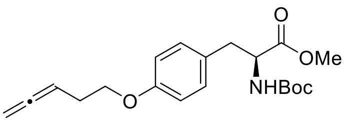

S34

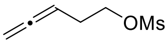

28

Propa-1,2-diene (**S24**) was bought in a 5 kg gas bottle from Air Liquide. A balloon was filled with propa-1,2-diene (ca. 500 mL) and the gas was bubbled through 10 mL of dry THF resulting in a 1.9 M propa-1,2-diene solution that was used for the photocatalysis.

The allenes **6**, **S28**, **S33**, **S34**[11], **S25**[12], **S26**[13], **S27**[14], **S29**[15], **S31**[16], **S32**[17], **28**[18] were synthesized as described previously.

Synthesis of S30:

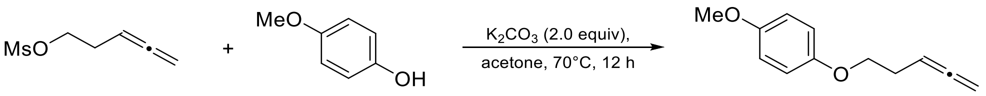

28 **S35** **S30**

p-Methoxyphenol (**S35**, 98%, 950 mg, 7.50 mmol, 1.5 equiv), K_2CO_3 (1.38 g, 10.0 mmol, 2.0 equiv) and mesylat **28**[18] (811 mg, 5.00 mmol, 1.0 equiv) were dissolved in dry acetone (10 mL) and stirred at 70°C for 21 h. After completion of the reaction (TLC), the mixture was evaporated under reduced pressure. The residue was mixed with H_2O (20 mL) and extracted with EtOAc (3 × 50 mL). The solvent was evaporated under reduced pressure and the crude product was purified by flash column chromatography [silica gel, *n*-pentane/EtOAc 29:1 v/v, R_f = 0.25 (*n*-pentane/Et_2O 19:1 v/v)]. Allene **S30** (698 mg, 3.67 mmol, 73%) was obtained as a colorless oil.

^{1}H-NMR (500 MHz, $CDCl_3$): δ = 2.47 (dtd, *J* = 9.9, 6.7, 3.1 Hz, 2H), 3.77 (s, 3H), 3.99 (t, *J* = 6.7 Hz, 2H), 4.72 (m, 2H), 5.22 (tt, *J* = 6.8 Hz, 1H), 6.84 (m, 4H) ppm.

^{13}C-NMR (126 MHz, $CDCl_3$): δ = 28.5, 55.8, 75.3, 86.4, 114.7, 115.7, 153.1, 153.9, 209.1 ppm.

HRMS ($C_{12}H_{15}O_2$, $[M + H]^+$, pos. APCI): *m/z*: calcd 191.1067, found 191.1069, Δ = 1.1 ppm.

2.4 Aldehydes

Commercially available:

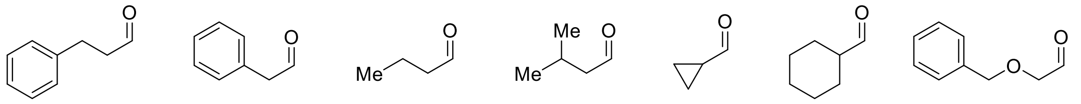

3 **S36** **S37** **S38** **15** **S39** **S40**

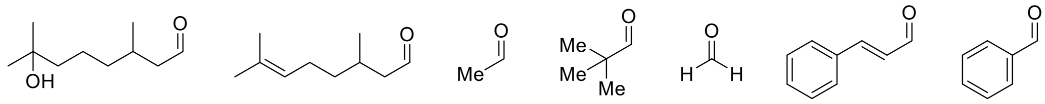

S41 **S42** **16** **S43** **S44** **S45** **S46**

Synthesized:

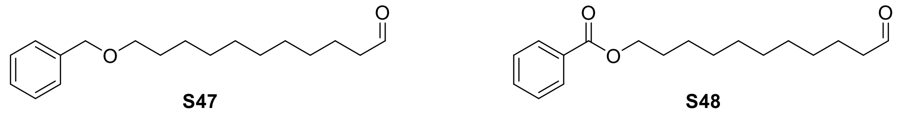

S47 **S48**

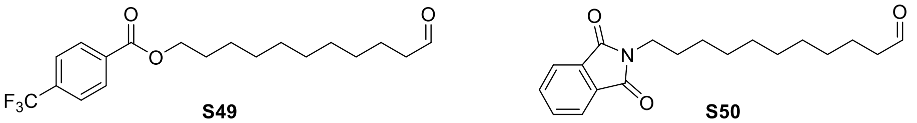

S49 **S50**

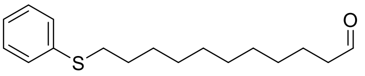

S51

Aldehydes from commercial suppliers were distilled prior to use. Synthesized aldehydes were purified by flash column chromatography.

Aldehyde synthesis:

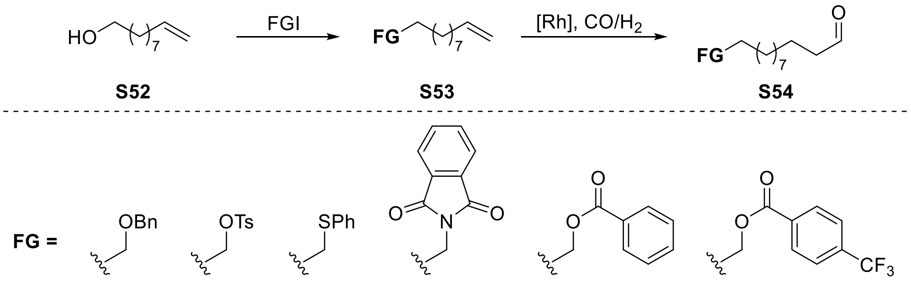

Alcohol functionalization:

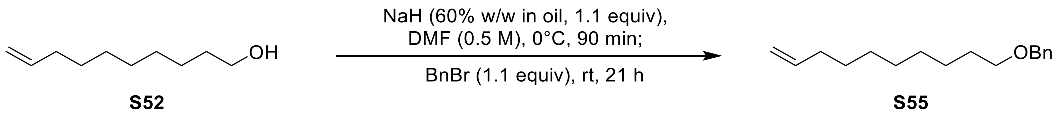

9-decen-1-ol (**S52**, 97%, 915 µL, 773 mg, 4.80 mmol, 1.0 equiv) in dry DMF (5 mL) was added to a solution of NaH (60% w/w in oil, 212 mg, 5.28 mmol, 1.1 equiv) in dry DMF (5 mL) at 0°C. The mixture was stirred for 25 min, then at rt for 90 min, cooled to 0°C and treated with BnBr (520 µL, 903 mg, 5.28 mmol, 1.1 equiv). After 15 min, the mixture was allowed to warm to rt and then stirred for additional 21 h. Following addition of Et_2O (15 mL) and washing with sat aq NH_4Cl-solution (3 × 10 mL), the combined organics were dried over $MgSO_4$. After the solvent was removed *in vacuo*, the residue was purified by flash column chromatography [silica gel, *n*-pentane/EtOAc 49:1 → 29:1 v/v, R_f = 0.48 (*n*-pentane/EtOAc 19:1 v/v)] to afford the product as a colorless oil (**S55**, 622 mg, 2.52 mmol, 53%).

This procedure is based on a previously reported one. Analytical data are in accordance with literature.[19]

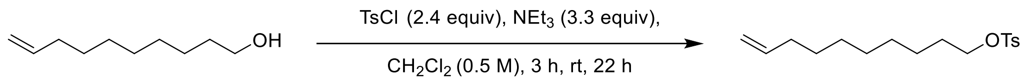

S52 **S56**

At 0°C *p*-TsCl (98%, 6.99 g, 36.0 mmol, 2.4 equiv) was added portionwise to a solution of 9-decen-1-ol (**S52**, 97%, 2.41 g, 2.86 mL, 15.0 mmol, 1.0 equiv) in dry CH_2Cl_2 (30 mL). The mixture was stirred at 0°C, then allowed to warm to ambient temperature and stirred at rt for 22 h. The solution was quenched with H_2O (10 mL), the aq. layer was separated and extracted with Et_2O (3 × 10 mL). The combined organic layers were washed with brine (10 mL), dried over Na_2SO_4 and the solvent was evaporated *in vacuo*. The crude product was purified by flash column chromatography [silica gel, *n*-pentane/EtOAc 19:1 → 9:1 v/v, R_f = 0.62 (*n*-pentane/EtOAc 3:1 v/v)] to afford the product as a colorless oil (**S56**, 58% pure, 6.82 g, 12.7 mmol, 85%) which remained contaminated with TsCl.

This procedure is based on a previously reported one.[11] Analytical data are in accordance with literature.[20]

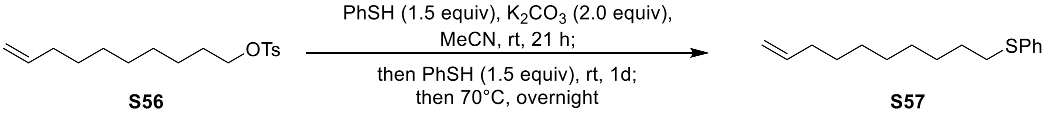

S56 **S57**

A solution of tosylate **S56** (58%, 2.85 mL, 3.21 g, 6.00 mmol, 1.0 equiv), thiophenol (924 µL, 1.00 g, 9.00 mmol, 1.5 equiv) and K_2CO_3 (98%, 2.57 g, 18.0 mmol, 3.0 equiv) in dry MeCN (24 mL, 0.25 M) was stirred at rt for 21 h. The reaction progress was checked via TLC. Remaining TsCl from the previous step required the addition of more thiophenol (924 µL, 1.00 g, 9.00 mmol, 1.5 equiv). After stirring for another 24 h, the conversion was still incomplete. Hence, the solution was stirred at 70°C overnight. Subsequently, the solvent was evaporated under reduced pressure, and the residue was mixed with H_2O (10 mL), and extracted with EtOAc (3 × 10 mL). The organic phase was dried over Na_2SO_4, the

solvent was removed in vacuo and the residue was purified via flash column chromatography [silica gel, *n*-pentane → *n*-pentane/EtOAc 10:1 v/v, R_f = 0.34 (*n*-pentane)] to afford the product as a colorless oil (**S57**, 501 mg, 2.02 mmol, 34%).

^{1}H-NMR (400 MHz, $CDCl_3$): δ = 1.37 (m, 6H), 1.65 (m, 2H), 2.04 (m, 2H), 2.92 (m, 2H), 4.97 (m, 2H), 5.82 (ddt, *J* = 16.9, 10.2, 6.7 Hz, 1H), 7.16 (m, 1H), 7.30 (m, 4H) ppm.

^{13}C-NMR (101 MHz, $CDCl_3$): δ = 139.2, 137.2, 129.0, 128.9, 125.7, 114.2, 33.9, 33.7, 29.4, 29.3, 29.2, 29.1, 29.0, 28.9 ppm.

HRMS ($C_{16}H_{25}S$, $[M + H]^+$, pos. APCI): *m/z*: calcd 249.1671, found 249.1678, Δ = 2.4 ppm.

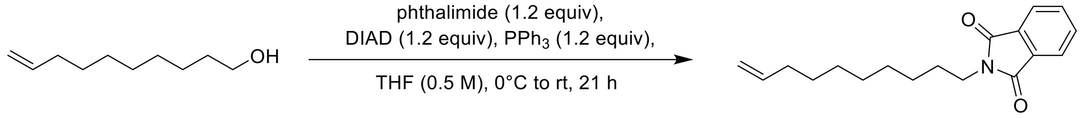

S52 **S58**

A stirred solution of PPh_3 (99.5%, 3.17 g, 12.0 mmol, 1.2 equiv), 9-decen-1-ol (**S52**, 97%, 1.61 g, 1.89 mL, 10.0 mmol, 1.0 equiv) and phthalimide (1.77 g, 12.0 mmol, 1.2 equiv) in dry THF (20 mL) was cooled to 0°C. DIAD (99%, 2.40 mL, 2.46 g, 12.0 mmol, 1.2 equiv) was added and the reaction mixture was stirred at rt for 21 h. Then, the solvent was removed *in vacuo* and the residue was purified by flash column chromatography [silica gel, *n*-pentane/EtOAc 19:1 → 4:1 v/v, R_f = 0.45 (*n*-pentane/EtOAc 9:1 v/v)] to afford the product as a colorless oil (**S58**, 2.64 g, 9.25 mmol, 93%).

This procedure is based on a previously reported one.[11] Analytical data are in accordance with literature.[21]

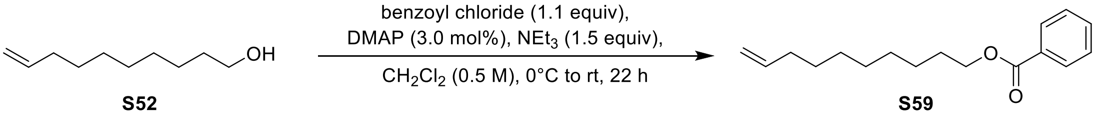

S52 **S59**

9-decen-1-ol (**S52**, 97%, 1.91 mL, 1.61 g, 10.0 mmol, 1.0 equiv), benzoyl chloride (99%, 1.21 mL, 1.46 g, 11.0 mmol, 1.1 equiv), and 4-dimethylaminopyridine (DMAP, 36.7 mg, 0.3 mmol, 3.0 mol%) were dissolved in dry CH_2Cl_2 (20 mL). The reaction mixture was cooled to 0°C, NEt_3 (2.10 mL, 1.52 g, 15.0 mmol, 1.5 equiv) was added dropwise over 5 min. The reaction was stirred for 22 h while the ice bath slowly thawed.. The organic layer was washed with aq. HCl-solution (1 M, 2 × 20 mL), water (2 × 20 mL), and saturated aq. NaCl (1 × 20 mL). The organic phase was dried over Na_2SO_4 and the solvent was removed *in vacuo*. The residue was purified via flash column chromatography [silica gel, *n*-pentane/EtOAc 29:1 → 19:1 v/v, R_f = 0.63 (*n*-pentane/EtOAc 19:1 v/v)] to afford the product as a colorless oil (**S59**, 2.29 g, 8.79 mmol, 88%).

This procedure is based on a previously reported one.[22] Analytical data are in accordance with literature.[23]

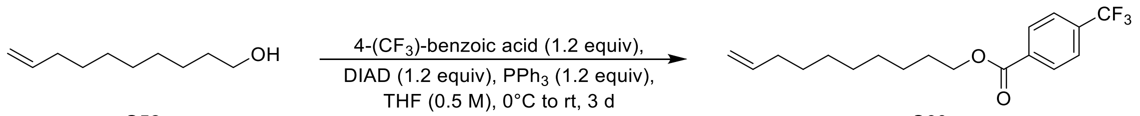

S52 S60

A stirred solution of PPh_3 (99.5%, 3.17 g, 12.0 mmol, 1.2 equiv), 9-decen-1-ol (**S52**, 1.89 mL, 1.61 g, 10.0 mmol, 1.0 equiv) and 4-(trifluoromethyl)benzoic acid (98%, 2.32 g, 12.0 mmol, 1.2 equiv) in dry THF (20 mL) was cooled to 0°C. DIAD (99%, 2.40 mL, 2.46 g, 12.0 mmol, 1.2 equiv) was added and the reaction mixture was stirred at rt for 3 d. Then, the solvent was removed under reduced pressure and the residue was purified by flash column chromatography [silica gel, *n*-pentane/EtOAc 39:1→ 29:1 v/v, R_f = 0.34 (*n*-pentane/EtOAc 29:1 v/v)]. The product was obtained as a colorless oil (**S60**, 2.85 g, 8.69 mmol, 87%).

^{1}H-NMR (700 MHz, $CDCl_3$): δ = 1.35 (m, 8H), 1.44 (m, 2H), 1.78 (m, 2H), 2.04 (m, 2H), 4.35 (t, *J* = 6.7 Hz, 2H), 4.96 (m, 2H), 5.81 (ddt, *J* = 16.9, 10.2 Hz, 6.7, 1H), 7.70 (m, 2H), 8.15 (m, 2H) ppm.

^{13}C-NMR (176 MHz, $CDCl_3$): δ = 26.1, 28.7, 29.0, 29.1, 29.3, 29.4, 33.9, 65.8, 114.3, 123.8 (q, *J* = 272.8 Hz), 125.5 (q, *J* = 3.8 Hz,), 130.0, 133.9, 134.4 (q, *J* = 33.4 Hz), 139.2, 165.5 ppm.

^{19}F-NMR (659 MHz, $CDCl_3$): δ = –63.1 ppm.

HRMS ($C_{18}H_{24}F_3O_2$, $[M + H]^+$, pos. APCI): *m/z*: calcd 329.1723, found 329.1718, Δ = –1.4 ppm.

Olefin Hydroformylation

Two methods developed in our group were applied to synthesize aldehydes from the previously functionalized olefins. The room temperature ambient pressure (RTAP) hydroformylation does not need autoclave equipment, however requires higher catalyst loadings and longer reaction times.

Hydroformylation reactions under RTAP conditions were conducted with a carbon monoxide/hydrogen mixture (CO/H_2 = 1:1, H_2 4.3, CO 3.7, "CRYSTAL") from Air Liquide. The gas mixture was filled in a standard laboratory grade balloon fitted with a three-way glass valve connected to the balloon, a high vacuum pump and the reaction vessel. A Schlenk-tube with a height of 10 cm and a diameter of 4 cm was sealed with a rubber septum, alternately put on vacuum and argon three times. Then, the reaction mixture was added and the Schlenk flask was flushed with syngas. The experimental setup is shown in Figure 1.[24]

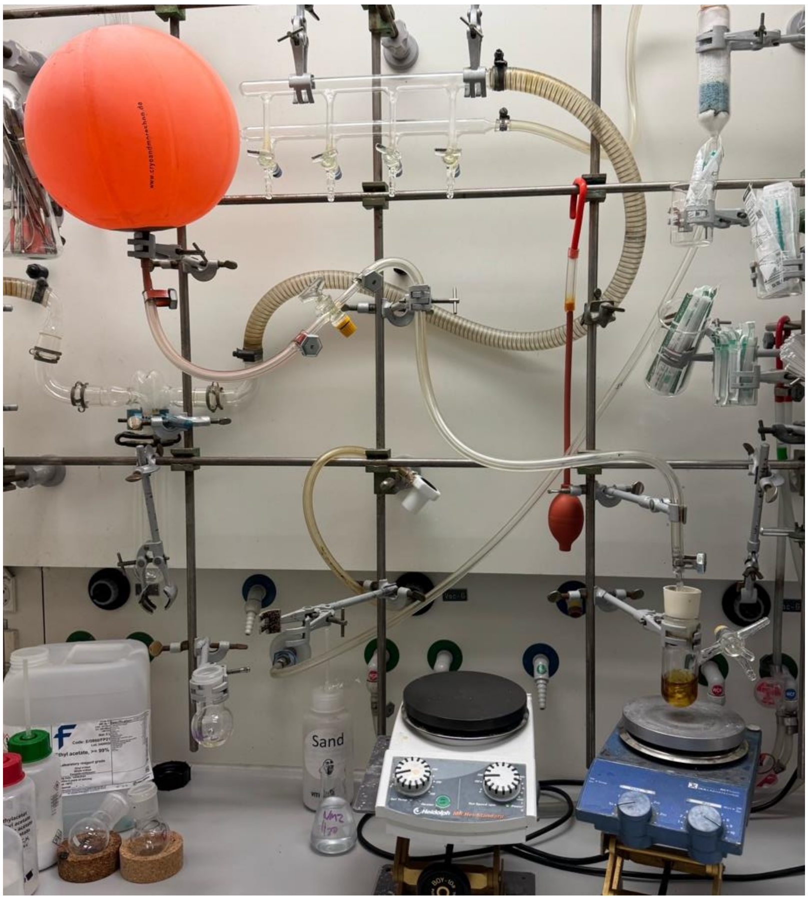

Figure 1: Experimental setup for RTAP hydroformylation.

Hydroformylation reactions under pressure were performed with a carbon monoxide/hydrogen mixture (CO/H_2 = 1:1, H_2 4.3, CO 3.7, "CRYSTAL") from Air Liquide. The reactions were done in a stainless-steel autoclave (self-build by the workshop of the chemical institute, V_{max} = 100 mL, p_{max} = 45 bar, heated through a fitting aluminium block, stirred magnetically. The autoclave headpiece was fitted with a pressure gauge, pressure relief valve, vent valve, Teflon flat seal and bayonet connection). The glass inlets used for the reactions were stored in a drying oven at 120°C. The bottom part of the autoclave was stored in a drying oven at 50°C, while the headpieces of the autoclaves were stored at rt. The dried glass inlets were sealed after addition of all chemicals with a silicon/PTFE cap ND20 with a crimp plier. During the reaction the reaction mixture was stirred magnetically with a cross shaped magnetic stirring bar (high speed helpful) and heated by an aluminium heating block. The seals were removed and disposed of after completed reaction. The experimental setup is depicted in Figure 2.

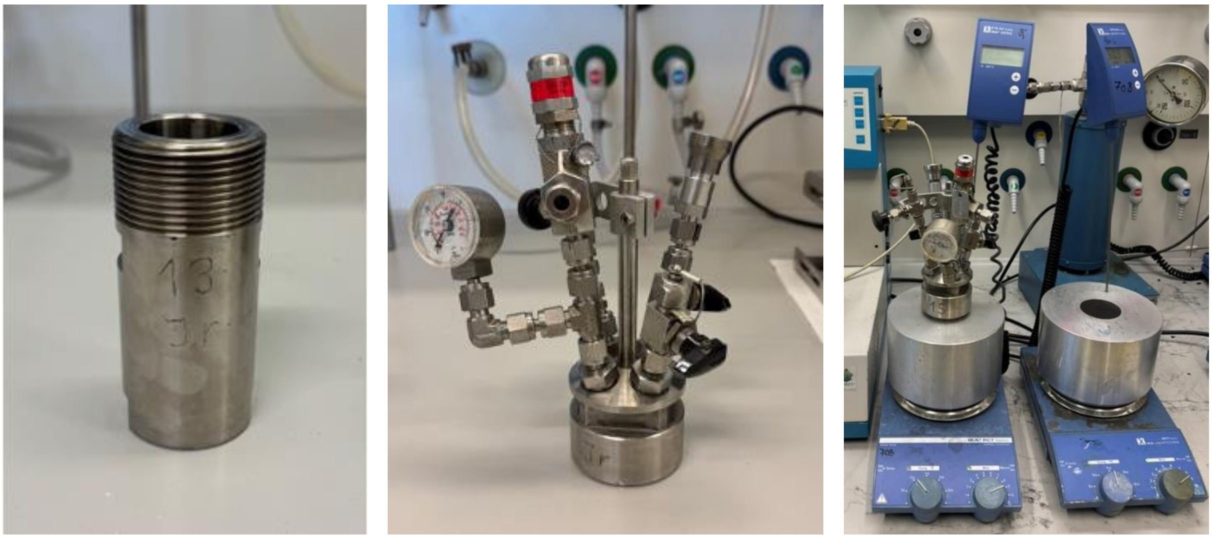

Figure 2: Autoclave parts and reaction setup in aluminum block.

General procedure A: RTAP-Hydroformylation

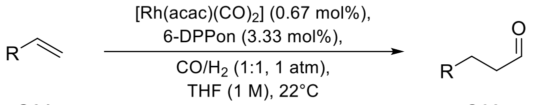

S61 **S62**

A Schlenk flask was charged with the olefin (**S61**, 1.0 equiv), [Rh(*acac*)$(CO)_2$] (0.67 mol%) and 6-diphenylphosphinopyridin-2-(1H)-one (6-DPPon, 3.33 mol%) in dry THF (1 M). The Schlenk flask was flushed with synthesis gas [CO/H_2 (1:1), 1 atm] with a cannula inserted through a septum. The solution was magnetically stirred using a cross-type stirring bar at rt for 23-44 h (depending on the conversion as determined by TLC, the balloon had to be refilled). The solvent was removed under reduced pressure and the crude product was purified by flash column chromatography.

This procedure is based on a previously reported one.[24,25]

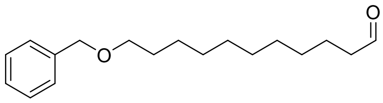

S47

S47 was synthesized according to general procedure A using alkene **S55** (608 mg, 2.47 mmol, 1.0 equiv) and was stirred for 44 h. Flash column chromatography: silica gel, *n*-pentane/EtOAc 29:1 → 9:1 v/v, R_f = 0.23 (*n*-pentane/EtOAc 19:1 v/v). The product was obtained as a colorless oil (**S47**, 355 mg, 1.28 mmol, 52%).

Analytical data are in accordance with literature.[10]

General procedure B: Autoclave Hydroformylation

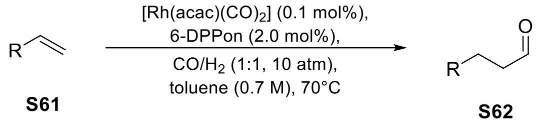

A mixture of [Rh(*acac*)$(CO)_2$] (0.5 mg, 2.00 µmol, 0.1 mol%) and 6-diphenylphosphinopyridin-2-(1*H*)-one (6-DPPon, 11.2 mg, 40.0 µmol, 2.0 mol%) was added to a previously dried 20 mL glass inlet equipped with a cross-shaped magnetic stirring bar. The glass vial was closed with an aluminum crimp seal with septum and alternately put on vacuum and argon three times before adding dry toluene (2.9 mL, 0.7 M) and the olefin (**S61**, 2.00 mmol, 1.0 equiv). Afterwards, the glass inlet fitted with a small cannula was inserted into a steel autoclave which was then sealed. The closed autoclave was then purged three times with CO/H_2-gas (1:1, 5 bar), followed by the adjustment of the reaction pressure to 10 bar. The reaction mixture was stirred in the autoclave for 19-72 h at 70°C. Afterwards, the autoclave was allowed to cool to rt and depressurized. The solvent was removed *in vacuo* and the residue was purified by flash column chromatography.

This procedure is based on a previously reported one.[26]

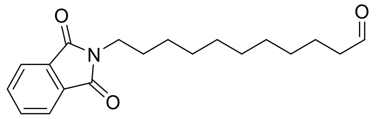

S50

Hydroformylation of **S58** (546 µL, 571 mg, 2.00 mmol, 1.0 equiv) using general procedure C (RTAP) resulted in incomplete conversion. Full conversion was achieved by transfer into an autoclave together with fresh [Rh(*acac*)$(CO)_2$] and 6-DPPon according to general procedure B. Flash column chromatography: silica gel, *n*-pentane/EtOAc 19:1 → 4:1 v/v, R_f = 0.16 (*n*-pentane/EtOAc 9:1 v/v). The product was obtained as a colorless oil (**S50**, 487 mg, 1.54 mmol, 77%).

Analytical data are in accordance with literature.[21]

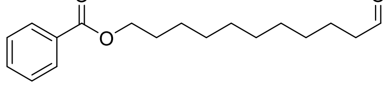

S48

S48 was synthesized according to general procedure B using alkene **S59** (529 µL, 531 mg, 2.0 mmol, 1.0 equiv) and was stirred for 70 h. Flash column chromatography: silica gel, *n*-pentane/EtOAc 29:1 →

4:1 v/v, R_f = 0.19 (*n*-pentane/EtOAc 19:1 v/v). The product was obtained as a colorless oil (**S48**, 538 mg, 1.95 mmol, 98%).

Analytical data are in accordance with literature.[25]

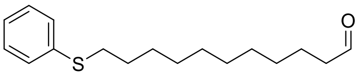

S51

S51 was synthesized according to general procedure B using alkene **S57** (461 mg, 1.85 mmol, 1.0 equiv), [Rh(*acac*)(CO)$_2$] (0.5 mg, 1.85 µmol, 0.1 mol%), 6-DPPon (1.2 mg, 37.0 µmol, 2.0 mol%) and was stirred for 19 h. Flash column chromatography: silica gel, *n*-pentane/EtOAc 9:1, R_f = 0.42 (*n*-pentane/EtOAc 9:1 v/v). The product was obtained as a colorless oil (**S51**, 404 mg, 1.45 mmol, 78%).

^{1}H-NMR (700 MHz, CDCl$_3$): δ = 1.30 (m, 10H), 1.41 (m, 2H), 1.64 (m, 4H), 2.41 (td, *J* = 7.4, 1.9 Hz, 2H), 2.91 (m, 2H), 7.16 (m, 1H), 7.27 (m, 2H), 7.32 (m, 2H), 9.76 (t, *J* = 1.9Hz, 1H) ppm.

^{13}C-NMR (176 MHz, CDCl$_3$): δ = 22.2, 28.9, 29.2, 29.2, 29.4, 29.5, 33.7, 44.0, 125.7, 128.9, 128.9, 137.1, 203.0 ppm.

HRMS ($C_{17}H_{27}OS$, [M + H]$^+$, pos. APCI): *m/z*: calcd 279.1777, found 279.1778 , Δ = 0.4 ppm.

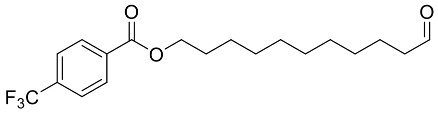

S49

S49 was synthesized according to general procedure B using alkene **S60** (657 mg, 2.0 mmol, 1.0 equiv) and was stirred for 21 h. Flash column chromatography: silica gel, *n*-pentane/EtOAc 29:1 → 19:1 v/v, R_f = 0.23 (*n*-pentane/EtOAc 19:1 v/v). The product was obtained as a colorless oil (**S49**, 582 mg, 1.62 mmol, 81%).

^{1}H-NMR (400 MHz, CDCl$_3$): δ = 1.33 (m, 10H), 1.43 (dtd, *J* = 9.5, 8.4, 6.2 Hz, 2H), 1.62 (p, *J* = 7.3 Hz, 2H), 1.77 (m, 2H), 2.41 (td, *J* = 7.3, 1.9 Hz, 2H), 4.34 (t, *J* = 6.7 Hz, 2H), 7.70 (m, 2H), 8.15 (m, 2H), 9.76 (t, *J* = 1.8, 1H) ppm.

^{13}C-NMR (101 MHz, CDCl$_3$): δ = 22.1, 26.1, 28.7, 29.2, 29.3, 29.4, 29.5, 44.0, 65.8, 123.7 (q, *J* = 272.8 Hz), 125.5 (q, *J* = 3.8 Hz), 130.0, 133.8, 134.4 (q, *J* = 32.4 Hz), 165.5, 202.9 ppm.

^{19}F-NMR (271 MHz, CDCl$_3$): δ = –63.12 ppm.

HRMS ($C_{19}H_{26}F_3O_3$, [M + H]$^+$, pos. APCI): *m/z*: calcd 359.1829, found 359.1832 , Δ = 1.0 ppm.

3. Photocatalytic Reactions

3.1 Experimental Setup

For photocatalytic reactions a photoreactor was build according to figure 3. As a blue light source FAVOLCANO® DC 12V blue LED strips (4.8 W/m, λ_{max} = 452 nm) were used. The strips (approx. 220 cm) were fixed on the inside of a glass bowl and the bowl was subsequently wrapped with aluminum foil to minimize light intensity loss. The photocatalyzed reactions were carried out in 10 mL Schlenk tubes as shown in figure 3. The tubes were placed close to the LED strips with the aid of a metal rack and stirred with a magnetic stirrer. A computer fan attached to a metal can was used to dissipate the heat produced by the LEDs. A reaction temperature of approximately 30°C could be maintained.

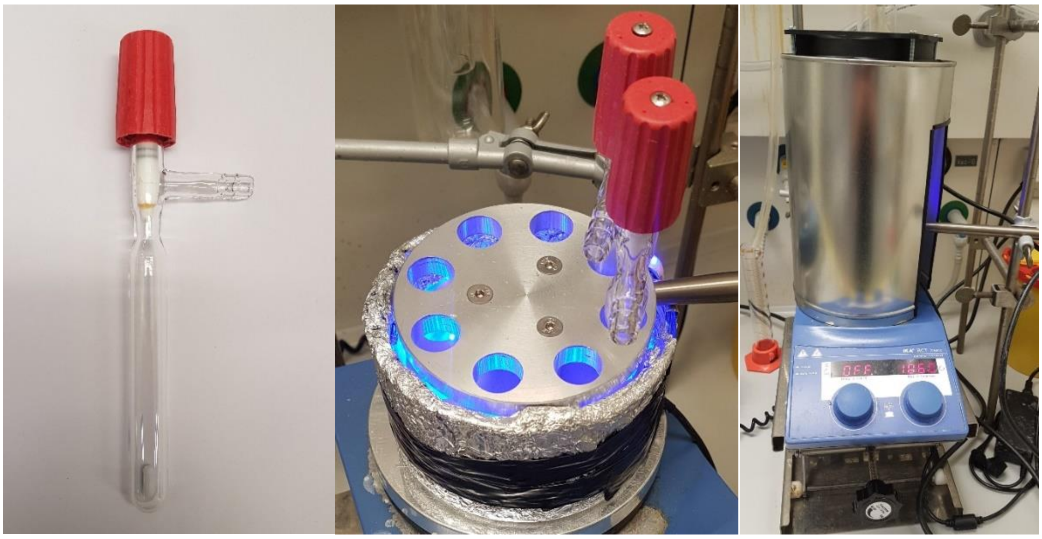

Figure 3: Experimental setup – 10 mL Schlenk tube (left), Photoreactor and metal rack with (right) and without (middle) ventilation system.

General Procedure C:

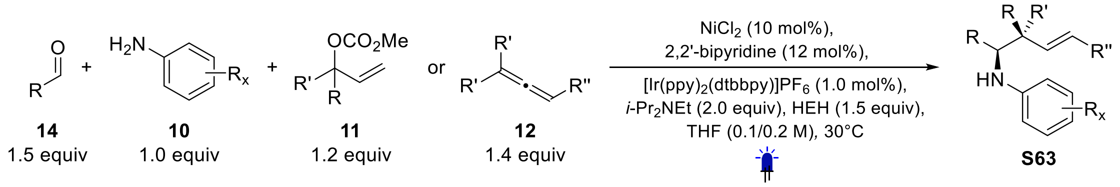

A 10 ml Schlenk tube was dried by heat gun under vacuum, backfilled with argon and cooled to room temperature using a standard Schlenk line apparatus. The Schlenk tube was charged with $NiCl_2$ (2.60 mg, 20.0 µmol, 10 mol%) and 2,2'-bipyridine (3.70 mg, 24.0 µmol, 12 mol%) and alternately put on vacuum and argon three times. Afterwards dry THF [1 mL for allyl carbonates (0.2 M), 2 mL for allenes (0.1 M)] was added by syringe under a flow of argon and the solution was stirred for 30 min.

Then, the aniline (**10**, 200 µmol, 1.0 equiv), [Ir(ppy)$_2$(dtbbpy)]PF$_6$ (1.80 mg, 2.00 µmol, 1.0 mol%), Hantzsch ester (76.0 mg, 300 µmol, 1.5 equiv), the aldehyde (**14**, 300 µmol, 1.5 equiv), *i*-Pr$_2$NEt (68.0 µL, 51.8 mg, 400 µmol, 2.0 equiv) and the allylic carbonate (**11**, 240 µmol, 1.2 equiv) or the allene (**12**, 280 µmol, 1.4 equiv) were added to the Schlenk tube. The tube was sealed by a screw cap and placed approximately 3 cm from blue led strips. The tube was irradiated and stirred for 18-24 h. Afterwards, the reaction mixture was filtered over silica and the solvent was evaporated under reduced pressure. The crude product was analyzed by ^{1}H-NMR spectroscopy (for optimization reactions 1,3,5-trimethoxybenzene (5-10 mg) was added). The homoallylic amines were isolated by flash column chromatography using silica as stationary phase and *n*-pentane/Et$_2$O mixtures as mobile phase. In most cases further purification with reversed-phase chromatography (H$_2$O/MeCN) was necessary. Reversed phase column chromatography was performed on a puriFlash 430 from Interchim using a PF-15C18AQ-F0012 column (cv = 19 mL) with a flow rate of 15.0 mL/min. The following method was used:

- column equilibration (H$_2$O/MeCN 95:5 v/v) for 2 column volumes
- sample injection
- linear gradient from 95:5 to 0:100 v/v H$_2$O/MeCN over 10 column volumes
- 100% MeCN for 6 column volumes

Comment on NMR evaluation:

The pure homoallylic amines were analyzed by NMR spectroscopy. Since the obtained diastereoisomers were not separable, the NMR spectra contained two sets of partially overlapping signals. When both NMR signals were properly resolved, integral values were given as decimal numbers, e.g., for a given proton 6.75 (ddd, 0.84H) and 6.93 (ddd, 0.16H) for the same proton of the diastereoisomer (dr = 5.3:1).

3.2 Reaction Optimization

Reactions were optimized based on NMR yields measuring the crude product in presence of 1,3,5-trimethoxybenzene as internal standard. Due to the fact that the reaction mixture contained, next to two product diastereoisomers, many different reactants and reagents it was often challenging to find product signals for precise integration in the crude ^{1}H-NMR spectrum to calculate the yield. Hence, NMR yields often overestimated the actual yields, however, still allowed for a reliable reaction optimization. Protons that gave the most reliable integral values are colorized in the structure below. Other factors such as appearance of the crude NMR spectrum or side-product formation were also taken into account to decide with which conditions optimizations should be continued.

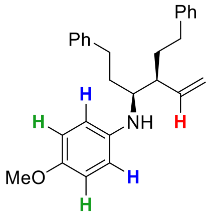

7

Allylic Carbonates:

Solvent:

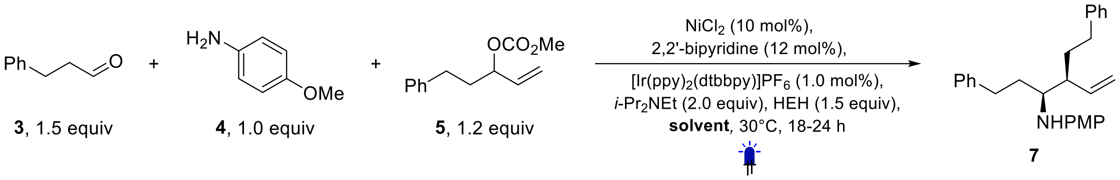

Entry	Solvent	NMR yield	dr
1	1,4-dioxane	91%	4:1
2	1,4-dioxane[a]	81%	4:1
3	1,4-dioxane[b]	93%	4:1
4	THF	90%	4:1
5	THF[b]	92%	4:1
**6**	**THF[c]**	**93%**	**4:1**
7	MeCN	28%	4:1
8	Toluene	63%	4:1
9	Acetone	71%	4:1

Reaction conditions: aldehyde (**3**, 1.5 equiv), *p*-anisidine (**4**, 200 µmol, 1.0 equiv), allylic carbonate (**5**, 1.2 equiv), $NiCl_2$ (10 mol%), 2,2’-bipyridine (12 mol%), $[Ir(ppy)_2(dtbbpy)]PF_6$ (1.0 mol%), *i*-Pr_2NEt (2.0 equiv), Hantzsch ester (1.5 equiv), solvent (0.1 M), 30°C, blue LEDs, 18-24 h. [a] w/o Ni catalyst preforming; [b] 1.4 equiv allylic carbonate; [c] c = 0.2 M; Yields and diastereoselectivities were calculated from the ^{1}H-NMR spectrum of the crude product. 1,3,5-Trimethoxybenzene was used as an internal standard.

Nickel catalyst:

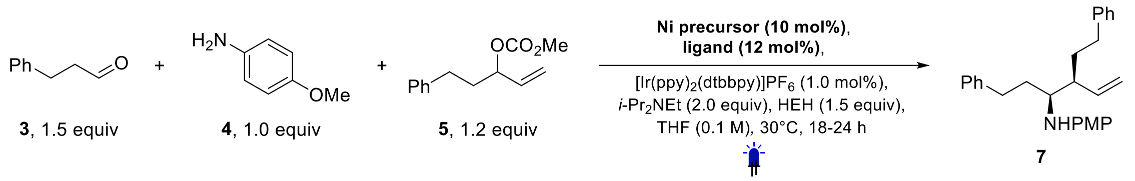

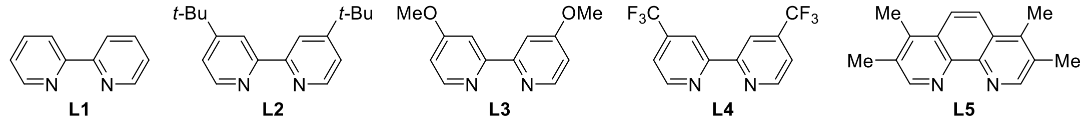

Entry	[Ni]	Ligand	NMR yield	dr
**1**	**$NiCl_2$**	**L1**	**90%**	**4:1**
2	$NiCl_2$(glyme)	L1	46%	4:1
3	$Ni(COD)_2$	L1	79%	4:1
4	$Ni(acac)_2$	L1	83%	4:1
5	$Ni(NO_3)_2 \cdot 6H_2O$	L1	36%	4:1
6	$NiCl_2$	L2	92%	4:1
7	$NiCl_2$	L3	25%	4:1
8	$NiCl_2$	L4	89%	4:1
9	$NiCl_2$	L5	29%	4:1

Reaction conditions: aldehyde (**3**, 1.5 equiv), *p*-anisidine (**4**, 200 µmol, 1.0 equiv), allylic carbonate (**5**, 1.2 equiv), [Ni] (10 mol%), ligand (12 mol%), $[Ir(ppy)_2(dtbbpy)]PF_6$ (1.0 mol%), *i*-Pr_2NEt (2.0 equiv), Hantzsch ester (1.5 equiv), THF (0.1 M), 30°C, blue LEDs, 18-24 h. Yields and diastereoselectivities were calculated from the ^{1}H-NMR spectrum of the crude product. 1,3,5-Trimethoxybenzene was used as an internal standard.

Photocatalyst:

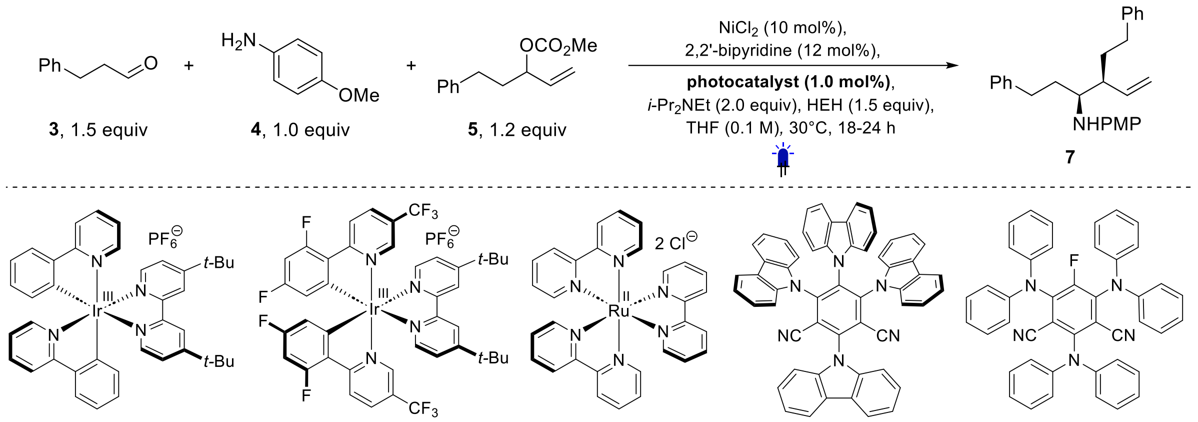

Entry	Photocatalyst	NMR yield	dr
**1**	**PC1 (9)**	**90%**	**4:1**
2	**PC2**	ca. 40%	ca. 4:1
3	**PC3**	0%	–
4	**PC4**	traces	–
5	**PC5**	ca. 28%	ca. 4:1

Reaction conditions: aldehyde (**3**, 1.5 equiv), *p*-anisidine (**4**, 200 µmol, 1.0 equiv), allylic carbonate (**5**, 1.2 equiv), $NiCl_2$ (10 mol%), 2,2'-bipyridine (12 mol%), **PC** (1.0 mol%), *i*-Pr_2NEt (2.0 equiv), Hantzsch ester (1.5 equiv), THF (0.1 M), 30°C, blue LEDs, 18-24 h. Yields and diastereoselectivities were calculated from the ^{1}H-NMR spectrum of the crude product. 1,3,5-Trimethoxybenzene was used as an internal standard.

Chiral ligands:

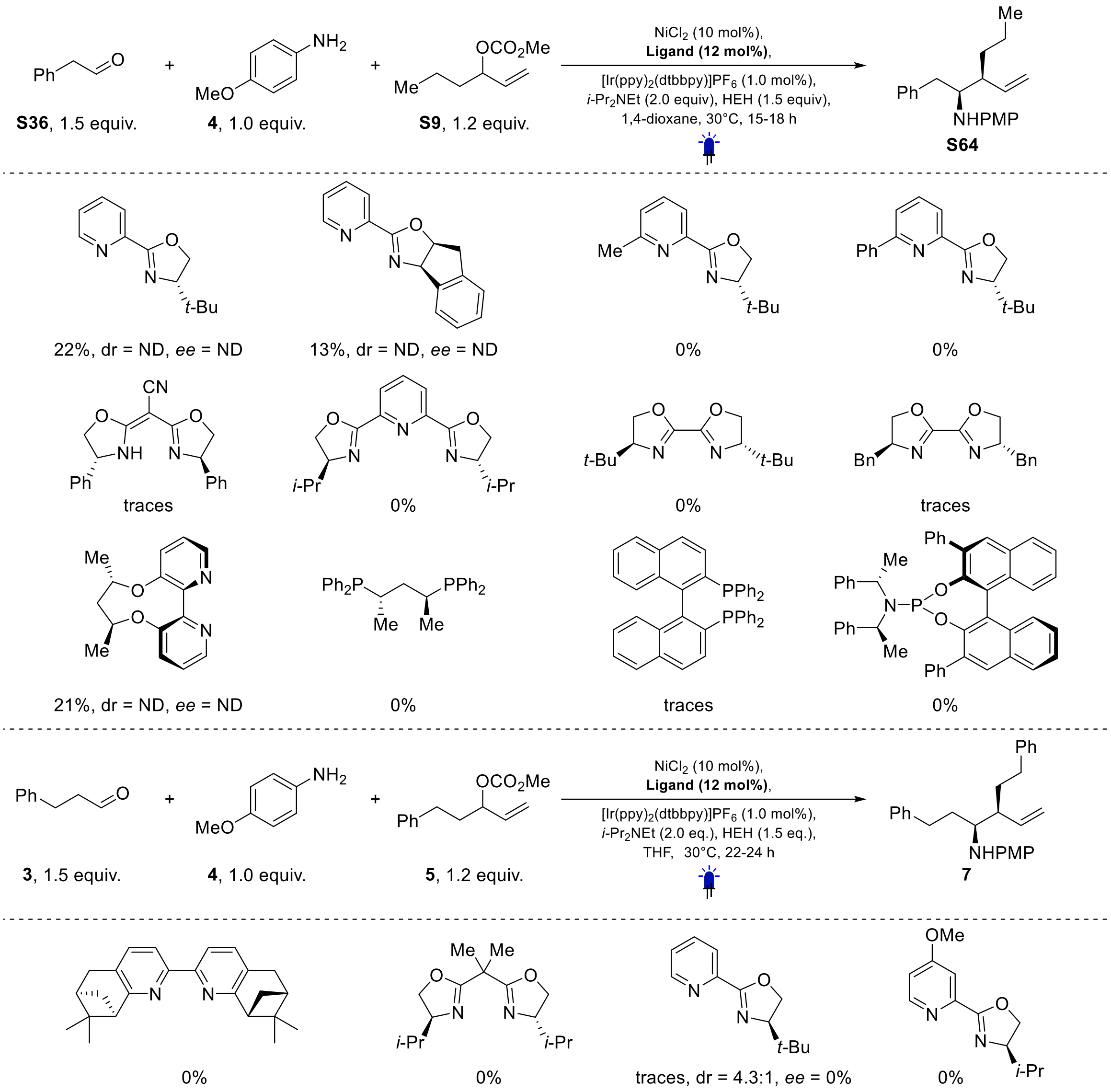

Screening of chiral ligands. Reaction conditions: aldehyde (**S36** or **3**, 1.5 equiv), *p*-anisidine (**4**, 200 μmol, 1.0 equiv), allylic carbonate (**S9** or **5**, 1.2 equiv), $NiCl_2$ (10 mol%), ligand (12 mol%), $[Ir(ppy)_2(dtbbpy)]PF_6$ (1.0 mol%), *i*-Pr_2NEt (2.0 equiv), Hantzsch ester (1.5 equiv), THF or 1,4-dioxane (0.1 M), 30°C, blue LEDs, 15-24 h. Yields and diastereoselectivities were calculated from the ^{1}H-NMR spectrum of the crude product. 1,3,5-Trimethoxybenzene was used as an internal standard.

Allenes:

Solvent:

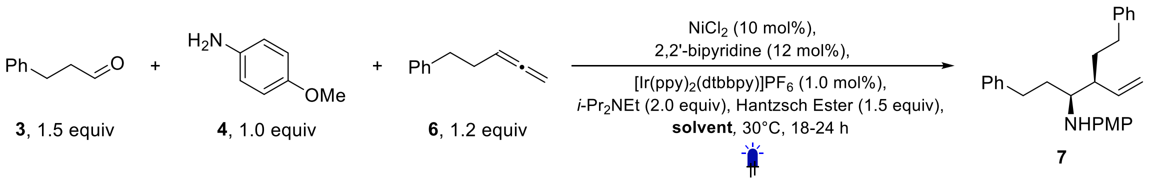

Entry	Solvent	NMR yield	dr
1	1,4-dioxane	70%	4:1
2	toluene	<10%	4:1
3	THF	75%	4:1

Reaction conditions: aldehyde (**3**, 1.5 equiv), *p*-anisidine (**4**, 200 µmol, 1.0 equiv), allene (**6**, 1.2 equiv), $NiCl_2$ (10 mol%), 2,2'-bipyridine (12 mol%), $[Ir(ppy)_2(dtbbpy)]PF_6$ (1.0 mol%), *i*-Pr_2NEt (2.0 equiv), Hantzsch ester (1.5 equiv), solvent (0.1 M), 30°C, blue LEDs, 18-24 h. Yields and diastereoselectivities were calculated from the ^{1}H-NMR spectrum of the crude product. 1,3,5-Trimethoxybenzene was used as an internal standard.

Nickel catalyst:

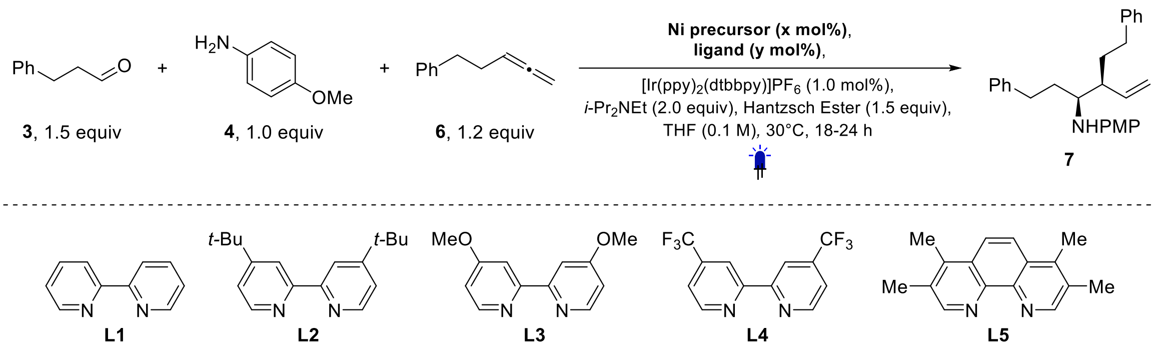

Entry	[Ni]	x	Ligand	y	NMR yield	dr
1	$NiCl_2$(glyme)	10	L1	12	35%	4:1
2	$Ni(COD)_2$	10	L1	12	57%	4:1
3	$Ni(acac)_2$	10	L1	12	30%	4:1
4	$Ni(NO_3)_2{\cdot}6H_2O$	10	L1	12	traces	–
**5**	**$NiCl_2$**	**10**	**L1**	**12**	**75%**	**4:1**
6	$NiCl_2$	10	L2	12	74%	4:1
7	$NiCl_2$	10	L3	12	<20%	4:1
8	$NiCl_2$	10	L4	12	traces	–
9	$NiCl_2$	10	L5	12	0%	–
10	$NiCl_2$	5	L1	5	45%	4:1
11	$NiCl_2$	5	L1	6	53%	4:1

Reaction conditions: aldehyde (**3**, 1.5 equiv), *p*-anisidine (**4**, 200 µmol, 1.0 equiv), allene (**6**, 1.2 equiv), [Ni] (x mol%), ligand (y mol%), $[Ir(ppy)_2(dtbbpy)]PF_6$ (1.0 mol%), *i*-Pr_2NEt (2.0 equiv), Hantzsch ester (1.5 equiv), solvent (0.1 M), 30°C, blue LEDs, 18-24 h. Yields and diastereoselectivities were calculated from the ^{1}H-NMR spectrum of the crude product. 1,3,5-Trimethoxybenzene was used as an internal standard.

Stoichiometry of reagents:

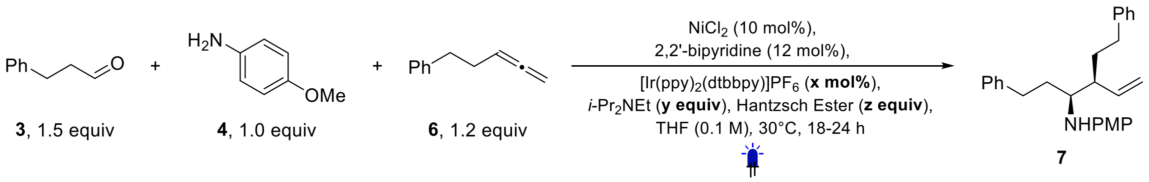

Entry	PC	*i*-Pr_2NEt	Hantzsch ester	NMR yield	dr
1	0.5 mol%	2.0 equiv	1.5 equiv	68%	4:1
2	1.0 mol%	2.0 equiv	1.5 equiv	75%	4:1
**3**[a]	**1.0 mol%**	**2.0 equiv**	**1.5 equiv**	**76% (68**[b]**)**	**4:1**
4	1.5 mol%	2.0 equiv	1.5 equiv	73%	4:1
5	1.0 mol%	0.5 equiv	1.5 equiv	69%	4:1
6	1.0 mol%	1.0 equiv	1.5 equiv	75%	4:1
7	1.0 mol%	1.5 equiv	1.5 equiv	63%	4:1
8	1.0 mol%	2.0 equiv	1.0 equiv	73%	4:1
9	1.0 mol%	2.0 equiv	2.0 equiv	72%	4:1
10	1.0 mol%	1.0 equiv	1.0 equiv	60%	4:1
11	1.0 mol%	1.1 equiv	1.1 equiv	66%	4:1

Reaction conditions: aldehyde (**3**, 1.5 equiv), *p*-anisidine (**4**, 200 µmol, 1.0 equiv), allene (**6**, 1.2 equiv), $NiCl_2$ (x mol%), ligand (y mol%), $[Ir(ppy)_2(dtbbpy)]PF_6$ (1.0 mol%), *i*-Pr_2NEt (2.0 equiv), Hantzsch ester (1.5 equiv), solvent (0.1 M), 30°C, blue LEDs, 18-24 h. [a] 1.4 equiv allene; [b] isolated; Yields and diastereoselectivities were calculated from the ^{1}H-NMR spectrum of the crude product. 1,3,5-Trimethoxybenzene was used as an internal standard.

Experiments with chromium:

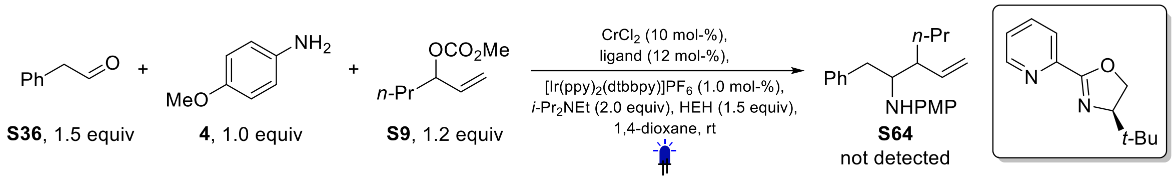

Previous reports on photochemical allylation of aldehydes used chromium as a metal catalyst. If $NiCl_2$ is replaced by $CrCl_2$ following general procedure C no product is formed.

In 2018, Glorius et al. published a protocol for the diastereoselective allylation of aldehydes via dual photoredox/chromium catalysis. Applying their conditions to our reaction did not furnish any homoallyl amine.[27]

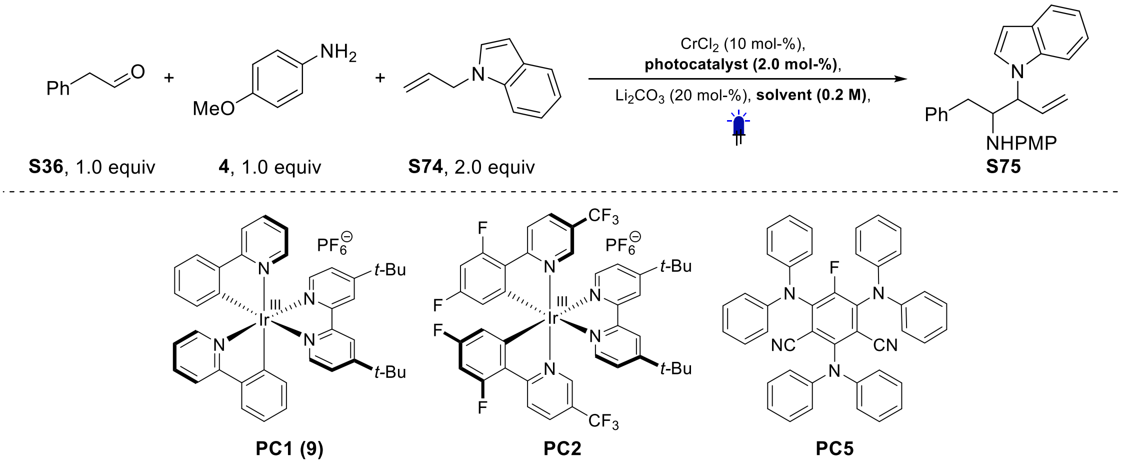

A 10 ml Schlenk tube was dried by heat gun under vacuum, backfilled with argon and cooled to room temperature using a standard Schlenk line apparatus. The Schlenk tube was charged with $CrCl_2$ (2.50 mg, 20.0 µmol, 10 mol%), photocatalyst (4.00 µmol, 2.0 mol%), *p*-anisidine (**4**, 24.6 mg, 200 µmol, 1.0 equiv) and Li_2CO_3 (3.00 mg, 40.0 µmol, 20.0 mol%) and alternately put on vacuum and argon three times. Afterwards dry, degassed solvent (1 mL), 2-phenylacetaldehyde (**S36**, 24.6 µL, 24.0 mg, 200 µmol, 1.0 equiv) and *N*-allyl indole (**S74**, 60.5 µL, 62.9 mg, 400 µmol, 2.0 equiv) were added. The tube was sealed by a screw cap and placed approximately 3 cm from blue led strips. The tube was irradiated and stirred for 24 h at room temp. Afterwards, the reaction mixture was filtered through silica and the solvent was evaporated under reduced pressure. The crude product was analyzed by ^{1}H-NMR spectroscopy.

Entry	Solvent	Photocatalyst	NMR yield	dr
1	DMF	**PC2**	0%	–
2	THF	**PC2**	0%	–
3	toluene	**PC2**	0%	–
4	CH_2Cl_2	**PC2**	0%	–
5	MeCN	**PC2**	0%	–
6	DMF	**PC1**	0%	–
7	DMF	**PC5**	0%	–

Experiments with cobalt:

In 2022, Xiao et al. reported the enantioselective addition of aryl cobalt complexes to aldehydes under photocatalytic conditions.[28] Employing their strategy for our system did not deliver the desired homoallylic amines.

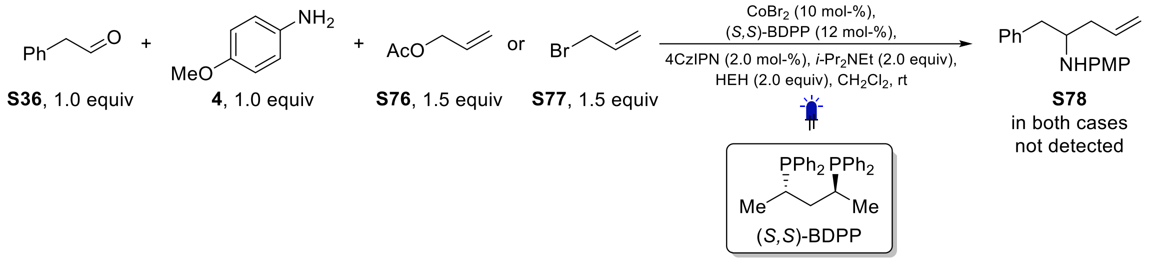

A 10 ml Schlenk tube was dried by heat gun under vacuum, backfilled with argon and cooled to room temperature using a standard Schlenk line apparatus. The Schlenk tube was charged with $CoBr_2$ (4.37 mg, 20.0 µmol, 10 mol%) and (*S,S*)-BDPP (10.9 mg, 24.0 µmol, 12 mol%) and alternately put on vacuum and argon three times. Afterwards dry, degassed CH_2Cl_2 (2 mL) was added by syringe under a flow of argon and the solution was stirred for 30 min. Then, 4-CzIPN (3.16 mg, 4.00 µmol, 2.0 mol%), *p*-anisidine (**4**, 24.6 mg, 200 µmol, 1.0 equiv), Hantzsch ester (101 mg, 400 µmol, 2.0 equiv), *i*-Pr_2NEt (68.0 µL, 51.8 mg, 200 µmol, 2.0 equiv), 2-phenylacetaldehyde (**S36**, 36.8 µL, 36.0 mg, 300 µmol, 1.5 equiv) and allyl–Br/OAc (**S76/77**, 300 µmol, 1.5 equiv) were added. The tube was sealed by a screw cap and placed approximately 3 cm from blue led strips. The tube was irradiated and stirred for 18 h at room temp. Afterwards, the reaction mixture was filtered through silica and the solvent was evaporated under reduced pressure. The crude product was analyzed by ^{1}H-NMR spectroscopy.

3.3 Allylic Carbonate Scope

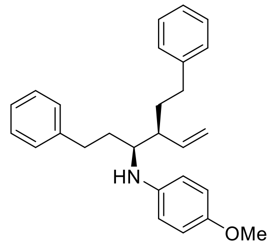

7

7 was synthesized according to general procedure C. Flash column chromatography: silica gel, *n*-pentane/Et_2O 19:1 → 9:1 v/v, R_f = 0.35 (*n*-pentane/Et_2O 9:1 v/v). Further purification was achieved by reversed-phase chromatography using the method described in general procedure C. The product (**7**, 61.4 mg, 159 µmol, 80%, dr = 3.8:1) was obtained as a yellow oil.

^{1}H-NMR (500 MHz, $CDCl_3$): δ = 1.54 (m, 1H), 1.70 (m, 2H), 1.84 (m, 0.24H), 1.93 (m, 0.82H), 2.35 (m, 1H), 2.45 (m, 1H), 2.62 (m, 2.28H), 2.86 (ddd, *J* = 13.9, 9.4, 4.7 Hz, 0.83H), 3.28 (ddd, *J* = 10.2, 3.7 Hz, 1.07H*), 3.34 (ddd, *J* = 8.5, 4.5 Hz, 0.55H*), 3.76 (s, 0.72H), 3.77 (s, 2.12H), 5.03 (ddd, *J* = 17.1, 2.1, 0.8 Hz, 0.80H), 5.18 (m, 1.01H), 5.25 (dd, *J* = 10.4, 2.0 Hz, 0.21H), 5.65 (ddd, *J* = 17.1, 10.2, 9.5 Hz, 0.79H),

5.77 (ddd, J = 17.2, 10.4, 8.8 Hz, 0.21H), 6.50 (m, 2H), 6.76 (m, 2H), 7.16 (m, 6H), 7.27 (m, 4H) ppm. *Superimposed by broad NH signal.

^{13}C-NMR (126 MHz, $CDCl_3$): δ = 32.5, 32.8, 32.9, 33.1, 33.6, 33.9, 34.9, 46.8, 47.1, 55.9, 55.9, 56.9, 57.2, 114.5, 115.1, 115.2, 117.7, 118.0, 125.8, 125.8, 125.9, 128.4, 128.4, 128.4, 128.5, 128.5, 128.6, 138.7, 138.8, 142.2, 142.2, 142.4, 142.5, 151.8, 151.9 ppm.

HRMS ($C_{27}H_{32}ON$, $[M + H]^+$, pos. ESI): m/z: calcd: 386.2478, found: 386.2471, Δ = –2.0 ppm.

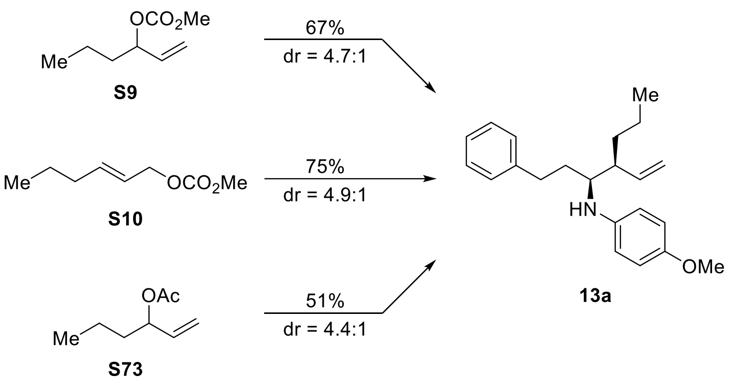

13a was synthesized according to general procedure C. Flash column chromatography: silica gel, n-pentane/Et_2O 29:1 → 19:1 v/v, R_f = 0.22 (n-pentane/Et_2O 19:1 v/v). In case of the reaction using **S9**, further purification was achieved by reversed-phase chromatography using the method described in general procedure C. The product [**13a**, 43.6 mg, 135 µmol, 67%, dr = 4.7:1 (from branched **S9**), 48.8 mg, 151 µmol, 75%, dr = 4.9:1 (from **S10**), 32.8 mg, 101 µmol, 51%, dr = 4.4:1 (from **S73**)] was obtained as a yellow oil. Analytical data for homoallylic amines synthesized from branched (**S9**) and linear allylic carbonate (**S10**) and allylic acetate (**S73**) were matching.

^{1}H-NMR (400 MHz, $CDCl_3$): δ = 0.86 (m, 3H), 1.18 (m, 1H), 1.34 (m, 3.21H), 1.52 (dddd, J = 13.9, 10.1, 9.4, 4.7 Hz, 0.87H), 1.73 (dddd, J = 13.8, 9.8, 7.9, 6.0 Hz, 0.21H), 1.93 (m, 1H), 2.31 (m, 1H), 2.68 (m, 1.24H), 2.89 (ddd, J = 14.1, 9.6, 4.7 Hz, 0.85H), 3.26 (m, 2H), 3.76 (m, 3H), 4.97 (ddd, J = 17.1, 2.2, 0.8 Hz, 0.84H), 5.11 (m, 1.01H), 5.16 (ddd, J = 10.3, 1.9, 0.6 Hz, 0.18H), 5.58 (ddd, J = 17.1, 10.2, 9.4 Hz, 0.82H), 5.71 (ddd, J = 17.2, 10.3, 8.8 Hz, 0.18H), 6.54 (m, 2H), 6.79 (m, 2H), 7.19 (m, 3H), 7.29 (m, 2H) ppm.

^{13}C-NMR (101 MHz, $CDCl_3$): δ = 14.2, 20.8, 20.8, 32.8, 32.9, 32.9, 33.0, 34.0, 34.9, 47.0, 47.3, 55.9, 55.9, 57.0, 57.2, 114.5, 115.1, 115.2, 116.9, 117.4, 125.8, 125.8, 128.4, 128.5, 128.6, 139.2, 142.3, 142.4, 142.5, 142.7, 151.7, 151.9 ppm.

HRMS ($C_{22}H_{30}ON$, $[M + H]^+$, pos. ESI): m/z: calcd: 324.2322, found: 324.2316, Δ = –1.9 ppm.

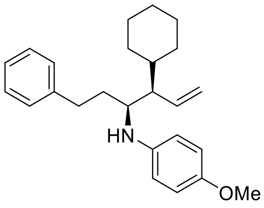

13b

13b was synthesized according to general procedure C. Flash column chromatography: silica gel, *n*-pentane/Et_2O 29:1 v/v, R_f = 0.24 (*n*-pentane/Et_2O 19:1 v/v). Further purification was achieved by reversed-phase chromatography using the method described in general procedure C. The product (**13b**, 44.2 mg, 122 µmol, 61%, dr = 3.4:1) was obtained as a yellow oil.

^{1}H-NMR (500 MHz, $CDCl_3$): δ = 0.75 (m, 0.79H), 0.86 (m, 1H), 0.98 (m, 0.25H), 1.15 (m, 3H), 1.30 (m, 0.87H), 1.44 (m, 1.17H), 1.64 (m, 5H), 1.81 (m, 0.47H), 1.89 (m, 0.75H), 1.99 (m, 1H), 2.64 (m, 1.25H), 2.92 (ddd, *J* = 13.7, 9.2, 4.5 Hz, 0.78H), 3.36 (br s, 1H), 3.49 (m, 1H), 3.75 (s, 0.68H), 3.77 (s, 2.25H), 4.90 (ddd, *J* = 17.1, 2.4, 0.6 Hz, 0.77H), 5.07 (ddd, *J* = 17.0, 2.2, 0.7 Hz, 0.23H), 5.14 (dd, *J* = 10.2, 2.4 Hz, 0.77H), 5.20 (dd, *J* = 10.2, 2.2 Hz, 0.23H), 5.54 (ddd, *J* = 17.0, 10.1 Hz, 0.77H), 5.67 (ddd, *J* = 17.1, 10.1 Hz, 0.23H), 6.48 (m, 0.45H), 6.53 (m, 1.54H), 6.74 (m, 0.46H), 6.78 (m, 1.53H), 7.15 (m, 0.51H), 7.19 (m, 2.50H), 7.29 (m, 2H) ppm.

^{13}C-NMR (126 MHz, $CDCl_3$): δ = 26.4, 26.5, 26.6, 26.6, 26.7, 30.6, 31.0, 31.8, 32.0, 32.3, 32.4, 32.9, 35.4, 37.9, 38.0, 52.5, 53.1, 53.4, 53.6, 55.9, 55.9, 114.5, 115.0, 115.1, 115.3, 118.1, 118.2, 125.8, 128.4, 128.5, 128.7, 137.5, 138.1, 142.3, 142.3, 142.6, 151.7, 151.9 ppm.

HRMS ($C_{25}H_{34}ON$, $[M + H]^+$, pos. ESI): *m/z*: calcd: 364.2635, found: 364.2636, Δ = 0.3 ppm.

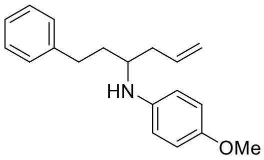

13c

13c was synthesized according to general procedure C. Flash column chromatography: silica gel, *n*-pentane/Et_2O 29:1 → 19:1 v/v, R_f = 0.18 (*n*-pentane/Et_2O 9:1 v/v). The product (**13c**, 49.2 mg, 175 µmol, 87%) was obtained as a yellow oil.

^{1}H-NMR (500 MHz, $CDCl_3$): δ = 1.80 (m, 1H), 1.89 (m, 1H), 2.34 (m, 2H), 2.75 (m, 2H), 3.28 (br s, 1H), 3.40 (dddd, *J* = 7.2, 5.6 Hz, 1H), 3.77 (s, 3H), 5.10 (m, 2H), 5.83 (dddd, *J* = 16.8, 10.4, 7.2 Hz, 1H), 6.54 (m, 2H), 6.79 (m, 2H), 7.20 (m, 3H), 7.30 (m, 2H) ppm.

^{13}C-NMR (126 MHz, $CDCl_3$): δ = 32.5, 36.2, 38.6, 52.9, 55.9, 114.9, 115.0, 117.8, 125.9, 128.4, 128.5, 134.8, 141.9, 142.1, 152.0 ppm.

HRMS ($C_{19}H_{24}ON$, $[M + H]^+$, pos. ESI): *m/z*: calcd: 282.1858, found: 282.1861, Δ = 3.2 ppm.

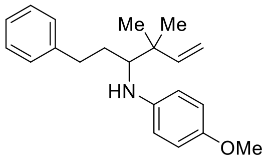

13d

13d was synthesized according to general procedure C. Flash column chromatography: silica gel, *n*-pentane/Et_2O 29:1 → 19:1 v/v, R_f = 0.37 (*n*-pentane/Et_2O 9:1 v/v). The product (**13d**, 43.6 mg, 141 µmol, 70%) was obtained as a yellow oil.

^{1}H-NMR (400 MHz, $CDCl_3$): δ = 1.03 (s, 3H), 1.06 (s, 3H), 1.49 (dddd, *J* = 13.8, 10.8, 9.8, 4.8 Hz, 1H), 2.08 (dddd, *J* = 13.4, 9.6, 7.0, 2.3 Hz, 1H), 2.55 (ddd, *J* = 13.8, 9.7, 7.1 Hz, 1H), 2.82 (ddd, *J* = 14.2, 9.9, 4.7 Hz, 1H), 3.10 (dd, *J* = 10.8, 2.4 Hz, 1H), 3.22 (s, 1H), 3.76 (s, 3H), 5.04 (m, 2H), 5.88 (dd, *J* = 17.4, 10.9 Hz, 1H), 6.55 (m, 2H), 6.76 (m, 2H), 7.11 (m, 2H), 7.18 (m, 1H), 7.26 (m, 2H) ppm.

^{13}C-NMR (101 MHz, $CDCl_3$): δ = 23.0, 25.9, 33.6, 34.8, 42.7, 56.0, 62.1, 112.8, 113.7, 115.0, 125.8, 128.3, 128.5, 142.4, 144.3, 145.6, 151.3 ppm.

HRMS ($C_{21}H_{28}ON$, $[M + H]^+$, pos. ESI): *m/z*: calcd: 310.2165, found: 310.2161, Δ = –1.3 ppm.

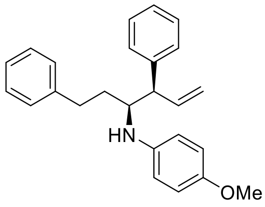

13e

13e was synthesized according to general procedure C. Flash column chromatography: silica gel, *n*-pentane/Et_2O 29:1 → 19:1 v/v, R_f = 0.30 (*n*-pentane/Et_2O 9:1 v/v). Further purification was achieved by reversed-phase chromatography using the method described in general procedure C. The product (**13e**, 24.3 mg, 68.0 µmol, 34%, dr >19:1) was obtained as a yellow oil.

^{1}H-NMR (400 MHz, $CDCl_3$): δ = 1.58 (m, 1H), 1.93 (dddd, *J* = 13.6, 9.9, 7.0, 3.1 Hz, 1H), 2.61 (ddd, *J* = 13.8, 9.6, 7.0 Hz, 1H), 2.83 (ddd, *J* = 14.3, 9.8, 4.9 Hz, 1H), 3.36 (br s, 1H), 3.63 (m, 2H), 3.77 (s, 3H), 5.11

(ddd, *J* = 16.9, 2.0, 0.7 Hz, 1H), 5.22 (dd, *J* = 10.2, 2.0 Hz, 1H), 6.17 (ddd, *J* = 17.0, 10.2, 9.2 Hz, 1H), 6.58 (m, 2H), 6.79 (m, 2H), 7.07 (m, 2H), 7.24 (m, 8H) ppm.

^{13}C-NMR (101 MHz, $CDCl_3$): δ = 32.7, 33.3, 53.2, 55.9, 58.2, 114.3, 115.1, 115.4, 117.9, 125.8, 126.5, 128.1, 128.4, 128.5, 137.1, 141.5, 142.1, 142.1, 152.1 ppm.

HRMS ($C_{25}H_{28}ON$, $[M + H]^+$, pos. ESI): *m/z*: calcd: 358.2165, found: 358.2166, Δ = 0.0 ppm.

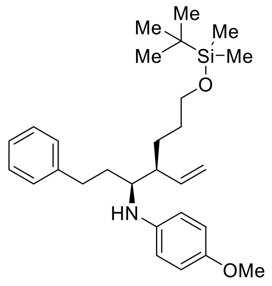

13f

13f was synthesized according to general procedure C. Flash column chromatography: silica gel, *n*-pentane/Et_2O 19:1 → 9:1 v/v, R_f = 0.34 (*n*-pentane/Et_2O 9:1 v/v). The product (**13f**, 74.9 mg, 165 µmol, 83%, dr = 5.0:1) was obtained as a yellow oil.

^{1}H-NMR (500 MHz, $CDCl_3$): δ = 0.03 (m, 6H), 0.89 (s, 2.04H), 0.90 (s, 6.89H), 1.45 (m, 4.79H), 1.73 (m, 0.26H), 1.85 (m, 0.20H), 1.94 (m, 0.84H), 2.28 (m, 1H), 2.65 (ddd, *J* = 13.7, 9.3, 7.4 Hz, 1H), 2.74 (m, 0.22H), 2.88 (ddd, *J* = 14.0, 9.6, 4.7 Hz, 0.85H), 3.26 (m, 1H), 3.33 (br s, 1H), 3.56 (m, 2H), 3.76 (m, 3H), 4.98 (ddd, *J* = 17.1, 2.2, 0.7 Hz, 0.83H), 5.12 (m, 1H), 5.17 (dd, *J* = 10.3, 1.9 Hz, 0.17H), 5.58 (ddd, *J* = 17.1, 10.2, 9.4 Hz, 0.83H), 5.70 (ddd, *J* = 17.1, 10.3, 8.7 Hz, 0.17H), 6.53 (m, 2H), 6.78 (m, 2H), 7.18 (m, 3H), 7.28 (m, 2H) ppm.

^{13}C-NMR (126 MHz, $CDCl_3$): δ = –5.2, –5.2, 18.4, 26.1, 26.8, 27.9, 30.9, 31.0, 32.8, 32.9, 35.0, 47.2, 47.3, 55.9, 55.9, 57.0, 57.3, 63.1, 63.3, 114.5, 115.1, 115.2, 117.2, 117.7, 125.8, 128.4, 128.5, 128.6, 139.0, 142.3, 142.3, 142.4, 142.6, 151.7, 151.9 ppm.

HRMS ($C_{28}H_{44}O_2NSi$, $[M + H]^+$, pos. ESI): *m/z*: calcd: 454.3136, found: 454.3135, Δ = –0.2 ppm.

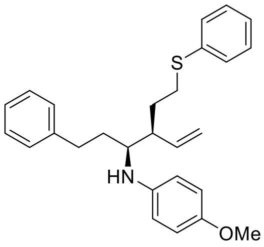

13g

13g was synthesized according to general procedure C. Flash column chromatography: silica gel, *n*-pentane/Et_2O 19:1 → 9:1 v/v, R_f = 0.25 (*n*-pentane/Et_2O 9:1 v/v). Further purification was achieved by reversed-phase chromatography using the method described in general procedure C. The product (**13g**, 43.3 mg, 104 µmol, 52%, dr = 4.8:1) was obtained as a yellow oil.

^{1}H-NMR (500 MHz, $CDCl_3$): δ = 1.53 (m, 1H), 1.65 (m, 1H), 1.81 (m, 2H), 2.48 (dddd, *J* = 9.4, 4.2 Hz, 0.83H), 2.63 (m, 1.18H), 2.75 (m, 1.27H), 2.85 (ddd, *J* = 14.0, 9.6, 4.7 Hz, 0.85H), 2.94 (m, 1H), 3.26 (m, 2H), 3.76 (s, 0.58H), 3.77 (s, 2.32H), 5.03 (ddd, *J* = 17.1, 2.1, 0.7 Hz, 0.82H), 5.17 (m, 1.00H), 5.22 (dd, *J* = 10.3, 1.8 Hz, 0.17H), 5.57 (ddd, *J* = 17.0, 10.2, 9.5 Hz, 0.83H), 5.69 (ddd, *J* = 17.2, 10.3, 8.9 Hz, 0.17H), 6.51 (m, 2H), 6.78 (m, 2H), 7.14 (m, 2H), 7.18 (m, 2H), 7.27 (m, 6H) ppm.

^{13}C-NMR (126 MHz, $CDCl_3$): δ = 30.0, 31.0, 31.8, 32.7, 33.3, 34.7, 46.1, 46.9, 55.9, 55.9, 56.9, 114.5, 115.1, 115.1, 118.2, 118.6, 125.8, 125.9, 125.9, 128.4, 128.5, 128.6, 128.9, 129.1, 129.2, 136.5, 136.6, 137.8, 137.9, 142.0, 142.1, 142.2, 151.9, 152.0 ppm.

HRMS ($C_{27}H_{32}ONS$, $[M + H]^+$, pos. ESI): *m/z*: calcd: 418.2199, found: 418.2203, Δ = 0.9 ppm.

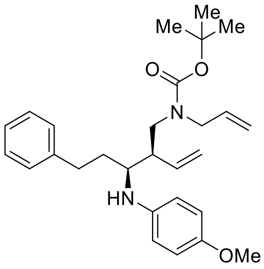

13h

13h was synthesized according to general procedure C. Further purification was achieved by reversed-phase chromatography using the method described in general procedure C. Flash column chromatography: silica gel, *n*-pentane/Et_2O 7:1 → 3:1 v/v, R_f = 0.30 (*n*-pentane/Et_2O 4:1 v/v). The product (**13h**, 49.4 mg, 110 µmol, 55%, dr = 8.4:1) was obtained as a yellow oil.

^{1}H-NMR (500 MHz, C_6D_6, 353 K): δ = 1.42 (s, 8.08H), 1.43 (s, 1.08H), 1.50 (m, 1H), 1.96 (m, 1H), 2.65 (ddd, *J* = 13.8, 9.4, 6.9 Hz, 1H), 2.72 (m, 1H), 2.81 (ddd, *J* = 14.3, 9.6, 5.0 Hz, 1H), 3.16 (dd, *J* = 14.1, 8.0

Hz, 1H), 3.34 (m, 1H), 3.45 (s, 4H), 3.66 (m, 1H), 3.76 (m, 1H), 4.96 (m, 4H), 5.63 (m, 2H), 6.44 (m, 0.21H), 6.52 (m, 1.79H), 6.75 (m, 0.21H), 6.78 (m, 1.79H), 7.05 (m, 1H), 7.13 (m, 4H) ppm.

^{13}C-NMR (126 MHz, C_6D_6, 353 K): δ = 28.6, 28.6, 32.2, 33.2, 33.2, 34.1, 35.1, 46.8, 47.8, 48.6, 48.7, 48.8, 49.1, 50.5, 50.8, 55.7, 55.7, 55.8, 79.2, 79.4, 115.4, 115.7, 115.8, 115.8, 116.0, 117.7, 118.0, 126.1, 126.1, 128.3, 128.6, 128.8, 128.9, 134.9, 135.0, 138.0, 142.7, 153.2, 155.6 ppm.

HRMS ($C_{28}H_{39}O_3N_2$, $[M + H]^+$, pos. ESI): *m/z*: calcd: 451.2955, found: 451.2952, Δ = –0.8 ppm.

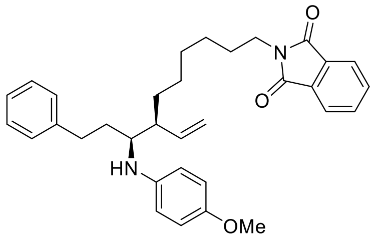

13i

13i was synthesized according to general procedure C. Flash column chromatography: silica gel, *n*-pentane/Et_2O 7:1 → 2:1 v/v, R_f = 0.18 (*n*-pentane/Et_2O 3:1 v/v). Further purification was achieved by reversed-phase chromatography using the method described in general procedure C. The product (**13i**, 62.1 mg, 122 µmol, 61%, dr = 3.9:1) was obtained as a yellow oil.

^{1}H-NMR (700 MHz, $CDCl_3$): δ = 1.12 (m, 0.80H), 1.30 (m, 7.23H), 1.43 (m, 0.28H), 1.49 (m, 0.83H), 1.63 (m, 2H), 1.71 (m, 0.24H), 1.84 (m, 0.26H), 1.90 (m, 0.82H), 2.27 (m, 1H), 2.64 (m, 1H), 2.72 (m, 0.23H), 2.87 (ddd, *J* = 14.0, 9.6, 4.7 Hz, 0.80H), 3.24 (ddd, *J* = 10.2, 4.4, 3.0 Hz, 0.83H), 3.32 (m, 1.16H), 3.66 (m, 2H), 3.74 (s, 0.66H), 3.75 (s, 2.35H), 4.95 (ddd, *J* = 17.1, 2.1, 0.7 Hz, 0.79H), 5.09 (m, 1H), 5.14 (dd, *J* = 10.4, 2.0 Hz, 0.21H), 5.55 (ddd, *J* = 17.1, 10.2, 9.5 Hz, 0.79H), 5.68 (ddd, *J* = 17.2, 10.3, 8.8 Hz, 0.20H), 6.52 (m, 2H), 6.76 (m, 2H), 7.17 (m, 3H), 7.26 (m, 2H), 7.70 (m, 2H), 7.84 (m, 2H) ppm.

^{13}C-NMR (176 MHz, $CDCl_3$): δ = 26.8, 27.5, 28.6, 28.6, 29.2, 29.3, 30.4, 31.6, 32.8, 32.9, 32.9, 34.9, 38.1, 47.2, 47.5, 55.9, 55.9, 56.9, 57.2, 114.4, 115.0, 115.1, 115.2, 117.0, 117.5, 123.2, 125.8, 125.8, 128.4, 128.5, 128.6, 132.2, 133.9, 133.9, 139.1, 139.1, 142.3, 142.3, 142.5, 142.6, 151.7, 151.8, 168.5 ppm.

HRMS ($C_{33}H_{39}O_3N_2$, $[M + H]^+$, pos. ESI): *m/z*: calcd: 511.2955, found: 511.2961, Δ = 1.2 ppm.

3.4 Aniline Scope

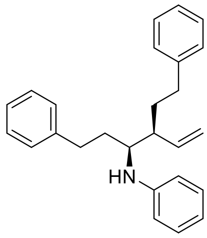

13j

13j was synthesized according to general procedure C. Flash column chromatography: silica gel, *n*-pentane/Et_2O 49:1 → 29:1 v/v, R_f = 0.41 (*n*-pentane/Et_2O 19:1 v/v). Further purification was achieved by reversed-phase chromatography using the method described in general procedure C. The product (**13j**, 47.1 mg, 132 µmol, 66%, dr = 4.4:1) was obtained as a yellow oil.

^{1}H-NMR (400 MHz, $CDCl_3$): δ = 1.58 (m, 1H), 1.74 (m, 2H), 1.88 (m, 0.21H), 1.98 (dddd, *J* = 13.9, 10.0, 7.4, 2.9 Hz, 0.85H), 2.38 (m, 1H), 2.49 (m, 1H), 2.67 (m, 2.27H), 2.86 (ddd, *J* = 14.0, 9.5, 4.8 Hz, 0.83H), 3.41 (ddd, *J* = 10.0, 3.8 Hz, 0.82H), 3.48 (m, 0.25H), 3.57 (br s, 1H), 5.06 (dd, *J* = 17.1, 2.1 Hz, 0.82H), 5.20 (m, 1.01H), 5.28 (dd, *J* = 10.4, 1.9 Hz, 0.19H), 5.68 (ddd, *J* = 17.1, 9.8 Hz, 0.81H), 5.80 (ddd, *J* = 17.2, 10.4, 8.7 Hz, 0.19H), 6.55 (m, 2H), 6.70 (m, 1H), 7.18 (m, 8H), 7.29 (m, 4H) ppm.

^{13}C-NMR (101 MHz, $CDCl_3$): δ = 32.5, 32.8, 32.8, 33.2, 33.4, 33.8, 33.8, 35.0, 46.9, 47.4, 55.7, 56.0, 113.1, 113.7, 116.9, 117.1, 117.8, 118.0, 125.8, 125.8, 125.9, 128.4, 128.4, 128.4, 128.5, 128.5, 128.6, 129.4, 138.5, 138.8, 142.1, 142.2, 142.3, 142.4, 148.1, 148.3 ppm.

HRMS ($C_{26}H_{30}N$, $[M + H]^+$, pos. APCI): *m/z*: calcd: 356.2373, found: 356.2373, Δ = 0.2 ppm.

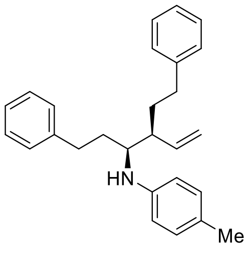

13k

13k was synthesized according to general procedure C. Flash column chromatography: silica gel, *n*-pentane/Et_2O 49:1 → 29:1 v/v, R_f = 0.42 (*n*-pentane/Et_2O 19:1 v/v). The product (**13k**, 64.2 mg, 174 µmol, 87%, dr = 4.0:1) was obtained as a yellow oil.

^{1}H-NMR (500 MHz, $CDCl_3$): δ = 1.58 (m, 0.88H), 1.69 (m, 1H), 1.78 (m, 1.26H), 1.89 (m, 0.23H), 1.98 (m, 0.23H), 2.29 (s, 0.72H), 2.30 (s, 2.19H), 2.39 (m, 1H), 2.50 (m, 1H), 2.67 (m, 2.32H), 2.88 (ddd, *J* = 14.0, 9.5, 4.7 Hz, 0.83H), 3.39 (ddd, *J* = 10.2, 4.4, 3.0 Hz, 0.89H), 3.46 (br s, 1H), 5.07 (ddd, *J* = 17.1, 2.1, 0.7 Hz,

0.81H), 5.22 (m, 1.01H), 5.29 (dd, *J* = 10.4, 1.9 Hz, 0.20H), 5.69 (ddd, *J* = 17.1, 10.2, 9.4 Hz, 0.80H), 5.81 (ddd, *J* = 17.2, 10.3, 8.8 Hz, 0.20H), 6.51 (m, 2H), 7.01 (m, 2H), 7.20 (m, 6H), 7.31 (m, 4H) ppm.

^{13}C-NMR (126 MHz, $CDCl_3$): δ = 20.4, 20.5, 32.5, 32.8, 32.8, 33.2, 33.5, 33.9, 35.0, 46.9, 47.3, 56.0, 56.4, 113.2, 113.8, 117.7, 118.0, 125.8, 125.8, 125.8, 126.0, 126.2, 128.3, 128.4, 128.4, 128.4, 128.5, 128.6, 129.9, 129.9, 138.6, 138.8, 142.1, 142.3, 142.4, 142.5, 145.8, 146.0 ppm.

HRMS ($C_{27}H_{32}N$, $[M + H]^+$, pos. ESI): *m/z*: calcd: 370.2529, found: 370.2538, Δ = 2.4 ppm.

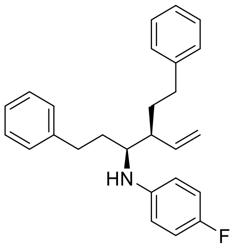

13l

13l was synthesized according to general procedure C. Flash column chromatography: silica gel, *n*-pentane/Et_2O 49:1 → 29:1 v/v, R_f = 0.33 (*n*-pentane/Et_2O 19:1 v/v). Further purification was achieved by reversed-phase chromatography using the method described in general procedure C. The product (**13l**, 47.4 mg, 127 µmol, 63%, dr = 4.4:1) was obtained as a yellow oil.

^{1}H-NMR (500 MHz, $CDCl_3$): δ = 1.56 (dddd, *J* = 14.2, 9.5, 4.8 Hz, 0.88H), 1.72 (m, 2.19H), 1.88 (m, 0.20H), 1.96 (m, 0.82H), 2.35 (m, 1H), 2.47 (m, 1H), 2.64 (m, 2.25H), 2.85 (ddd, *J* = 13.8, 9.3, 4.8 Hz, 0.82H), 3.29 (ddd, *J* = 10.2, 4.4, 3.1 Hz, 0.82H), 3.35 (ddd, *J* = 8.5, 4.5 Hz, 0.22H), 3.44 (br s, 1H), 5.03 (dd, *J* = 17.1, 2.1 Hz, 0.81H), 5.20 (m, 1.00H), 5.27 (dd, *J* = 10.4, 1.8 Hz, 0.19H), 5.66 (ddd, *J* = 17.1, 9.8 Hz, 0.81H), 5.77 (ddd, *J* = 17.1, 10.4, 8.8 Hz, 0.18H), 6.45 (m, 2H), 6.87 (m, 2H), 7.15 (m, 4H), 7.21 (m, 2H), 7.30 (m, 4H) ppm.

^{13}C-NMR (126 MHz, $CDCl_3$): δ = 32.5, 32.7, 32.8, 33.0, 33.4, 33.8, 33.8, 34.8, 46.7, 47.0, 56.5, 56.9, 113.9 (d, *J* = 7.3 Hz), 114.5 (d, *J* = 7.4 Hz), 115.7 (d, *J* = 22.3 Hz), 115.8 (d, *J* = 22.2 Hz), 117.9, 118.2, 125.8, 125.9, 125.9, 128.4 , 128.4, 128.4, 128.5, 128.6, 138.5, 138.6, 142.0, 142.1, 142.2, 142.3, 144.3 (d, *J* = 1.8 Hz), 144.6 (d, *J* = 1.8 Hz), 155.5 (d, *J* = 234.5 Hz), 155.6 (d, *J* = 234.6 Hz) ppm.

^{19}F-NMR (471 MHz, $CDCl_3$): δ = –128.68 (m), –128.42 (m, *dia*-**13l**) ppm.

HRMS ($C_{26}H_{29}FN$, $[M + H]^+$, pos. ESI): *m/z*: calcd: 374.2279, found: 374.2285, Δ = 1.8 ppm.

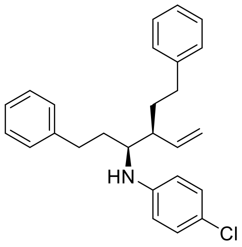

13m

13m was synthesized according to general procedure C. Flash column chromatography: silica gel, *n*-pentane/Et_2O 49:1 → :1 v/v, R_f = 0.40 (*n*-pentane/Et_2O 19:1 v/v). Further purification was achieved by reversed-phase chromatography using the method described in general procedure C. The product (**13m**, 39.0 mg, 100 µmol, 50%, dr = 4.9:1) was obtained as a yellow oil.

^{1}H-NMR (500 MHz, $CDCl_3$): δ = 1.56 (dddd, *J* = 13.9, 10.2, 9.0, 4.8 Hz, 1H), 1.70 (m, 2H), 1.88 (m, 0.18H), 1.96 (dddd, *J* = 14.0, 9.2, 7.7, 3.0 Hz, 0.82H), 2.33 (m, 1H), 2.46 (m, 1H), 2.62 (m, 2.23H), 2.81 (ddd, *J* = 13.9, 9.2, 4.8 Hz, 0.83H), 3.30 (ddd, *J* = 10.2, 3.8 Hz, 0.82H), 3.37 (ddd, *J* = 8.5, 4.5 Hz, 0.18H), 3.56 (br s, 1H), 5.02 (ddd, *J* = 17.1, 2.0, 0.7 Hz, 0.81H), 5.18 (m, 0.98H), 5.27 (ddd, *J* = 10.4, 1.9, 0.6 Hz, 0.18H), 5.64 (ddd, *J* = 17.1, 10.2, 9.4 Hz, 0.81H), 5.75 (ddd, *J* = 17.2, 10.3, 8.8 Hz, 0.17H), 6.41 (m, 1.62H), 6.45 (m, 0.41H), 7.08 (m, 2H), 7.13 (m, 4H), 7.21 (m, 2H), 7.28 (m, 4H) ppm.

^{13}C-NMR (126 MHz, $CDCl_3$): δ = 32.5, 32.6, 32.7, 33.0, 33.3, 33.7, 33.8, 34.8, 46.7, 47.1, 55.8, 56.2, 114.1, 114.6, 118.1, 118.2, 121.3, 121.4, 125.9, 125.9, 126.0, 128.4, 128.5, 128.6, 129.2, 129.2, 138.2, 138.5, 141.8, 142.0, 142.1, 142.3, 146.6, 146.8 ppm.

HRMS ($C_{26}H_{29}ClN$, $[M + H]^+$, pos. APCI): *m/z*: calcd: 390.1983, found: 390.1990, Δ = 1.8 ppm.

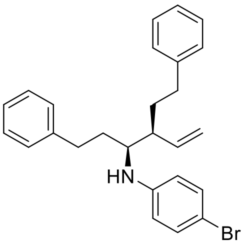

13n

13n was synthesized according to general procedure C. Flash column chromatography: silica gel, *n*-pentane/Et_2O 49:1 → 29:1 v/v, R_f = 0.27 (*n*-pentane/Et_2O 19:1 v/v). Further purification was achieved by reversed-phase chromatography using the method described in general procedure C. The product (**13n**, 35.9 mg, 82.6 µmol, 41%, dr = 4.9:1) was obtained as a yellow oil.

^{1}H-NMR (500 MHz, $CDCl_3$): δ = 1.55 (m, 1H), 1.70 (m, 2H), 1.88 (dddd, *J* = 14.1, 9.3, 6.6, 4.9 Hz, 0.19H), 1.95 (dddd, *J* = 13.9, 9.2, 7.7, 3.0 Hz, 0.83H), 2.31 (m, 1H), 2.46 (m, 1H), 2.61 (m, 2.26H), 2.81 (ddd, *J* =

13.8, 9.2, 4.8 Hz, 0.84H), 3.30 (m, 0.82H), 3.36 (m, 0.19H), 3.57 (br s, 1H), 5.02 (ddd, *J* = 17.1, 2.0, 0.7 Hz, 0.83H), 5.18 (m, 1.00H), 5.26 (dd, *J* = 10.3, 1.9 Hz, 0.17H), 5.63 (ddd, *J* = 17.1, 10.2, 9.4 Hz, 0.83H), 5.74 (ddd, *J* = 17.2, 10.4, 8.7 Hz, 0.17H), 6.36 (m, 1.62H), 6.39 (m, 0.36H), 7.12 (m, 4H), 7.21 (m, 4H), 7.28 (m, 4H) ppm.

^{13}C-NMR (126 MHz, $CDCl_3$): δ = 32.5, 32.6, 32.7, 33.0, 33.2, 33.7, 33.8, 34.8, 46.7, 47.1, 55.7, 56.1, 108.2, 108.4, 114.6, 115.1, 118.1, 118.3, 125.9, 125.9, 126.0, 128.4, 128.5, 128.5, 128.6, 132.0, 132.1, 138.2, 138.5, 141.8, 141.9, 142.1, 142.2, 147.0, 147.2 ppm.

HRMS ($C_{26}H_{29}BrN$, $[M + H]^+$, pos. APCI): *m/z*: calcd: 434.1478, found: 434.1487, Δ = 2.1 ppm.

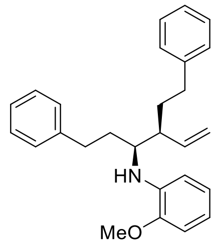

13o

13o was synthesized according to general procedure C. Flash column chromatography: silica gel, *n*-pentane/Et_2O 49:1 → 29:1 v/v, R_f = 0.38 (*n*-pentane/Et_2O 19:1 v/v). Further purification was achieved by reversed-phase chromatography using the method described in general procedure C. The product (**13o**, 18.0 mg, 46.6 µmol, 23%, dr = 2.2:1) was obtained as a yellow oil.

^{1}H-NMR (500 MHz, $CDCl_3$): δ = 1.70 (m, 3H), 1.88 (m, 0.34H), 1.96 (dddd, *J* = 13.2, 10.1, 7.2, 3.1 Hz, 0.68H), 2.32 (dddd, *J* = 9.3, 4.4 Hz, 0.73H), 2.45 (m, 1.33H), 2.64 (m, 2.38H), 2.82 (ddd, *J* = 14.2, 9.7, 4.8 Hz, 0.69H), 3.40 (ddd, *J* = 8.6, 3.7 Hz, 0.68H), 3.45 (ddd, *J* = 8.4, 4.3 Hz, 0.32H), 3.86 (s, 0.95H), 3.87 (s, 1.92H), 4.23 (br s, 1H), 5.00 (dd, *J* = 17.1, 2.0 Hz, 0.68H), 5.17 (m, 0.98H), 5.24 (dd, *J* = 10.4, 1.9 Hz, 0.31H), 5.68 (ddd, *J* = 17.1, 10.2, 9.2 Hz, 0.68H), 5.80 (ddd, *J* = 17.1, 10.3, 8.9 Hz, 0.32H), 6.49 (dd, *J* = 8.0, 1.4 Hz, 0.67H), 6.55 (dd, *J* = 8.0, 1.4 Hz, 0.31H), 6.64 (m, 1H), 6.80 (m, 2H), 7.15 (m, 6H), 7.27 (m, 4H) ppm.

^{13}C-NMR (126 MHz, $CDCl_3$): δ = 32.4, 32.7, 32.8, 33.2, 33.7, 33.8, 33.9, 35.0, 47.0, 47.5, 55.6, 55.6, 55.7, 56.0, 109.8, 109.9, 110.1, 110.5, 115.7, 115.9, 117.6, 121.4, 121.4, 125.7, 125.8, 128.3, 128.4, 128.4, 128.5, 128.5, 128.5, 128.6, 138.1, 138.3, 138.7, 139.0, 142.2, 142.4, 142.4, 142.6, 146.7, 146.9 ppm.

HRMS ($C_{27}H_{32}NO$, $[M + H]^+$, pos. ESI): *m/z*: calcd: 386.2478, found: 386.2483, Δ = 1.2 ppm.

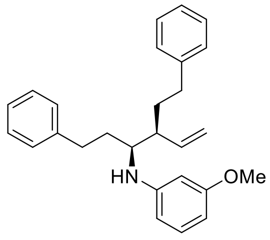

13p

13p was synthesized according to general procedure C. Flash column chromatography: silica gel, *n*-pentane/Et_2O 29:1 → 19:1 v/v, R_f = 0.20 (*n*-pentane/Et_2O 19:1 v/v). Further purification was achieved by reversed-phase chromatography using the method described in general procedure C. The product (**13p**, 52.3 mg, 136 µmol, 68%, dr = 4.6:1) was obtained as a yellow oil.

^{1}H-NMR (500 MHz, $CDCl_3$): δ = 1.57 (dddd, *J* = 14.2, 9.7, 4.8 Hz, 1H), 1.66 (m, 0.83H), 1.77 (m, 1.32H), 1.88 (m, 0.21H), 1.97 (dddd, *J* = 13.9, 9.9, 7.3, 2.9 Hz, 0.83H), 2.37 (dddd, *J* = 9.3, 4.3 Hz, 1H), 2.47 (m, 1H), 2.64 (m, 2.27H), 2.84 (ddd, *J* = 14.0, 9.5, 4.7 Hz, 0.83H), 3.39 (ddd, *J* = 10.0, 3.7 Hz, 0.81H), 3.45 (ddd, *J* = 8.5, 4.4 Hz, 0.20H), 3.60 (br s, 1H), 3.78 (s, 0.58H), 3.78 (s, 2.33H), 5.07 (dd, *J* = 17.1, 2.0 Hz, 0.82H), 5.20 (m, 1.01H), 5.27 (dd, *J* = 10.4, 1.9 Hz, 0.19H), 5.67 (ddd, *J* = 17.1, 9.8 Hz, 0.82H), 5.78 (ddd, *J* = 17.2, 10.4, 8.7 Hz, 0.18H), 6.14 (dd, *J* = 2.3 Hz, 1H), 6.18 (m, 1H), 6.27 (m, 1H), 7.08 (m, 1H), 7.18 (m, 6H), 7.29 (m, 4H) ppm.

^{13}C-NMR (126 MHz, $CDCl_3$): δ = 32.5, 32.7, 32.8, 33.3, 33.5, 33.8, 33.8, 35.0, 46.8, 47.6, 55.1, 55.1, 55.8, 56.0, 99.0, 99.6, 102.1, 102.2, 106.2, 106.8, 117.9, 118.1, 125.8, 125.8, 125.9, 128.4, 128.4, 128.4, 128.4, 128.5, 128.6, 130.1, 130.1, 138.4, 138.8, 142.0, 142.2, 142.3, 142.4, 149.5, 149.7, 161.0 ppm.

HRMS ($C_{27}H_{32}NO$, $[M + H]^+$, pos. ESI): *m/z*: calcd: 386.2478, found: 386.2482, Δ = 0.9 ppm.

3.5 Allene Scope

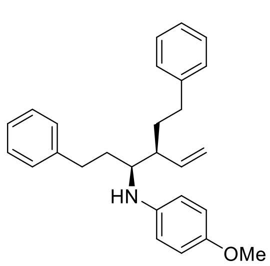

7

7 was synthesized according to general procedure C. Flash column chromatography: silica gel, *n*-pentane/Et_2O 29:1 → 9:1 v/v, R_f = 0.19 (*n*-pentane/Et_2O 19:1 v/v). The product (**7**, 52.6 mg, 136 µmol, 68%, dr = 4.1:1) was obtained as a yellow oil. Analytical data were in accordance with those measured for the allylic carbonate reaction.

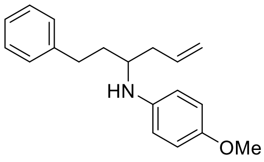

13c

13c was synthesized according to general procedure C using propa-1,2-diene (1.9 M in THF, 211 µL, 400 µmol, 2.0 equiv). Flash column chromatography: silica gel, *n*-pentane/Et_2O 29:1 → 19:1 v/v, R_f = 0.27 (*n*-pentane/Et_2O 9:1 v/v). The product (**13c**, 25.5 mg, 90.6 µmol, 45%) was obtained as a yellow oil. Analytical data were in accordance with those measured for the allylic carbonate reaction.

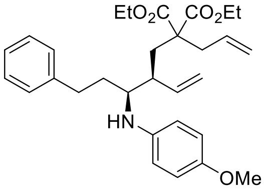

13q

13q was synthesized according to general procedure C. Flash column chromatography: silica gel, *n*-pentane/Et_2O 7:1 → 4:1 v/v, R_f = 0.35 (*n*-pentane/Et_2O 3:1 v/v). Further purification was achieved by reversed-phase chromatography using the method described in general procedure C. The product (**13q,** 51.5 mg, 104 µmol, 52%, dr = 16:1) was obtained as a yellow oil.

^{1}H-NMR (400 MHz, $CDCl_3$): δ = 1.21 (m, 7H), 1.46 (m, 1H), 1.98 (m, 3H), 2.38 (dddd, *J* = 9.9, 4.0, 2.3 Hz, 1H), 2.62 (m, 3H), 2.90 (ddd, *J* = 14.2, 10.1, 4.5 Hz, 1H), 3.21 (ddd, *J* = 10.6, 4.0, 2.7 Hz, 1H), 3.45 (br s, 1H), 3.75 (s, 0.18H), 3.76 (s, 2.81H), 4.09 (m, 4H), 4.90 (ddd, *J* = 17.1, 2.0, 0.7 Hz, 1H), 5.05 (m, 3H), 5.53 (m, 2H), 6.51 (m, 2H), 6.76 (m, 2H), 7.19 (m, 3H), 7.28 (m, 2H) ppm.

^{13}C-NMR (101 MHz, $CDCl_3$): δ = 14.0, 14.0, 32.6, 33.0, 34.1, 36.8, 42.2, 55.9, 57.0, 58.8, 61.0, 61.3, 115.1, 115.5, 118.4, 119.2, 125.8, 128.4, 128.6, 132.5, 138.1, 141.8, 142.3, 152.0, 170.8, 171.2 ppm.

HRMS ($C_{30}H_{40}O_5N$, $[M + H]^+$, pos. ESI): *m/z*: calcd: 494.2901, found: 494.2911, Δ = 2.1 ppm.

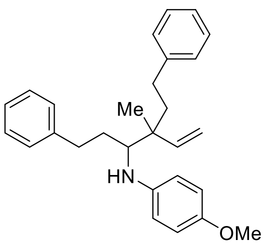

13r

13r was synthesized according to general procedure C. Flash column chromatography: silica gel, *n*-pentane/Et_2O 29:1 → 19:1 v/v, R_f = 0.29 (*n*-pentane/Et_2O 1:1 v/v). Further purification was achieved by reversed-phase chromatography using the method described in general procedure C. The product (**13r**, 54.3 mg, 136 µmol, 68%, dr = 1.1:1) was obtained as a yellow oil.

^{1}H-NMR (500 MHz, $CDCl_3$): δ = 1.11 (s, 1.48H), 1.16 (s, 1.32H), 1.55 (m, 1H), 1.76 (m, 2H), 2.13 (m, 1H), 2.53 (m, 3H), 2.85 (dddd, *J* = 14.2, 9.8, 5.0 Hz, 1H), 3.24 (m, 2H), 3.78 (m, 3H), 5.10 (dd, *J* = 17.6, 1.3 Hz, 0.49H), 5.12 (dd, *J* = 17.6, 1.4 Hz, 0.56H), 5.20 (dd, *J* = 10.9, 1.3 Hz, 0.46H), 5.25 (dd, *J* = 10.8, 1.5 Hz, 0.52H), 5.87 (dd, *J* = 17.5, 10.8 Hz, 0.53H), 5.91 (dd, *J* = 17.6, 10.9 Hz, 0.47H), 6.58 (m, 2H), 6.79 (m, 2H), 7.05 (m, 1H), 7.12 (m, 3H), 7.20 (m, 2H), 7.27 (m, 4H) ppm.

^{13}C-NMR (126 MHz, $CDCl_3$): δ = 18.7, 20.8, 30.8, 30.8, 33.6, 33.7, 34.8, 34.9, 39.4, 40.9, 45.6, 46.1, 55.9, 56.0, 61.3, 61.6, 113.7, 114.0, 114.5, 114.8, 115.1, 125.7, 125.7, 125.8, 128.3, 128.4, 128.5, 128.6, 142.3, 142.4, 143.1, 143.1, 143.9, 144.0, 144.1, 144.2, 151.3, 151.5 ppm.

HRMS ($C_{28}H_{33}ON$, $[M + H]^+$, pos. ESI): *m/z*: calcd: 400.2635, found: 400.2640, Δ = 1.4 ppm.

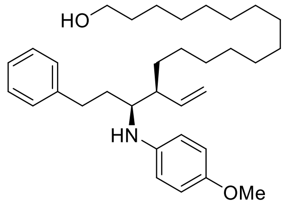

13s

13s was synthesized according to general procedure C. Flash column chromatography: silica gel, *n*-pentane/Et_2O 3:1 → 1:1 v/v, R_f = 0.24 (*n*-pentane/Et_2O 1:1 v/v). Further purification was achieved by reversed-phase chromatography using the method described in general procedure C. The product (**13s**, 54.9 mg, 111 µmol, 56%, dr = 4.0:1) was obtained as a yellow oil.

^{1}H-NMR (500 MHz, $CDCl_3$): δ = 1.27 (m, 25H), 1.50 (m, 1H), 1.57 (m, 2H), 1.71 (dddd, *J* = 13.7, 9.9, 7.8, 5.8 Hz, 0.36H), 1.84 (m, 0.28H), 1.92 (dddd, *J* = 13.9, 10.1, 7.4, 2.9 Hz, 0.86H), 2.27 (ddq, *J* = 13.8, 9.2, 4.4 Hz, 1H), 2.66 (m, 1.33H), 2.87 (ddd, *J* = 14.0, 9.6, 4.7 Hz, 0.85H), 3.25 (ddd, *J* = 10.2, 4.2, 2.9 Hz, 0.83H), 3.30 (ddd, *J* = 7.8, 5.1, 3.8 Hz, 0.24H), 3.64 (dd, *J* = 6.6 Hz, 2H), 3.75 (s, 0.68H), 3.76 (s, 2.30H), 4.95 (ddd, *J* = 17.1, 2.2, 0.7 Hz, 0.80H), 5.09 (m, 0.99H), 5.15 (dd, *J* = 10.4, 2.0 Hz, 0.20H), 5.57 (ddd, *J* = 17.0, 10.1, 9.4 Hz, 0.80H), 5.69 (ddd, *J* = 17.1, 10.3, 8.7 Hz, 0.20H), 6.53 (m, 2H), 6.77 (m, 2H), 7.17 (m, 3H), 7.27 (m, 2H).

^{13}C-NMR (126 MHz, $CDCl_3$): δ = 25.8, 27.7, 29.5, 29.6, 29.7, 29.7, 29.8, 30.5, 31.7, 32.9, 32.9, 33.0, 34.9, 47.2, 47.4, 55.9, 55.9, 56.9, 57.2, 63.2, 114.5, 115.0, 115.1, 115.2, 116.9, 117.4, 125.8, 128.4, 128.5, 128.6, 139.2, 139.3, 142.3, 142.4, 142.5, 142.6, 151.7, 151.8 ppm.

HRMS ($C_{33}H_{52}O_2N$, $[M + H]^+$, pos. ESI): *m/z*: calcd: 494.3993, found: 494.4002, Δ = 1.9 ppm.

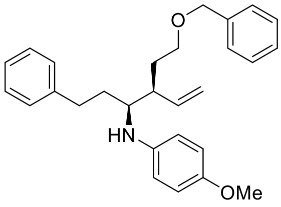

13t

13t was synthesized according to general procedure C. Flash column chromatography: silica gel, *n*-pentane/Et_2O 19:1 → 7:1 v/v, R_f = 0.14 (*n*-pentane/Et_2O 9:1 v/v). The product (**13t**, 56.7 mg, 136 µmol, 68%, dr = 5.3:1) was obtained as a yellow oil.

^{1}H-NMR (500 MHz, $CDCl_3$): δ = 1.55 (m, 1H), 1.63 (m, 1H), 1.77 (m, 1H), 1.86 (m, 0.36H), 1.95 (m, 0.86H), 2.51 (dddd, *J* = 9.3, 4.5 Hz, 0.85H), 2.58 (dddd, *J* = 8.9, 4.2 Hz, 0.19H), 2.66 (m, 1H), 2.74 (m, 0.21H), 2.88 (ddd, *J* = 13.8, 9.3, 4.7 Hz, 0.86H), 3.31 (ddd, *J* = 10.1, 4.5, 2.9 Hz, 1H), 3.42 (m, 3H), 3.76 (m, 3H), 4.44 (m, 2H), 4.97 (ddd, *J* = 17.1, 2.1, 0.8 Hz, 0.84H), 5.12 (m, 1H), 5.18 (dd, *J* = 10.4, 1.9 Hz, 0.17H), 5.61 (ddd, *J* = 17.1, 10.2, 9.4 Hz, 0.84H), 5.73 (ddd, *J* = 17.1, 10.3, 8.7 Hz, 0.16H), 6.53 (m, 2H), 6.76 (m, 2H), 7.18 (m, 3H), 7.32 (m, 7H) ppm.

^{13}C-NMR (126 MHz, $CDCl_3$): δ = 30.4, 30.8, 31.7, 32.7, 32.8, 33.0, 35.1, 43.9, 44.1, 55.9, 55.9, 56.6, 56.9, 68.5, 68.6, 73.0, 73.0, 114.5, 115.0, 115.1, 117.5, 117.8, 125.8, 127.6, 127.7, 127.7, 128.4, 128.5, 128.6, 138.3, 138.5, 138.5, 138.6, 142.2, 142.2, 142.3, 142.5, 151.7, 151.8 ppm.

HRMS ($C_{28}H_{34}O_2N$, $[M + H]^+$, pos. ESI): *m/z*: calcd: 416.2584, found: 416.2586, Δ = 0.5 ppm.

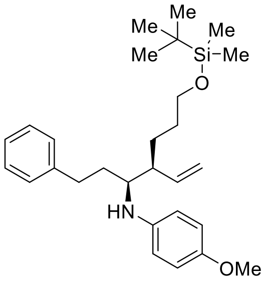

13u

13u was synthesized according to general procedure C. Flash column chromatography: silica gel, *n*-pentane/Et_2O 29:1 → 19:1 v/v, R_f = 0.35 (*n*-pentane/Et_2O 9:1 v/v). Further purification was achieved by reversed-phase chromatography using the method described in general procedure C. The product (**13u**, 56.4 mg, 124 µmol, 62%, dr = 4.3:1) was obtained as a yellow oil. Analytical data were in accordance with those measured for the allylic carbonate reaction.

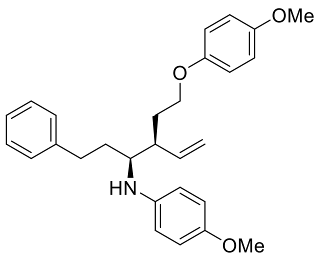

13v

13v was synthesized according to general procedure C. Flash column chromatography: silica gel, *n*-pentane/Et_2O 9:1 → 5:1 v/v, R_f = 0.35 (*n*-pentane/Et_2O 4:1 v/v). Further purification was achieved by reversed-phase chromatography using the method described in general procedure C. The product (**13v**, 43.6 mg, 101 µmol, 51%, dr = 4.9:1) was obtained as a yellow oil.

^{1}H-NMR (700 MHz, $CDCl_3$): δ = 1.57 (dddd, *J* = 14.0, 10.0, 9.2, 4.8 Hz, 1H), 1.75 (m, 1H), 1.94 (m, 2H), 2.57 (dddd, *J* = 9.4, 4.4 Hz, 0.84H), 2.67 (m, 1.20H), 2.75 (ddd, *J* = 13.8, 9.6, 5.9 Hz, 0.19H), 2.88 (ddd, *J* = 14.0, 9.5, 4.5 Hz, 0.86H), 3.33 (ddd, *J* = 10.1, 4.6, 2.9 Hz, 1H), 3.38 (m, 1H), 3.75 (s, 0.48H), 3.76 (s, 2.32H), 3.77 (s, 2.86H), 3.80 (m, 1H), 3.86 (ddd, *J* = 9.2, 7.1, 5.2 Hz, 0.87H), 3.92 (ddd, *J* = 9.3, 6.7, 5.2 Hz, 0.20H), 5.01 (ddd, *J* = 17.0, 2.0, 0.7 Hz, 0.84H), 5.15 (m, 0.99H), 5.21 (dd, *J* = 10.3, 1.8 Hz, 0.16H), 5.64 (ddd, *J* = 17.0, 10.2, 9.4 Hz, 0.83H), 5.75 (ddd, *J* = 17.2, 10.4, 8.7 Hz, 0.17H), 6.52 (m, 2H), 6.77 (m, 4H), 6.82 (m, 2H), 7.17 (m, 3H), 7.27 (m, 2H) ppm.

^{13}C-NMR (176 MHz, $CDCl_3$): δ = 29.8, 30.4, 30.4, 31.2, 32.8, 33.2, 35.0, 43.7, 44.1, 55.8, 55.9, 55.9, 56.7, 56.9, 66.7, 66.8, 114.5, 114.7, 114.7, 115.1, 115.1, 115.6, 117.9, 118.2, 125.9, 128.4, 128.4, 128.5, 128.6, 138.0, 138.2, 142.1, 142.2, 142.3, 142.4, 151.8, 151.9, 153.1, 153.2, 153.8, 153.8 ppm.

HRMS ($C_{28}H_{34}O_3N$, $[M + H]^+$, pos. ESI): *m/z*: calcd: 432.2533, found: 432.2536, Δ = 0.6 ppm.

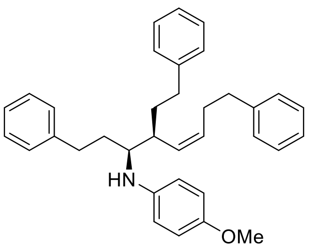

13w

13w was synthesized according to general procedure C. Flash column chromatography: silica gel, *n*-pentane/Et_2O 99:1 → 19:1 v/v, R_f = 0.31 (*n*-pentane/Et_2O 9:1 v/v). Further purification was achieved by reversed-phase chromatography using the method described in general procedure C. The product (**13w**, 21.4 mg, 43.7 µmol, 22%, dr > 19:1, *Z/E* >19:1) was obtained as a yellow oil.

^{1}H-NMR (500 MHz, $CDCl_3$): δ = 1.54 (m, 2H), 1.73 (dddd, *J* = 13.2, 10.5, 6.4, 4.0 Hz, 1H), 1.93 (dddd, *J* = 13.8, 9.9, 7.3, 2.9 Hz, 1H), 2.16 (m, 2H), 2.33 (ddd, *J* = 13.8, 10.4, 6.4 Hz, 1H), 2.52 (ddd, *J* = 13.7, 10.6, 5.1 Hz, 1H), 2.58 (m, 3H), 2.68 (m, 1H), 2.83 (ddd, *J* = 14.0, 9.6, 4.8 Hz, 1H), 3.26 (m, 2H), 3.76 (s, 3H), 5.25 (dddd, *J* = 10.8, 1.6 Hz, 1H), 5.64 (ddd, *J* = 10.8, 7.2 Hz, 1H), 6.45 (m, 2H), 6.74 (m, 2H), 7.02 (m, 2H), 7.09 (m, 2H), 7.17 (m, 6H), 7.26 (m, 6H) ppm.

^{13}C-NMR (126 MHz, $CDCl_3$): δ = 29.8, 33.0, 33.2, 33.9, 34.4, 36.0, 40.3, 55.9, 57.0, 114.4, 115.1, 125.8, 125.8, 125.9, 128.3, 128.4, 128.4, 128.5, 128.6, 130.4, 132.5, 141.8, 142.3, 142.4, 142.4, 151.7 ppm.

HRMS ($C_{35}H_{39}ON$, $[M + H]^+$, pos. ESI): *m/z*: calcd: 490.3104, found: 490.3112, Δ = 1.6 ppm.

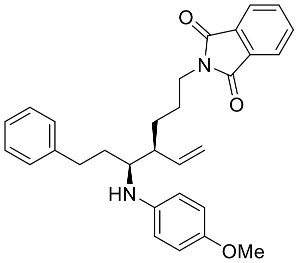

13x

13x was synthesized according to general procedure C. Flash column chromatography: silica gel, *n*-pentane/Et_2O 3:1 → 1:1 v/v, R_f = 0.14 (*n*-pentane/Et_2O 3:1 v/v). Further purification was achieved by reversed-phase chromatography using the method described in general procedure C. The product (**13x**, 50.7 mg, 108 µmol, 54%, dr = 4.5:1) was obtained as a yellow oil.

^{1}H-NMR (500 MHz, $CDCl_3$): δ = 1.34 (m, 0.89H), 1.50 (m, 3H), 1.68 (m, 1.20H), 1.83 (dddd, *J* = 14.9, 9.8, 6.4, 5.1 Hz, 0.21H), 1.92 (dddd, *J* = 13.8, 9.9, 7.2, 2.9 Hz, 0.83H), 2.30 (m, 1H), 2.65 (m, 1.26H), 2.86 (ddd, *J* = 14.0, 9.7, 4.6 Hz, 0.83H), 3.24 (ddd, *J* = 9.9, 4.9, 2.9 Hz, 1H), 3.31 (m, 1H), 3.63 (m, 2H), 3.74

(s, 0.61H), 3.75 (s, 2.32H), 4.98 (dd, J = 17.1, 2.0 Hz, 0.81H), 5.12 (m, 0.98H), 5.17 (dd, J = 10.3, 1.9 Hz, 0.18H), 5.54 (ddd, J = 17.1, 9.9 Hz, 0.82H), 5.67 (ddd, J = 17.1, 10.3, 8.9 Hz, 0.18H), 6.51 (m, 2H), 6.74 (m, 2H), 7.16 (m, 3H), 7.26 (m, 2H), 7.70 (m, 2H), 7.83 (m, 2H) ppm.

^{13}C-NMR (126 MHz, $CDCl_3$): δ = 26.7, 26.8, 27.8, 28.7, 30.4, 32.6, 32.8, 33.2, 34.9, 37.8, 38.0, 47.2, 47.5, 55.9, 55.9, 56.8, 57.2, 114.4, 115.0, 115.1, 117.7, 118.1, 123.2, 123.2, 125.8, 125.8, 128.4, 128.5, 128.6, 132.2, 133.9, 133.9, 138.3, 138.5, 142.1, 142.3, 142.4, 142.4, 151.7, 151.8, 168.4, 168.4 ppm.

HRMS ($C_{30}H_{33}O_3N_2$, $[M + H]^+$, pos. ESI): *m/z*: calcd: 469.2486, found: 469.2497, Δ = 2.4 ppm.

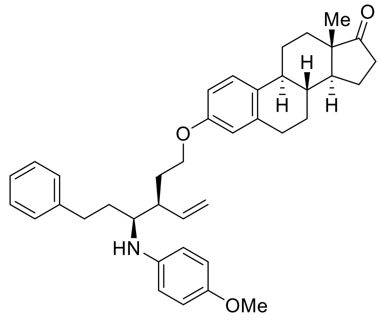

13y

13y was synthesized according to general procedure C. Flash column chromatography: silica gel, *n*-pentane/Et_2O 4:1 → 2:1 v/v, R_f = 0.16 (*n*-pentane/Et_2O 2:1 v/v). The product (**13y**, 76.7 mg, 133 µmol, 66%, dr = 4.9:1) was obtained as a yellow oil.

^{1}H-NMR (400 MHz, $CDCl_3$): δ = 0.92 (s, 3H), 1.53 (m, 8H), 1.75 (m, 1H), 2.02 (m, 6H), 2.28 (m, 1H), 2.40 (m, 1H), 2.51 (m, 1H), 2.65 (m, 2H), 2.88 (m, 3H), 3.35 (m, 1H), 3.76 (s, 0.56H), 3.76 (s, 2.28H), 3.87 (m, 2H), 5.02 (ddd, J = 17.0, 2.0, 0.7 Hz, 0.85H), 5.16 (m, 0.99H), 5.21 (dd, J = 10.4, 1.9 Hz, 0.17H), 5.65 (ddd, J = 17.1, 10.2, 9.4 Hz, 0.83H), 5.76 (ddd, J = 17.1, 10.4, 8.7 Hz, 0.17H), 6.55 (m, 2H), 6.59 (m, 1H), 6.66 (m, 1H), 6.77 (m, 2H), 7.18 (m, 4H), 7.28 (m, 2H) ppm.

^{13}C-NMR (101 MHz, $CDCl_3$): δ = 13.9, 21.7, 26.0, 26.6, 29.7, 30.4, 31.2, 31.7, 32.8, 33.2, 34.3, 35.0, 35.9, 38.5, 43.8, 44.1, 44.2, 48.1, 50.5, 55.9, 56.7, 56.9, 65.9, 66.0, 112.2, 112.2, 114.6, 114.6, 114.7, 115.1, 115.1, 117.9, 118.2, 125.9, 126.3, 128.4, 128.4, 128.5, 128.6, 132.0, 132.1, 137.7, 137.9, 138.2, 142.1, 142.2, 142.3, 142.4, 151.9, 151.9, 156.9, 157.0, 220.8 ppm.

HRMS ($C_{39}H_{48}O_3N$, $[M + H]^+$, pos. APCI): *m/z*: calcd: 578.3629, found: 578.3621, Δ = –1.3 ppm.

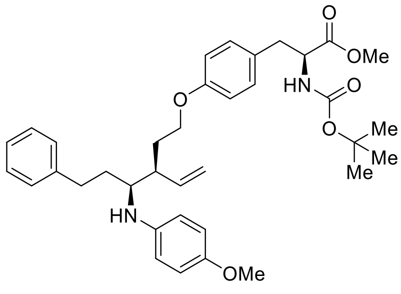

13z

13z was synthesized according to general procedure C. Flash column chromatography: silica gel, *n*-pentane/Et_2O 3:1 → 1:1 v/v, R_f = 0.38 (*n*-pentane/Et_2O 1:1 v/v). Further purification was achieved by reversed-phase chromatography using the method described in general procedure C. The product (**13z**, 80.7 mg, 134 µmol, 67%, dr = 4.9:1) was obtained as a yellow oil.

^{1}H-NMR (500 MHz, $CDCl_3$): δ = 1.43 (s, 9H), 1.57 (dddd, *J* = 14.2, 9.5, 4.8 Hz, 1H), 1.76 (m, 1H), 1.93 (m, 2H), 2.56 (dddd, *J* = 9.4, 4.3 Hz, 0.85H), 2.69 (m, 1.42H), 2.88 (ddd, *J* = 13.9, 9.4, 4.7 Hz, 0.92H), 3.02 (m, 2H), 3.35 (m, 2H), 3.71 (s, 3H), 3.75 (s, 0.56H), 3.76 (s, 2.30H), 3.84 (m, 2H), 4.35 (br s, 0.15H), 4.54 (br ddd, *J* = 6.2 Hz, 0.85H), 4.69 (s, 0.12H), 4.96 (br d, *J* = 8.4 Hz, 0.77H), 5.01 (ddd, *J* = 17.1, 2.0, 0.7 Hz, 0.89H), 5.14 (m, 0.99H), 5.21 (dd, *J* = 10.4, 1.8 Hz, 0.17H), 5.63 (ddd, *J* = 17.0, 10.2, 9.4 Hz, 0.83H), 5.75 (ddd, *J* = 17.2, 10.4, 8.7 Hz, 0.17H), 6.53 (m, 2H), 6.76 (m, 4H), 7.01 (m, 2H), 7.17 (m, 3H), 7.27 (m, 2H) ppm.

^{13}C-NMR (126 MHz, $CDCl_3$): δ = 28.4, 30.3, 30.4, 31.1, 32.7, 33.2, 35.0, 37.5, 43.7, 44.1, 52.2, 54.6, 55.9, 55.9, 56.7, 56.7, 56.8, 66.0, 66.1, 79.9, 114.5, 114.6, 115.1, 118.0, 118.3, 125.9, 127.9, 128.4, 128.4, 128.5, 128.6, 130.3, 137.8, 138.1, 142.0, 142.1, 142.2, 151.9, 151.9, 155.2, 158.0, 158.1, 172.5 ppm.

HRMS ($C_{36}H_{47}O_6N_2$, $[M + H]^+$, pos. ESI): *m/z*: calcd: 603.3429, found: 603.3436, Δ = 1.3 ppm.

3.6 Aldehyde Scope for Allylic Carbonates

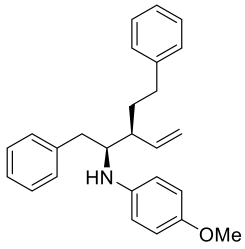

14a

14a was synthesized according to general procedure C. Flash column chromatography: silica gel, *n*-pentane/Et_2O 19:1 → 9:1 v/v, R_f = 0.12 (*n*-pentane/Et_2O 19:1 v/v). The product (**14a**, 53.5 mg, 144 µmol, 72%, dr = 3.2:1) was obtained as a yellow oil.

^{1}H-NMR (700 MHz, $CDCl_3$): δ = 1.75 (m, 1H), 1.86 (m, 0.25H), 1.91 (dddd, *J* = 13.8, 10.4, 6.8, 3.6 Hz, 0.83H), 2.34 (m, 0.26H), 2.38 (m, 0.79H), 2.47 (ddd, *J* = 13.8, 10.2, 6.4 Hz, 0.27H), 2.52 (ddd, *J* = 13.8, 10.0, 6.8 Hz, 0.82H), 2.61 (m, 1H), 2.73 (m, 1H), 2.83 (dd, *J* = 13.8, 6.0 Hz, 0.27H), 2.92 (dd, *J* = 14.1, 4.5 Hz, 0.82H), 3.37 (br s, 1H), 3.57 (ddd, *J* = 8.9, 4.6 Hz, 0.78H), 3.62 (ddd, *J* = 6.6, 3.6 Hz, 0.27H), 3.74 (s, 2.18H), 3.76 (s, 0.79H), 5.10 (ddd, *J* = 17.2, 2.1, 0.8 Hz, 0.78H), 5.20 (ddd, *J* = 17.2, 1.9, 1.0 Hz, 0.24H), 5.26 (dd, *J* = 10.3, 2.0 Hz, 0.76H), 5.33 (dd, *J* = 10.4, 1.9 Hz, 0.23H), 5.79 (ddd, *J* = 17.1, 10.2, 9.2 Hz, 0.76H), 5.86 (ddd, *J* = 17.3, 10.4, 8.7 Hz, 0.24H), 6.45 (m, 1.48H), 6.56 (m, 0.46H), 6.72 (m, 1.49H), 6.77 (m, 0.47H), 7.08 (m, 0.47H), 7.23 (m, 9.72H) ppm.

^{13}C-NMR (176 MHz, $CDCl_3$): δ = 33.0, 33.2, 33.8, 33.9, 37.3, 38.6, 45.2, 46.7, 55.9, 55.9, 59.1, 59.5, 114.8, 115.0, 115.1, 115.2, 118.0, 118.2, 125.7, 125.9, 126.1, 126.2, 128.3, 128.4, 128.5, 129.3, 129.3, 138.3, 139.2, 139.3, 139.5, 141.9, 142.0, 142.4, 142.4, 151.9, 152.0 ppm.

HRMS ($C_{26}H_{30}ON$, $[M + H]^+$, pos. APCI): *m/z*: calcd: 372.2322, found: 372.2327, Δ = 1.4 ppm.

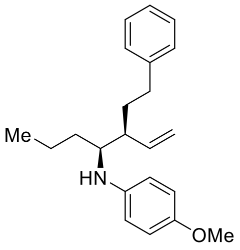

14b

14b was synthesized according to general procedure C. Flash column chromatography: silica gel, *n*-pentane/Et_2O 29:1 → 19:1 v/v, R_f = 0.21 (*n*-pentane/Et_2O 19:1 v/v). Further purification was achieved by reversed-phase chromatography using the method described in general procedure C. The product (**14b**, 39.3 mg, 121 µmol, 61%, dr = 3.8:1) was obtained as a yellow oil.

^{1}H-NMR (400 MHz, $CDCl_3$): δ = 0.90 (m, 3H), 1.28 (m, 2H), 1.55 (m, 2H), 1.69 (m, 1H), 1.80 (m, 1H), 2.34 (m, 1H), 2.50 (m, 1H), 2.68 (m, 1H), 3.27 (m, 2H), 3.76 (s, 3H), 5.03 (ddd, *J* = 17.1, 2.2, 0.8 Hz, 0.8H), 5.17 (m, 1.00H), 5.24 (ddd, *J* = 10.3, 1.9, 0.5 Hz, 0.21H), 5.68 (ddd, *J* = 17.1, 10.2, 9.4 Hz, 0.79H), 5.77 (ddd, *J* = 17.2, 10.4, 8.8 Hz, 0.21H), 6.52 (m, 2H), 6.76 (m, 2H), 7.18 (m, 3H), 7.28 (m, 2H) ppm.

^{13}C-NMR (101 MHz, $CDCl_3$): δ = 14.3, 19.7, 19.9, 32.5, 33.5, 33.7, 34.0, 34.0, 35.4, 46.9, 47.4, 55.9, 55.9, 57.6, 57.6, 114.3, 114.8, 115.0, 115.1, 117.3, 117.7, 125.7, 125.8, 128.3, 128.4, 128.4, 139.0, 139.1, 142.5, 142.6, 142.8, 151.6, 151.7 ppm.

HRMS ($C_{22}H_{30}ON$, $[M + H]^+$, pos. APCI): *m/z*: calcd: 324.2322, found: 324.2332, Δ = 3.1 ppm.

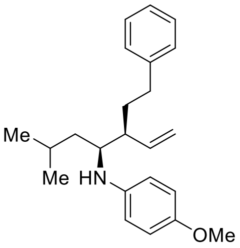

14c

14c was synthesized according to general procedure C. Flash column chromatography: silica gel, *n*-pentane/Et_2O 29:1 → 19:1 v/v, R_f = 0.20 (*n*-pentane/Et_2O 19:1 v/v). Further purification was achieved by reversed-phase chromatography using the method described in general procedure C. The product (**14c**, 39.5 mg, 117 µmol, 59%, dr = 4.6:1) was obtained as a yellow oil.

^{1}H-NMR (400 MHz, $CDCl_3$): δ = 0.90 (m, 6H), 1.21 (m, 0.85H), 1.32 (m, 1.27H), 1.74 (m, 3H), 2.37 (m, 1H), 2.52 (m, 1H), 2.67 (m, 1H), 3.25 (br s, 1H), 3.36 (ddd, *J* = 10.5, 3.4 Hz, 0.87H), 3.42 (ddd, *J* = 6.9, 3.2 Hz, 0.19H), 3.76 (s, 3H), 5.00 (ddd, *J* = 17.1, 2.2, 0.8 Hz, 0.82H), 5.17 (m, 0.98H), 5.23 (ddd, *J* = 10.4, 2.0, 0.6 Hz, 0.18H), 5.66 (ddd, *J* = 17.1, 10.2, 9.4 Hz, 0.82H), 5.78 (ddd, *J* = 17.2, 10.4, 8.7 Hz, 0.18H), 6.50 (m, 1.60H), 6.55 (m, 0.36H), 6.76 (m, 2H), 7.18 (m, 3H), 7.29 (m, 2H) ppm.

^{13}C-NMR (101 MHz, $CDCl_3$): δ = 21.9, 22.5, 23.3, 24.0, 24.8, 24.9, 32.2, 33.6, 34.1, 34.1, 40.4, 42.3, 46.9, 47.0, 55.7, 55.7, 55.9, 55.9, 114.3, 114.9, 115.1, 115.1, 117.3, 117.9, 125.7, 125.8, 128.3, 128.4, 128.5, 139.0, 139.0, 142.3, 142.5, 142.7, 151.6, 151.7 ppm.

HRMS ($C_{23}H_{32}ON$, $[M + H]^+$, pos. APCI): *m/z*: calcd: 338.2478, found: 324.2477, Δ = –0.3 ppm.

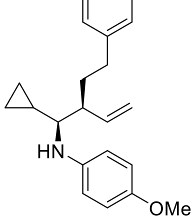

14d

14d was synthesized according to general procedure C. Flash column chromatography: silica gel, *n*-pentane/Et_2O 19:1 → 9:1 v/v, R_f = 0.34 (*n*-pentane/Et_2O 9:1 v/v). Further purification was achieved by reversed-phase chromatography using the method described in general procedure C. The product (**14d**, 20.0 mg, 62.2 µmol, 31%, dr = 1.6:1) was obtained as a yellow oil.

^{1}H-NMR (400 MHz, $CDCl_3$): δ = 0.23 (m, 1.35H), 0.31 (m, 0.60H), 0.40 (m, 1H), 0.49 (m, 1H), 0.89 (m, 1H), 1.74 (m, 0.52H), 1.86 (m, 1H), 1.95 (m, 0.46H), 2.46 (m, 2H), 2.69 (m, 1H), 2.81 (m, 1H), 3.74 (m, 3H), 5.06 (ddd, *J* = 17.1, 2.2, 0.8 Hz, 0.61H), 5.15 (ddd, *J* = 17.2, 2.0, 0.9 Hz, 0.39H), 5.21 (m, 1H), 5.85 (m, 1H), 6.49 (m, 1.16H), 6.54 (m, 0.80H), 6.73 (m, 2H), 7.17 (m, 3H), 7.26 (m, 2H) ppm.

^{13}C-NMR (101 MHz, $CDCl_3$): δ = 1.9, 2.5, 3.7, 3.9, 12.7, 14.3, 32.3, 33.8, 33.9, 34.1, 48.7, 48.8, 55.9, 55.9, 61.3, 61.8, 114.9, 114.9, 115.1, 117.0, 117.8, 125.7, 125.8, 128.3, 128.4, 128.5, 128.5, 139.3, 139.9, 142.3, 142.6, 142.6, 142.9, 151.9, 152.0 ppm.

HRMS ($C_{22}H_{28}ON$, $[M + H]^+$, pos. APCI): *m/z*: calcd: 322.2165, found: 322.2165, Δ = –0.2 ppm.

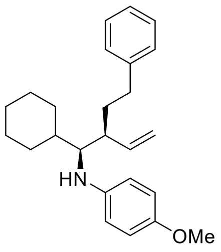

14e

14e was synthesized according to general procedure C. Flash column chromatography: silica gel, *n*-pentane/Et_2O 49:1 → 29:1 v/v, R_f = 0.31 (*n*-pentane/Et_2O 19:1 v/v). Further purification was achieved by reversed-phase chromatography using the method described in general procedure C. The product (**14e**, 24.7 mg, 67.9 µmol, 34%, dr = 8.6:1) was obtained as a yellow oil.

^{1}H-NMR (500 MHz, $CDCl_3$): δ = 1.08 (m, 5H), 1.40 (m, 1H), 1.54 (m, 0.52H), 1.63 (m, 1H), 1.73 (m, 5H), 1.84 (m, 1H), 2.50 (m, 2H), 2.62 (m, 1H), 3.07 (dd, J = 7.8, 4.1 Hz, 1H), 3.19 (s, 1H), 3.74 (s, 3H), 5.11 (m, 1.11H), 5.21 (ddd, J = 10.4, 2.0, 0.5 Hz, 0.91H), 5.75 (m, 1H), 6.53 (m, 2H), 6.73 (m, 2H), 7.13 (m, 3H), 7.25 (m, 2H) ppm.

^{13}C-NMR (126 MHz, $CDCl_3$): δ = 26.4, 26.6, 27.5, 30.0, 30.9, 31.7, 32.4, 33.6, 33.8, 33.9, 41.3, 42.5, 45.4, 47.7, 55.9, 56.0, 62.2, 62.7, 113.7, 113.8, 115.0, 115.0, 116.2, 117.2, 125.7, 128.3, 128.5, 128.6, 138.7, 141.0, 142.6, 142.7, 144.2, 144.4, 151.2, 151.3 ppm.

HRMS ($C_{25}H_{34}ON$, $[M + H]^+$, pos. APCI): *m/z*: calcd: 364.2635, found: 364.2641, Δ = 1.7 ppm.

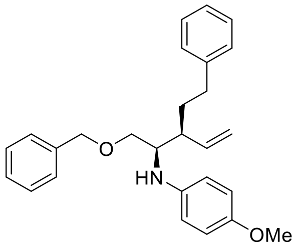

14f

14f was synthesized according to general procedure C. Flash column chromatography: silica gel, *n*-pentane/Et_2O 9:1 → 5:1 v/v, R_f = 0.20 (*n*-pentane/Et_2O 7:1 v/v). The product (**14f**, 49.0 mg, 122 µmol, 61%, dr = 1.8:1) was obtained as a yellow oil.

^{1}H-NMR (400 MHz, $CDCl_3$): δ = 1.66 (m, 0.75H), 1.77 (m, 0.40H), 1.88 (m, 0.40H), 2.02 (dddd, *J* = 13.7, 10.4, 6.8, 3.5 Hz, 0.67H), 2.44 (dddd, *J* = 9.8, 6.3, 3.4 Hz, 0.76H), 2.54 (m, 1.35H), 2.71 (m, 1H), 3.41 (m, 0.75H), 3.54 (m, 2.97H), 3.76 (s, 3H), 4.47 (s, 1.28H), 4.50 (s, 0.71H), 5.15 (m, 1.67H), 5.25 (ddd, *J* = 10.4, 2.0, 0.5 Hz, 0.36H), 5.74 (m, 1H), 6.57 (m, 2H), 6.77 (m, 2H), 7.17 (m, 3H), 7.31 (m, 7H) ppm.

^{13}C-NMR (101 MHz, $CDCl_3$): δ = 32.9, 33.2, 33.8, 44.8, 46.5, 55.9, 55.9, 57.2, 57.7, 69.9, 70.3, 73.3, 73.3, 114.8, 115.0, 115.0, 115.4, 117.5, 118.0, 125.7, 125.8, 127.6, 127.6, 127.7, 127.8, 128.3, 128.4, 128.4, 128.4, 128.4, 128.5, 128.5, 138.3, 138.4, 138.5, 139.5, 141.9, 142.0, 142.5, 152.1 ppm.

HRMS ($C_{27}H_{32}O_2N$, $[M + H]^+$, pos. ESI): *m/z*: calcd: 402.2428, found: 402.2433, Δ = 1.3 ppm.

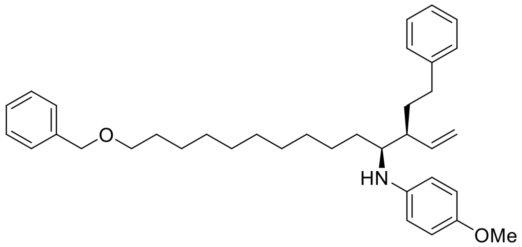

14g

14g was synthesized according to general procedure C. Flash column chromatography: silica gel, *n*-pentane/Et_2O 19:1 → 9:1 v/v, R_f = 0.49 (*n*-pentane/Et_2O 3:1 v/v). Further purification was achieved by reversed-phase chromatography using the method described in general procedure C. The product (**14g**, 65.3 mg, 124 µmol, 62%, dr = 3.8:1) was obtained as a yellow oil.

^{1}H-NMR (700 MHz, $CDCl_3$): δ = 1.27 (m, 12H), 1.36 (m, 2H), 1.50 (m, 1H), 1.65 (m, 4H), 1.80 (m, 1H), 2.33 (m, 1H), 2.50 (m, 1H), 2.68 (m, 1H), 3.26 (m, 2H), 3.47 (m, 2H), 3.75 (s, 3H), 4.51 (s, 2H), 5.02 (ddd, *J* = 16.9, 2.2, 0.8 Hz, 0.79H), 5.15 (ddd, *J* = 17.1, 2.0, 0.9 Hz, 0.22H), 5.18 (dd, *J* = 10.2, 2.1 Hz, 0.77H), 5.23 (dd, *J* = 10.3, 1.9 Hz, 0.20H), 5.67 (ddd, *J* = 17.1, 10.2, 9.4 Hz, 0.79H), 5.77 (ddd, *J* = 17.2, 10.3, 8.7 Hz, 0.21H), 6.50 (m, 1.56H), 6.54 (m, 0.44H), 6.76 (m, 2H), 7.17 (m, 3H), 7.28 (m, 3H), 7.35 (m, 4H) ppm.

^{13}C-NMR (176 MHz, $CDCl_3$): δ = 26.3, 26.5, 26.8, 29.5, 29.6, 29.6, 29.6, 29.8, 29.9, 31.4, 32.4, 33.0, 33.5, 34.0, 34.0, 46.8, 47.3, 55.9, 55.9, 57.8, 57.9, 70.6, 72.9, 114.4, 114.9, 115.0, 115.1, 117.3, 117.7, 125.7, 125.8, 127.5, 127.7, 128.3, 128.4, 128.5, 138.8, 139.1, 139.1, 142.5, 142.5, 142.6, 142.8, 151.7, 151.7 ppm.

HRMS ($C_{36}H_{50}O_2N$, $[M + H]^+$, pos. ESI): *m/z*: calcd: 528.3836, found: 528.3846, Δ = 1.9 ppm.

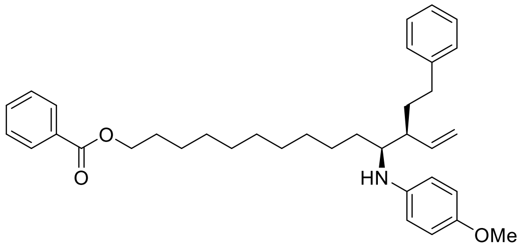

14h

14h was synthesized according to general procedure C. Flash column chromatography: silica gel, *n*-pentane/Et_2O 19:1 → 9:1 v/v, R_f = 0.42 (*n*-pentane/Et_2O 3:1 v/v). Further purification was achieved by reversed-phase chromatography using the method described in general procedure C. The product (**14h**, 76.6 mg, 141 µmol, 71%, dr = 3.5:1) was obtained as a yellow oil.

^{1}H-NMR (400 MHz, $CDCl_3$): δ = 1.27 (m, 12H), 1.45 (m, 3H), 1.74 (m, 5H), 2.34 (m, 1H), 2.50 (m, 1H), 2.68 (m, 1H), 3.25 (m, 1H), 3.75 (s, 3H), 4.33 (m, 2H), 5.02 (ddd, *J* = 17.1, 2.2, 0.8 Hz, 0.78H), 5.17 (m, 1.00H), 5.23 (dd, *J* = 10.4, 2.0 Hz, 0.22H), 5.67 (ddd, *J* = 17.0, 10.2, 9.3 Hz, 0.78H), 5.77 (ddd, *J* = 17.1, 10.4, 8.8 Hz, 0.22H), 6.52 (m, 2H), 6.75 (m, 2H), 7.17 (m, 3H), 7.27 (m, 2H), 7.45 (m, 2H), 7.55 (m, 1H), 8.06 (m, 2H) ppm.

^{13}C-NMR (101 MHz, $CDCl_3$): δ = 26.1, 26.5, 26.7, 28.8, 29.3, 29.6, 29.6, 29.6, 29.8, 29.8, 31.4, 32.4, 33.0, 33.5, 34.0, 34.0, 46.8, 47.3, 55.9, 55.9, 57.8, 57.9, 65.2, 114.4, 114.9, 115.0, 117.4, 117.7, 125.7, 125.8, 128.3, 128.4, 128.4, 128.4, 129.6, 130.6, 132.8, 139.1, 139.1, 142.5, 142.5, 142.6, 151.7, 151.7, 166.7 ppm.

HRMS ($C_{36}H_{48}O_3N$, $[M + H]^+$, pos. ESI): *m/z*: calcd: 542.3629, found: 542.3634, Δ = 1.0 ppm.

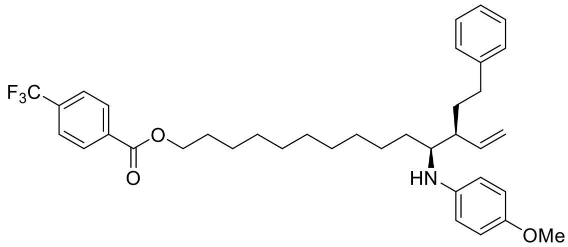

14i

14i was synthesized according to general procedure C. Flash column chromatography: silica gel, *n*-pentane/Et_2O 19:1 → 9:1 v/v, R_f = 0.27 (*n*-pentane/Et_2O 9:1 v/v). Further purification was achieved by reversed-phase chromatography using the method described in general procedure C. The product (**14i**, 73.2 mg, 120 µmol, 60%, dr = 3.6:1) was obtained as a yellow oil.

^{1}H-NMR (700 MHz, $CDCl_3$): δ = 1.25 (m, 10H), 1.35 (m, 3H), 1.43 (m, 2H), 1.49 (m, 1H), 1.59 (m, 1H), 1.69 (m, 1H), 1.78 (m, 3H), 2.33 (m, 1H), 2.49 (m, 1H), 2.67 (m, 1H), 3.23 (m, 1.66H), 3.28 (m, 0.28H), 3.75 (s, 3H), 4.35 (m, 2H), 5.01 (ddd, *J* = 17.2, 2.2, 0.8 Hz, 0.78H), 5.14 (ddd, *J* = 17.2, 2.0, 0.9 Hz, 0.23H), 5.17 (dd, *J* = 10.2, 2.1 Hz, 0.77H), 5.22 (dd, *J* = 10.3, 1.9 Hz, 0.21H), 5.66 (ddd, *J* = 17.0, 10.2, 9.4 Hz, 0.78H), 5.76 (ddd, *J* = 17.2, 10.3, 8.8 Hz, 0.22H), 6.50 (m, 1.53H), 6.54 (m, 0.45H), 6.75 (m, 2H), 7.13 (m, 0.43H), 7.18 (m, 2.50H), 7.27 (m, 2H), 7.71 (m, 2H), 8.16 (m, 2H) ppm.

^{13}C-NMR (176 MHz, $CDCl_3$): δ = 26.1, 26.5, 26.8, 28.7, 29.3, 29.5, 29.6, 29.6, 29.6, 29.6, 29.8, 29.8, 31.4, 32.4, 33.0, 33.6, 34.0, 34.0, 55.9, 55.9, 57.8, 57.9, 65.8, 114.4, 114.9, 115.1, 115.1, 117.4, 117.7, 123.7 (q, *J* = 272.6 Hz), 125.4 (q, *J* = 3.7 Hz), 125.7, 125.8, 128.3, 128.4, 128.5, 130.0, 133.8, 134.4 (q, *J* = 32.6 Hz), 139.1, 142.5, 142.5, 142.6, 142.8, 151.7, 151.7, 165.5 ppm.

^{19}F-NMR (659 MHz, $CDCl_3$): δ = –63.1 ppm.

HRMS ($C_{37}H_{47}O_3NF_3$, $[M + H]^+$, pos. ESI): *m/z*: calcd: 610.3503, found: 610.3048, Δ = 0.4 ppm.

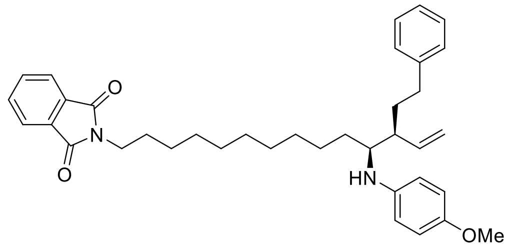

14j

14j was synthesized according to general procedure C. Flash column chromatography: silica gel, *n*-pentane/Et_2O 9:1 → 2:1 v/v, R_f = 0.33 (*n*-pentane/Et_2O 2:1 v/v). Further purification was achieved by reversed-phase chromatography using the method described in general procedure C. The product (**14j**, 82.1 mg, 145 µmol, 72%, dr = 3.8:1) was obtained as a yellow oil.

^{1}H-NMR (400 MHz, $CDCl_3$): δ = 1.25 (m, 15H), 1.46 (m, 1.20H), 1.57 (m, 0.87H), 1.67 (m, 3H), 1.78 (dddd, *J* = 13.3, 10.5, 6.8, 3.8 Hz, 1H), 2.33 (m, 1H), 2.49 (m, 1H), 2.66 (m, 1H), 3.23 (m, 2H), 3.67 (m, 2H), 3.74 (s, 3H), 5.01 (ddd, *J* = 17.1, 2.2, 0.7 Hz, 0.80H), 5.16 (m, 1.00H), 5.22 (ddd, *J* = 10.4, 2.0, 0.5 Hz, 0.21H), 5.66 (ddd, *J* = 17.1, 10.2, 9.4 Hz, 0.79H), 5.76 (ddd, *J* = 17.2, 10.4, 8.8 Hz, 0.21H), 6.50 (m, 2H), 6.74 (m, 2H), 7.16 (m, 3H), 7.26 (m, 2H), 7.70 (m, 2H), 7.84 (m, 2H) ppm.

^{13}C-NMR (101 MHz, $CDCl_3$): δ = 26.5, 26.7, 26.9, 28.7, 29.2, 29.5, 29.5, 29.6, 29.6, 29.8, 29.8, 32.4, 33.0, 33.5, 34.0, 34.0, 38.1, 46.8, 47.3, 55.9, 55.9, 57.8, 57.9, 114.4, 114.9, 115.0, 115.1, 117.3, 117.7, 123.2, 125.7, 125.8, 128.3, 128.4, 128.5, 132.3, 133.9, 139.1, 139.2, 142.5, 142.5, 142.6, 142.8, 151.6, 151.7, 168.5 ppm.

HRMS ($C_{37}H_{47}O_3N_2$, $[M + H]^+$, pos. ESI): *m/z*: calcd: 567.3581, found: 567.3590, Δ = 1.5 ppm.

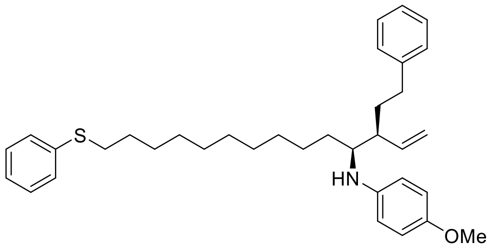

14k

14k was synthesized according to general procedure C. Flash column chromatography: silica gel, *n*-pentane/Et_2O 49:1 → 19:1 v/v, R_f = 0.21 (*n*-pentane/Et_2O 19:1 v/v). Further purification was achieved by reversed-phase chromatography using the method described in general procedure C. The product (**14k**, 88.6 mg, 167 µmol, 84%, dr = 3.8:1) was obtained as a yellow oil.

^{1}H-NMR (400 MHz, $CDCl_3$): δ = 1.24 (m, 12H), 1.40 (m, 2H), 1.65 (m, 4H), 1.78 (m, 1H), 2.33 (m, 1H), 2.49 (m, 1H), 2.66 (m, 1H), 2.91 (m, 2H), 3.24 (m, 1H), 3.74 (s, 3H), 5.01 (ddd, *J* = 17.0, 2.2, 0.7 Hz, 0.81H), 5.16 (m, 1.00H), 5.22 (dd, *J* = 10.4, 2.0 Hz, 0.21H), 5.66 (ddd, *J* = 17.1, 10.2, 9.4 Hz, 0.79H), 5.76 (ddd, *J* = 17.2, 10.3, 8.8 Hz, 0.21H), 6.51 (m, 2H), 6.74 (m, 2H), 7.16 (m, 4H), 7.29 (m, 6H) ppm.

^{13}C-NMR (101 MHz, $CDCl_3$): δ = 22.3, 26.5, 26.8, 28.7, 28.9, 29.2, 29.4, 29.5, 29.6, 29.6, 29.8, 29.8, 31.4, 32.4, 33.6, 33.7, 34.0, 34.0, 46.8, 47.3, 55.9, 55.9, 57.5, 58.0, 115.0, 115.1, 115.1, 117.4, 117.8, 124.1, 125.7, 125.7, 125.8, 128.3, 128.4, 128.5, 128.9, 128.9, 129.3, 131.0, 137.1, 139.1, 139.1, 142.5, 142.6, 144.2, 151.8 ppm.

HRMS ($C_{35}H_{48}ONS$, $[M + H]^+$, pos. ESI): *m/z*: calcd: 530.3451, found: 530.3462, Δ = 2.0 ppm.

3.7 Aldehyde Scope for Allenes

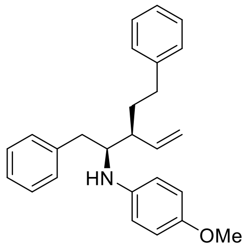

14a

14a was synthesized according to general procedure C. Flash column chromatography: silica gel, *n*-pentane/Et_2O 19:1 → 9:1 v/v, R_f = 0.16 (*n*-pentane/Et_2O 19:1 v/v). Further purification was achieved by reversed-phase chromatography using the method described in general procedure C. The product (**14a**, 33.8 mg, 91.0 µmol, 45%, dr = 3.2:1) was obtained as a yellow oil. Analytical data were in accordance with those measured for the allylic carbonate reaction.

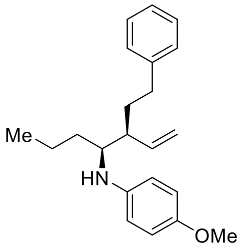

14b

14b was synthesized according to general procedure C. Flash column chromatography: silica gel, *n*-pentane/Et_2O 29:1 → 19:1 v/v, R_f = 0.23 (*n*-pentane/Et_2O 19:1 v/v). Further purification was achieved by reversed-phase chromatography using the method described in general procedure C. The product (**14b**, 29.4 mg, 90.9 µmol, 45%, dr = 3.9:1) was obtained as a yellow oil. Analytical data were in accordance with those measured for the allylic carbonate reaction.

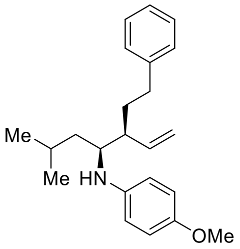

14c

14c was synthesized according to general procedure C. Flash column chromatography: silica gel, *n*-pentane/Et_2O 29:1 → 19:1 v/v, R_f = 0.20 (*n*-pentane/Et_2O 19:1 v/v). Further purification was achieved by reversed-phase chromatography using the method described in general procedure C. The product

(**14c**, 33.2 mg, 98.3 µmol, 49%, dr = 4.8:1) was obtained as a yellow oil. Analytical data were in accordance with those measured for the allylic carbonate reaction.

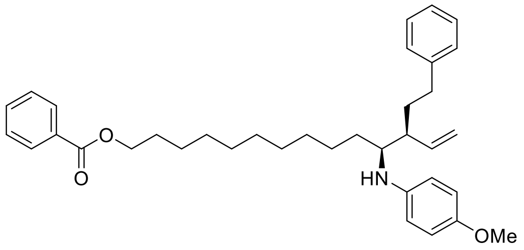

14h

14h was synthesized according to general procedure C. Flash column chromatography: silica gel, *n*-pentane/Et_2O 19:1 → 9:1 v/v, R_f = 0.18 (*n*-pentane/Et_2O 9:1 v/v). Further purification was achieved by reversed-phase chromatography using the method described in general procedure C. The product (**14h**, 56.8 mg, 105 µmol, 52%, dr = 3.5:1) was obtained as a yellow oil. Analytical data were in accordance with those measured for the allylic carbonate reaction.

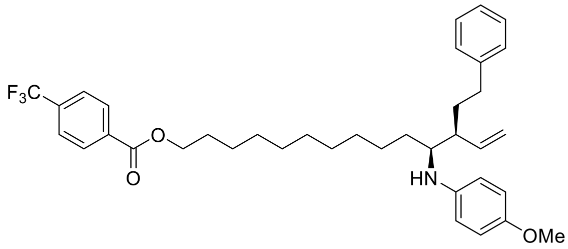

14i

14i was synthesized according to general procedure C. Flash column chromatography: silica gel, *n*-pentane/Et_2O 19:1 → 9:1 v/v, R_f = 0.24 (*n*-pentane/Et_2O 9:1 v/v). Further purification was achieved by reversed-phase chromatography using the method described in general procedure C. The product (**14i**, 52.2 mg, 85.6 µmol, 43%, dr = 3.6:1) was obtained as a yellow oil. Analytical data were in accordance with those measured for the allylic carbonate reaction.

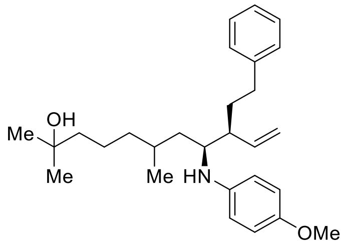

14l

14l was synthesized according to general procedure C. Flash column chromatography: silica gel, *n*-pentane/Et_2O 2:1 → 1:1 v/v, R_f = 0.24 (*n*-pentane/Et_2O 1:1 v/v). Further purification was achieved by reversed-phase chromatography using the method described in general procedure C. The product (**14l**, 36.9 mg, 87.1 µmol, 44%, dr = 5.3:4.1:1.2:1) was obtained as a yellow oil.

^{1}H-NMR (700 MHz, $CDCl_3$): δ = 0.85 (d, *J* = 6.6 Hz, 0.32H), 0.88 (d, *J* = 6.5 Hz, 1.50H), 0.90 (m, 1.19H), 1.17 (s, 2H), 1.30 (m, 15H), 1.70 (m, 3H), 2.36 (m, 1H), 2.50 (m, 1H), 2.66 (m, 1H), 3.36 (m, 0.88H), 3.43 (m, 0.21H), 3.74 (s, 0.25H), 3.75 (s, 0.98H), 3.75 (s, 1.65H), 4.99 (ddd, *J* = 17.1, 2.2, 0.8 Hz, 0.46H), 5.01 (ddd, *J* = 17.1, 2.1, 0.8 Hz, 0.36H), 5.16 (m, 1.00H), 5.23 (m, 0.19H), 5.66 (m, 0.81H), 5.77 (m, 0.19H), 6.49 (m, 1.59H), 6.54 (m, 0.40H), 6.75 (m, 2H), 7.14 (m, 0.43H), 7.18 (m, 2.52H), 7.27 (m, 2H) ppm.

^{13}C-NMR (176 MHz, $CDCl_3$): δ = 19.4, 19.7, 20.3, 20.8, 21.6, 21.7, 21.8, 29.2, 29.2, 29.3, 29.3, 29.3, 29.4, 29.4, 29.5, 29.6, 29.6, 29.7, 30.4, 32.0, 32.5, 33.3, 33.7, 34.0, 34.1, 34.1, 35.9, 36.6, 37.3, 38.1, 38.5, 38.7, 39.3, 40.5, 40.6, 44.2, 44.2, 44.2, 44.3, 46.4, 47.0, 47.0, 47.2, 55.4, 55.6, 55.6, 55.8, 55.9, 55.9, 55.9, 71.0, 71.1, 71.1, 114.3, 114.5, 114.9, 115.0, 115.1, 115.1, 115.1, 117.3, 117.5, 117.8, 118.0, 125.7, 125.8, 128.3, 128.4, 128.4, 128.5, 138.8, 138.9, 139.1, 139.2, 142.3, 142.5, 142.6, 142.6, 142.6, 142.7, 151.6, 151.7, 151.7 ppm.

HRMS ($C_{28}H_{42}O_2N$, $[M + H]^+$, pos. ESI): *m/z*: calcd: 424.3210, found: 424.3219, Δ = 2.1 ppm.

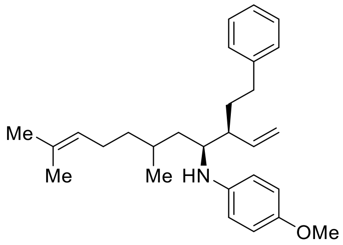

14m

14m was synthesized according to general procedure C. Flash column chromatography: silica gel, *n*-pentane/Et_2O 49:1 → 29:1 v/v, R_f = 0.28 (*n*-pentane/Et_2O 19:1 v/v). Further purification was achieved by reversed-phase chromatography using the method described in general procedure C. The product (**14m**, 37.9 mg, 93.4 µmol, 47%, dr = 4.5:3.4:1.0:1) was obtained as a yellow oil.

^{1}H-NMR (700 MHz, $CDCl_3$): δ = 0.85 (d, *J* = 6.6 Hz, 0.32H), 0.88 (d, *J* = 6.5 Hz, 1.41H), 0.90 (d, *J* = 6.7 Hz, 0.33H), 0.92 (d, *J* = 6.7 Hz, 1.07H), 1.11 (m, 1H), 1.26 (m, 2H), 1.44 (m, 1H), 1.58 (d, *J* = 1.3 Hz, 1.4H), 1.61 (d, *J* = 1.4 Hz, 1.61H), 1.70 (m, 6H), 1.88 (m, 0.50H), 1.97 (m, 1.66H), 2.36 (m, 1H), 2.51 (m, 1H), 2.67 (m, 1H), 3.21 (br s, 1H), 3.37 (m, 0.84H), 3.44 (m, 0.20H), 3.76 (m, 3H), 4.99 (ddd, *J* = 12.6, 2.1, 0.8 Hz, 0.44H), 5.02 (ddd, *J* = 12.6, 2.2, 0.8 Hz, 0.35H), 5.08 (m, 1H), 5.15 (m, 0.20H), 5.18 (dd, *J* = 10.3, 2.2 Hz, 0.81H), 5.23 (m, 0.20H), 5.66 (m, 0.80H), 5.77 (m, 0.19H), 6.50 (m, 1.60H), 6.54 (m, 0.40H), 6.76 (m, 2H), 7.14 (m, 0.41H), 7.18 (m, 2.53H), 7.28 (m, 2H) ppm.

^{13}C-NMR (176 MHz, $CDCl_3$): δ = 17.7, 17.8, 17.8, 19.4, 19.7, 20.2, 20.8, 25.4, 25.6, 25.7, 25.8, 25.8, 29.1, 29.2, 29.3, 29.6, 32.0, 32.5, 33.4, 33.7, 34.0, 34.1, 34.1, 36.3, 37.0, 37.7, 37.9, 38.1, 38.3, 38.4, 39.2, 40.4, 40.6, 46.5, 47.0, 47.2, 47.2, 55.4, 55.6, 55.6, 55.9, 55.9, 109.8, 109.8, 114.3, 114.4, 114.9, 115.0,

115.1, 115.1, 115.1, 117.2, 117.5, 117.8, 117.9, 124.8, 124.9, 124.9, 124.9, 125.7, 125.8, 125.8, 128.3, 128.4, 128.4, 128.4, 128.5, 131.2, 131.2, 131.2, 138.7, 138.9, 139.2, 139.2, 142.4, 142.5, 142.5, 142.6, 142.7, 142.7, 151.6, 151.7, 151.7, 151.7 ppm.

HRMS ($C_{28}H_{40}ON$, $[M + H]^+$, pos. ESI): *m/z*: calcd: 406.3104, found: 406.3109, Δ = 1.3 ppm.

3.8 Failed Substrates

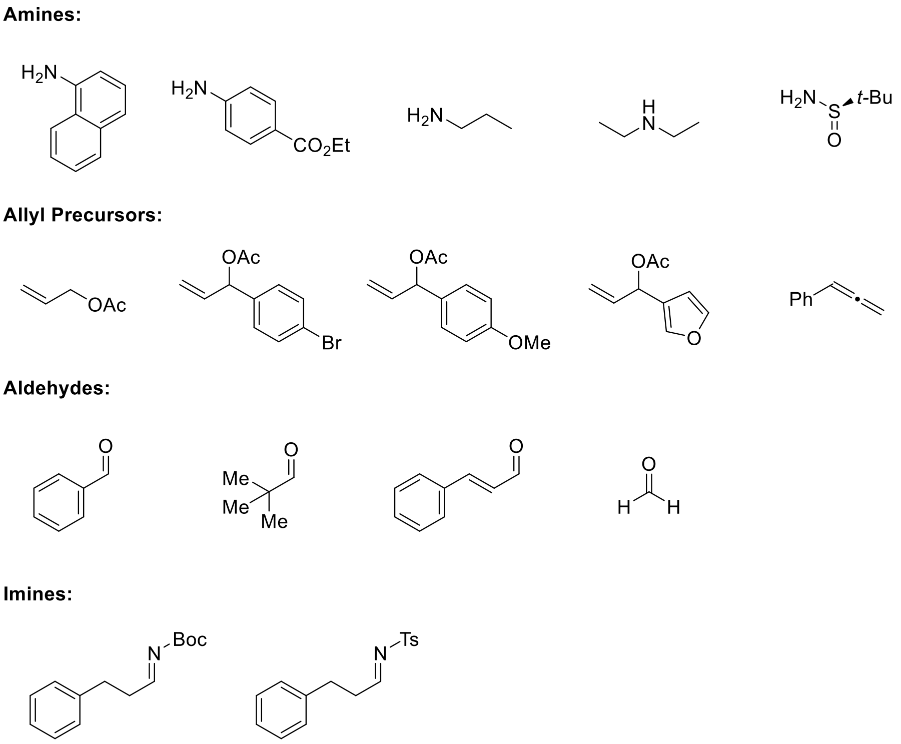

4. Scale-Up

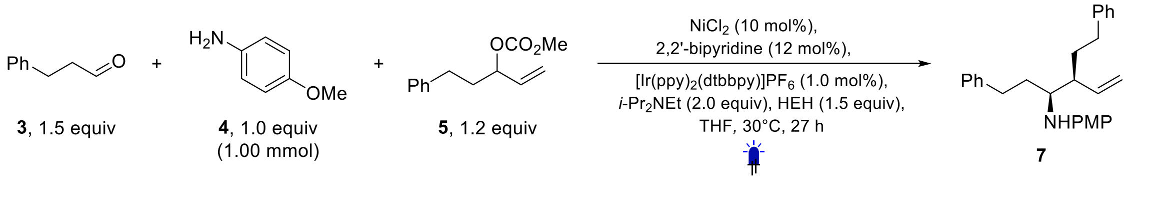

7 was synthesized on a 1.00 mmol scale according to general procedure C. Flash column chromatography: silica gel, *n*-pentane/Et_2O 19:1 → 9:1 v/v, R_f = 0.37 (*n*-pentane/Et_2O 9:1 v/v). The product (**7**, 261 mg, 676 µmol, 68%, dr = 3.8:1) was obtained as a yellow oil. Analytical data were in accordance with those reported above.

5. Mechanistic Experiments

5.1 Control Experiments

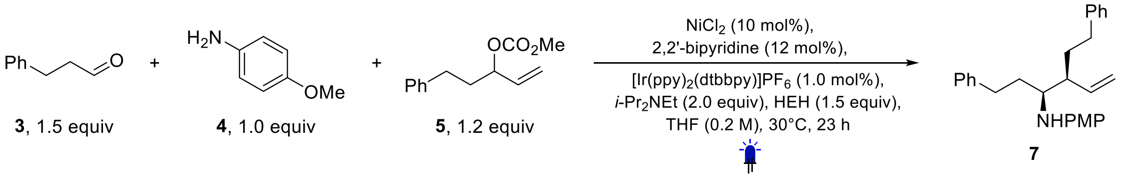

Entry	Control	NMR yield	dr
1	w/o $NiCl_2$	0%	–
2	w/o ligand	0%	–
3	w/o PC	0%	–
4	w/o Hantzsch ester	0%	–
5	w/o *i*-Pr_2NEt	0%	–
6	w/o light	0%	–

Reaction conditions: aldehyde (**3**, 1.5 equiv), *p*-anisidine (**4**, 200 µmol, 1.0 equiv), allylic carbonate (**5**, 1.2 equiv), $NiCl_2$ (10 mol%), 2,2'-bipyridine (12 mol%), $[Ir(ppy)_2(dtbbpy)]PF_6$ (1.0 mol%), *i*-Pr_2NEt (2.0 equiv), Hantzsch ester (1.5 equiv), THF (0.2 M), 30°C, blue LEDs, 23 h. Yields and diastereoselectivities were calculated from the ^{1}H-NMR spectrum of the crude product. 1,3,5-Trimethoxybenzene was used as an internal standard.

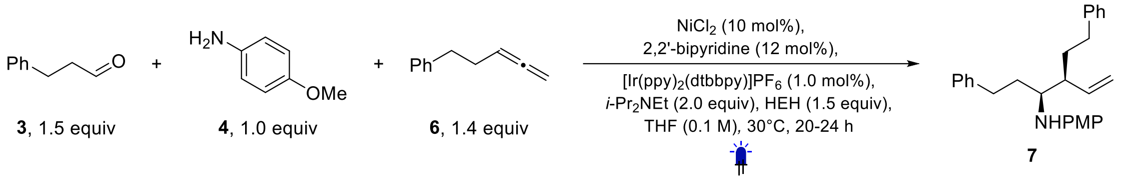

Entry	Control	NMR yield	dr
1	w/o $NiCl_2$	0%	–
2	w/o ligand	0%	–
3	w/o PC	0%	–
4	w/o Hantzsch ester	23% isolated	3.9:1
5	w/o *i*-Pr_2NEt	0%	–
6	w/o light	0%	–

Reaction conditions: aldehyde (**3**, 1.5 equiv), *p*-anisidine (**4**, 200 µmol, 1.0 equiv), allene (**6**, 1.4 equiv), $NiCl_2$ (10 mol%), 2,2'-bipyridine (12 mol%), $[Ir(ppy)_2(dtbbpy)]PF_6$ (1.0 mol%), *i*-Pr_2NEt (2.0 equiv), Hantzsch ester (1.5 equiv), THF (0.1 M), 30°C, blue LEDs, 20-24 h. Yields and diastereoselectivities were calculated from the ^{1}H-NMR spectrum of the crude product. 1,3,5-Trimethoxybenzene was used as an internal standard.

5.2 Detection of Radical Intermediates

Radical clock:

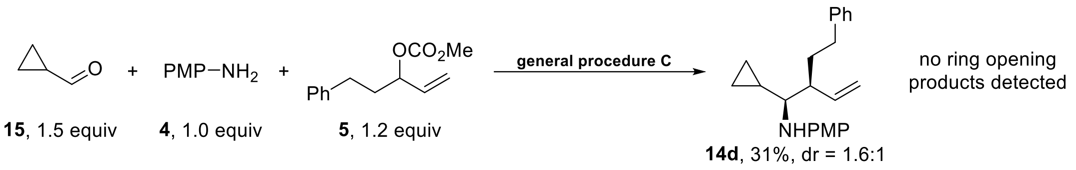

A radical clock experiment was performed according to general procedure C using cyclopropyl carbaldehyde (**15**). The homoallylic amine (**14d**) was obtained in 31% yield and no ring opening products were detected. We believe that the low yield can be explained by a poor performance of α-branched aldehydes in general. A similarly result was obtained for cyclohexyl carbaldehyde.

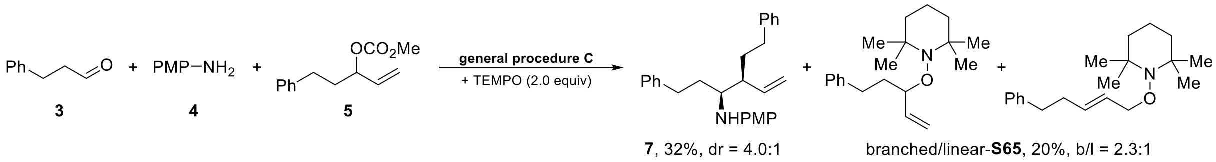

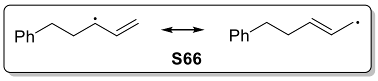

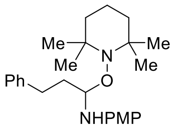

S67, not detected

The reaction was performed according to general procedure C adding TEMPO (2.0 equiv) as a radical trapping agent. TEMPO significantly inhibits the product formation. Next to 32% of **14d**, 20% of TEMPO allylation product (**S65**, b/l = 2.3:1) were isolated. This indicates the presence of allyl radical **S66**. No trapping adducts of α-amino radicals (**S67**) were found. When using the allene under these conditions the same products were observed. It was previously reported, that TEMPO itself can quench $[Ir(ppy)_2(dtbbpy)]PF_6$.[29] We believe that this and the reaction with allyl carbonate **5** are responsible for the diminished yield.

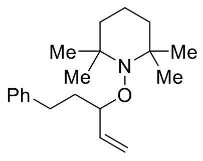

branched-**S65**

^{1}H-NMR (400 MHz, $CDCl_3$): δ = 1.11 (m, 12H), 1.44 (m, 6H), 1.81 (dddd, *J* = 13.3, 10.5, 7.9, 5.5 Hz, 1H), 2.05 (dddd, *J* = 13.3, 10.9, 6.3, 4.8 Hz, 1H), 2.63 (m, 2H), 4.14 (m, 1H), 5.13 (m, 2H), 5.87 (ddd, *J* = 17.1, 10.4, 8.4 Hz, 1H), 7.19 (m, 3H), 7.27 (m, 2H) ppm. Sample contains 12 mol% linear-**S65**.

^{13}C-NMR (101 MHz, $CDCl_3$): δ = 17.4, 20.4, 20.6, 31.7, 33.1, 34.1, 35.2, 36.2, 40.3, 59.2, 60.2, 85.2, 116.0, 125.7, 128.3, 128.5, 140.8, 142.6 ppm.

HRMS ($C_{20}H_{32}ON$, $[M + H]^+$, pos. ESI): *m/z*: calcd: 302.2478, found: 302.2484, Δ = 2.0 ppm.

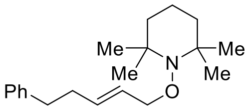

linear-**S65**

^{1}H-NMR (400 MHz, $CDCl_3$): δ = 1.11 (br s, 6H), 1.16 (br s, 5H), 1.44 (m, 4H), 1.54 (s, 2H), 2.36 (m, 2H), 2.71 (m, 2H), 4.22 (ddd, *J* = 6.0, 1.1 Hz, 2H), 5.60 (m, 1H), 5.73 (m, 1H), 7.19 (m, 3H), 7.27 (m, 2H) ppm. Sample contains 21 mol% branched-**S65**.

^{13}C-NMR (101 MHz, $CDCl_3$): δ = 17.3, 20.3, 29.8, 33.1, 34.4, 35.7, 39.8, 59.8, 78.3, 125.9, 126.3, 128.4, 128.5, 132.7, 142.0 ppm.

HRMS ($C_{20}H_{32}ON$, $[M + H]^+$, pos. ESI): *m/z*: calcd: 302.2478, found: 302.2486, Δ = 2.4 ppm.

5.3 Nickel Oxidation States:

Allylic Carbonates:

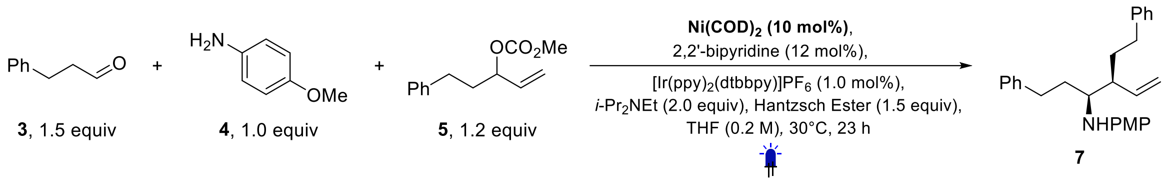

Entry	Deviation	NMR yield	dr
1	none	69%	ca. 4:1
2	w/o HE	45%	ca. 4:1
3	w/o *i*-Pr_2NEt	56%	ca. 4:1
4	w/o *i*-Pr_2NEt/HE	0%	–

Reaction conditions: aldehyde (**3**, 1.5 equiv), *p*-anisidine (**4**, 200 µmol, 1.0 equiv), allylic carbonate (**5**, 1.2 equiv), $Ni(COD)_2$ (10 mol%), 2,2'-bipyridine (12 mol%), $[Ir(ppy)_2(dtbbpy)]PF_6$ (1.0 mol%), *i*-Pr_2NEt (2.0 equiv), Hantzsch ester (1.5 equiv), THF (0.2 M), 30°C, blue LEDs, 23 h. Yields and diastereoselectivities were calculated from the ^{1}H-NMR spectrum of the crude product. 1,3,5-Trimethoxybenzene was used as an internal standard.

These experiments clearly show the necessity of Hantzsch ester and Hünig's base for product formation. We believe these reagents act as reducing agents for the Nickel catalyst and thereby keep the catalysis going. Apparently, both reagents can fulfill this role, however, together product formation is more efficient. Without Hantzsch ester and Hünig's base no product is formed.

To further support this hypothesis several experiments using stoichiometric amounts of $Ni(COD)_2$ were performed.

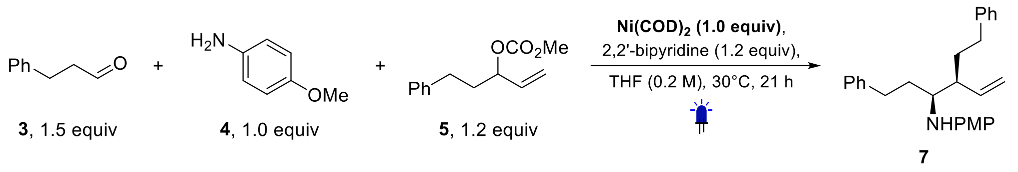

Entry	Irradiation	NMR yield	dr
1	Yes	56%	ca. 4:1
2	No	61%	ca. 4:1

Reaction conditions: aldehyde (**3**, 1.5 equiv), *p*-anisidine (**4**, 50.0 µmol, 1.0 equiv), allylic carbonate (**5**, 1.2 equiv), $Ni(COD)_2$ (1.0 equiv), 2,2'-bipyridine (1.2 equiv), THF (0.2 M), 30°C, blue LEDs, 21 h. Yields and diastereoselectivities were calculated from the ^{1}H-NMR spectrum of the crude product. 1,3,5-Trimethoxybenzene was used as an internal standard.

If 1.0 equiv of $Ni(COD)_2$ and 1.2 equiv of ligand are used the homoallylic amine can be formed without PC and reducing agents in yields comparable to the standard conditions with 10 mol% $Ni(COD)_2$. This confirms the role of HE and Hünig's base as electron donors. Entry 2 also rules out photocatalytic activity of the nickel-bipyridine complex. Our findings are in accordance with the following mechanistic scenario:

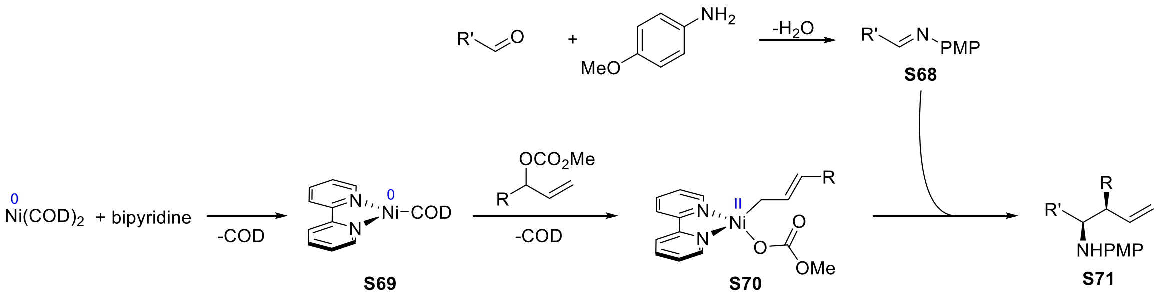

After formation of Ni(COD)(bipy) (**S69**), it can undergo an oxidative addition with the allylic carbonate yielding the Ni(II) complex **S70**. Strong evidence for this process can be found in literature.[30] Subsequently, **S70** performs the allylation reaction with imine **S68**.

Allenes:

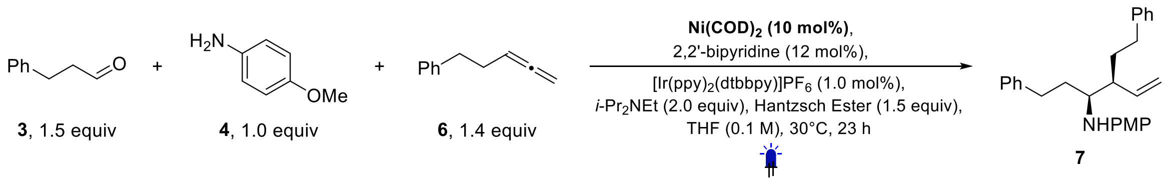

Entry	Deviation	NMR yield	dr
1	none	17%	–
2	w/o HE	0%	–
3	w/o *i*-Pr_2NEt	traces	–
4	w/o *i*-Pr_2NEt/HE	0%	–

Reaction conditions: aldehyde (**3**, 1.5 equiv), *p*-anisidine (**4**, 200 µmol, 1.0 equiv), allene (**6**, 1.4 equiv), $Ni(COD)_2$ (10 mol%), 2,2'-bipyridine (12 mol%), $[Ir(ppy)_2(dtbbpy)]PF_6$ (1.0 mol%), *i*-Pr_2NEt (2.0 equiv), Hantzsch ester (1.5 equiv), THF (0.1 M), 30°C, blue LEDs, 23 h. Yields and diastereoselectivities were calculated from the ^{1}H-NMR spectrum of the crude product. 1,3,5-Trimethoxybenzene was used as an internal standard.

$Ni(COD)_2$ turned out to be a poor catalyst for homoallylic amine formation from allenes only yielding 17% of product. These experiments also show the dependence of the reaction success from Hantzsch ester and Hünig's base which seems to be even stronger than for allylic carbonates. Next to their role as electron donors, both reagents could also serve as proton (or hydrogen atom) source for the formation of a nickel hydride complex. To further support this hypothesis two experiments using stoichiometric amounts of $Ni(COD)_2$ with and without proton source were performed.

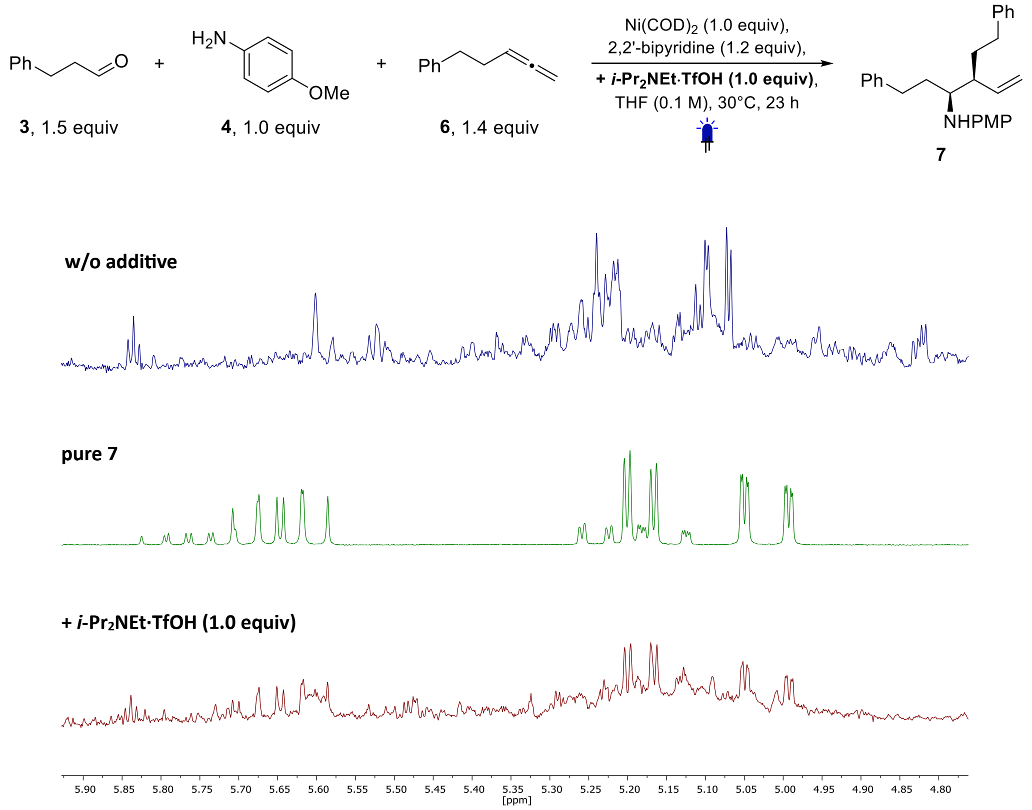

Figure 4: Effect of stoichiometric $Ni(COD)_2$ and the precences of *i*-$Pr_2NEt{\cdot}TfOH$ as a proton source. Reaction conditions: aldehyde (**3**, 1.5 equiv), *p*-anisidine (**4**, 50.0 µmol, 1.0 equiv), allene (**6**, 1.4 equiv), $Ni(COD)_2$ (1.0 equiv), 2,2'-bipyridine (1.2 equiv), *i*-$Pr_2NEt{\cdot}TfOH$ (1.0 equiv), THF (0.1 M), 30°C, blue LEDs, 23 h.

Unlike in the allylic substitution reaction, stoichiometric $Ni(COD)_2$ does not lead to product formation. This can be explained by the fact that Hantzsch ester and Hünig's base are involved in the formation of the nickel allyl species later reacting with the imine. The radical cations formed by oxidation of Hantzsch ester and Hünig's base are quite acidic[31] and may be involved in the formation of a Ni–H species. Addition of *i*-Pr_2NEt·TfOH as a proton source facilitates the product formation. Other potential proton sources present in the reaction mixture such as aniline and H_2O formed upon condensation with the aldehyde cannot sufficiently fulfil this role.

5.4 Deuterium labeling

To further back the formation of a Ni–H species we performed a deuterium labeling experiment. The reaction was performed according to general procedure C adding D_2O (5.0 equiv) as deuterium source. We believed that a rapid H/D exchange with other potential proton sources would lead to D incoperation in the product.

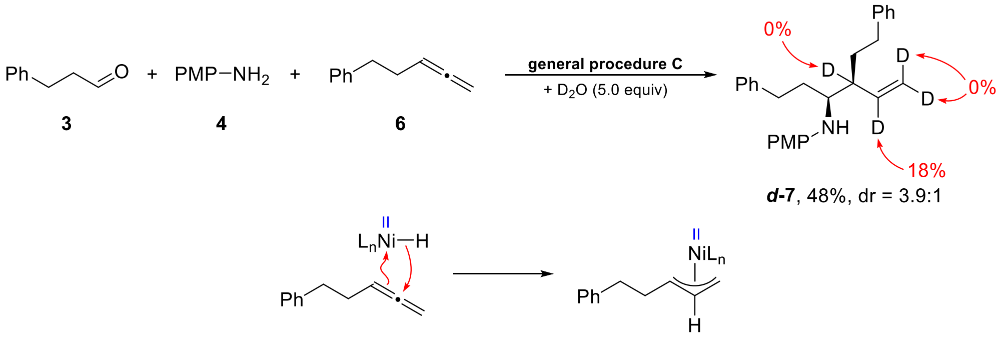

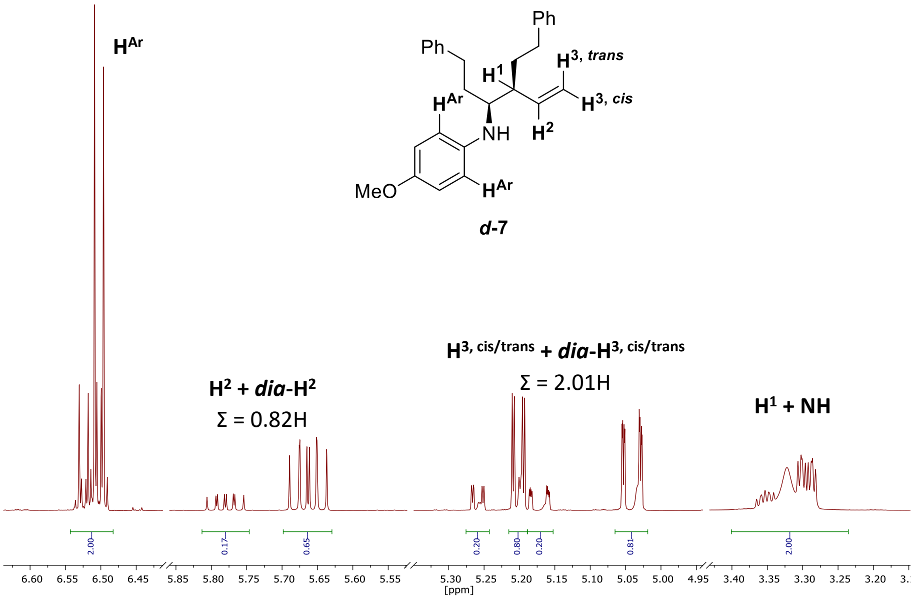

Figure 5: Relevant signals from the ^{1}H-NMR spectrum of ***d*-7** – Integrals indicate 18% deuterium replacement of H^2.

The ^{1}H-NMR spectrum from Figure 6 confirms the presence of deuterated product and regioselective incorporation of deuterium at the central carbon of the allene.

5.5 Stern-Volmer Quenching

To identify the reaction participants that undergo SET processes with $[Ir(ppy)_2(dtbbpy)]PF_6$ (PC), Stern-Volmer quenching studies were conducted. All measurements were performed on a Perkin Elmer LS 45 Fluorescence Spectrometer. Volumetric measurements for sample preparation were done with Eppendorf pipettes. 30 µL PC stock solution (1 mM) was mixed with increasing amounts of quencher solutions in Eppendorf tubes and filled to 1 mL with Eppendorf pipettes. 200 µL aliquots were filled into a fluorescence cuvette. The samples were excited at 450 nm and the fluorescence was detected between 500-675 nm. Fluorescence intensities were compared at 560 nm. This procedure was repeated for each quencher solution.

**c (PC)**	**30 µM**	**30 µM**	**30 µM**	**30 µM**	**30 µM**	**30 µM**
**c (Allene)**	0 mM	5 mM	10 mM	15 mM	20 mM	25 mM
**c (Allylic carbonate)**	0 mM	5 mM	10 mM	15 mM	20 mM	25 mM
**c (Aldehyde)**	0 mM	5 mM	10 mM	15 mM	20 mM	25 mM
**c (HEH)**	0 mM	5 mM	10 mM	15 mM	20 mM	25 mM
**c (*i*-Pr_2NEt)**	0 mM	5 mM	10 mM	15 mM	20 mM	25 mM
**c (HEH+*i*-Pr_2NEt)**	0 mM	5 mM	10 mM	15 mM	20 mM	25 mM
**c (*p*-Anisidine)**	0 mM	1.67 mM	3.33 mM	5 mM	6.67 mM	8.33 mM
**c (Imine)**	0 mM	1.67 mM	3.33 mM	5 mM	6.67 mM	8.33 mM

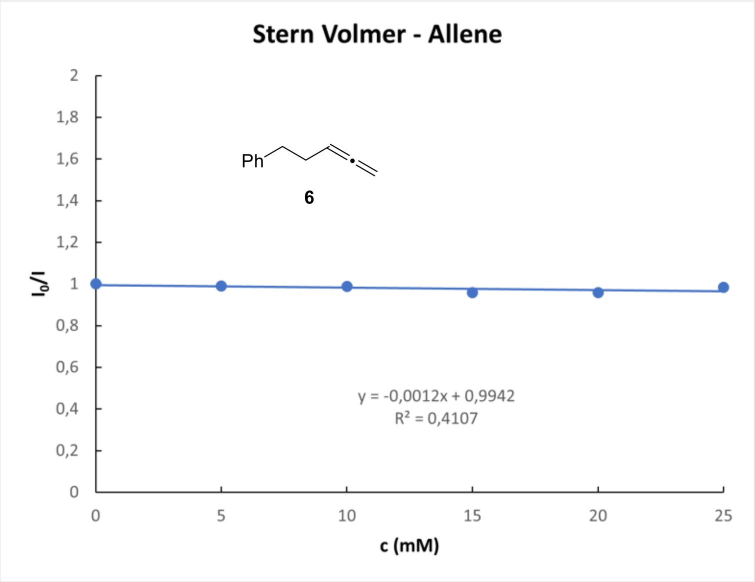

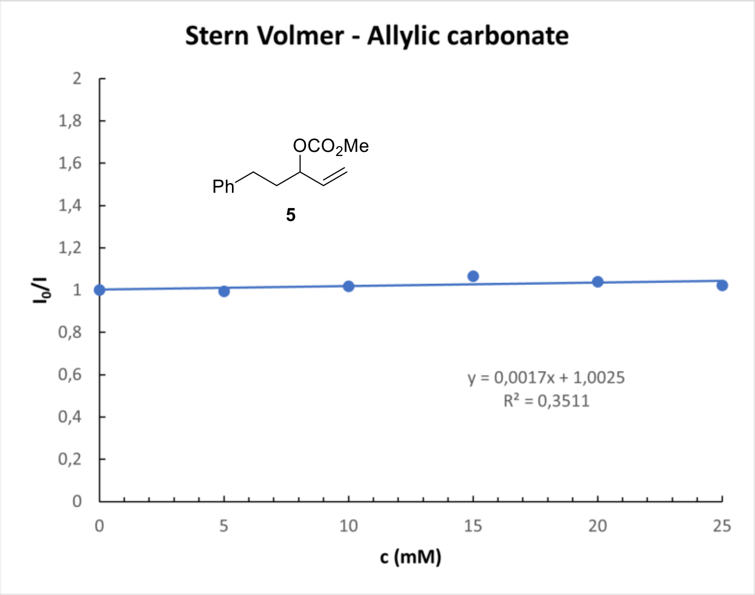
Stern Volmer - Allylic carbonate
OCO2Me
Ph
5
y = 0,0017x + 1,0025
R² = 0,3511
I0/I
c (mM)

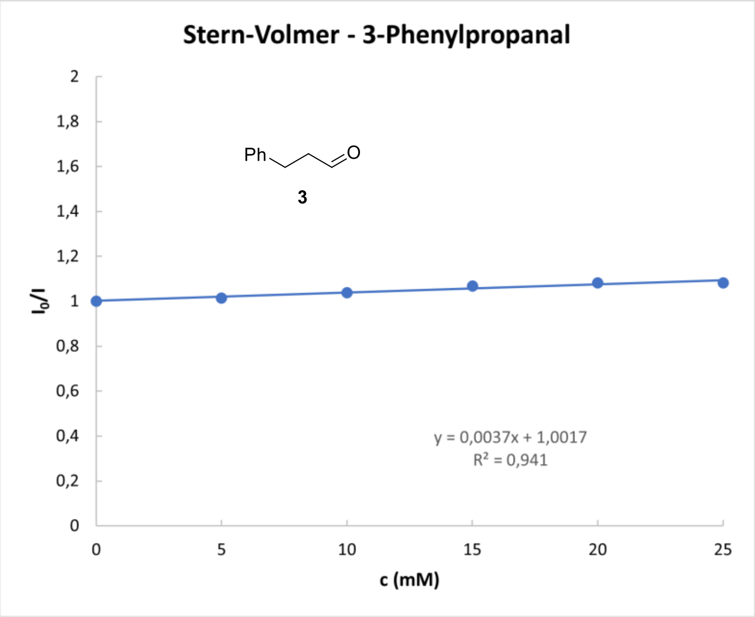
Stern-Volmer - 3-Phenylpropanal
Ph
O
3
y = 0,0037x + 1,0017
R² = 0,941
I0/I
c (mM)

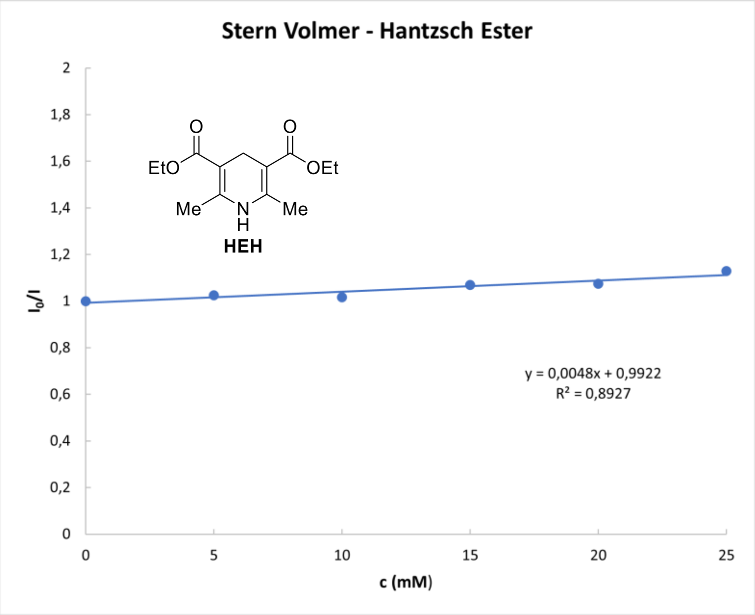
Stern Volmer - Hantzsch Ester
EtO
OEt
Me
Me
HEH
y = 0,0048x + 0,9922
R² = 0,8927
I0/I
c (mM)

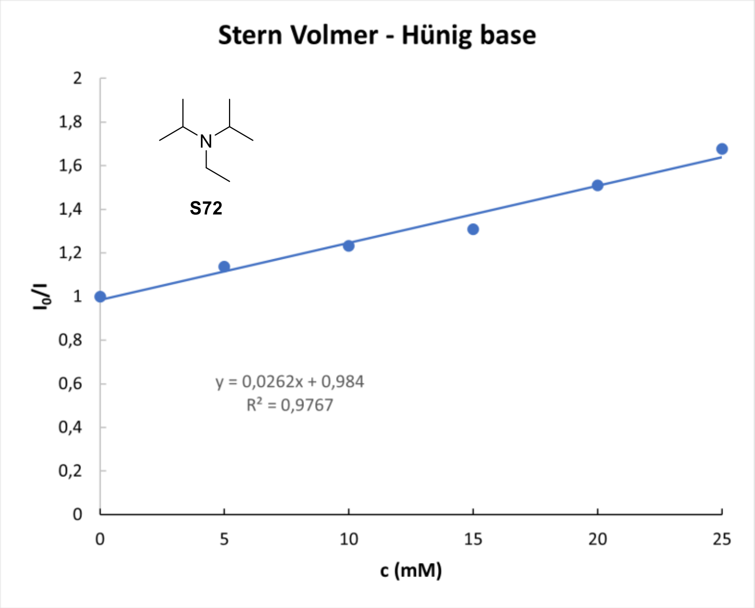
Stern Volmer - Hünig base
S72
y = 0,0262x + 0,984
R² = 0,9767
I0/I
c (mM)

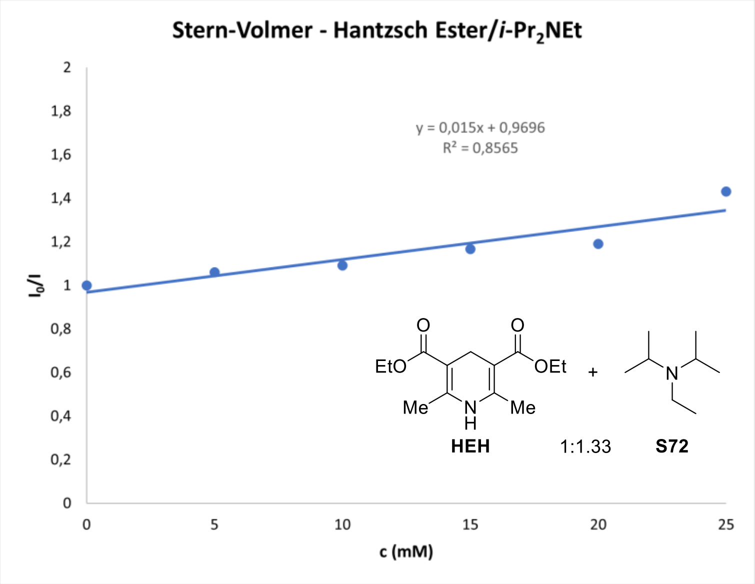
Stern-Volmer - Hantzsch Ester/i-Pr2NEt
y = 0,015x + 0,9696
R² = 0,8565
EtO
OEt
Me
Me
HEH
1:1.33
S72
I0/I
c (mM)

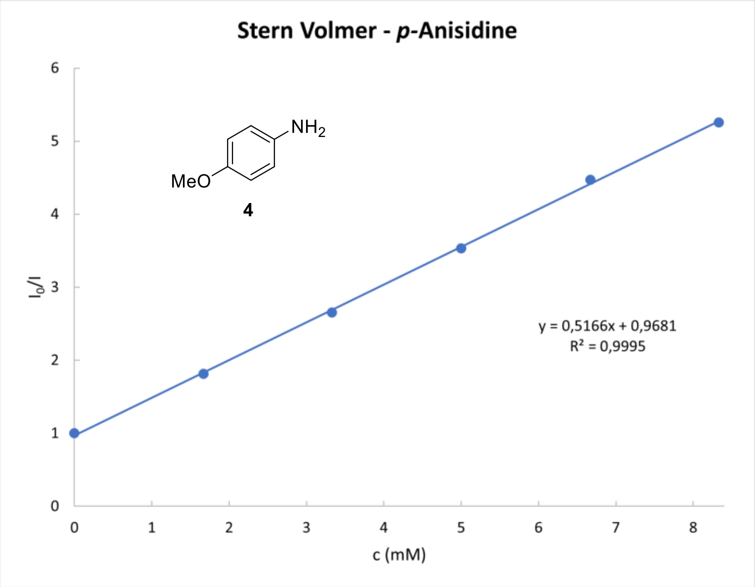
Stern Volmer - p-Anisidine
NH2
MeO
4
y = 0,5166x + 0,9681
R² = 0,9995
I0/I
c (mM)

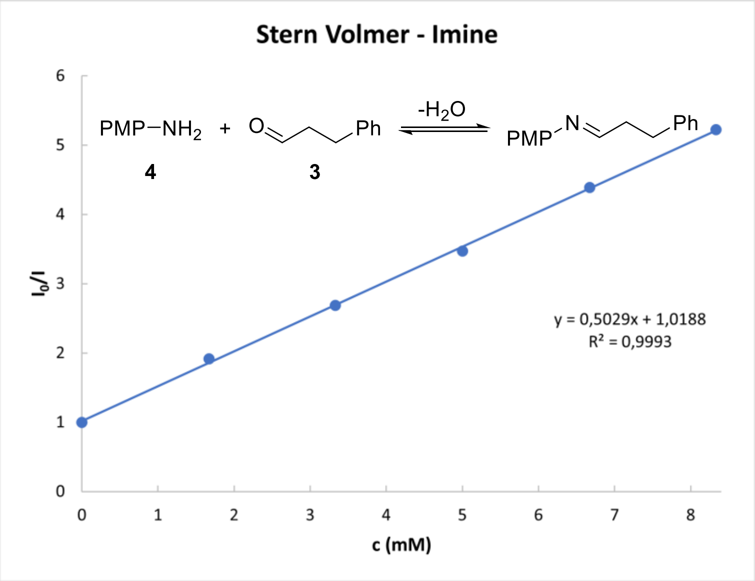

A 1:1 mixture of 3-phenylpropanal (**3**) and *p*-anisidine (**4**) was also probed as a potential quencher in Stern-Volmer experiments. A strong quenching similar to *p*-anisidine was observed. It was not possible to determine whether quenching is caused by the imine or by *p*-anisidine present in equilibrium with the imine. NMR studies show that imine formation occurs rapid at room temperature but not quantitative and *p*-anisidine is also present. The slope of the obtained curve is a little bit lower indicating a slightly decreased quenching. Literature suggests very low reduction potentials for imines derived from aliphatic aldehydes and anilines (E_{red} ≈ −3.0 V) which is beyond the scope of Ir[(ppy)$_2$(dtbbpy)]PF$_6$.[32] Photocatalytic oxidation of the imine also seems unlikely due to the low oxidation power of Ir[(ppy)$_2$(dtbbpy)]PF$_6$ [E(PC*/PC$_{red}$) = +0.66 V].[33]

The quenching of Ir[(ppy)$_2$(dtbbpy)]PF$_6$ by NiCl$_2$(bpy) was ruled out in a previously published Stern-Volmer experiment.[1]

6. Follow-Up Chemistry

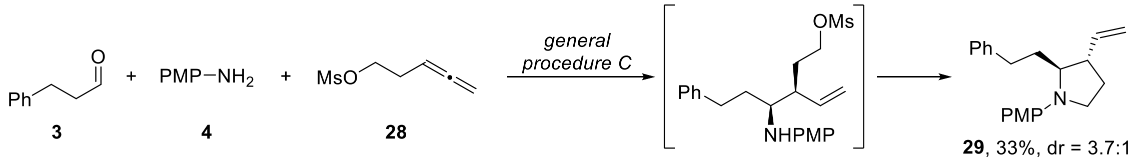

29 was synthesized according to general procedure C. Flash column chromatography: silica gel, *n*-pentane/Et_2O 19:1 → 9:1 v/v, R_f = 0.56 (*n*-pentane/Et_2O 7:1 v/v). Further purification was achieved by reversed-phase chromatography using the method described under general procedure C. The product (**29**, 20.1 mg, 65.2 µmol, 33%, dr = 3.7:1) was obtained as a yellow oil.

^{1}H-NMR (700 MHz, $CDCl_3$): δ = 1.74 (m, 1H), 1.84 (m, 1H), 2.04 (m, 1.29H), 2.25 (m, 0.87H), 2.65 (m, 1H), 2.73 (m, 1H), 2.77 (ddd, *J* = 11.9, 7.7, 3.8 Hz, 0.84H), 2.95 (m, 0.22H), 3.17 (ddd, *J* = 8.6 Hz, 0.23H), 3.24 (ddd, *J* = 8.6, 7.2 Hz, 0.86H), 3.41 (ddd, *J* = 8.9, 7.8, 3.7 Hz, 0.84H), 3.48 (m, 1H), 3.72 (m, 0.28H), 3.75 (s, 2.27H), 3.76 (s, 0.65H), 4.99 (ddd, *J* = 10.3, 1.2 Hz, 0.79H), 5.04 (ddd, *J* = 17.1, 1.4 Hz, 0.81H), 5.20 (ddd, *J* = 10.3, 1.9, 0.9 Hz, 0.22H), 5.24 (ddd, *J* = 17.1, 1.9, 1.2 Hz, 0.23H), 5.83 (ddd, *J* = 17.1, 10.3, 7.9 Hz, 0.78H), 6.04 (ddd, *J* = 17.2, 10.3, 8.0 Hz, 0.21H), 6.42 (m, 2H), 6.80 (m, 2H), 7.20 (m, 3H), 7.29 (m, 2H) ppm.

^{13}C-NMR (176 MHz, $CDCl_3$): δ = 29.4, 30.0, 32.4, 32.6, 33.2, 34.6, 47.0, 47.4, 47.8, 48.1, 56.0, 56.1, 60.8, 63.4, 112.5, 112.9, 114.0, 115.1, 115.1, 116.4, 125.9, 125.9, 128.4, 128.4, 128.5, 128.5, 137.5, 141.1, 142.0, 142.1, 142.4, 150.7, 150.8 ppm.

HRMS ($C_{21}H_{26}ON$, $[M + H]^+$, pos. APCI): *m/z*: calcd: 308.2009, found: 308.2014, Δ = 1.7 ppm.

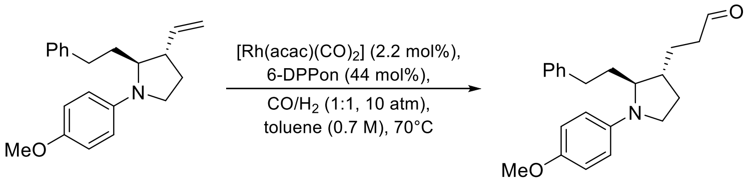

Modified general procedure B: A mixture of $[Rh(acac)(CO)_2]$ (0.52 mg, 2.00 µmol, 2.2 mol%), 6-DPPon (11.2 mg, 40.0 µmol, 44 mol%), and the olefin **29** (27.9 mg, 90.7 µmol, 1.0 equiv) dissolved in dry toluene (1 mL) was added to a previously dried 10 mL glass inlet equipped with a cross-shaped magnetic stirring bar. Afterwards, the glass inlet was inserted into a steel autoclave which was then sealed. The closed autoclave was then purged three times with CO/H_2-gas (1:1, 5 bar), followed by the adjustment of the reaction pressure to 10 bar. The reaction mixture was stirred in the autoclave for 17 h at 70°C. Afterwards, the autoclave was allowed to cool to rt and depressurized. The solvent was removed in vacuo and the residue was purified by flash column chromatography over silica gel [*n*-

pentane/Et_2O 3:1 → 2:1 v/v, R_f = 0.19 (*n*-pentane/Et_2O 3:1 v/v)]. The aldehyde **30** (25.1 mg, 74.4 µmol, 82%) was obtained as an oil.

^{1}H-NMR (500 MHz, $CDCl_3$): δ = 1.67 (m, 4H), 2.01 (m, 1H), 2.10 (m, 1H), 2.22 (m, 1H), 2.49 (ddd, *J* = 7.5, 1.6 Hz, 1.63H), 2.64 (m, 2.55H), 3.14 (ddd, *J* = 9.4, 7.2 Hz, 0.24H), 3.21 (ddd, *J* = 9.1, 7.1 Hz, 0.83H), 3.36 (m, 1.61H), 3.44 (dd, *J* = 8.8 Hz, 0.23H), 3.75 (s, 2.25H), 3.76 (s, 0.61H), 6.40 (m, 1.56H), 6.46 (m, 0.45H), 6.81 (m, 2H), 7.20 (m, 3H), 7.30 (m, 2H), 9.78 (t, *J* = 1.6 Hz, 0.64H), 9.84 (t, *J* = 1.5 Hz, 0.19H) ppm.

^{13}C-NMR (126 MHz, $CDCl_3$): δ = 22.1, 26.3, 28.7, 29.3, 32.3, 32.7, 33.5, 34.5, 42.3, 42.4, 42.6, 43.0, 46.9, 48.0, 56.1, 59.8, 63.9, 112.4, 112.6, 115.1, 115.2, 125.9, 126.0, 128.4, 128.4, 128.5, 141.7, 142.0, 142.3, 142.6, 150.6, 150.7, 201.8, 202.0 ppm.

HRMS ($C_{22}H_{28}O_2N$, $[M + H]^+$, pos. ESI): *m/z*: calcd: 338.2115, found: 338.2115, Δ = 0.2 ppm.

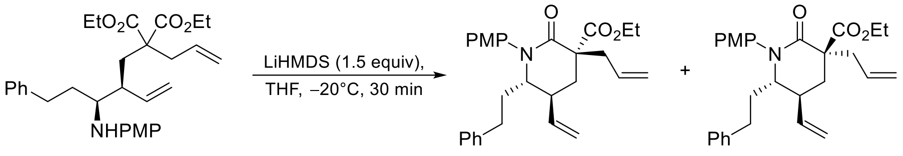

5-Amino ester **13q** (30.0 mg, 60.8 µmol, 1.0 equiv) was dissolved in THF (1 mL) and cooled to −20°C. Then, LiHMDS (1 M in THF, 93.0 µL, 93.0 µmol, 1.5 equiv) was added dropwise over 1 min and the mixture was stirred at −20°C for 90 min and at room temp for 1 h. Subsequently, sat aq NH_4Cl solution (2 drops), EtOAc (5 mL) and Na_2SO_4 were added. The mixture was filtered over silica and concentrated under reduced pressure. The residue was purified by flash column chromatography over silica gel [*n*-pentane/Et_2O 2:1 → 1:1 v/v, $R_{f,\,\mathbf{31}}$ = 0.27, $R_{f,\,dia\text{-}\mathbf{31}}$ = 0.21 (*n*-pentane/Et_2O 1:1 v/v)]. After reversed-phase chromatography, **31** (9.80 mg, 21.9 µmol, 36%) and *dia*-**31** (10.5 mg, 23.5 µmol, 39%) were obtained as colorless oils.

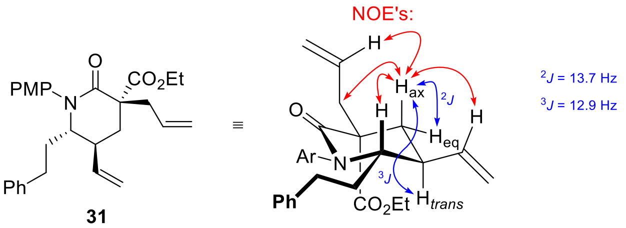

^{1}H-NMR (700 MHz, $CDCl_3$): δ = 1.35 (t, *J* = 7.1 Hz, 3H), 1.58 (dddd, *J* = 14.6, 12.1, 4.5 Hz, 1H), 1.77 (dddd, *J* = 15.0, 12.2, 5.5, 2.9 Hz, 1H), 1.96 (dd, *J* = 13.7, 12.9 Hz, 1H, H_{ax}), 2.17 (dd, *J* = 13.6, 3.5 Hz, 1H, H_{eq}), 2.44 (ddd, *J* = 13.5, 12.0, 5.5 Hz, 1H), 2.55 (m, 2H), 2.78 (m, 1H, H_{trans}), 2.86 (dddd, *J* = 13.7, 6.3, 1.4 Hz, 1H), 3.71 (ddd, *J* = 10.0, 4.5, 2.9 Hz, 1H), 3.82 (s, 3H), 4.29 (m, 2H), 5.11 (m, 2H), 5.17 (dd, *J* = 10.2, 1.4

Hz, 1H), 5.23 (ddd, *J* = 17.0, 1.1 Hz, 1H), 5.70 (ddd, *J* = 17.0, 10.2, 8.3 Hz, 1H), 5.79 (dddd, *J* = 17.1, 10.1, 8.3, 6.3 Hz, 1H), 6.90 (m, 2H), 6.95 (m, 2H), 7.12 (m, 1H), 7.18 (m, 4H) ppm.

^{13}C-NMR (176 MHz, $CDCl_3$): δ = 14.4, 29.1, 32.8, 35.4, 39.1, 40.8, 54.1, 55.6, 61.7, 63.4, 114.5, 117.4, 119.2, 125.9, 128.2, 128.4, 128.6, 133.4, 134.0, 138.9, 141.6, 158.4, 169.2, 173.0 ppm.

HRMS ($C_{28}H_{34}O_4N$, $[M + H]^+$, pos. ESI): *m/z*: calcd: 448.2482, found: 448.2482, Δ = 0.0 ppm.

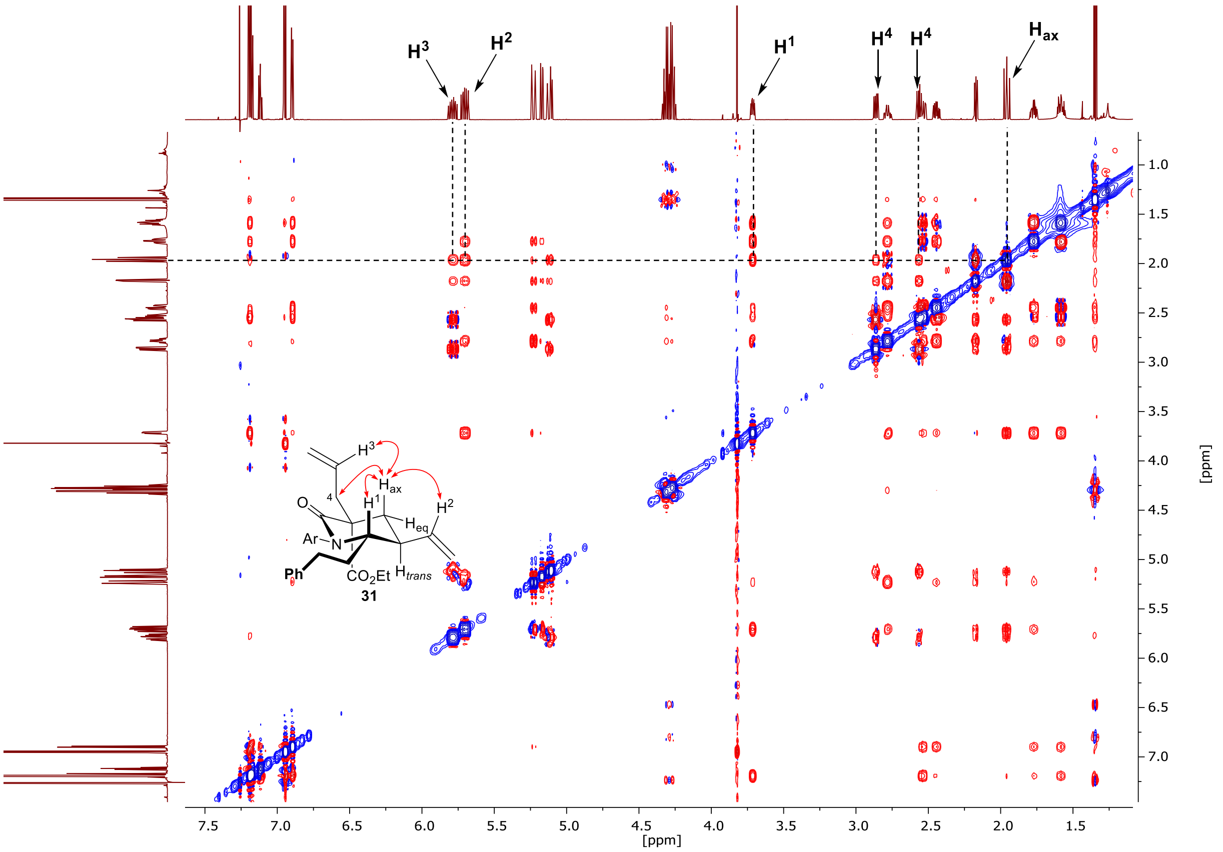

Figure 6: NOESY spectrum of **31**.

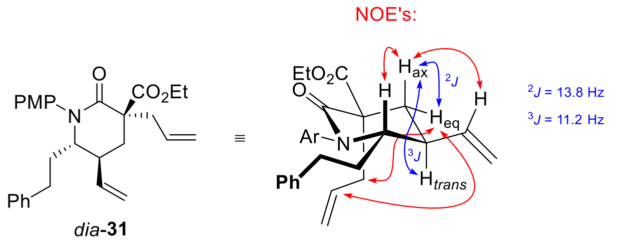

^{1}H-NMR (700 MHz, $CDCl_3$): δ = 1.25 (t, *J* = 7.2 Hz, 3H), 1.67 (dddd, *J* = 14.7, 10.2, 5.2 Hz, 1H), 1.84 (dddd, *J* = 14.4, 11.1, 6.2, 3.2 Hz, 1H), 2.07 (dd, *J* = 13.8, 3.8 Hz, 1H, H_{eq}), 2.37 (dd, *J* = 13.8, 11.2 Hz, 1H, H_{ax}), 2.49 (m, 2H), 2.72 (dddd, *J* = 14.0, 8.1, 1.1 Hz, 1H), 2.78 (m, 1H, H_{trans}), 3.02 (dddd, *J* = 14.0, 6.5, 1.3 Hz, 1H), 3.77 (ddd, *J* = 8.6, 5.1, 3.2 Hz, 1H), 3.81 (s, 3H), 4.14 (m, 1H), 4.20 (m, 1H), 5.17 (dd, *J* = 10.2, 1.5

Hz, 1H), 5.21 (m, 3H), 5.80 (ddd, J = 17.1, 10.3, 8.4 Hz, 1H), 5.92 (dddd, J = 16.8, 10.1, 8.0, 6.5 Hz, 1H), 6.92 (m, 4H), 7.17 (m, 5H) ppm.

^{13}C NMR (176 MHz, $CDCl_3$): δ = 14.1, 29.9, 33.1, 33.2, 37.8, 39.7, 53.8, 55.6, 61.6, 63.2, 114.5, 117.2, 118.9, 126.1, 128.2, 128.5, 128.8, 133.3, 134.0, 138.9, 141.2, 158.5, 170.3, 172.1 ppm.

HRMS ($C_{28}H_{34}O_4N$, $[M + H]^+$, pos. ESI): *m/z*: calcd: 448.2482, found: 448.2483, Δ = 0.1 ppm.

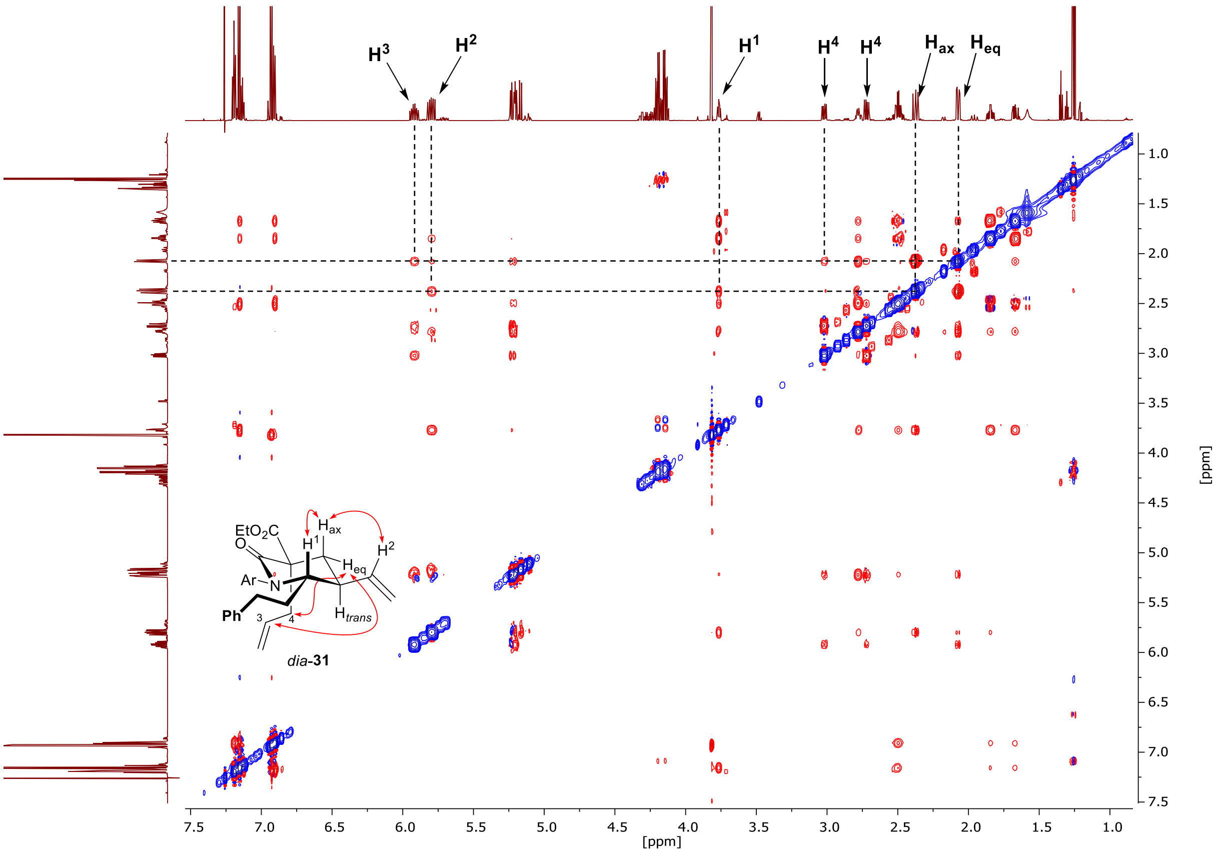

Figure 7: NOESY spectrum of *dia*-**31**.

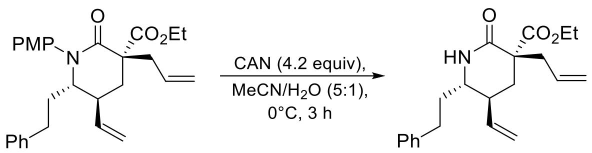

N-PMP lactam **31** (19.0 mg, 42.5 µmol, 1.0 equiv) was dissolved in a mixture of $MeCN/H_2O$ [5:1, 1.1 mL] and then cooled to 0°C. After 5 min, CAN (97.6 mg, 178 µmol, 4.2 equiv) was added in 4 portions. After complete addition, the reaction mixture was stirred for 3 h at 0°C and was subsequently quenched by the addition of sat aq $NaHCO_3$ (4 drops). The suspension was diluted with EtOAc (5 mL), dried over Na_2SO_4 and filtered over Celite. The solvent was removed under reduced pressure and the residue was

purified by reversed-phase chromatography. The amide **32** (8.0 mg, 23.4 µmol, 55%) was obtained as an oil. Following the same procedure from *dia*-**31** (17.0 mg, 38.0 µmol, 1.0 equiv) yielded *dia*-**32** (7.5 mg, 22.0 µmol, 58%) after 1.5 h reaction time.

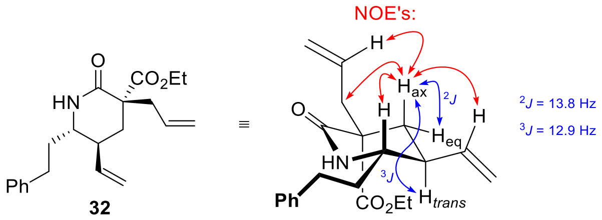

^{1}H-NMR (700 MHz, $CDCl_3$): δ = 1.29 (t, *J* = 7.1 Hz, 3H), 1.74 (dddd, *J* = 14.3, 10.4, 7.6, 5.3 Hz, 1H), 1.81 (dd, *J* = 13.8, 12.9 Hz, 1H, H_{ax}), 1.92 (dddd, *J* = 14.0, 10.9, 6.2, 2.9 Hz, 1H), 2.00 (dd, *J* = 13.8, 3.2 Hz, 1H, H_{eq}), 2.47 (dddd, *J* = 12.9, 10.8, 8.4, 3.2 Hz, 1H, H_{trans}), 2.54 (dddd, *J* = 13.7, 8.4, 0.9 Hz, 1H), 2.60 (ddd, *J* = 13.8, 10.4, 6.2 Hz, 1H), 2.74 (ddd, *J* = 13.8, 10.8, 5.3 Hz, 1H), 2.85 (dddd, *J* = 13.7, 6.1, 1.4 Hz, 1H), 3.18 (ddd, *J* = 10.4, 7.6, 2.9 Hz, 1H), 4.23 (m, 2H), 5.14 (m, 4H), 5.57 (ddd, *J* = 17.0, 10.3, 8.5 Hz, 1H), 5.73 (dddd, *J* = 17.0, 10.2, 8.5, 6.1 Hz, 1H), 6.01 (br s, 1H), 7.15 (m, 2H), 7.20 (m, 1H), 7.29 (m, 2H) ppm.

^{13}C-NMR (176 MHz, $CDCl_3$): δ = 14.3, 30.8, 35.7, 36.0, 40.0, 40.7, 53.1, 56.8, 61.7, 117.8, 119.6, 126.3, 128.4, 128.7, 133.2, 137.9, 140.9, 170.2, 172.4 ppm.

HRMS ($C_{21}H_{28}O_3N$, $[M + H]^+$, pos. ESI): *m/z*: calcd: 342.2064, found: 342.2069, Δ = 1.4 ppm.

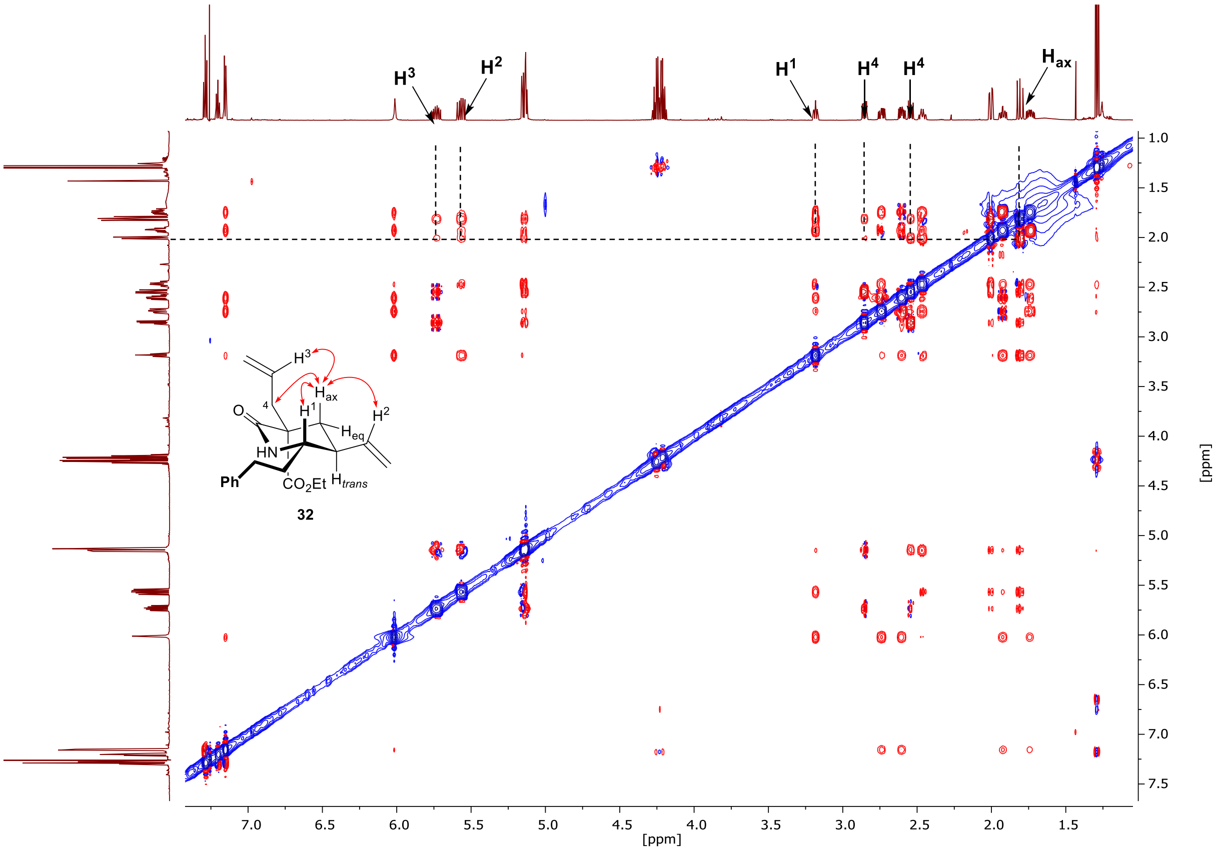

Figure 8: NOESY spectrum of **32**.

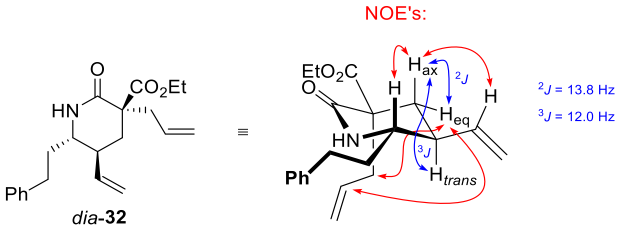

^{1}H-NMR (700 MHz, $CDCl_3$): δ = 1.26 (t, *J* = 7.1 Hz, 3H), 1.70 (dddd, *J* = 14.4, 10.2, 8.0, 5.4 Hz, 1H), 1.93 (dd, *J* = 13.7, 4.0 Hz, 1H, H_{eq}), 1.96 (m, 1H), 2.18 (dd, *J* = 13.8, 12.0 Hz, 1H, H_{ax}), 2.35 (m, 1H, H_{trans}), 2.60 (m, 2H), 2.75 (ddd, *J* = 13.7, 10.6, 5.3 Hz, 1H), 2.88 (dddd, *J* = 14.2, 6.7, 1.3 Hz, 1H), 3.28 (ddd, *J* = 10.6, 7.9, 2.9 Hz, 1H), 4.19 (q, *J* = 7.2 Hz, 2H), 5.13 (m, 4H), 5.58 (ddd, *J* = 16.7, 10.7, 8.6 Hz, 1H), 5.86 (dddd, *J* = 16.9, 10.1, 7.9, 6.6 Hz, 1H), 5.98 (s, 1H), 7.16 (m, 2H), 7.21 (m, 1H), 7.29 (m, 2H) ppm.

^{13}C-NMR (176 MHz, $CDCl_3$): δ = 14.1, 31.0, 35.1, 35.7, 39.8, 40.5, 53.3, 56.2, 61.7, 117.8, 118.8, 126.4, 128.4, 128.7, 133.9, 137.9, 140.7, 171.2, 171.9 ppm.

HRMS ($C_{21}H_{28}O_3N$, $[M + H]^+$, pos. ESI): *m/z*: calcd: 342.2064, found: 342.2070, Δ = 1.9 ppm.

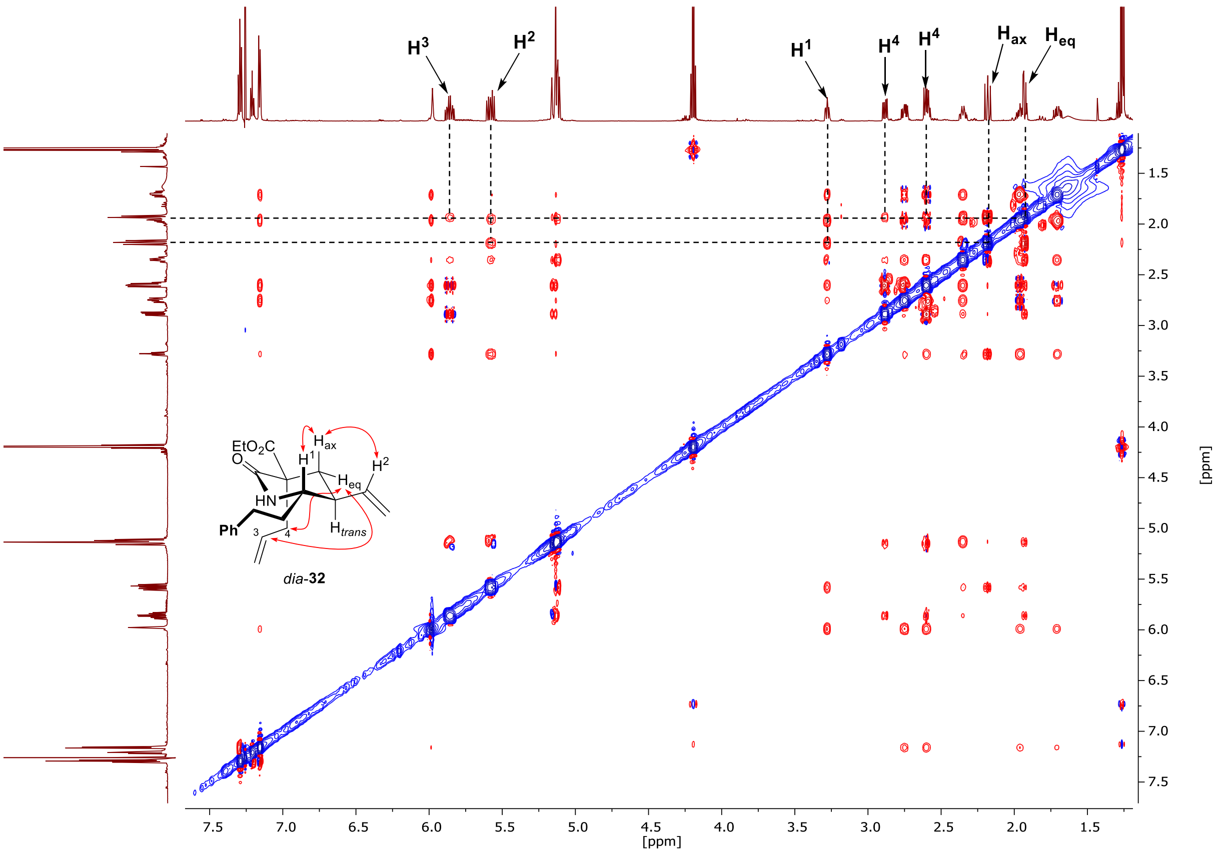

Figure 9: NOESY spectrum of *dia*-**32**.

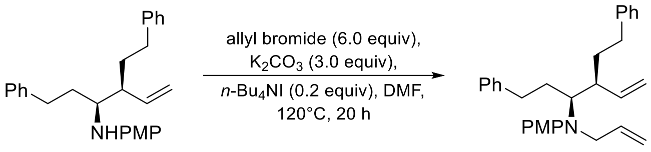

7 **33**, 76%

Anilin **7** (86.9 mg, 225 µmol, 1.0 equiv), *n*-Bu_4NI (98%, 16.9 mg, 45.0 µmol, 0.2 equiv) and K_2CO_3 (93.3 mg, 675 µmol, 3.0 equiv) were dissolved in dry DMF (1 mL). Allyl bromide (99%, 118 µL, 165 mg, 1.35 µmol, 6.0 equiv) was added and the reaction mixture was stirred at 120°C for 20 h. The solution was filtered over celite and concentrated in vacuo. The residue was purified by flash column chromatography over silica gel [*n*-pentane/Et_2O 49:1 → 29:1 v/v, R_f = 0.35 (*n*-pentane/Et_2O 19:1 v/v)]. The product (**33**, 73.1 mg, 172 µmol, 76%) was obtained as an oil.

^{1}H-NMR (500 MHz, $CDCl_3$): δ = 1.41 (m, 0.86H), 1.58 (m. 0.16H), 1.78 (m, 1H), 1.95 (m, 2H), 2.24 (m, 1H), 2.42 (m, 2H), 2.66 (m, 2H), 3.59 (m, 0.83H), 3.76 (m, 5.24H), 5.12 (m, 4H), 5.59 (m, 1H), 5.80 (m, 1H), 6.69 (m, 2H), 6.77 (m, 2H), 7.05 (m, 3H), 7.14 (m, 3H), 7.23 (m, 4H) ppm.

^{13}C-NMR (126 MHz, $CDCl_3$): δ = 33.2, 33.5, 33.6, 33.6, 33.7, 34.4, 46.8, 47.4, 48.8, 49.3, 55.8, 55.8, 63.1, 63.6, 114.4, 114.6, 115.4, 115.6, 116.1, 116.2, 116.5, 117.3, 125.7, 125.7, 125.8, 125.8, 128.3, 128.3, 128.4, 128.4, 128.5, 128.5, 128.6, 136.5, 136.7, 140.4, 140.6, 142.3, 142.4, 142.4, 142.5, 144.6, 151.2, 151.3 ppm.

HRMS ($C_{30}H_{36}ON$, $[M + H]^+$, pos. ESI): *m/z*: calcd: 426.2791, found: 426.2799, Δ = 1.8 ppm.

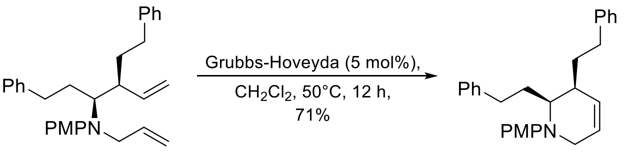

33 **S72**

The bisolefin **33** (73.1 mg, 172 µmol, 1.0 equiv) and Grubbs-Hoveyda catalyst (5.39 mg, 8.60 µmol, 5.0 mol%) were dissolved in dry, degassed CH_2Cl_2 (3.4 mL, 0.05 M). The mixture was stirred at 50°C for 15 h. Subsequently, the solvent was evaporated under reduced pressure and the residue was purified by flash column chromatography over silica gel [*n*-pentane/Et_2O 29:1 → 19:1 v/v, R_f = 0.18 (*n*-pentane/Et_2O 19:1 v/v)]. The product (**S72**, 48.6 mg, 122 µmol, 71%) was obtained as yellow oil.

^{1}H-NMR (500 MHz, $CDCl_3$): δ = 1.63 (m, 2H), 1.70 (dddd, *J* = 13.9, 7.0, 3.6 Hz, 1H), 1.92 (m, 1H), 2.45 (ddd, *J* = 13.8, 9.9, 7.1 Hz, 1H), 2.64 (m, 4H), 3.73 (m, 2H), 3.78 (s, 3H), 3.83 (m, 1H), 5.67 (dddd, *J* = 10.1, 3.4, 2.0 Hz, 1H), 5.80 (dddd, *J* = 10.1, 2.9 Hz, 1H), 6.77 (m, 2H), 6.82 (m, 2H), 7.08 (m, 2H), 7.23 (m, 8H) ppm.

^{13}**C-NMR** (126 MHz, $CDCl_3$): δ = 27.8, 33.3, 33.5, 34.1, 37.5, 43.9, 55.8, 57.2, 114.7, 116.8, 124.6, 125.7, 126.0, 128.3, 128.4, 128.5, 128.8, 142.1, 142.6, 145.5, 152.3 ppm.

HRMS ($C_{28}H_{32}ON$, $[M + H]^+$, pos. ESI): *m/z*: calcd: 398.2478, found: 398.2484, Δ = 1.4 ppm.

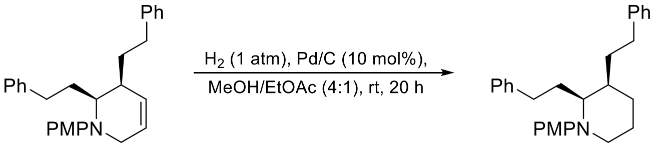

S72 **34**

The olefin **S72** (45.0 mg, 113 µmol, 1.0 equiv) and Pd/C (10% w/w, 12.0 mg, 11.3 µmol, 10 mol%) were dissolved in MeOH/EtOAc (4:1 v/v, 1.25 mL). Hydrogen was bubbled through the solution for 5 min using a needle connected to a baloon and the mixture was stirred at room temperature for 20 h. Afterwards the mixture was filtered over celite and concentrated under reduced pressure. The crude product was purified by flash column chromatography over silica gel [*n*-pentane/Et_2O 49:1 → 9:1 v/v, R_f = 0.16 (*n*-pentane/Et_2O 19:1 v/v)]. The product (**34**, 29.4 mg, 73.6 µmol, 65%) was obtained as a yellow oil.

^{1}**H-NMR** (500 MHz, $CDCl_3$): δ = 1.57 (m, 7H), 1.86 (m, 1H), 2.04 (m, 1H), 2.44 (ddd, *J* = 13.7, 10.1, 6.7 Hz, 1H), 2.59 (m, 3H), 3.12 (m, 1H), 3.40 (m, 1H), 3.73 (ddd, *J* = 9.0, 4.1 Hz, 1H), 3.78 (s, 3H), 6.79 (m, 4H), 7.09 (m, 2H), 7.18 (m, 4H), 7.26 (m, 4H) ppm.

^{13}**C-NMR** (126 MHz, $CDCl_3$): δ = 24.4, 26.2, 26.6, 33.6, 33.8, 35.7, 38.2, 42.3, 55.8, 60.0, 114.6, 117.4, 125.7, 125.8, 128.3, 128.4, 128.4, 128.5, 142.6, 142.8, 145.7, 152.0 ppm.

HRMS ($C_{28}H_{34}ON$, $[M + H]^+$, pos. ESI): *m/z*: calcd: 400.2635, found: 400.2633, Δ = –0.5 ppm.

7. Determination of Main Diastereoisomer

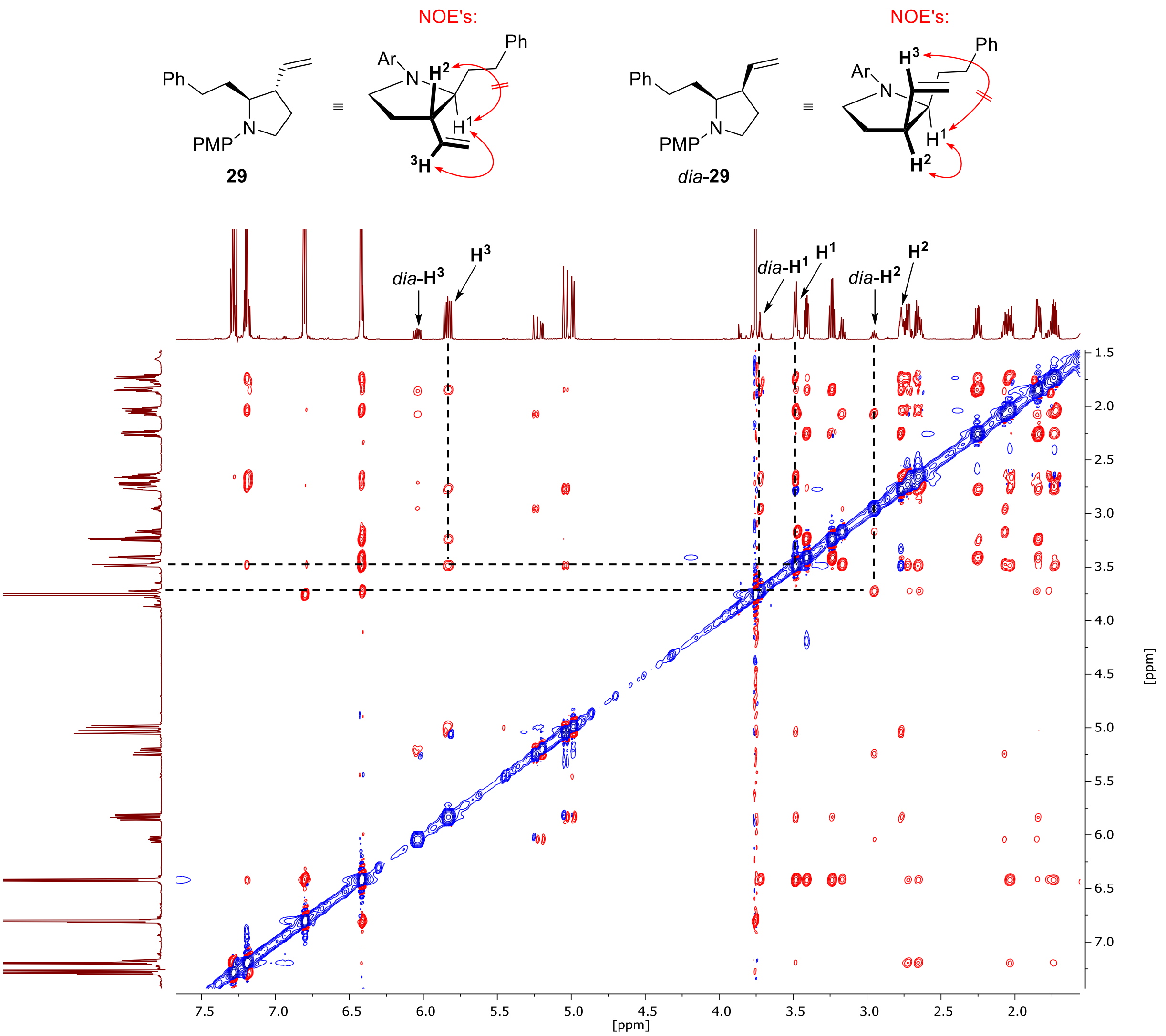

Figure 10: NOESY spectrum of 3.7:1 mixture of **29** and *dia*-**29**.

8. DFT Calculations

General Remarks

All DFT calculations have been carried out using Gaussian16.[34] All geometries were optimized using the UB3LYP[35] functional in combination with the def2SVP basis set[36] for all atoms except nickel, for which the SDD[37] pseudopotential was applied. In all cases the ultrafine integral grid was employed (int=grid=ultrafine). During the geometry optimization we considered the solvent THF by the use of the smd model[38] and the dispersion energies were considered by the use of the Grimme D3 correction together with Becke-Johnson damping (keyword EmpiricalDispersion=GD3BJ).[39] Frequency calculations were performed in order to obtain thermal corrections at 298 K. All transition states showed only one imaginary frequency during vibrational analysis. Furthermore, all transition states were confirmed by the analysis of the intrinsic reaction coordinates (IRC with the calcall keyword).

Comparison of Zimmerman-Traxler Transition States for *syn* and *anti* Product

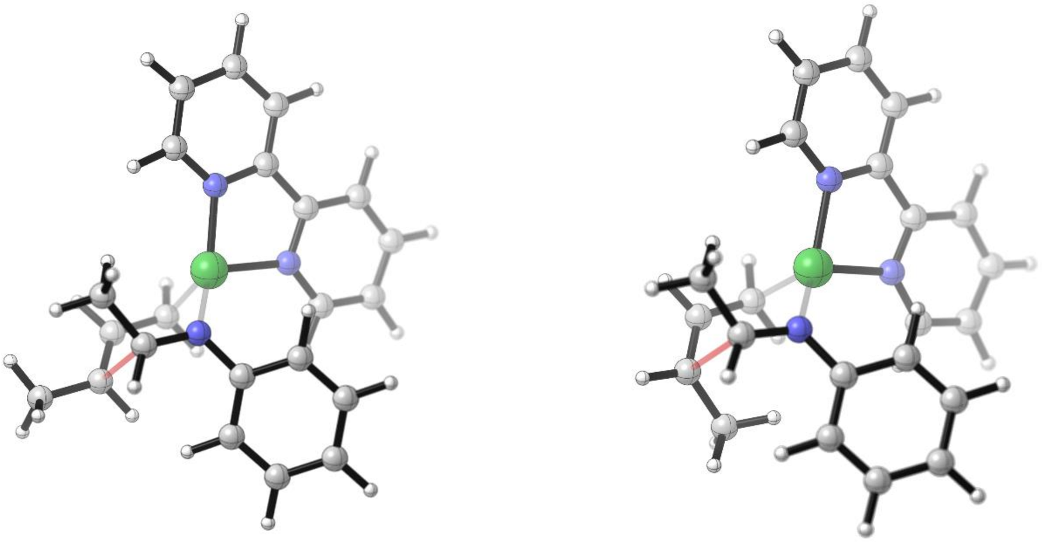

TS-1 0.00 kcal/mol

TS-2 2.43 kcal/mol

Figure 11: Relative ΔG of both Zimmerman-Traxler transition states [UB3LYP/def2SVP/SDD/D3/SMD(THF)]

TS-1

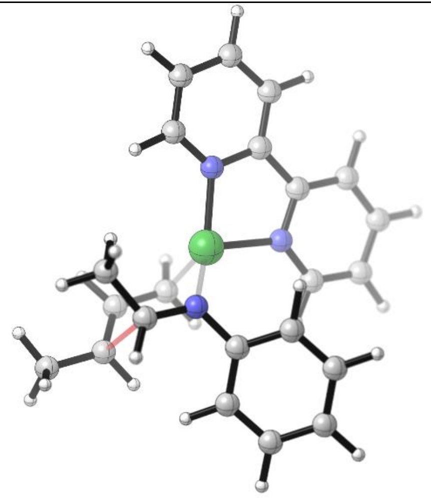

**E/hartree**	-1187.417771
**E+zvp/hartree**	-1187.010778
**G/hartree**	-1187.066390
**Imaginary frequency**	
-260.61 cm^{-1}	

C	4.0782810	-2.4501656	-1.1370506
C	2.7641330	-2.1758056	-0.8076246
C	3.2839020	0.0278264	-0.1996786
C	4.6364870	-0.1914706	-0.5194546
C	5.0434590	-1.4305446	-0.9912916
H	4.3509840	-3.4417576	-1.5028406
H	1.9874320	-2.9390386	-0.9059996
H	5.3611510	0.6141914	-0.3994316
H	6.0900850	-1.6116346	-1.2439896
C	2.7489820	1.2817404	0.3158504
C	3.5240500	2.4156574	0.6291974
C	2.9070920	3.5448044	1.1473874
H	4.6029830	2.4029784	0.4735034
C	0.8106330	2.3804634	1.0309174
C	1.5157860	3.5278354	1.3597194
H	3.4968300	4.4302834	1.3945144
H	-0.2695060	2.3200284	1.1772734
H	0.9913120	4.3914344	1.7719244
N	2.3526900	-0.9689726	-0.3512006
N	1.3979340	1.2875794	0.5092754
C	-0.3989040	-0.9421026	1.9774254
C	-1.9688770	-1.4649026	-0.9438176
C	-1.2560870	-2.0539186	1.7598624
H	-0.8848390	0.0154674	2.2105724
H	0.5028900	-1.1268116	2.5747394
C	-2.5600740	-2.0136066	1.2976154
H	-3.0331240	-1.4063766	-1.2077456
H	-0.7931470	-3.0498816	1.8305684
H	-3.0810420	-1.0529896	1.3598384
Ni	0.5023020	-0.4668126	0.1079254
C	-1.9117080	0.9013394	-0.9113316
C	-1.2039660	1.9876264	-1.4632536
C	-3.2216220	1.1269724	-0.4443326
C	-1.7799530	3.2546124	-1.5391996
H	-0.1903400	1.8147864	-1.8289176
C	-3.7919510	2.4009524	-0.5186036
H	-3.7944510	0.3087324	-0.0086116
C	-3.0784670	3.4725294	-1.0632166
H	-1.2088800	4.0796564	-1.9731666
H	-4.8067320	2.5549094	-0.1415546
H	-3.5290010	4.4664634	-1.1181776
N	-1.2648830	-0.3510806	-0.8594646
C	-1.2611260	-2.7411836	-1.2792616
H	-0.3130920	-2.8309326	-0.7254666
H	-1.0108530	-2.7465296	-2.3566786
H	-1.8789770	-3.6250496	-1.0722426
C	-3.4325740	-3.2313306	1.1704024
H	-4.0546980	-3.2091716	0.2570154
H	-2.8330540	-4.1561006	1.1397604
H	-4.1374690	-3.3306996	2.0183184

TS-2

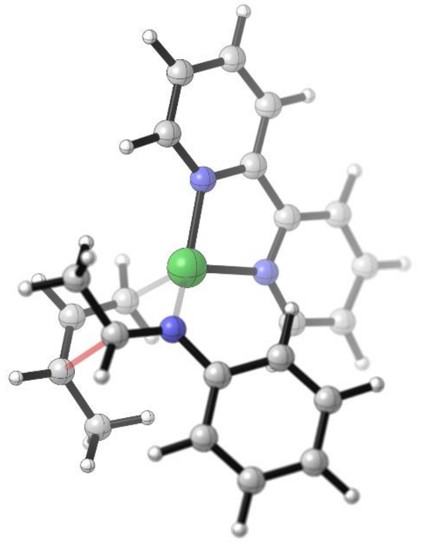

**E/hartree**	-1187.413963
**E+zvp/hartree**	-1187.007038
**G/hartree**	-1187.062511
**Imaginary frequency**	
-247.06 cm^{-1}	

C	4.3144892	-1.9264066	-1.4188219
C	2.9743112	-1.8476516	-1.0846709
C	3.2016602	0.3561394	-0.3239849
C	4.5730332	0.3366964	-0.6386719
C	5.1376402	-0.8041446	-1.1900609
H	4.7144072	-2.8451976	-1.8514489
H	2.3013762	-2.6952476	-1.2428459
H	5.1880682	1.2169214	-0.4490909
H	6.2000592	-0.8310966	-1.4404979
C	2.5043332	1.4920154	0.2688961
C	3.1209002	2.7009564	0.6453341
C	2.3612672	3.7044254	1.2286461
H	4.1896252	2.8434004	0.4846301
C	0.4419552	2.2724894	1.0488721
C	0.9869442	3.4855604	1.4400901
H	2.8270002	4.6468944	1.5247111
H	-0.6190548	2.0568274	1.1926911
H	0.3530232	4.2456584	1.8997541
N	2.4115392	-0.7392336	-0.5529189
N	1.1680022	1.2999634	0.4662071
C	0.1721212	-1.3493246	1.9725211
C	-1.8435808	-1.9586026	-0.7982949
C	-0.6564368	-2.4845296	1.7625881
H	-0.2695878	-0.4822656	2.4796381
H	1.2063172	-1.5469706	2.2819051
C	-2.0327288	-2.4857356	1.6193071
H	-2.9203358	-2.0835496	-0.9703859
H	-0.1406588	-3.4307146	1.5549321
Ni	0.5300092	-0.5518086	0.0244901
C	-2.1375578	0.3903634	-0.9937249
C	-1.5184468	1.5561774	-1.4855419
C	-3.5303888	0.4232654	-0.7779819
C	-2.2545018	2.7152534	-1.7326649
H	-0.4444288	1.5318114	-1.6744409
C	-4.2625488	1.5847784	-1.0285369
H	-4.0477298	-0.4554756	-0.3954009
C	-3.6336058	2.7411254	-1.5028849
H	-1.7432198	3.6042674	-2.1114969
H	-5.3402108	1.5858304	-0.8433359
H	-4.2121618	3.6480804	-1.6939859
N	-1.3214628	-0.7475546	-0.7885099
C	-0.9869748	-3.1301156	-1.1639739
H	0.0414802	-3.0171666	-0.7885989
H	-0.9253268	-3.1873246	-2.2678399
H	-1.4010128	-4.0804596	-0.7994049
C	-2.8689888	-1.3186926	2.0619781
H	-2.8590148	-1.1985306	3.1638071
H	-2.4914928	-0.3704696	1.6468081
H	-3.9218648	-1.4224016	1.7579631
H	-2.5362418	-3.4482326	1.4842471

Experiment:

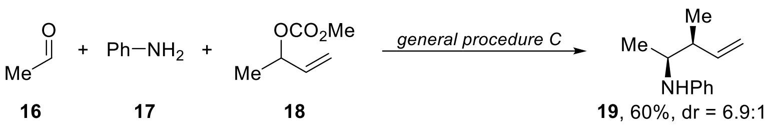

19 was synthesized according to general procedure C. Flash column chromatography: silica gel, *n*-pentane/Et_2O 49:1 → 29:1 v/v, R_f = 0.46 (*n*-pentane/Et_2O 19:1 v/v). The product (**19**, 20.9 mg, 119 µmol, 60%, dr = 6.9:1) was obtained as a yellow oil.

^{1}H-NMR (700 MHz, $CDCl_3$): δ = 1.05 (d, *J* = 6.8 Hz, 0.41H), 1.10 (d, *J* = 7.0 Hz, 2.84H), 1.11 (d, *J* = 6.5 Hz, 2.84H), 1.15 (d, *J* = 6.6 Hz, 0.42H), 2.46 (m, 1H), 3.46 (m, 1H), 3.57 (br s, 1H), 5.05 (ddd, *J* = 17.1, 2.0, 1.1 Hz, 0.91H), 5.09 (ddd, *J* = 10.2, 2.0, 0.8 Hz, 0.91H), 5.12 (m, 0.26H), 5.77 (ddd, *J* = 17.1, 10.2, 8.2 Hz, 0.87H), 5.85 (ddd, *J* = 17.2, 10.4, 7.2 Hz, 0.13H), 6.59 (m, 2H), 6.67 (m, 1H), 7.16 (m, 2H) ppm.

^{13}C-NMR (176 MHz, $CDCl_3$): δ = 14.9, 16.3, 17.1, 17.5, 41.9, 42.2, 52.3, 52.4, 113.2, 113.5, 115.2, 116.0, 117.0, 117.0, 129.4, 140.0, 141.1, 147.5, 147.7 ppm.

HRMS ($C_{12}H_{18}N$, $[M + H]^+$, pos. APCI): *m/z*: calcd: 176.1434, found: 176.1430, Δ = –2.2 ppm.

9. Literature

1 J. Zheng, C. Nopper, R. Bibi, A. Nikbakht, F. Bauer and B. Breit, *ACS Catal.*, 2022, **12**, 5949.

2 L. L. Anka-Lufford, M. R. Prinsell and D. J. Weix, *J. Org. Chem.*, 2012, **77**, 9989.

3 a) K. Takizawa, T. Sekino, S. Sato, T. Yoshino, M. Kojima and S. Matsunaga, *Angew. Chem. Int. Ed.*, 2019, **58**, 9199; b) A. Bongini, G. Cardillo, M. Orena, G. Porzi and S. Sandri, *J. Org. Chem.*, 1982, **47**, 4626;

4 T. Schlatzer, H. Schröder, M. Trobe, C. Lembacher-Fadum, S. Stangl, C. Schlögl, H. Weber and R. Breinbauer, *Adv. Synth. Catal.*, 2020, **362**, 331.

5 C. Fischer, C. Defieber, T. Suzuki and E. M. Carreira, *J. Am. Chem. Soc.*, 2004, **126**, 1628.

6 B. Plietker, A. Dieskau, K. Möws and A. Jatsch, *Angew. Chem. Int. Ed.*, 2008, **47**, 198.

7 Y. Zhou and B. Breit, *Chem. Eur. J.*, 2017, **23**, 18156.

8 D. C. Vrieze, G. S. Hoge, P. Z. Hoerter, J. T. van Haitsma and B. M. Samas, *Org. Lett.*, 2009, **11**, 3140.

9 M. Weiss, J. Holz and R. Peters, *Eur. J. Org. Chem.*, 2016, **2016**, 210.

10 K. J. Fraunhoffer, D. A. Bachovchin and M. C. White, *Org. Lett.*, 2005, **7**, 223.

11 C. Nopper, A. Veith, F. Himmelsbach, L. Söhner and B. Breit, *Chem. Eur. J.*, 2025, **31**, e202403495.

12 Y. Zhou, A. Nikbakht, F. Bauer and B. Breit, *Chem. Sci.*, 2019, **10**, 4805.

13 T. Bury, S. Kullmann and B. Breit, *Adv. Synth. Catal.*, 2023, **365**, 335.

14 S. Ganss and B. Breit, *Angew. Chem. Int. Ed.*, 2016, **55**, 9738.

15 K. Semba, M. Shinomiya, T. Fujihara, J. Terao and Y. Tsuji, *Chem. Eur. J.*, 2013, **19**, 7125.

16 a) L. J. Hilpert and B. Breit, *Angew. Chem. Int. Ed.*, 2019, **58**, 9939; b) B. Bolte, Y. Odabachian and F. Gagosz, *J. Am. Chem. Soc.*, 2010, **132**, 7294;

17 B. M. Trost, A. B. Pinkerton and M. Seidel, *J. Am. Chem. Soc.*, 2001, **123**, 12466.

18 A. Hawkins, P. Jakubec, A. Ironmonger and D. J. Dixon, *Tetrahedron Lett.*, 2013, **54**, 365.

19 T. Yoshimitsu, T. Makino and H. Nagaoka, *J. Org. Chem.*, 2004, **69**, 1993.

20 G. Qin, C. M. Yam, A. Kumar, J. M. Lopez-Romero, S. Li, T. Huynh, Y. Li, B. Yang, R. Contreras-Caceres and C. Cai, *RSC Adv.*, 2017, **7**, 14466.

21 G. Makado, T. Morimoto, Y. Sugimoto, K. Tsutsumi, N. Kagawa and K. Kakiuchi, *Adv. Synth. Catal.*, 2010, **352**, 299.

22 A. Musgrove, C. R. Bridges, G. M. Sammis and D. Bizzotto, *Langmuir*, 2013, **29**, 3347.

23 T. Sastraruji, S. G. Pyne and A. T. Ung, *Tetrahedron*, 2012, **68**, 598.

24 L. Miller, F. Bauer and B. Breit, *Chem. Eur. J.*, 2024, **30**, e202400188.

25 A. T. Straub, M. Otto, I. Usui and B. Breit, *Adv. Synth. Catal.*, 2013, **355**, 2071.

26 B. Breit and W. Seiche, *J. Am. Chem. Soc.*, 2003, **125**, 6608.

27 J. L. Schwarz, F. Schäfers, A. Tlahuext-Aca, L. Lückemeier and F. Glorius, *J. Am. Chem. Soc.*, 2018, **140**, 12705.

28 X. Jiang, H. Jiang, Q. Yang, Y. Cheng, L.-Q. Lu, J. A. Tunge and W.-J. Xiao, *J. Am. Chem. Soc.*, 2022, **144**, 8347.

29 Y. Yasu, T. Koike and M. Akita, *Adv. Synth. Catal.*, 2012, **354**, 3414.

30 a) D. Ghorai, À. Cristòfol and A. W. Kleij, *Eur. J. Inorg. Chem.*, 2022, **2022**; b) F. Calogero, S. Potenti, E. Bassan, A. Fermi, A. Gualandi, J. Monaldi, B. Dereli, B. Maity, L. Cavallo, P. Ceroni and P. G. Cozzi, *Angew. Chem. Int. Ed.*, 2022, **61**, e202114981; c) Q. Zeng, F. Gao, J. Benet-Buchholz and A. W. Kleij, *ACS Catal.*, 2023, **13**, 7514; d) Z. Zhang, Z. Han, J. Li, H.-S. Hu, J. Li and C. Xi, *ACS Catal.*, 2024, **14**, 12392;

31 a) J. A. Leitch, T. Rogova, F. Duarte and D. J. Dixon, *Angew. Chem. Int. Ed.*, 2020, **59**, 4121; b) J. Hu, J. Wang, T. H. Nguyen and N. Zheng, *Beilstein J. Org. Chem.*, 2013, **9**, 1977;

32 D. K. Root and W. H. Smith, *J. Electrochem. Soc.*, 1982, **129**, 1231.

33 J. I. Day, K. Teegardin, J. Weaver and J. Chan, *Org. Process Res. Dev.*, 2016, **20**, 1156.

34 M. J. Frisch, G. W. Trucks, H. B. Schlegel, G. E. Scuseria, M. A. Robb, J. R. Cheeseman, G. Scalmani, V. Barone, G. A. Petersson, H. Nakatsuji, X. Li, M. Caricato, A. V. Marenich, J. Bloino, B. G. Janesko, R. Gomperts, B. Mennucci, H. P. Hratchian, J. V. Ortiz, A. F. Izmaylov, J. L. Sonnenberg, D. Williams-Young, F. Ding, F. Lipparini, F. Egidi, J. Goings, B. Peng, A. Petrone, T. Henderson, D. Ranasinghe, V. G. Zakrzewski, J. Gao, N. Rega, G. Zheng, W. Liang, M. Hada, M. Ehara, K. Toyota, R. Fukuda, J. Hasegawa, M. Ishida, T. Nakajima, Y. Honda, O. Kitao, H. Nakai, T. Vreven, K. Throssell, J. A. Montgomery, Jr., J. E. Peralta, F. Ogliaro, M. J. Bearpark, J. J. Heyd, E. N. Brothers, K. N. Kudin, V. N. Staroverov, T. A. Keith, R. Kobayashi, J. Normand, K. Raghavachari, A. P. Rendell, J. C. Burant, S. S. Iyengar, J. Tomasi, M. Cossi, J. M. Millam, M. Klene, C. Adamo, R. Cammi, J. W. Ochterski, R. L. Martin, K. Morokuma, O. Farkas, J. B. Foresman, and D. J. Fox, Gaussian 16, Gaussian, Inc., Wallingford CT, 2016.

35 a) A. D. Becke, *J. Chem. Phys.*, 1993, **98**, 5648; b) Lee, Yang and Parr, *Phys. Rev. B*, 1988, **37**, 785;

36 F. Weigend and R. Ahlrichs, *Phys. Chem. Chem. Phys.*, 2005, **7**, 3297.

37 M. Dolg, U. Wedig, H. Stoll and H. Preuss, *J. Chem. Phys.*, 1987, **86**, 866.

38 A. V. Marenich, C. J. Cramer and D. G. Truhlar, *J. Phys. Chem. B*, 2009, **113**, 6378.

39 a) S. Grimme, J. Antony, S. Ehrlich and H. Krieg, *J. Chem. Phys.*, 2010, **132**, 154104; b) S. Grimme, S. Ehrlich and L. Goerigk, *J. Comput. Chem.*, 2011, **32**, 1456;

10. NMR-Spectra

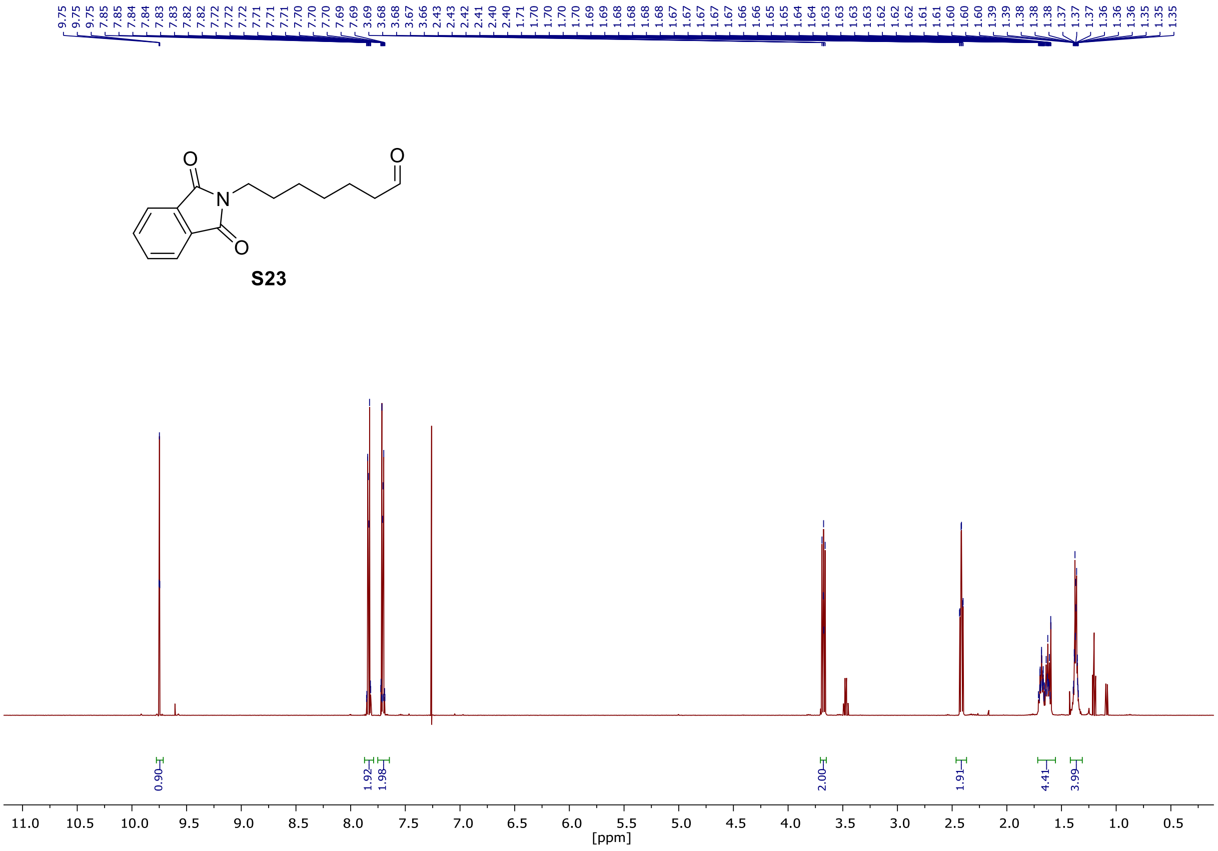

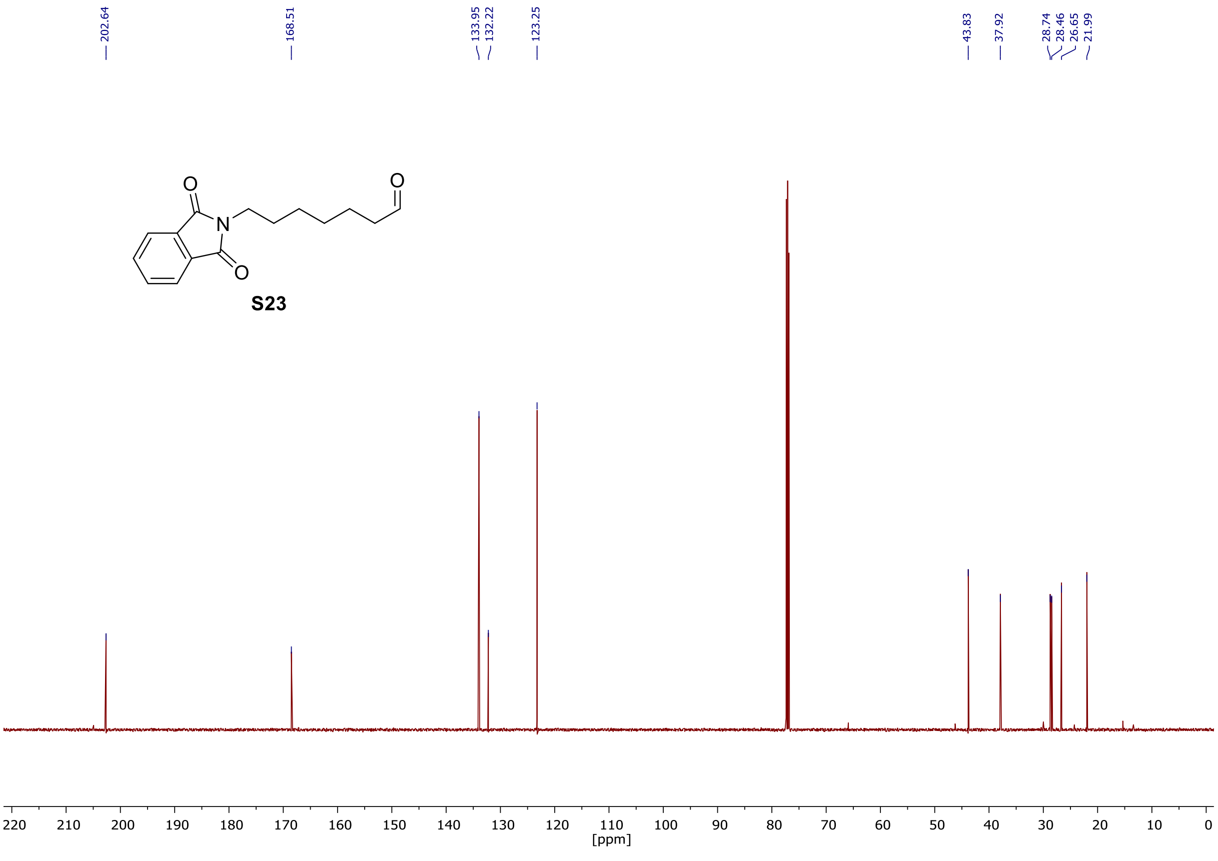

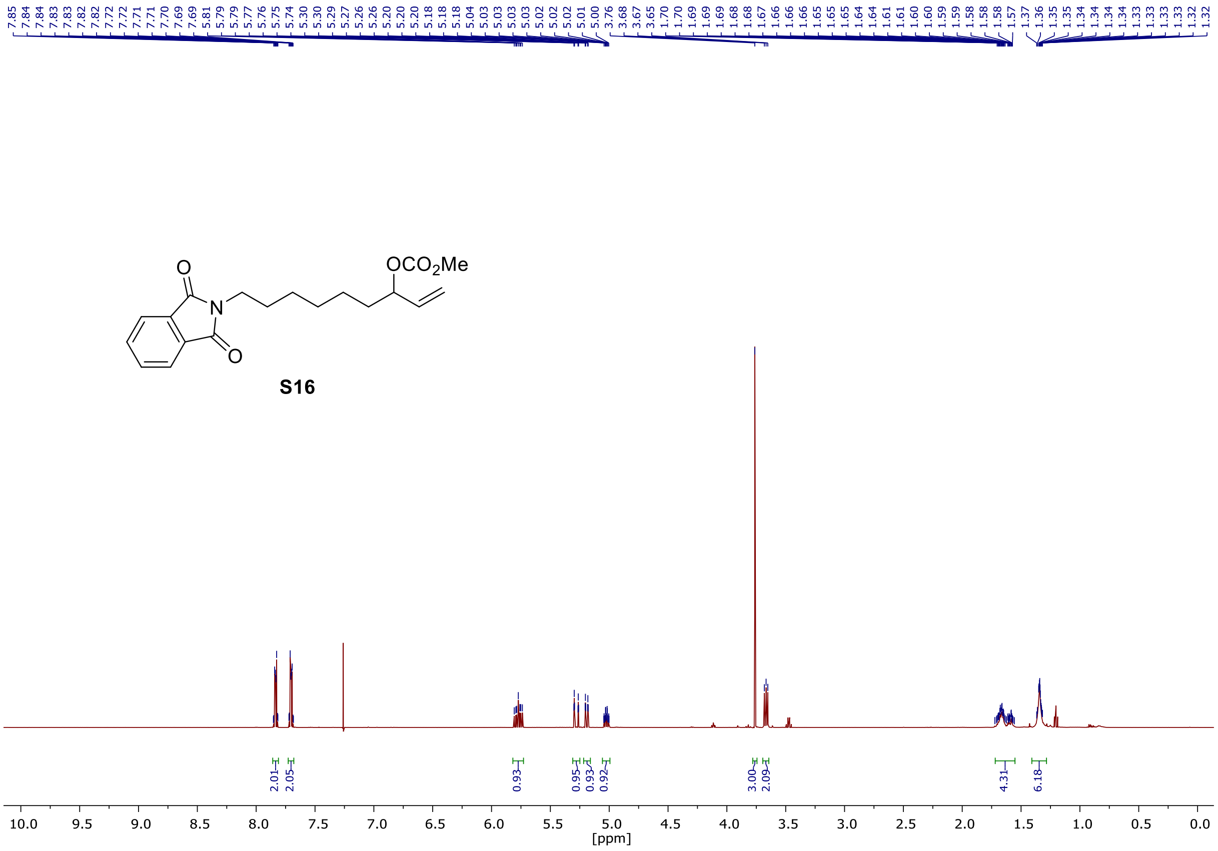
OCO2Me
S16
[ppm]

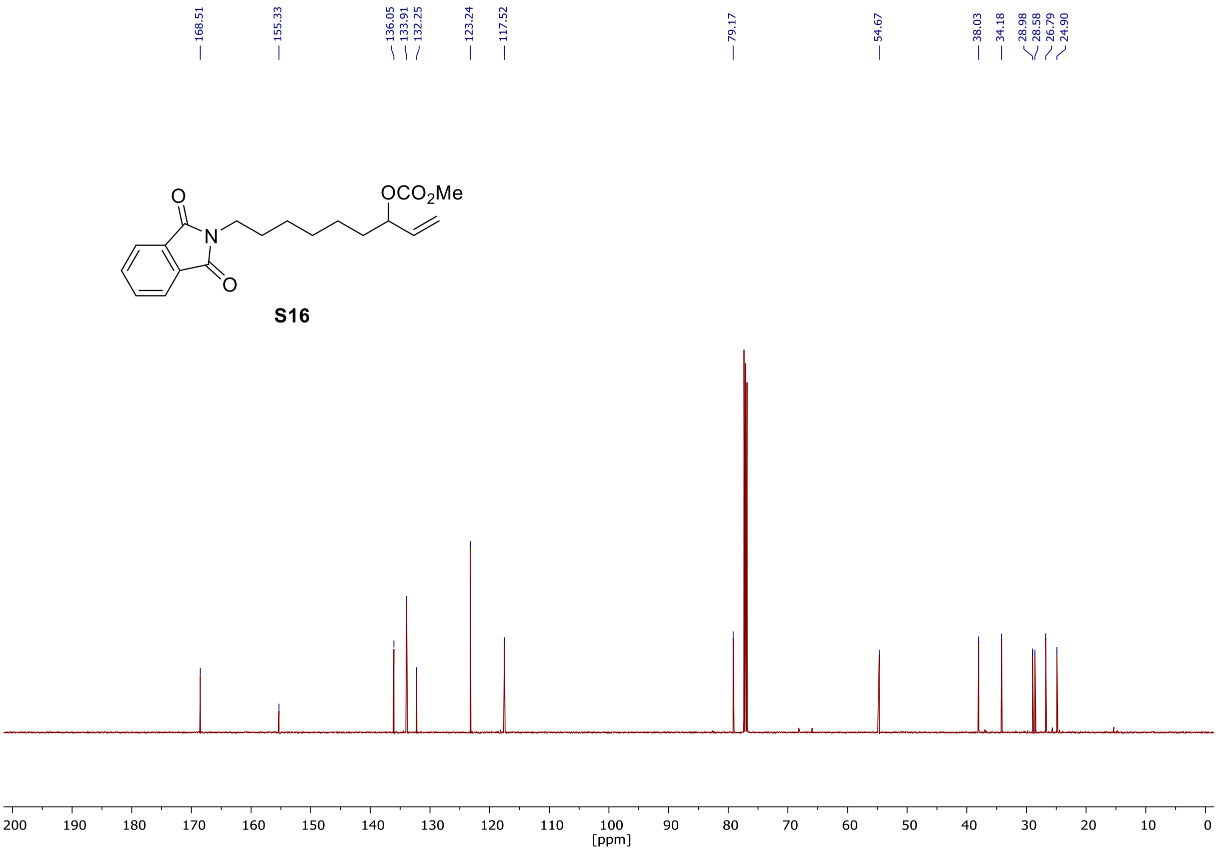
168.51
155.33
136.05
133.91
132.25
123.24
117.52
79.17
54.67
38.03
34.18
28.98
28.58
26.79
24.90
OCO2Me
S16
[ppm]

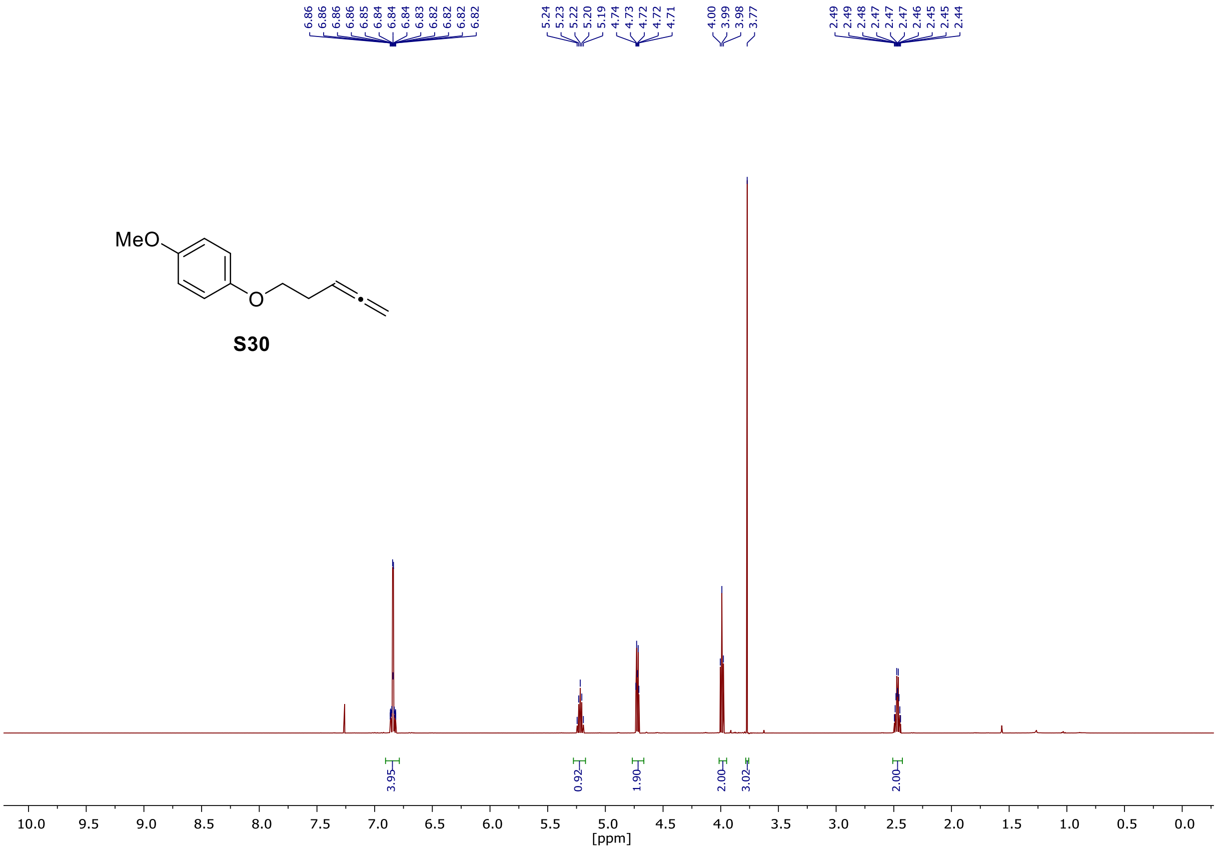
6.86 6.86 6.86 6.86 6.85 6.84 6.84 6.84 6.83 6.82 6.82 6.82 6.82
5.24 5.23 5.22 5.20 5.19 4.74 4.73 4.72 4.72 4.71
4.00 3.99 3.98 3.77
2.49 2.49 2.48 2.47 2.47 2.47 2.46 2.45 2.45 2.44
MeO
O
S30
3.95 0.92 1.90 2.00 3.02 2.00
10.0 9.5 9.0 8.5 8.0 7.5 7.0 6.5 6.0 5.5 5.0 4.5 4.0 3.5 3.0 2.5 2.0 1.5 1.0 0.5 0.0
[ppm]

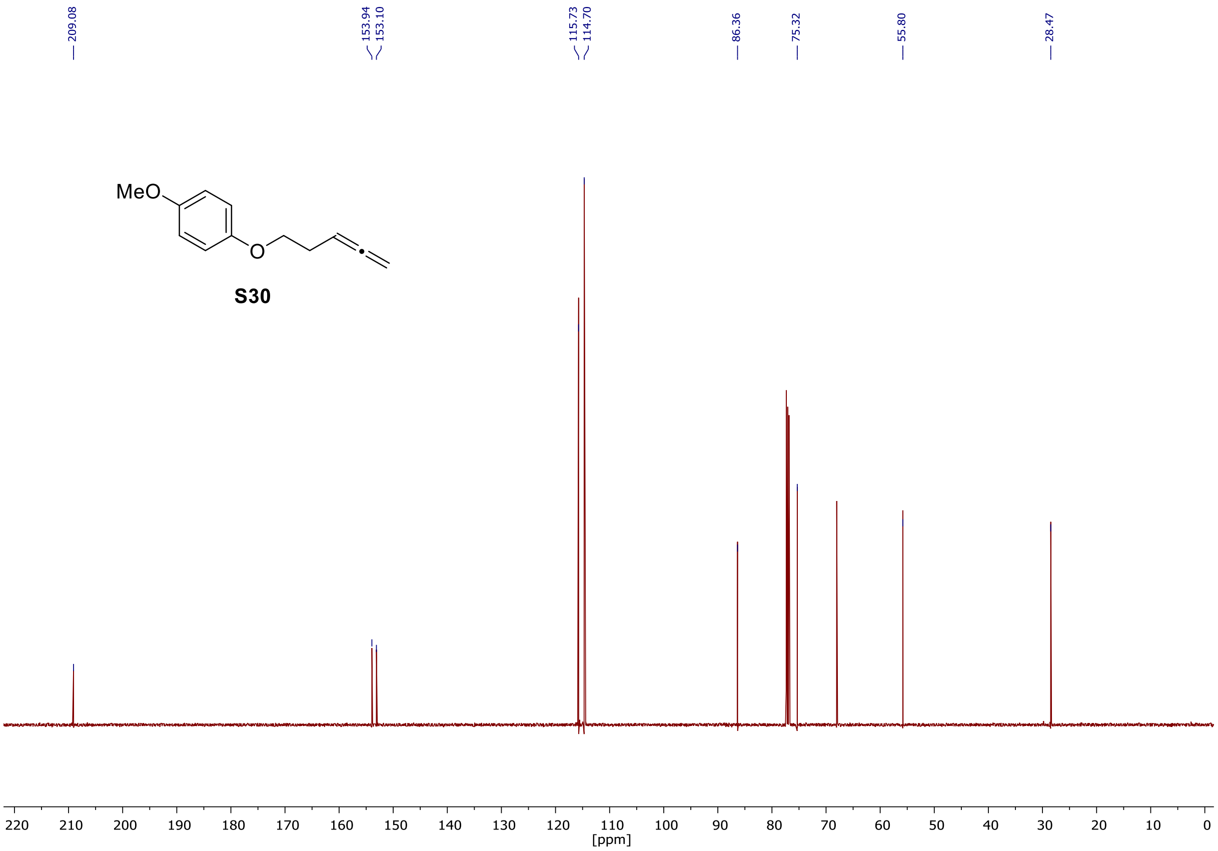
209.08
153.94 153.10
115.73 114.70
86.36
75.32
55.80
28.47
MeO
O
S30
220 210 200 190 180 170 160 150 140 130 120 110 100 90 80 70 60 50 40 30 20 10 0
[ppm]

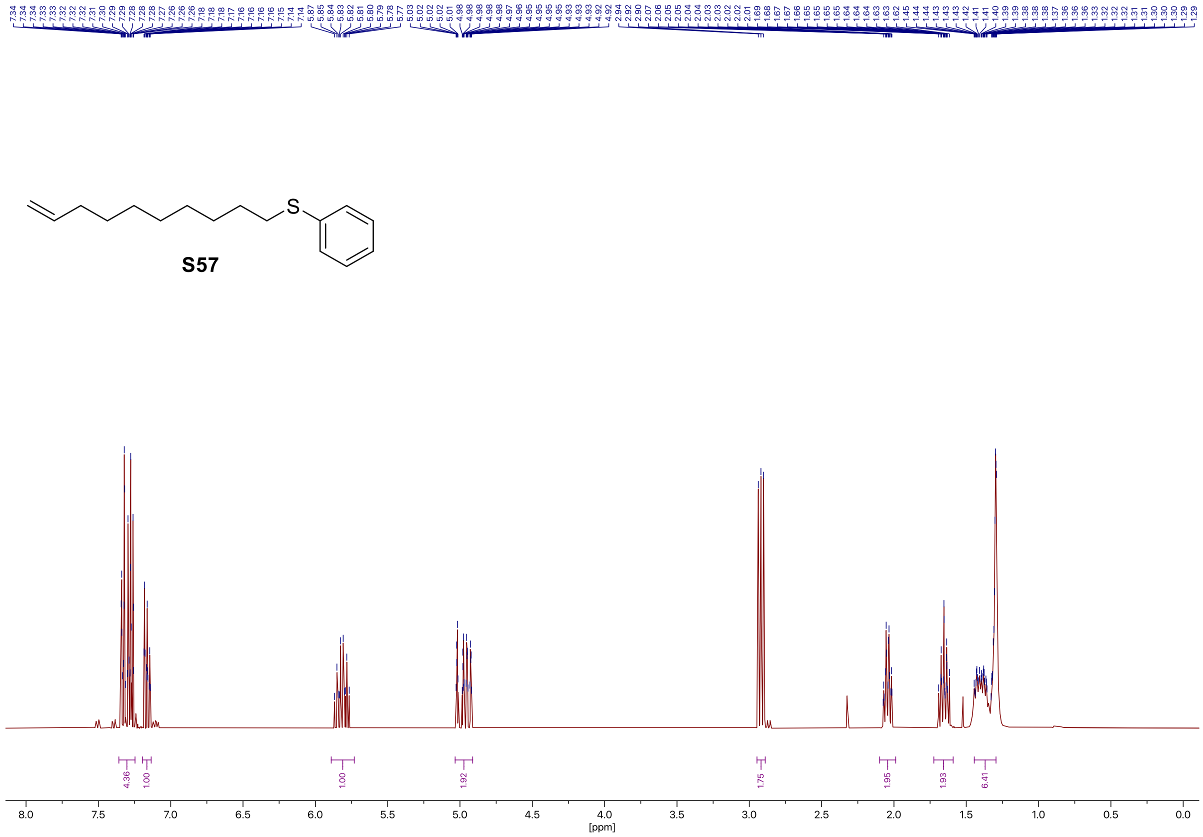
7.34 7.34 7.33 7.32 7.32 7.32 7.31 7.30 7.29 7.29 7.28 7.28 7.28 7.27 7.26 7.26 7.26 7.18 7.18 7.18 7.17 7.16 7.16 7.16 7.16 7.15 7.14 7.14
5.87 5.85 5.84 5.83 5.82 5.81 5.80 5.79 5.78 5.77
5.03 5.02 5.02 5.01 4.98 4.98 4.98 4.98 4.98 4.97 4.96 4.95 4.95 4.95 4.93 4.93 4.93 4.92 4.92
2.94 2.92 2.90 2.07 2.06 2.05 2.05 2.04 2.04 2.03 2.03 2.02 2.02 2.01 1.69 1.68 1.67 1.67 1.66 1.65 1.65 1.65 1.65 1.64 1.64 1.63 1.63 1.62 1.45 1.44 1.44 1.43 1.43 1.43 1.42 1.41 1.41 1.40 1.39 1.39 1.38 1.38 1.38 1.37 1.36 1.36 1.33 1.32 1.32 1.31 1.31 1.30 1.30 1.29 1.29
S
S57
4.36
1.00
1.00
1.92
1.75
1.95
1.93
6.41
8.0 7.5 7.0 6.5 6.0 5.5 5.0 4.5 4.0 3.5 3.0 2.5 2.0 1.5 1.0 0.5 0.0
[ppm]

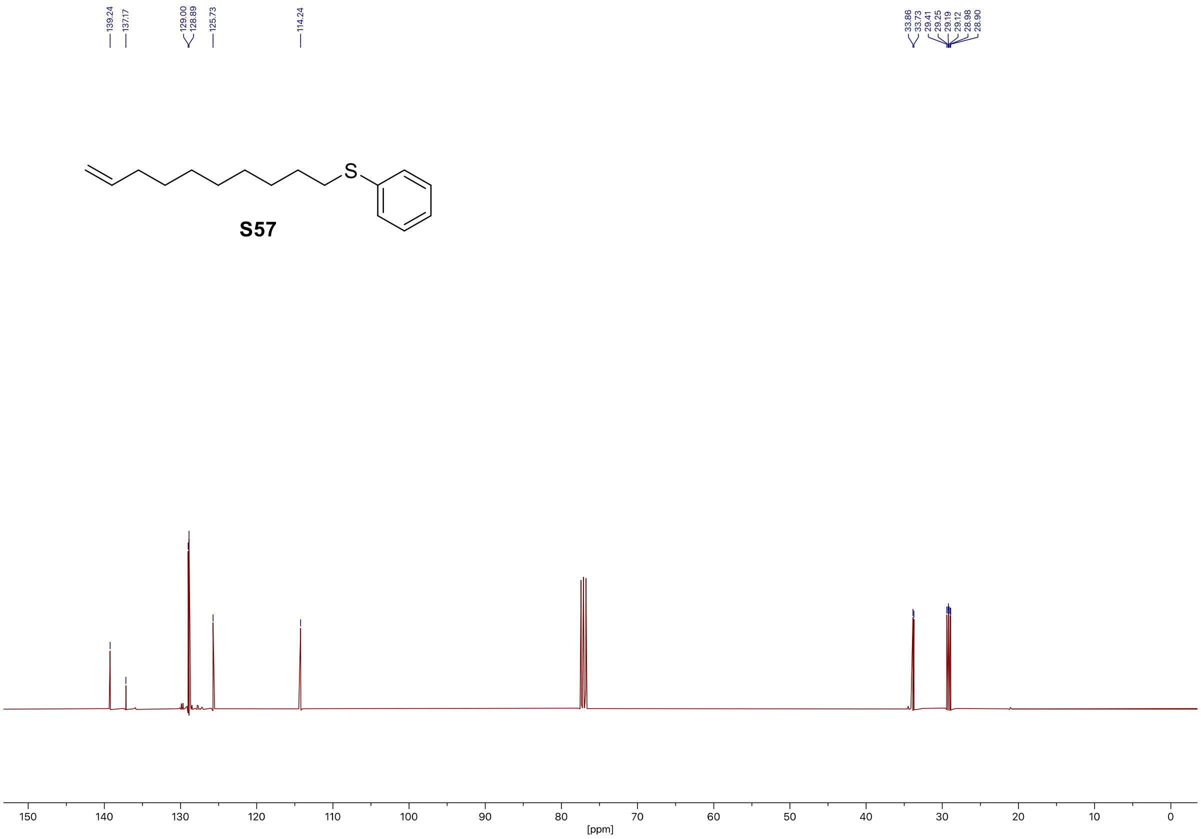
139.24
137.17
129.00
128.89
125.73
114.24
33.86
33.73
29.41
29.25
29.19
29.12
28.98
28.90
S
S57
150 140 130 120 110 100 90 80 70 60 50 40 30 20 10 0
[ppm]

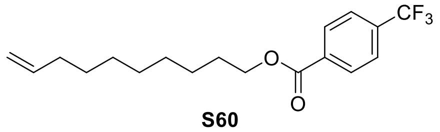

S60

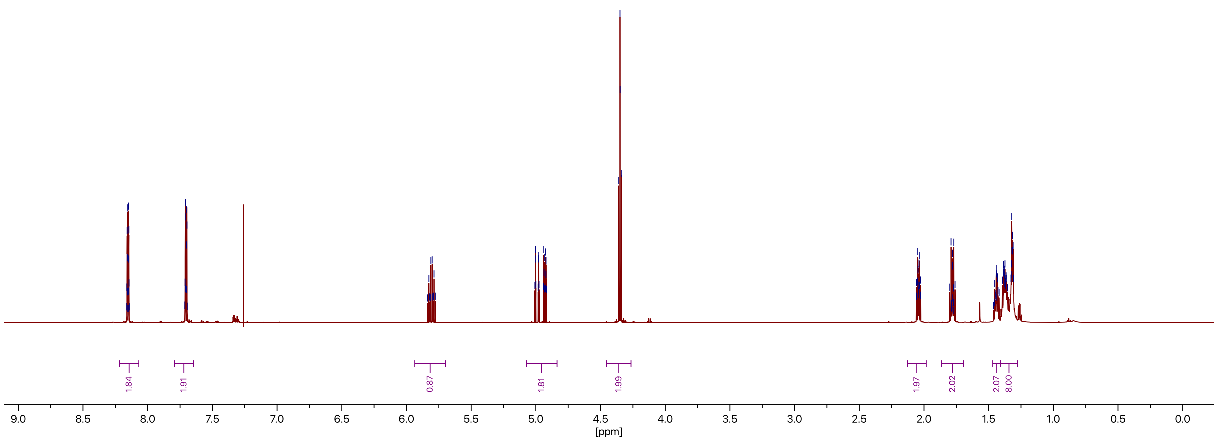
1.84
1.91
0.87
1.81
1.99
1.97
2.02
2.07
8.00
[ppm]

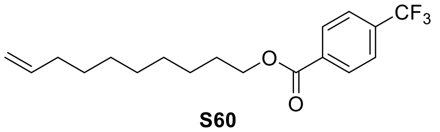

S60

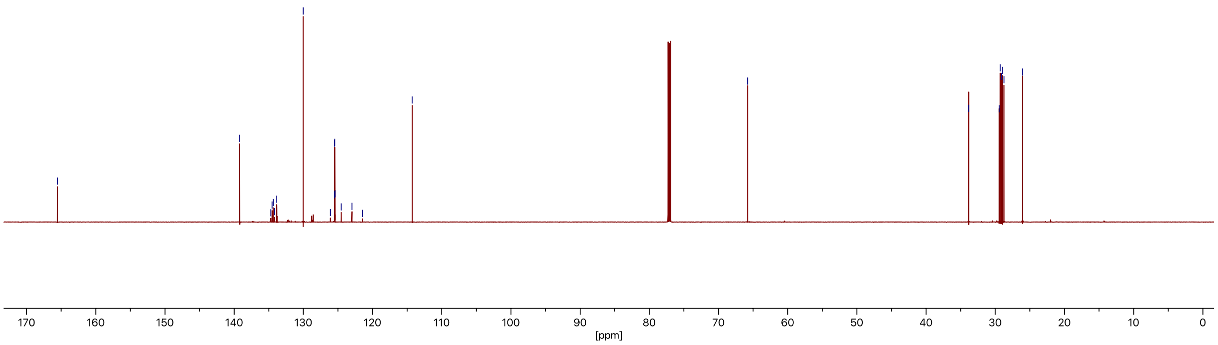
165.53
139.20
134.71
134.53
134.34
134.16
133.85
130.03
126.08
125.49
125.47
125.45
125.43
124.53
122.98
121.44
114.27
65.79
33.85
29.43
29.28
29.11
28.97
28.73
26.07
[ppm]

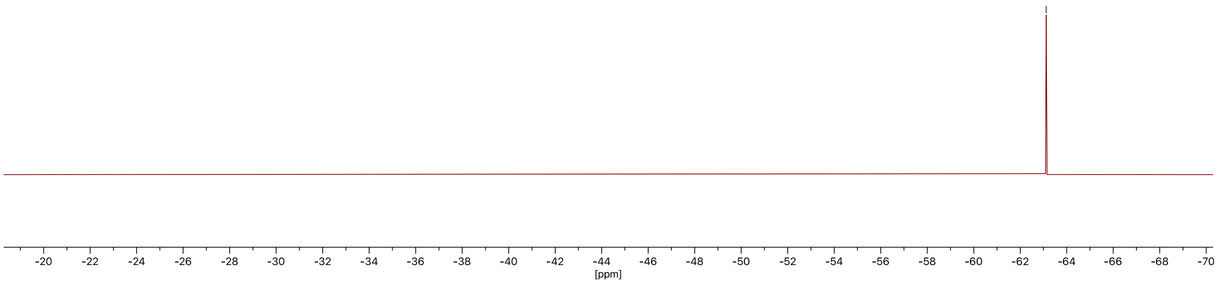

-63.12
-20
-22
-24
-26
-28
-30
-32
-34
-36
-38
-40
-42
-44
-46
-48
-50
-52
-54
-56
-58
-60
-62
-64
-66
-68
-70
[ppm]

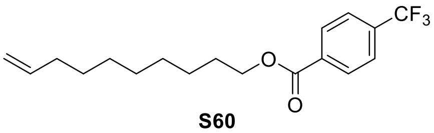

CF$_3$
O
O

S60

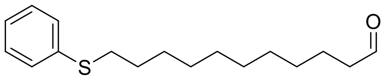

S
O

S51

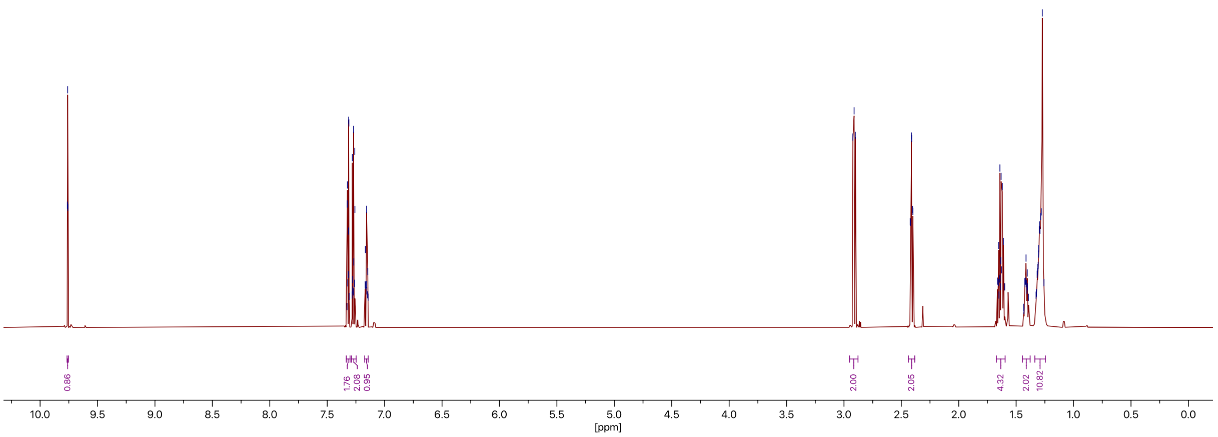

10.0
9.5
9.0
8.5
8.0
7.5
7.0
6.5
6.0
5.5
5.0
4.5
4.0
3.5
3.0
2.5
2.0
1.5
1.0
0.5
0.0
[ppm]

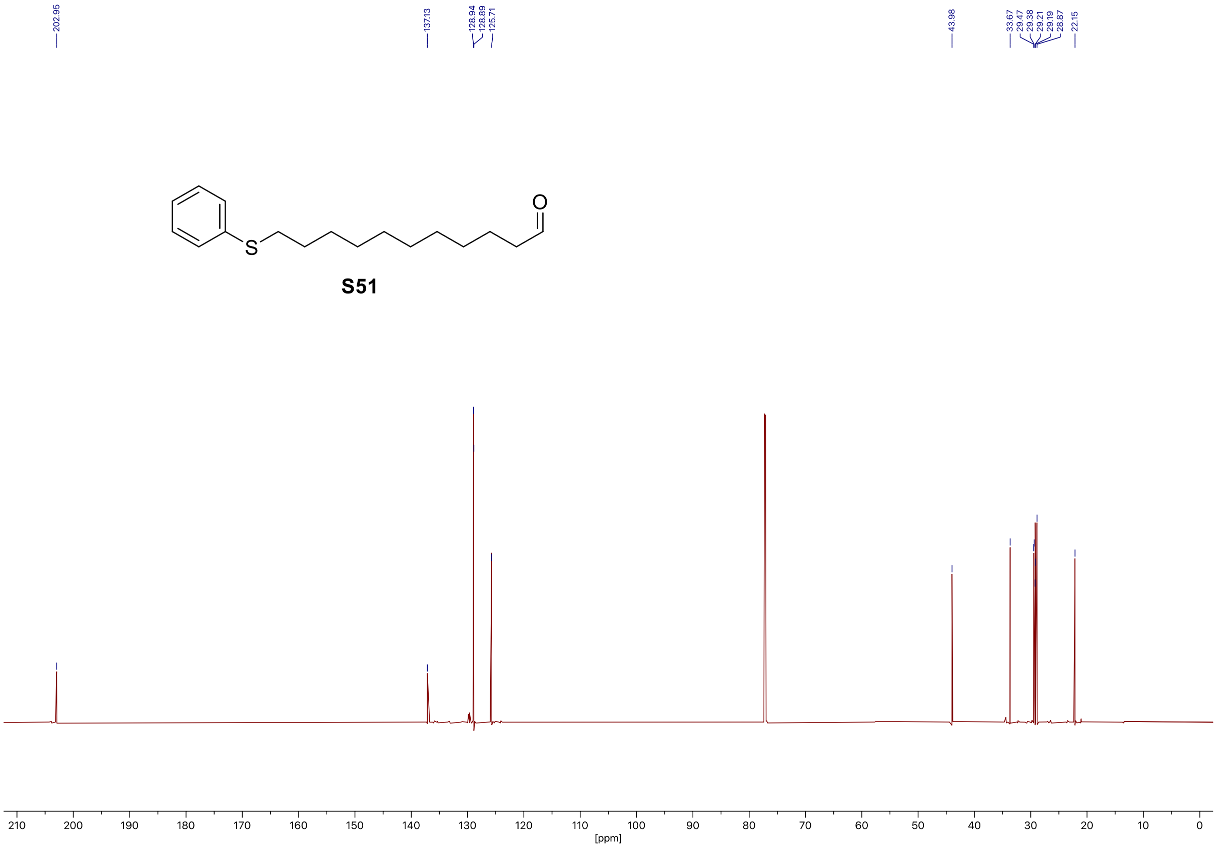
202.95
137.13
128.94
128.89
125.71
43.98
33.67
29.47
29.38
29.21
29.19
28.67
22.15
O
S
[ppm]

S51

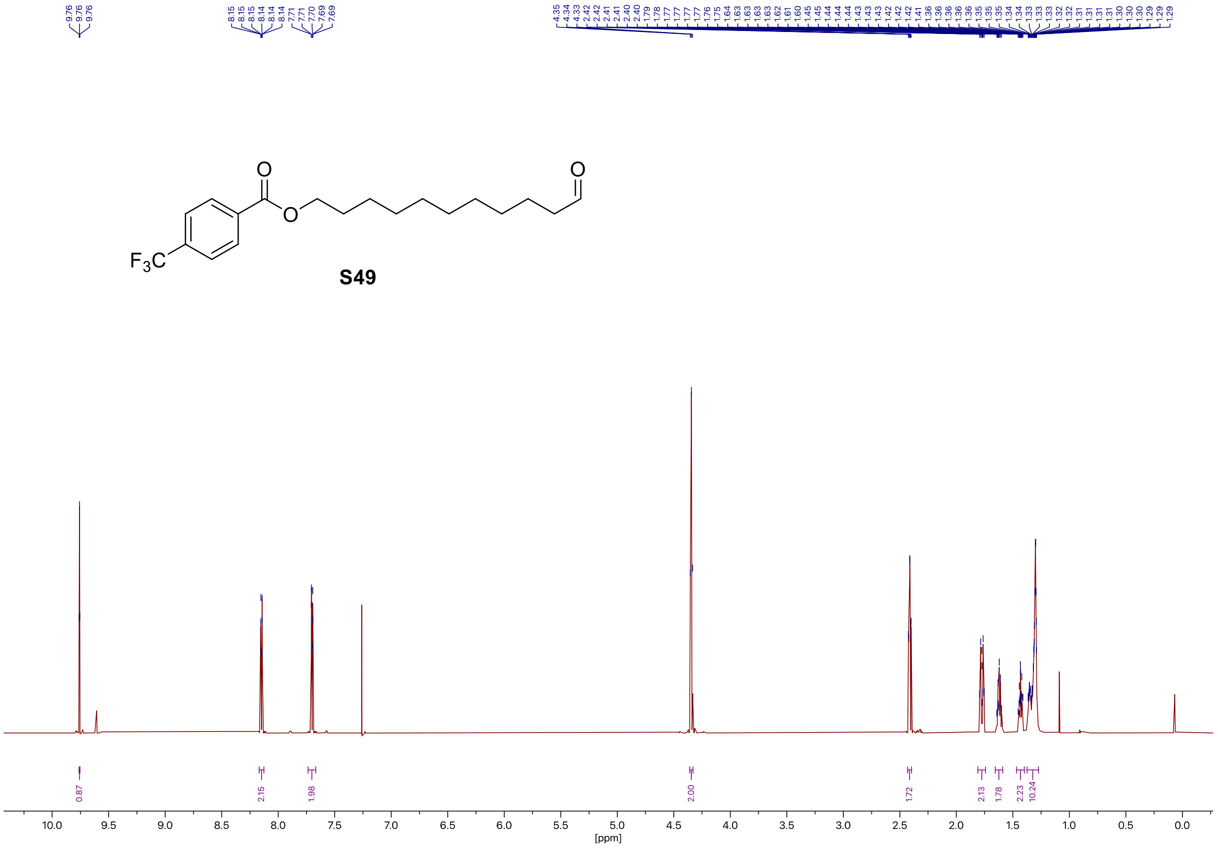
9.76
9.76
9.76
8.15
8.15
8.15
8.14
8.14
8.14
7.71
7.71
7.70
7.69
7.69
O
O
O
F3C
0.87
2.15
1.98
2.00
1.72
2.13
1.78
2.23
10.24
[ppm]

S49

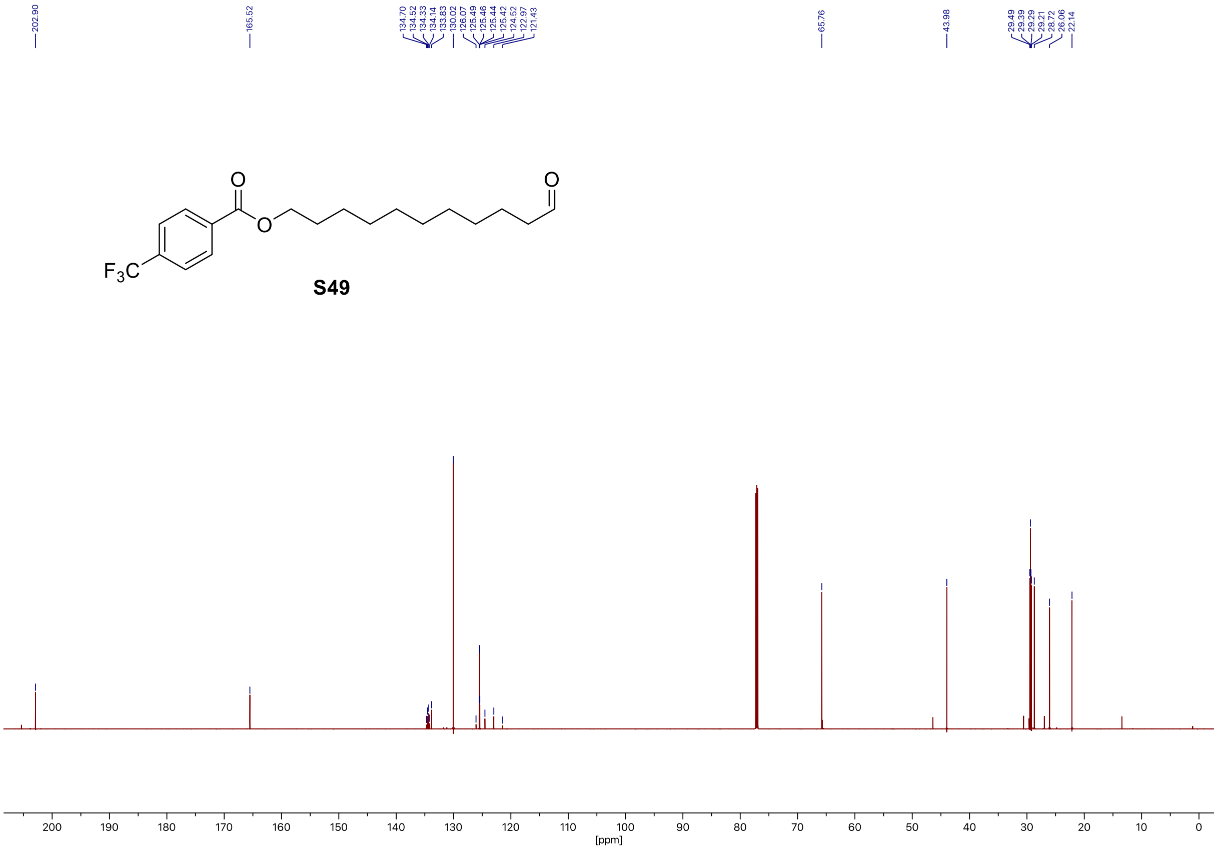
202.90
165.52
134.70
134.52
134.33
134.14
133.83
130.02
126.07
125.49
125.46
125.44
125.42
124.52
122.97
121.43
65.76
43.98
29.49
29.39
29.29
29.21
28.72
26.06
22.14
O
O
O
O
F3C
S49
[ppm]

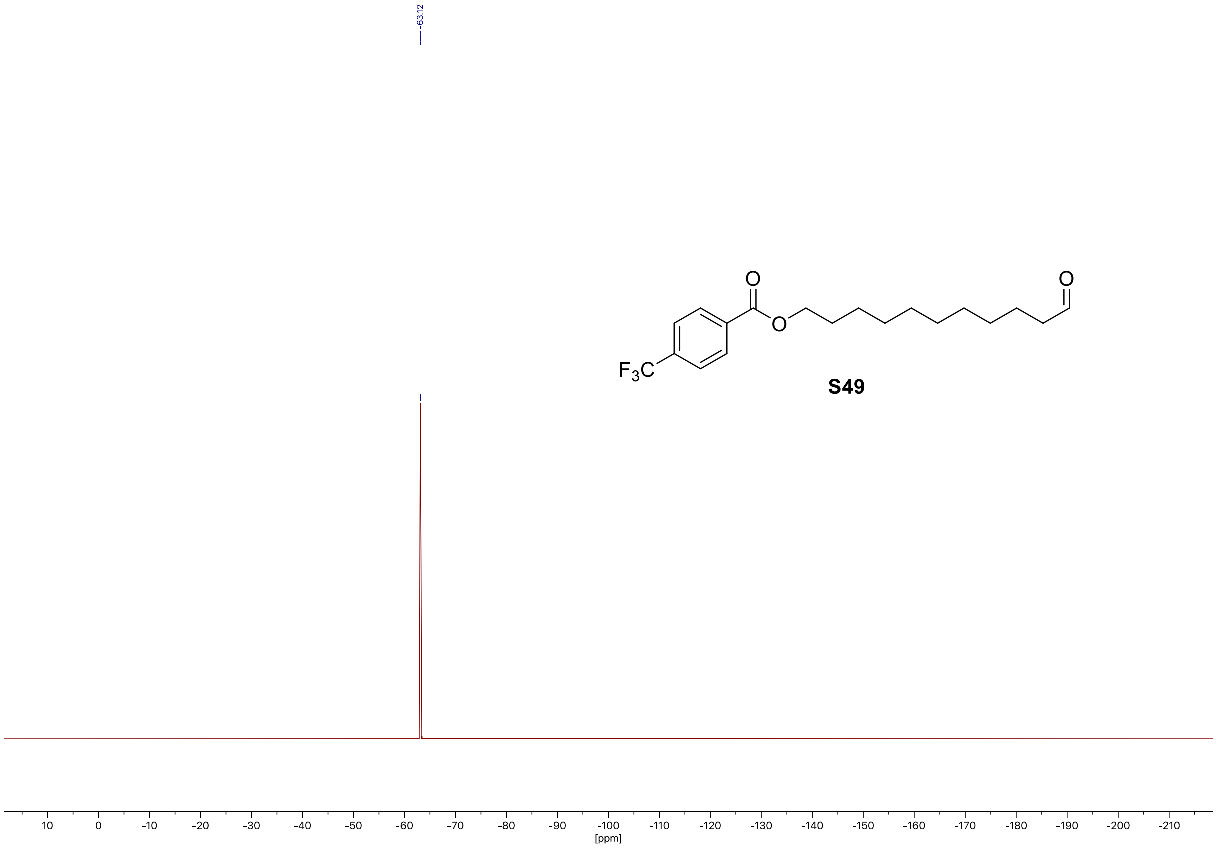
-63.12
O
O
O
O
F3C
S49
[ppm]

Homoallylic amines

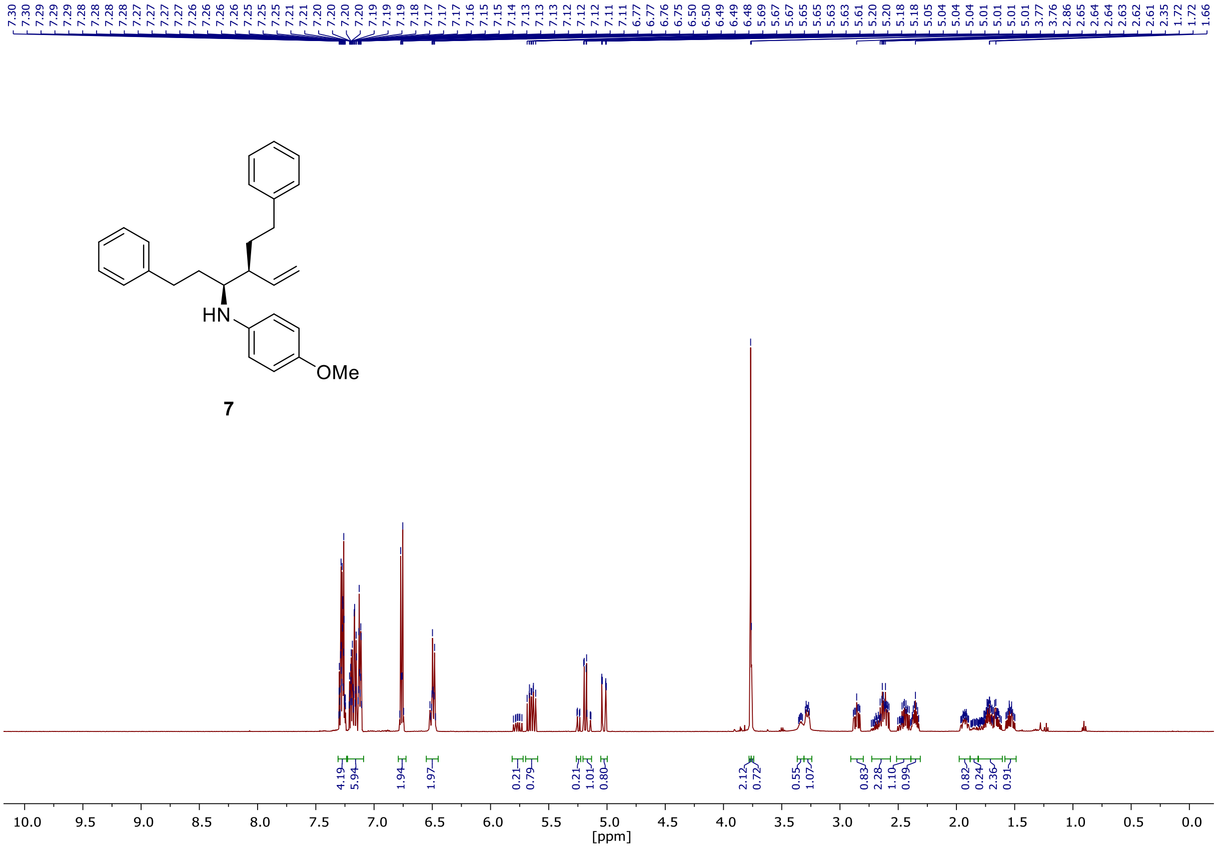

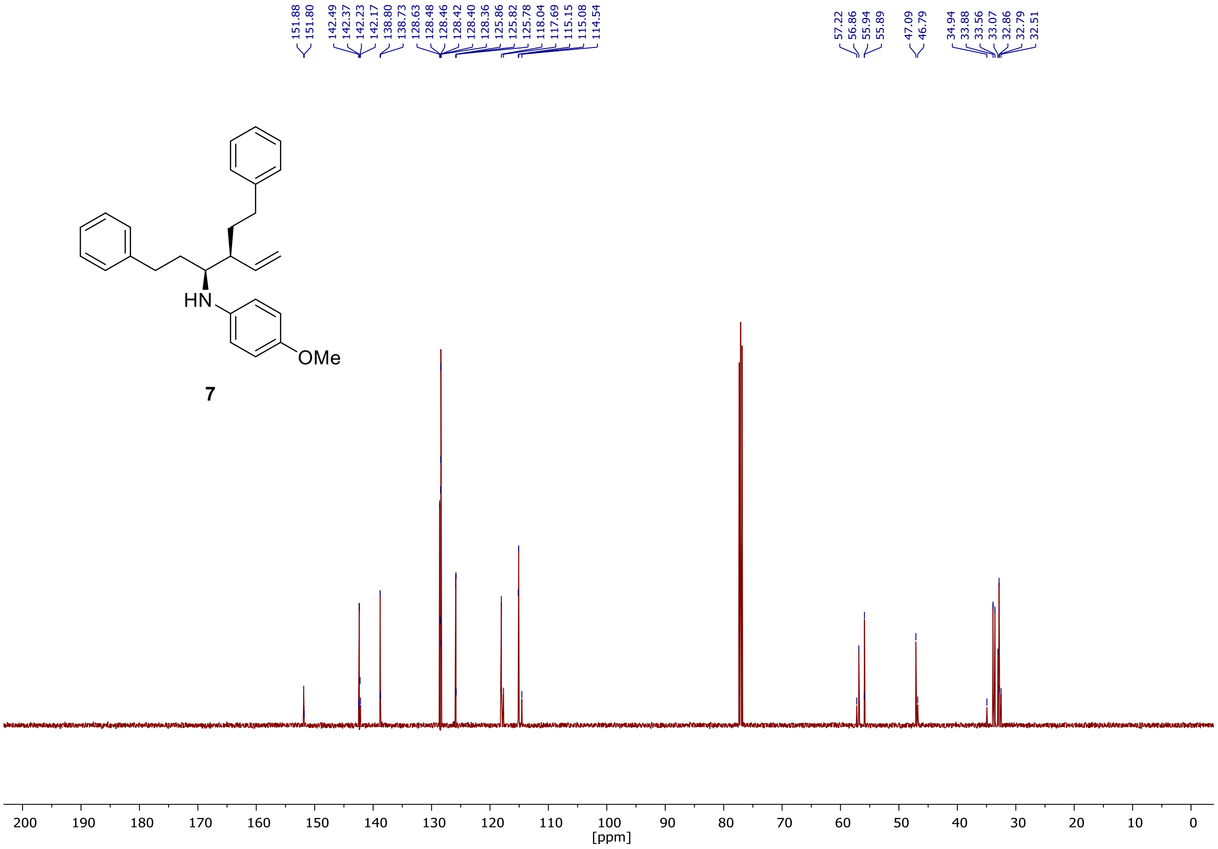

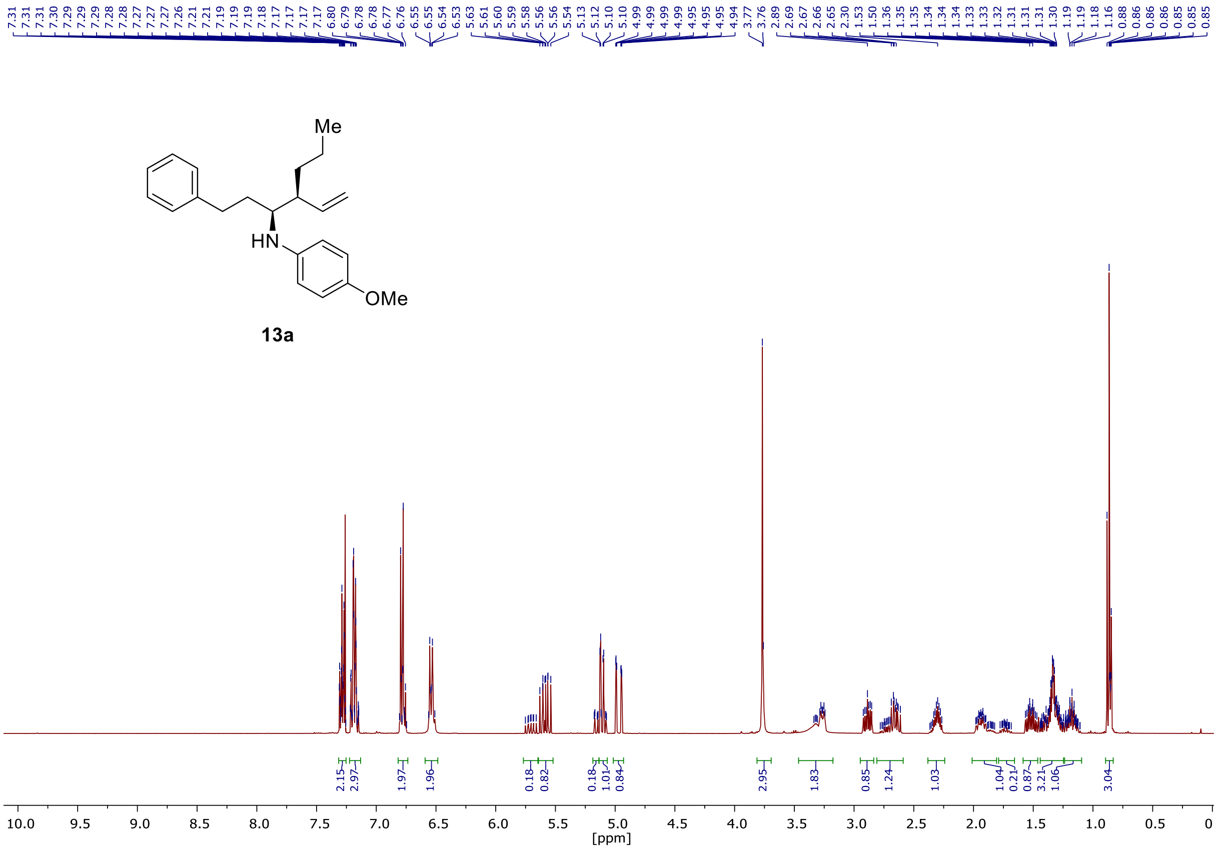
7.31 7.31 7.30 7.29 7.29 7.29 7.28 7.28 7.27 7.27 7.27 7.26 7.21 7.21 7.19 7.19 7.19 7.18 7.17 7.17 7.17 7.17 7.17 6.80 6.79 6.78 6.78 6.77 6.76 6.55 6.55 6.54 6.53 5.63 5.61 5.60 5.59 5.58 5.56 5.56 5.54 5.13 5.12 5.10 5.10 4.99 4.99 4.99 4.99 4.95 4.95 4.95 4.94 3.77 3.76 2.89 2.69 2.67 2.66 2.65 2.30 1.53 1.50 1.36 1.35 1.35 1.34 1.34 1.34 1.33 1.33 1.32 1.31 1.31 1.31 1.30 1.19 1.19 1.18 1.16 0.88 0.86 0.86 0.86 0.86 0.85 0.85 0.85
Me
HN
OMe
13a
2.15 2.97 1.97 1.96 0.18 0.82 0.18 1.01 0.84 2.95 1.83 0.85 1.24 1.03 1.04 0.21 0.87 3.21 1.06 3.04
10.0 9.5 9.0 8.5 8.0 7.5 7.0 6.5 6.0 5.5 5.0 4.5 4.0 3.5 3.0 2.5 2.0 1.5 1.0 0.5 0
[ppm]

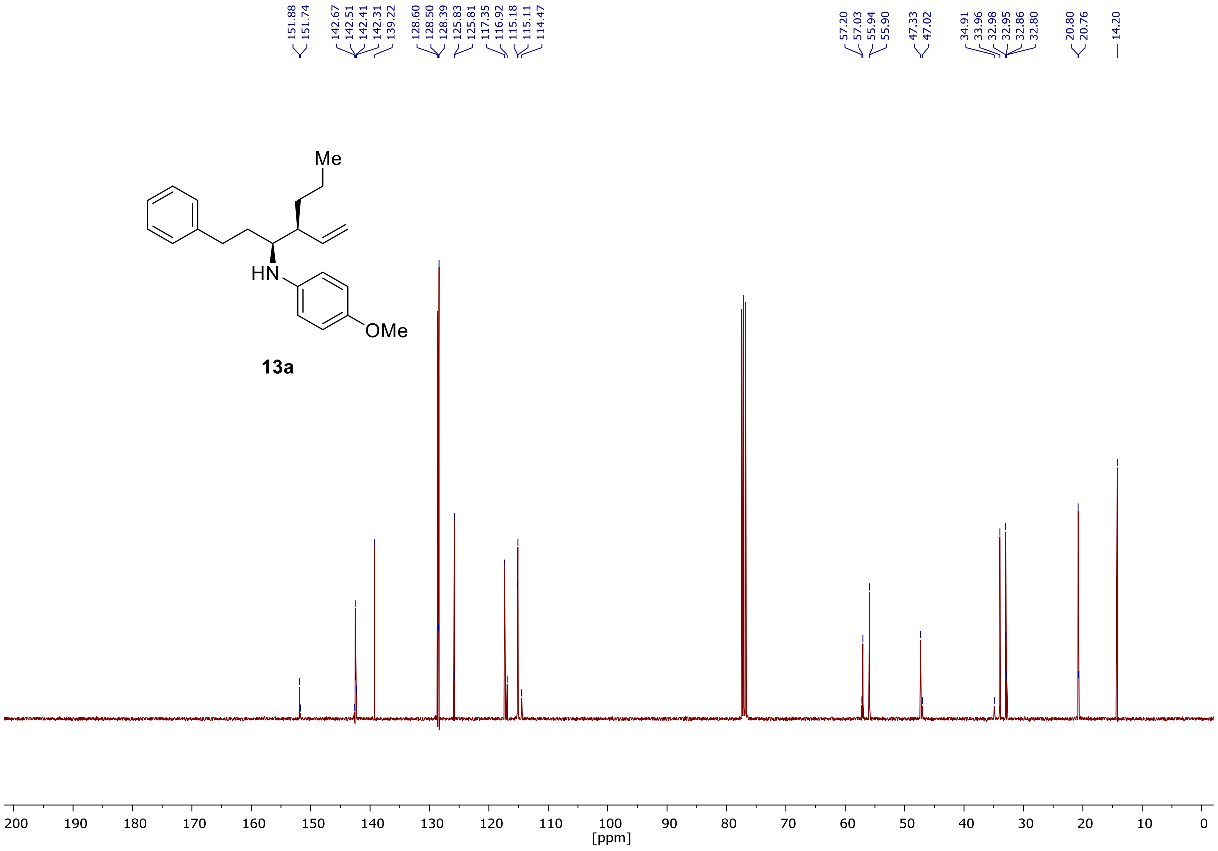
151.88 151.74 142.67 142.51 142.41 142.31 139.22 128.60 128.50 128.39 125.83 125.81 117.35 116.92 115.18 115.11 114.47
57.20 57.03 55.94 55.90 47.33 47.02 34.91 33.96 32.98 32.95 32.86 32.80 20.80 20.76 14.20
Me
HN
OMe
13a
200 190 180 170 160 150 140 130 120 110 100 90 80 70 60 50 40 30 20 10 0
[ppm]

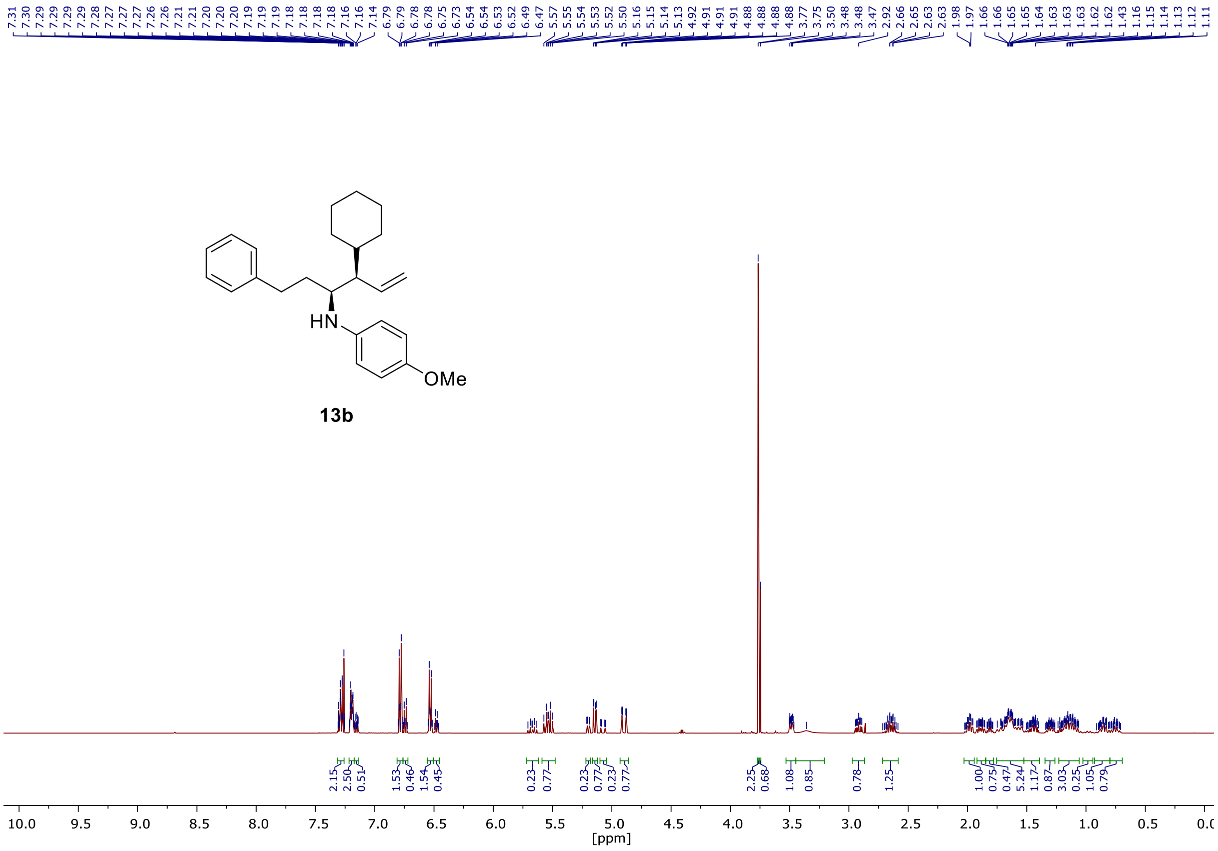
HN
OMe
13b
[ppm]

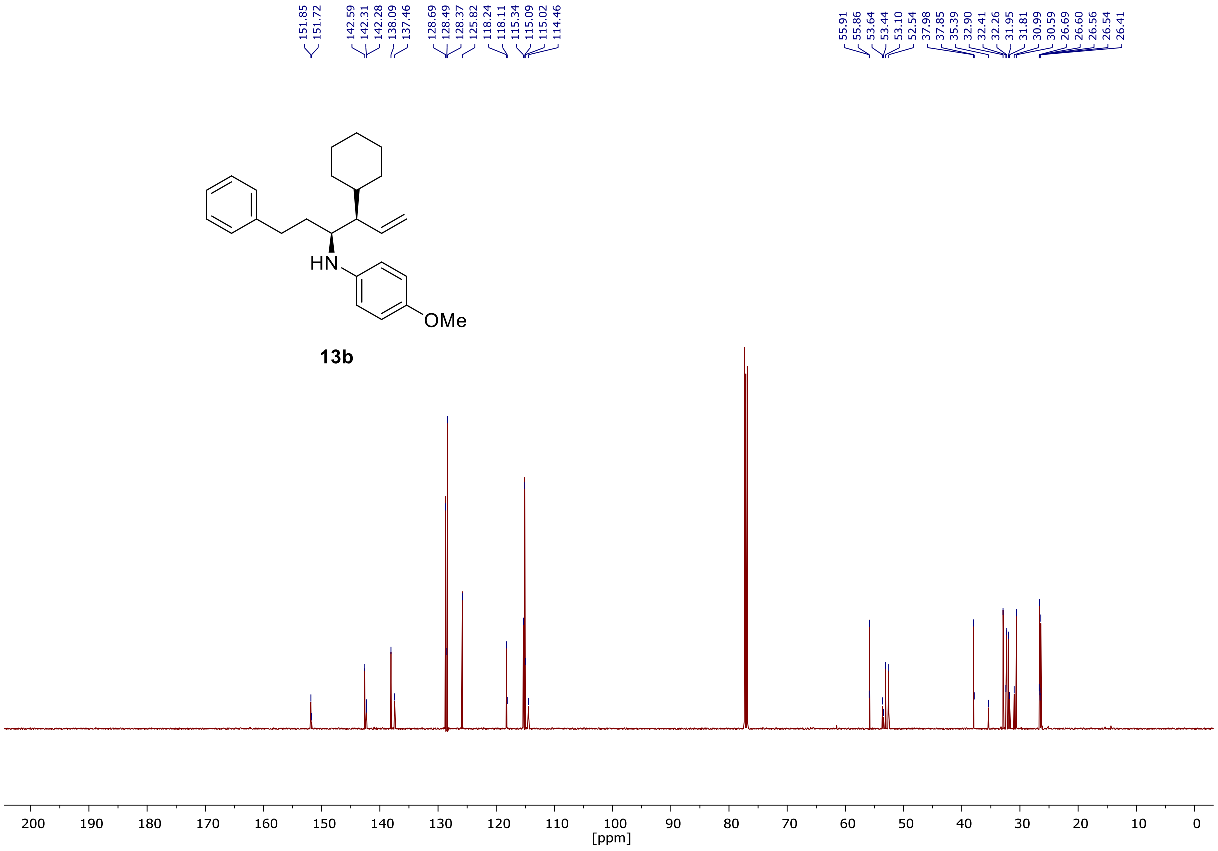
HN
OMe
13b
[ppm]

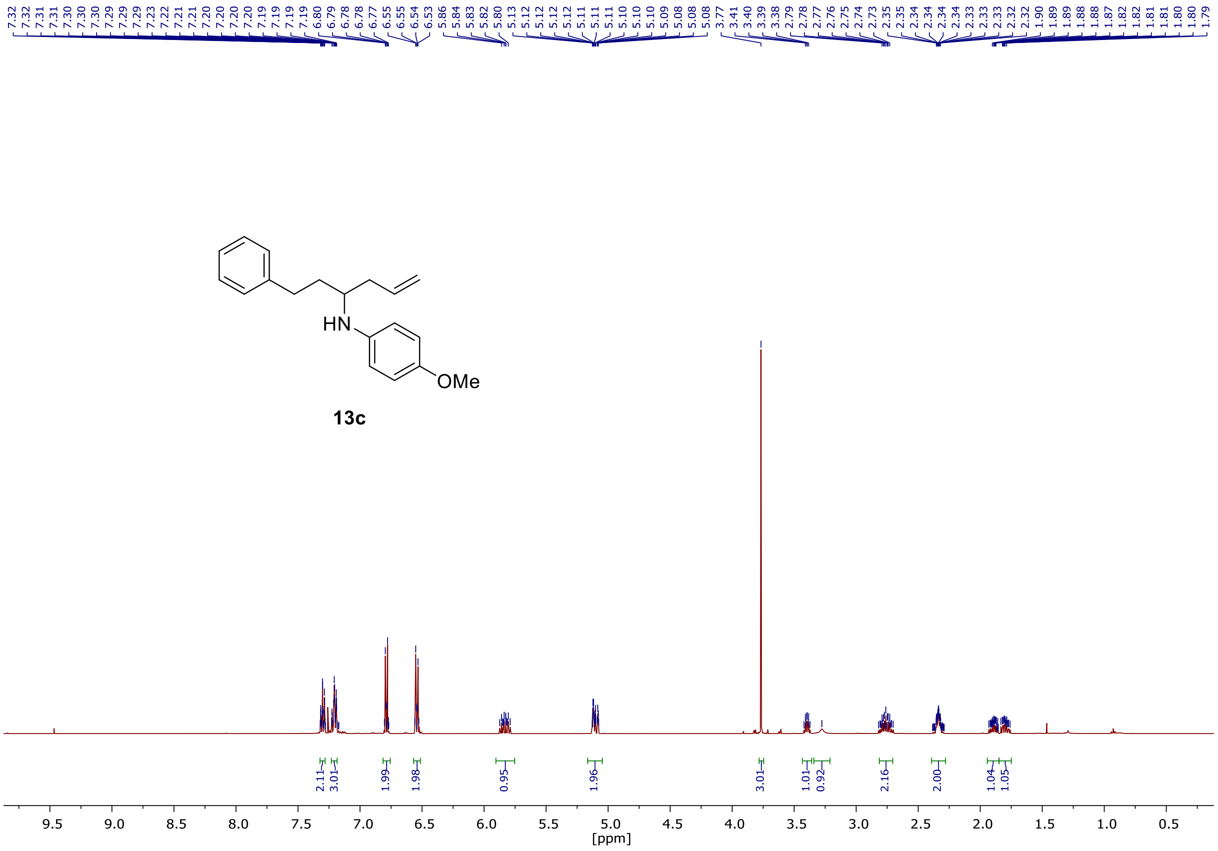
7.32 7.32 7.31 7.30 7.30 7.30 7.29 7.29 7.29 7.23 7.22 7.21 7.21 7.20 7.20 7.20 7.19 7.19 7.19 7.19 6.80 6.79 6.78 6.78 6.77 6.55 6.55 6.54 6.53 5.86 5.84 5.83 5.82 5.80 5.13 5.12 5.12 5.12 5.11 5.11 5.11 5.10 5.10 5.09 5.08 5.08 5.08 3.77 3.41 3.40 3.39 3.38 2.79 2.78 2.77 2.76 2.75 2.74 2.73 2.35 2.35 2.34 2.34 2.34 2.33 2.33 2.33 2.32 2.32 1.90 1.89 1.89 1.88 1.88 1.87 1.82 1.82 1.81 1.81 1.80 1.79
HN
OMe
13c
2.11 3.01 1.99 1.98 0.95 1.96 3.01 1.01 0.92 2.16 2.00 1.04 1.05
9.5 9.0 8.5 8.0 7.5 7.0 6.5 6.0 5.5 5.0 4.5 4.0 3.5 3.0 2.5 2.0 1.5 1.0 0.5
[ppm]

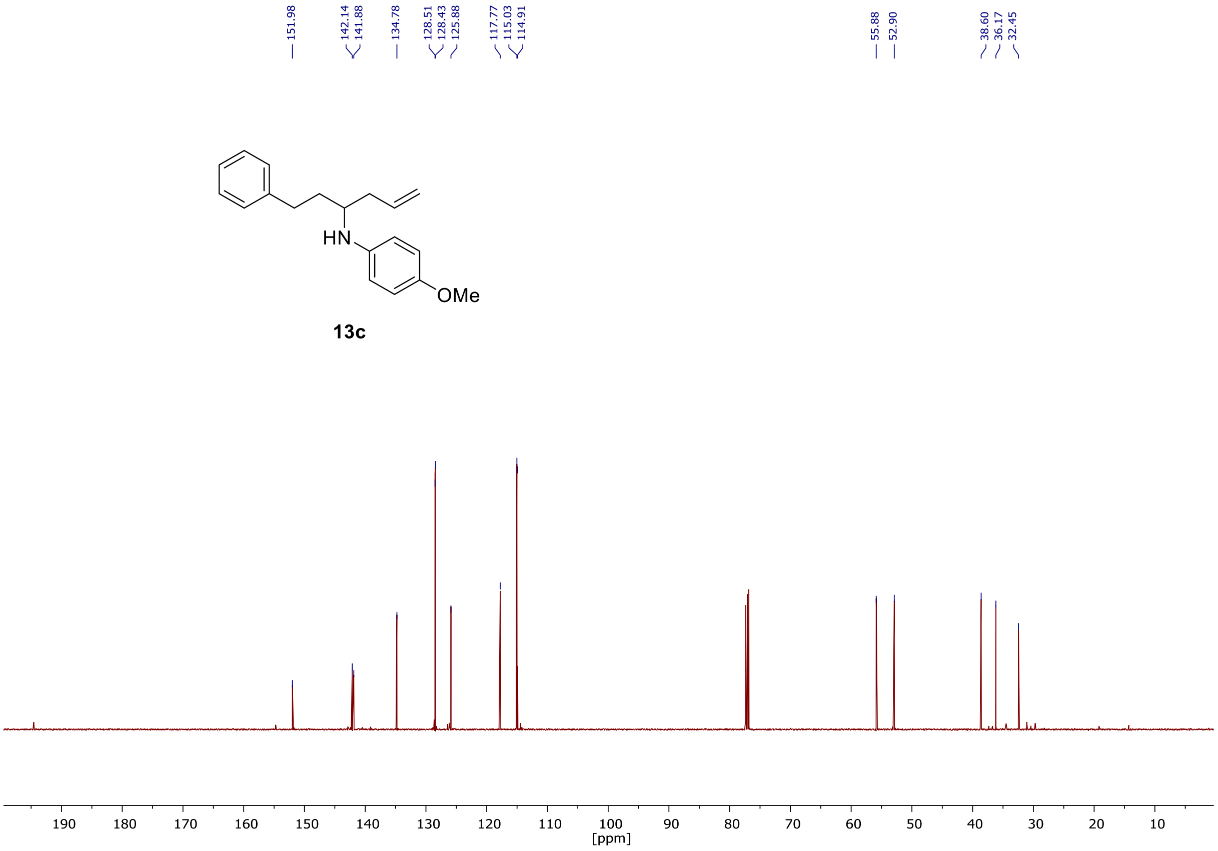
151.98
142.14 141.88
134.78
128.51 128.43 125.88
117.77 115.03 114.91
55.88 52.90
38.60 36.17 32.45
HN
OMe
13c
190 180 170 160 150 140 130 120 110 100 90 80 70 60 50 40 30 20 10
[ppm]

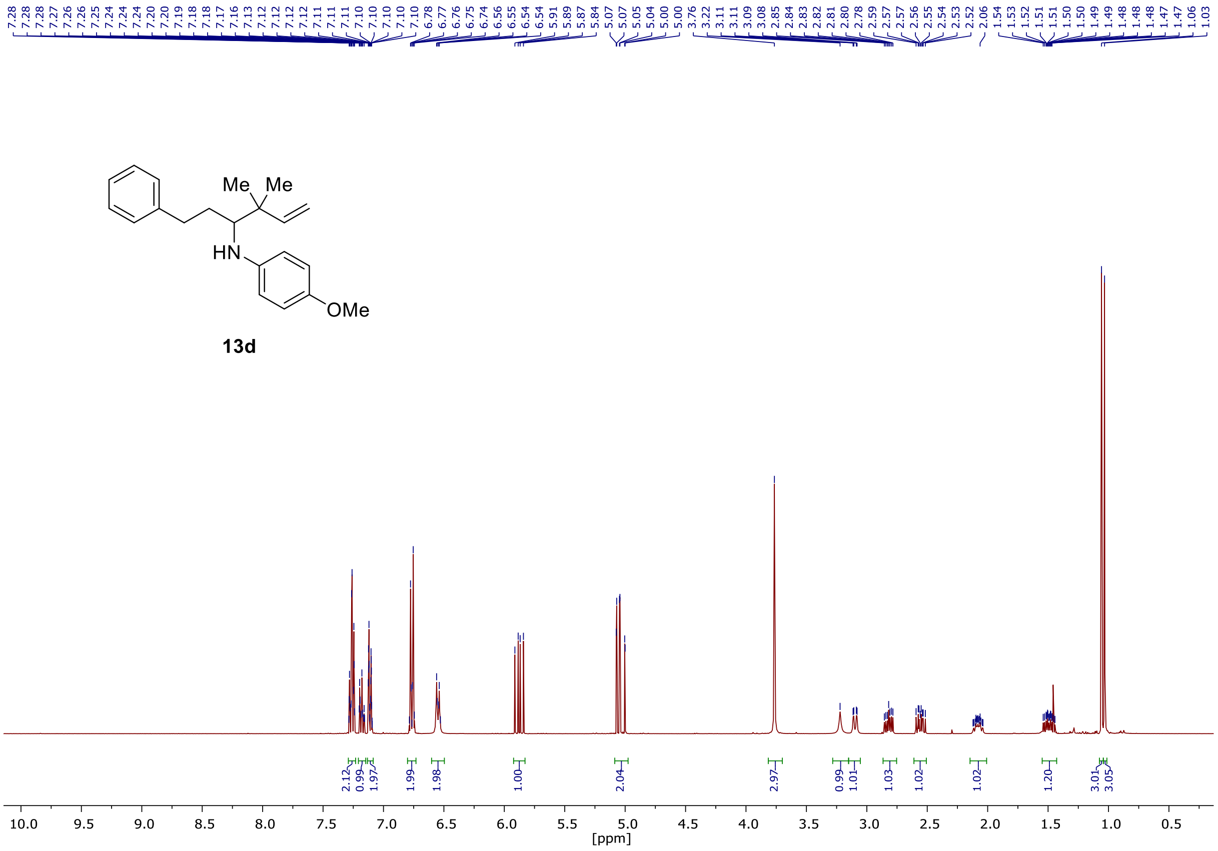

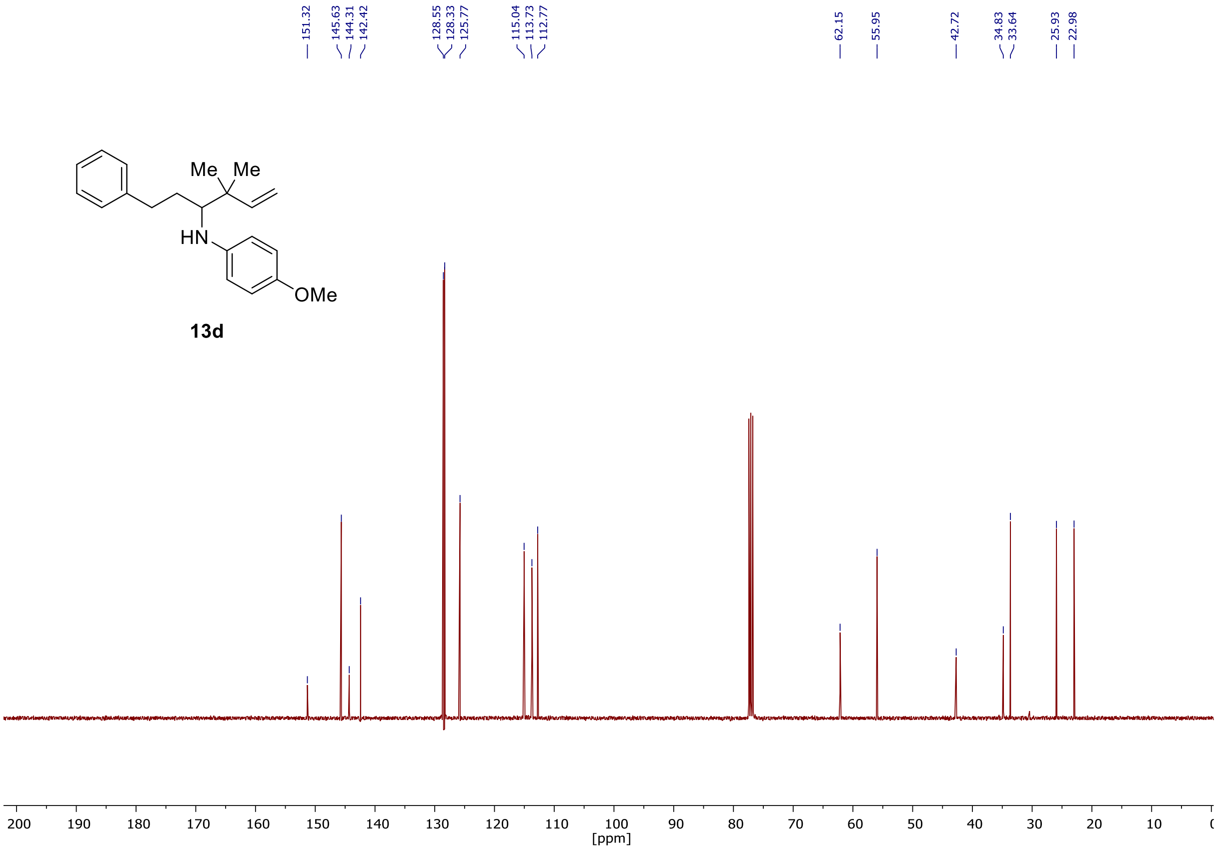

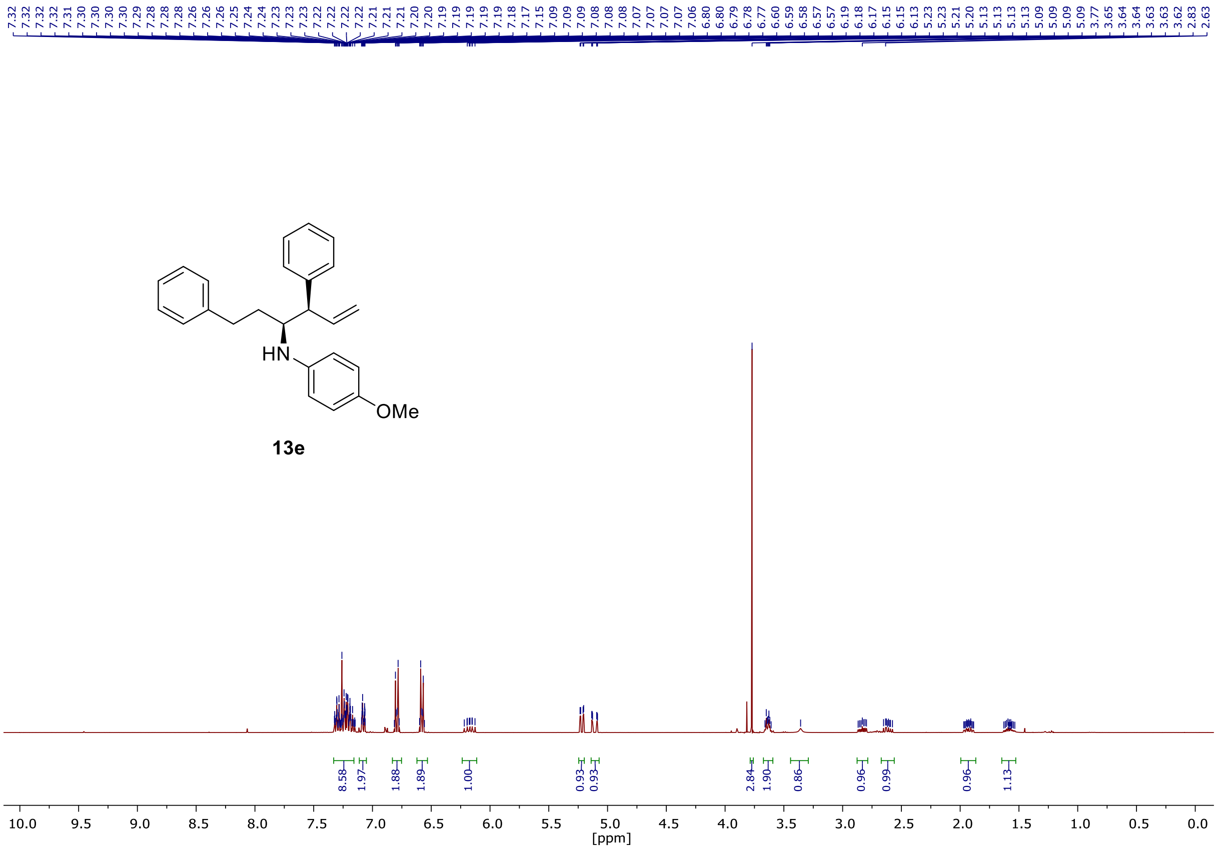
7.32 7.32 7.32 7.32 7.31 7.30 7.30 7.30 7.30 7.29 7.28 7.28 7.28 7.26 7.26 7.25 7.24 7.24 7.23 7.23 7.23 7.22 7.22 7.22 7.22 7.21 7.21 7.21 7.20 7.19 7.19 7.19 7.19 7.18 7.17 7.15 7.09 7.09 7.09 7.08 7.08 7.08 7.07 7.07 7.07 7.07 7.06 6.80 6.80 6.79 6.78 6.77 6.60 6.59 6.58 6.57 6.57 6.19 6.18 6.17 6.15 6.15 6.13 5.23 5.23 5.21 5.20 5.13 5.13 5.13 5.13 5.09 5.09 5.09 5.09 3.77 3.65 3.64 3.64 3.63 3.63 3.62 2.83 2.63
HN
OMe
13e
8.58 1.97 1.88 1.89 1.00 0.93 0.93 2.84 1.90 0.86 0.96 0.99 0.96 1.13
10.0 9.5 9.0 8.5 8.0 7.5 7.0 6.5 6.0 5.5 5.0 4.5 4.0 3.5 3.0 2.5 2.0 1.5 1.0 0.5 0.0
[ppm]

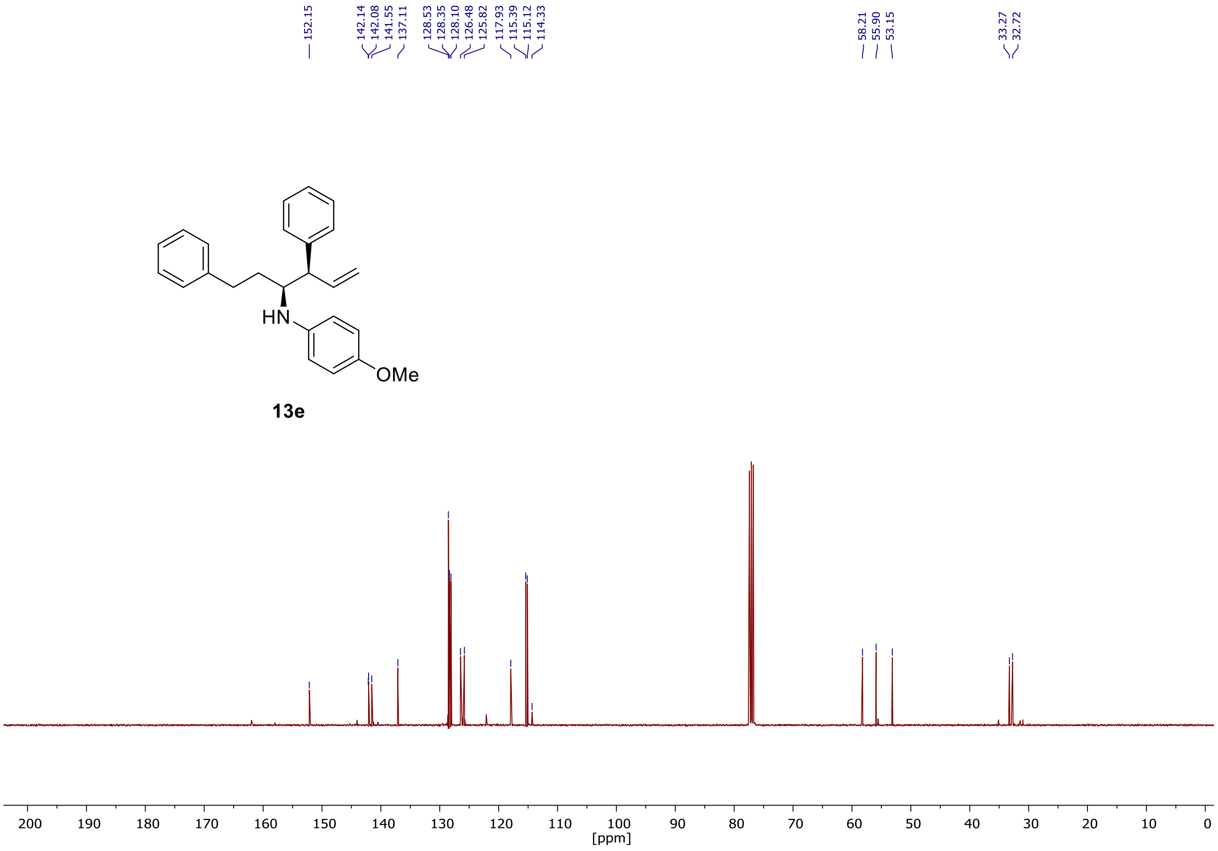
152.15
142.14 142.08 141.55 137.11
128.53 128.35 128.10 126.48 125.82
117.93 115.39 115.12 114.33
58.21 55.90 53.15
33.27 32.72
HN
OMe
13e
200 190 180 170 160 150 140 130 120 110 100 90 80 70 60 50 40 30 20 10 0
[ppm]

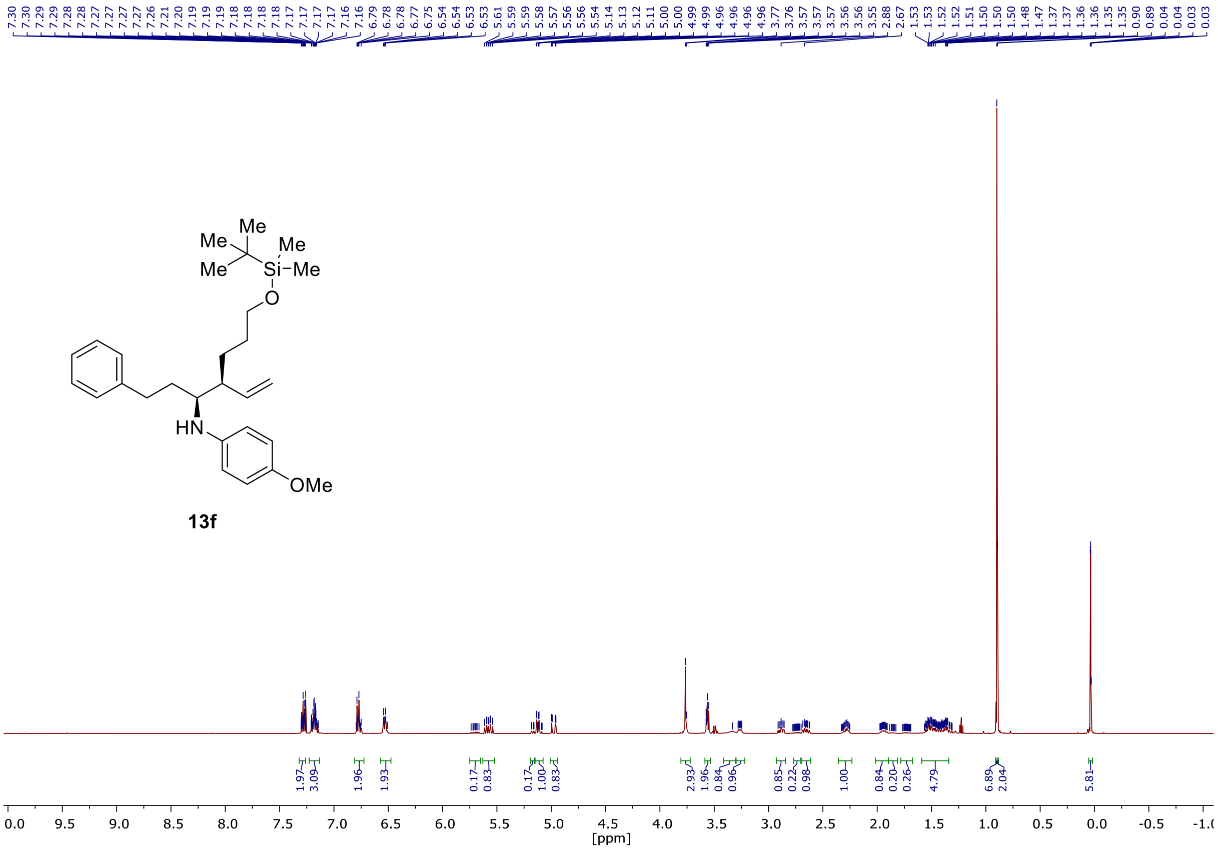
7.30 7.30 7.29 7.29 7.28 7.28 7.27 7.27 7.27 7.27 7.26 7.21 7.20 7.19 7.19 7.19 7.18 7.18 7.18 7.18 7.17 7.17 7.17 7.17 7.16 7.16 6.79 6.78 6.78 6.77 6.75 6.54 6.54 6.53 6.53 5.61 5.59 5.59 5.58 5.57 5.56 5.56 5.54 5.14 5.13 5.12 5.11 5.00 5.00 4.99 4.99 4.96 4.96 4.96 4.96 3.77 3.76 3.57 3.57 3.57 3.56 3.56 3.55 2.88 2.67 1.53 1.53 1.52 1.52 1.51 1.50 1.50 1.50 1.48 1.47 1.37 1.37 1.36 1.36 1.35 1.35 0.90 0.89 0.04 0.04 0.03 0.03
Me
Me
Me
Me
Si
Me
O
HN
OMe
13f
1.97 3.09 1.96 1.93 0.17 0.83 0.17 1.00 0.83 2.93 1.96 0.84 0.96 0.85 0.22 0.98 1.00 0.84 0.20 0.26 4.79 6.89 2.04 5.81
10.0 9.5 9.0 8.5 8.0 7.5 7.0 6.5 6.0 5.5 5.0 4.5 4.0 3.5 3.0 2.5 2.0 1.5 1.0 0.5 0.0 -0.5 -1.0
[ppm]

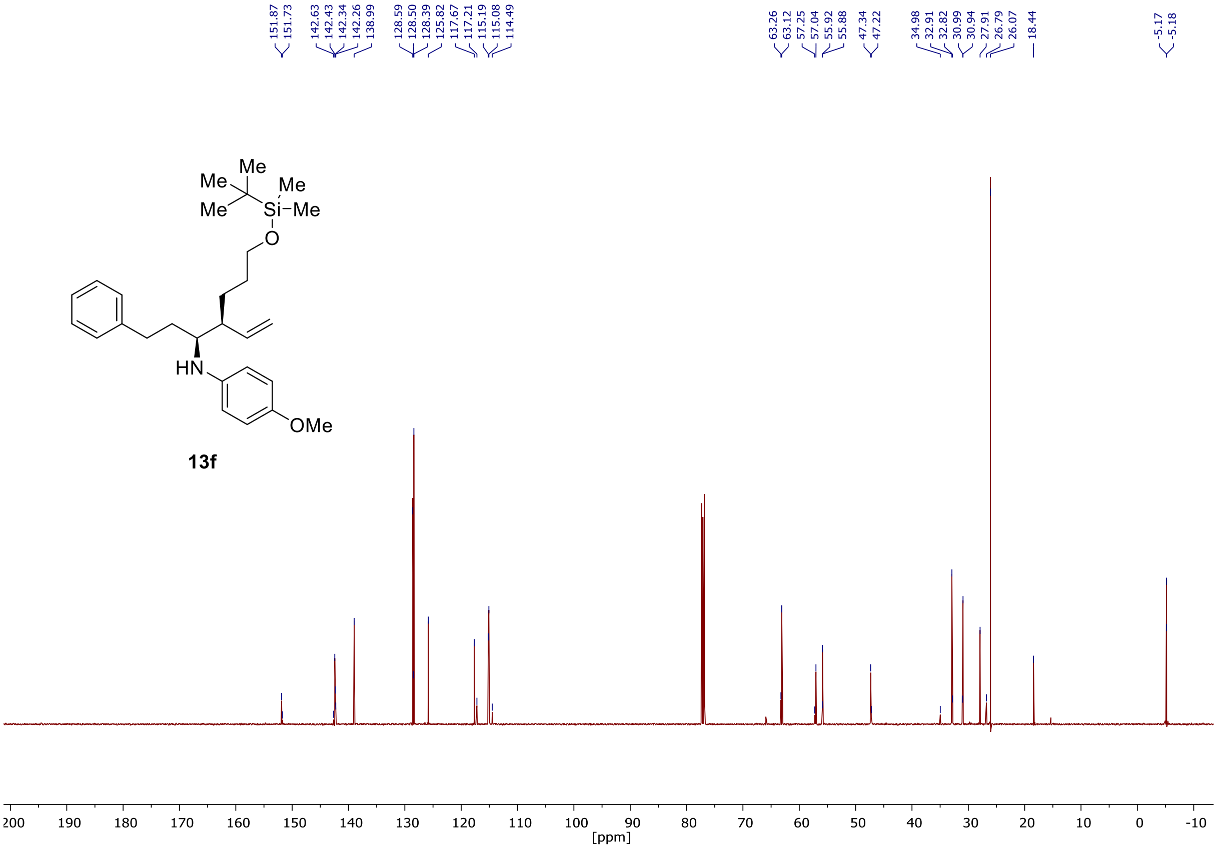
151.87 151.73 142.63 142.43 142.34 142.26 138.99 128.59 128.50 128.39 125.82 117.67 117.21 115.19 115.08 114.49 63.26 63.12 57.25 57.04 55.92 55.88 47.34 47.22 34.98 32.91 32.82 30.99 30.94 27.91 26.79 26.07 18.44 -5.17 -5.18
Me
Me
Me
Me
Si
Me
O
HN
OMe
13f
200 190 180 170 160 150 140 130 120 110 100 90 80 70 60 50 40 30 20 10 0 -10
[ppm]

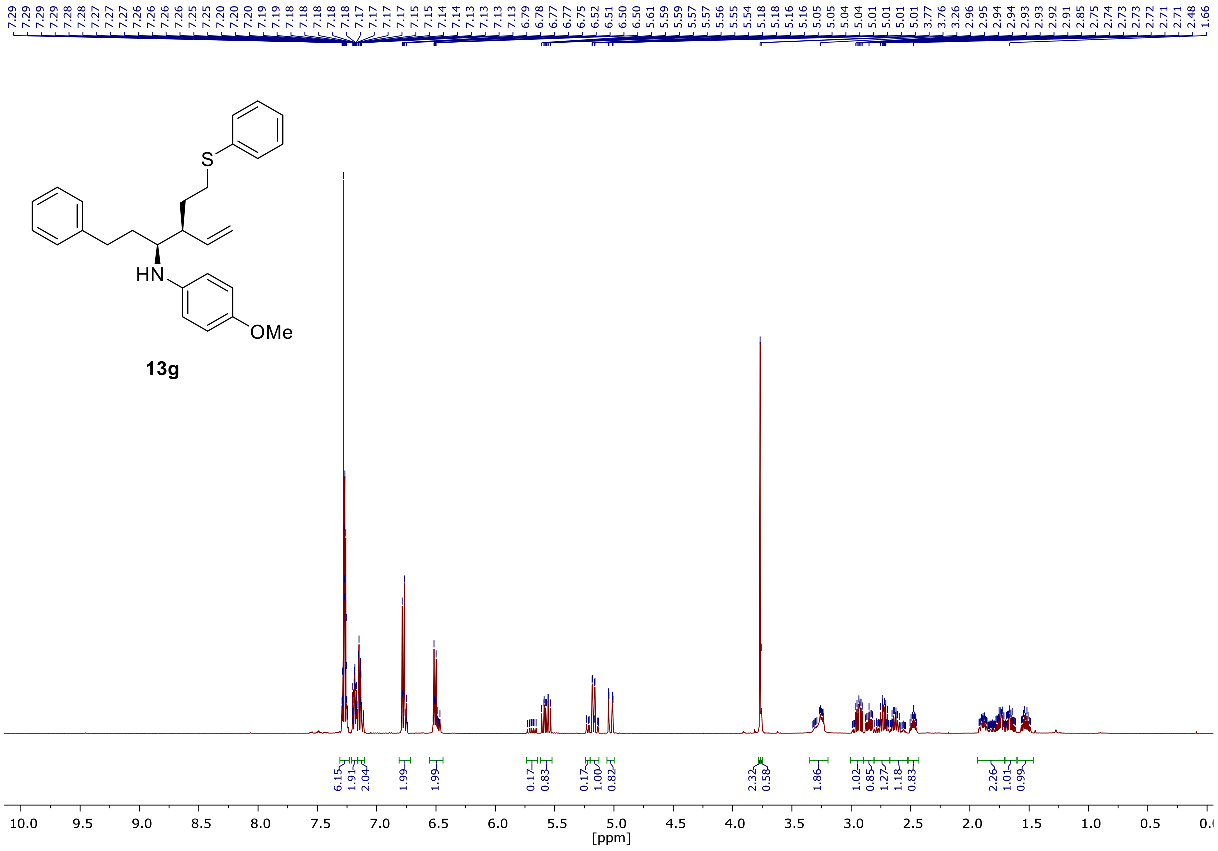
7.29 7.29 7.29 7.28 7.27 7.27 7.27 7.26 7.26 7.26 7.25 7.25 7.20 7.20 7.19 7.19 7.18 7.18 7.18 7.18 7.17 7.17 7.17 7.15 7.14 7.14 7.13 7.13 7.13 7.13 6.79 6.78 6.77 6.77 6.75 6.52 6.51 6.50 6.50 5.61 5.59 5.59 5.57 5.56 5.55 5.55 5.54 5.18 5.18 5.16 5.16 5.05 5.05 5.04 5.04 5.01 5.01 5.01 3.77 3.76 3.26 2.96 2.95 2.94 2.94 2.93 2.93 2.92 2.91 2.85 2.75 2.74 2.73 2.72 2.71 2.48 1.66
S
HN
OMe
13g
6.15 1.91 2.04 1.99 1.99 0.17 0.83 0.17 1.00 0.82 2.32 0.58 1.86 1.02 0.85 1.27 1.18 0.83 2.26 1.01 0.99
[ppm]

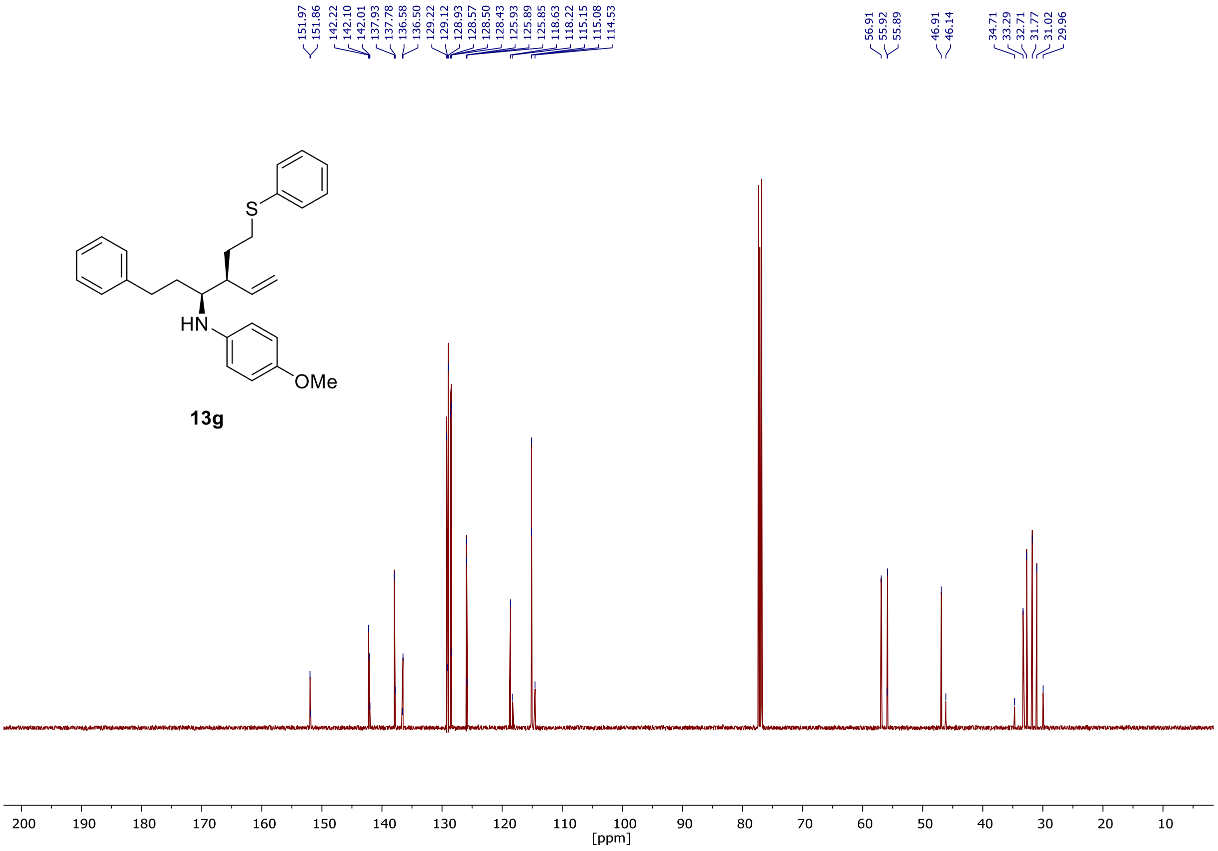
151.97 151.86 142.22 142.10 142.01 137.93 137.78 136.58 136.50 129.22 129.12 128.93 128.57 128.50 128.43 125.93 125.89 125.85 118.63 118.22 115.15 115.08 114.53
56.91 55.92 55.89 46.91 46.14 34.71 33.29 32.71 31.77 31.02 29.96
S
HN
OMe
13g
[ppm]

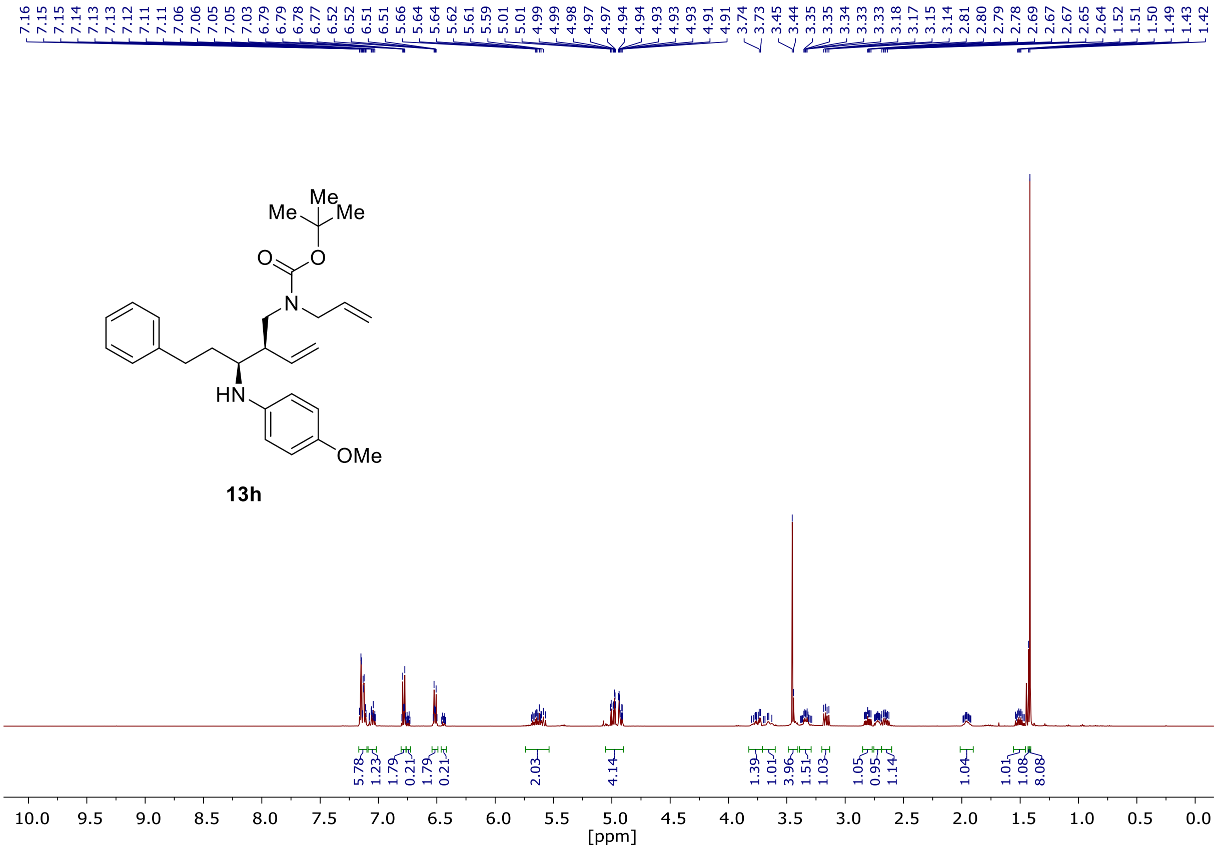
7.16 7.15 7.15 7.14 7.13 7.13 7.12 7.11 7.11 7.06 7.05 7.05 7.03 6.79 6.79 6.78 6.77 6.52 6.52 6.51 6.51 5.66 5.64 5.64 5.62 5.61 5.59 5.01 5.01 4.99 4.99 4.98 4.97 4.97 4.94 4.94 4.93 4.93 4.93 4.91 4.91 3.74 3.73 3.45 3.44 3.35 3.35 3.34 3.33 3.33 3.18 3.17 3.15 3.14 2.81 2.80 2.79 2.78 2.69 2.67 2.65 2.64 1.52 1.51 1.50 1.49 1.43 1.42
Me
Me
Me
O
O
N
HN
OMe
13h
5.78 1.23 1.79 0.21 1.79 0.21 2.03 4.14 1.39 1.01 3.96 1.51 1.03 1.05 0.95 1.14 1.04 1.01 1.08 8.08
10.0 9.5 9.0 8.5 8.0 7.5 7.0 6.5 6.0 5.5 5.0 4.5 4.0 3.5 3.0 2.5 2.0 1.5 1.0 0.5 0.0
[ppm]

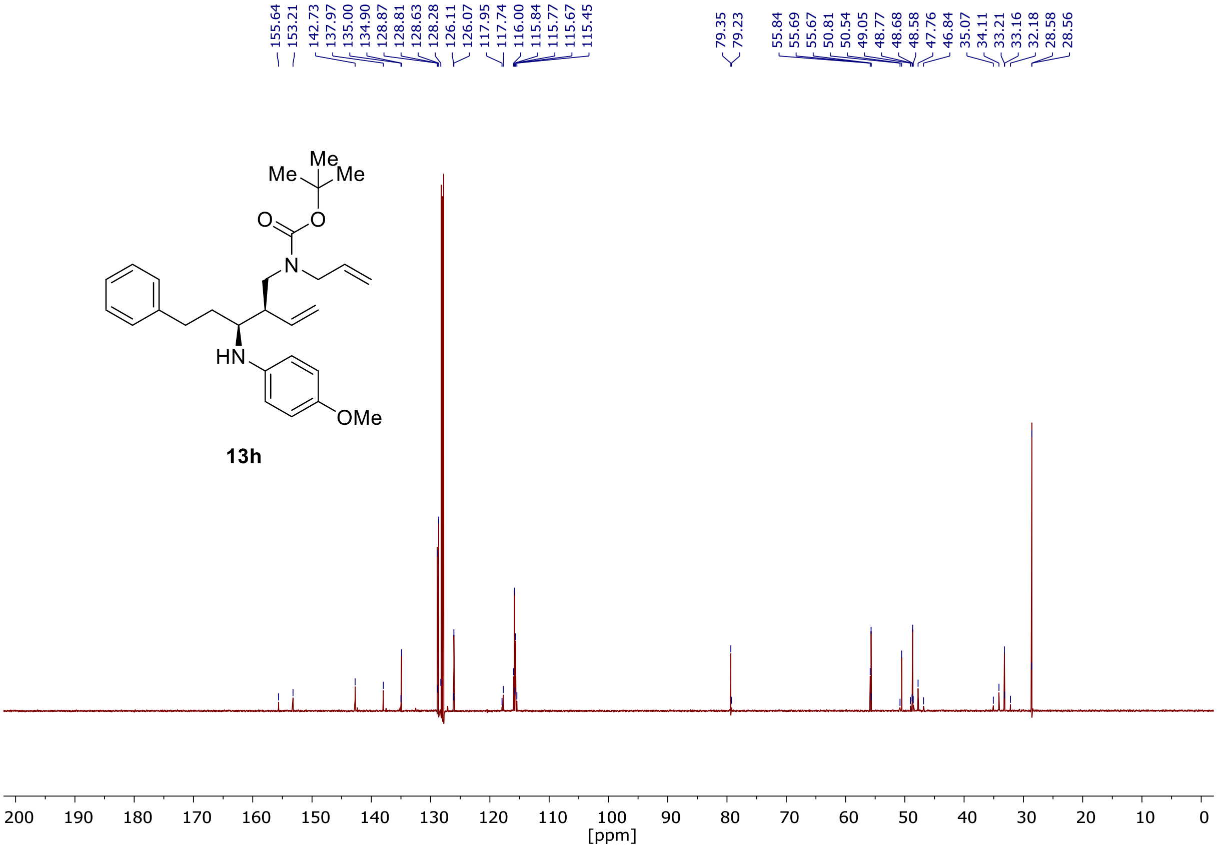
155.64 153.21 142.73 137.97 135.00 134.90 128.87 128.81 128.63 128.28 126.11 126.07 117.95 117.74 116.00 115.84 115.77 115.67 115.45
79.35 79.23
55.84 55.69 55.67 50.81 50.54 49.05 48.77 48.68 48.58 47.76 46.84 35.07 34.11 33.21 33.16 32.18 28.58 28.56
Me
Me
Me
O
O
N
HN
OMe
13h
200 190 180 170 160 150 140 130 120 110 100 90 80 70 60 50 40 30 20 10 0
[ppm]

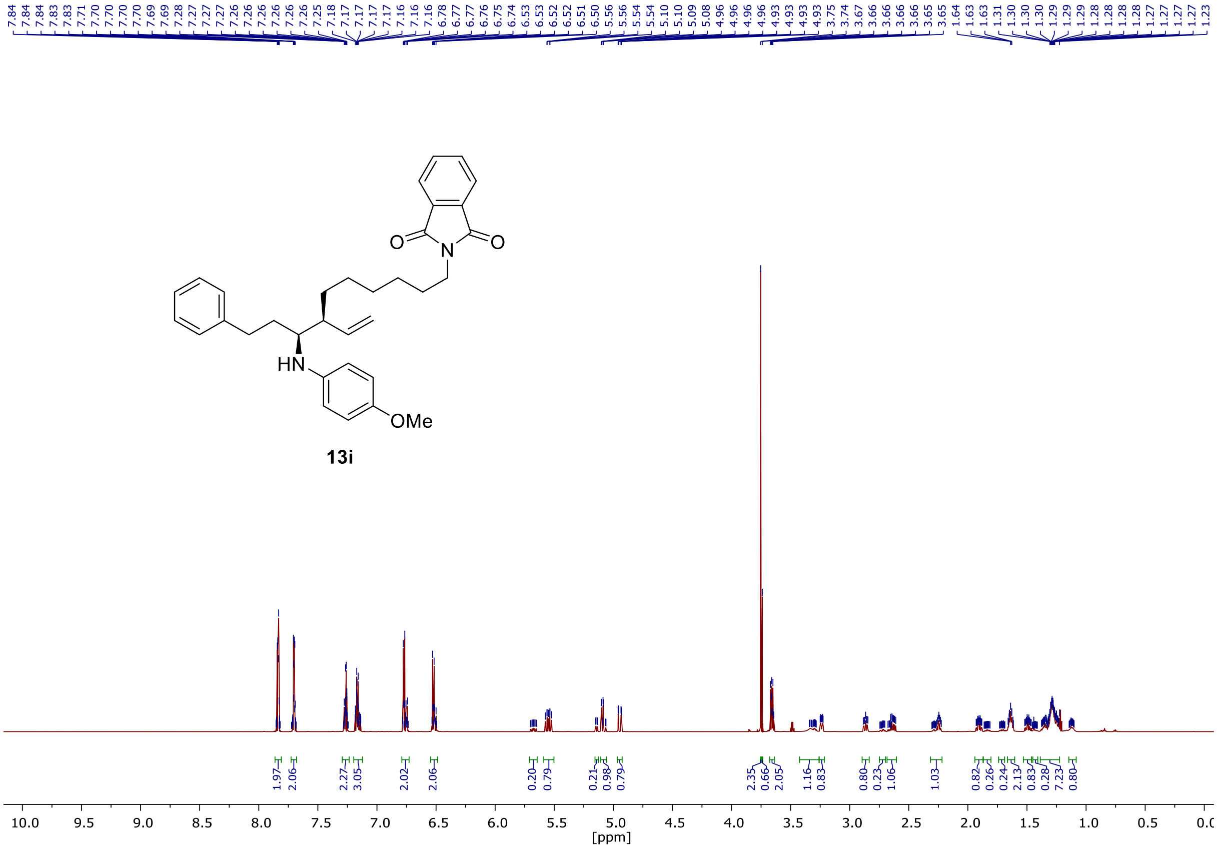
7.84 7.84 7.84 7.83 7.71 7.70 7.70 7.70 7.69 7.69 7.28 7.27 7.27 7.26 7.26 7.26 7.26 7.26 7.25 7.18 7.17 7.17 7.17 7.16 7.16 6.78 6.77 6.77 6.76 6.75 6.74 6.53 6.53 6.52 6.52 6.51 6.50 5.56 5.56 5.54 5.54 5.10 5.10 5.09 5.08 4.96 4.96 4.96 4.93 4.93 4.93 4.93 3.75 3.74 3.67 3.66 3.66 3.66 3.66 3.65 1.64 1.63 1.63 1.31 1.30 1.30 1.30 1.29 1.29 1.29 1.28 1.28 1.28 1.28 1.27 1.27 1.27 1.23
O
N
O
HN
OMe
13i
1.97 2.06 2.27 3.05 2.02 2.06 0.20 0.79 0.21 0.98 0.79 2.35 0.66 2.05 1.16 0.83 0.80 0.23 1.06 1.03 0.82 0.26 0.24 2.13 0.83 0.28 7.23 0.80
10.0 9.5 9.0 8.5 8.0 7.5 7.0 6.5 6.0 5.5 5.0 4.5 4.0 3.5 3.0 2.5 2.0 1.5 1.0 0.5 0.0
[ppm]

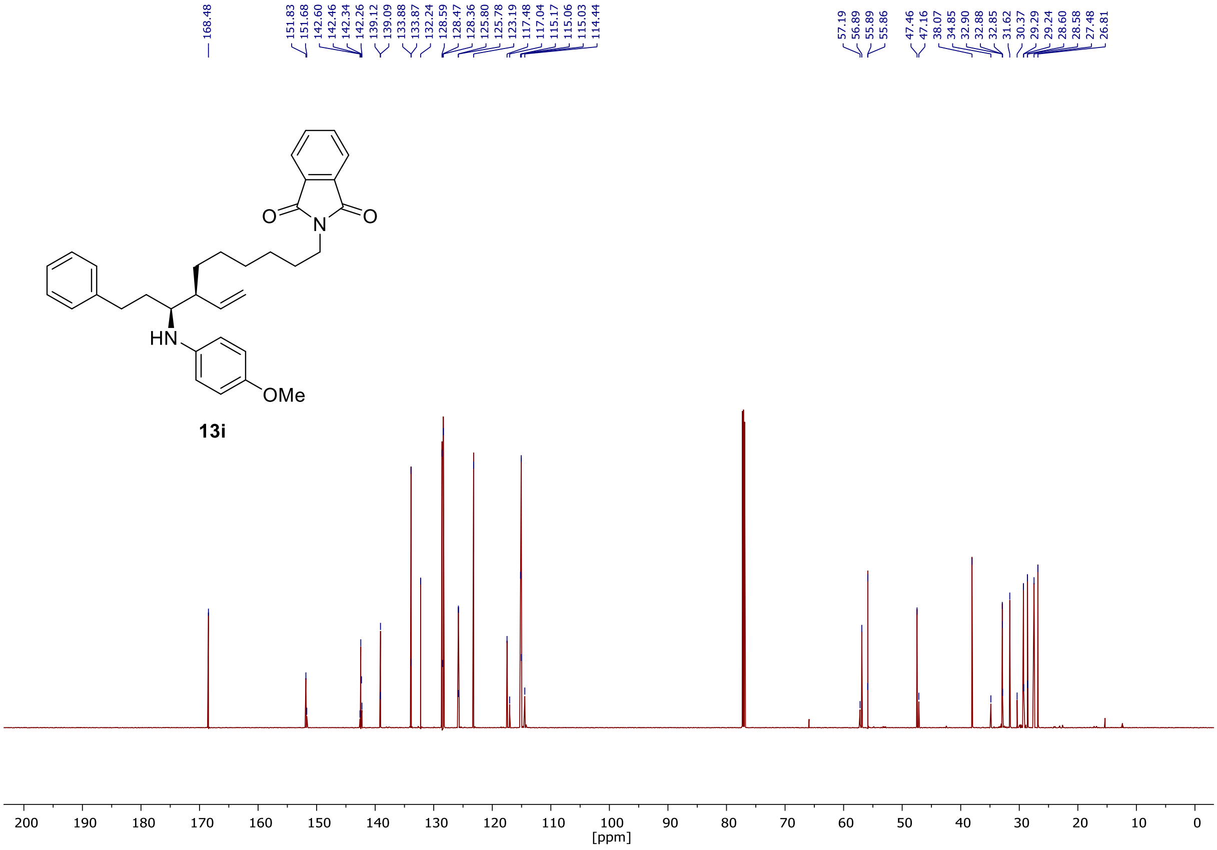
168.48
151.83 151.68 142.60 142.46 142.34 142.26 139.12 139.09 133.88 133.87 132.24 128.59 128.47 128.36 125.80 125.78 123.19 117.48 117.04 115.17 115.06 115.03 114.44
57.19 56.89 55.89 55.86 47.46 47.16 38.07 34.85 32.90 32.88 32.85 31.62 30.37 29.29 29.24 28.60 28.58 27.48 26.81
O
N
O
HN
OMe
13i
200 190 180 170 160 150 140 130 120 110 100 90 80 70 60 50 40 30 20 10 0
[ppm]

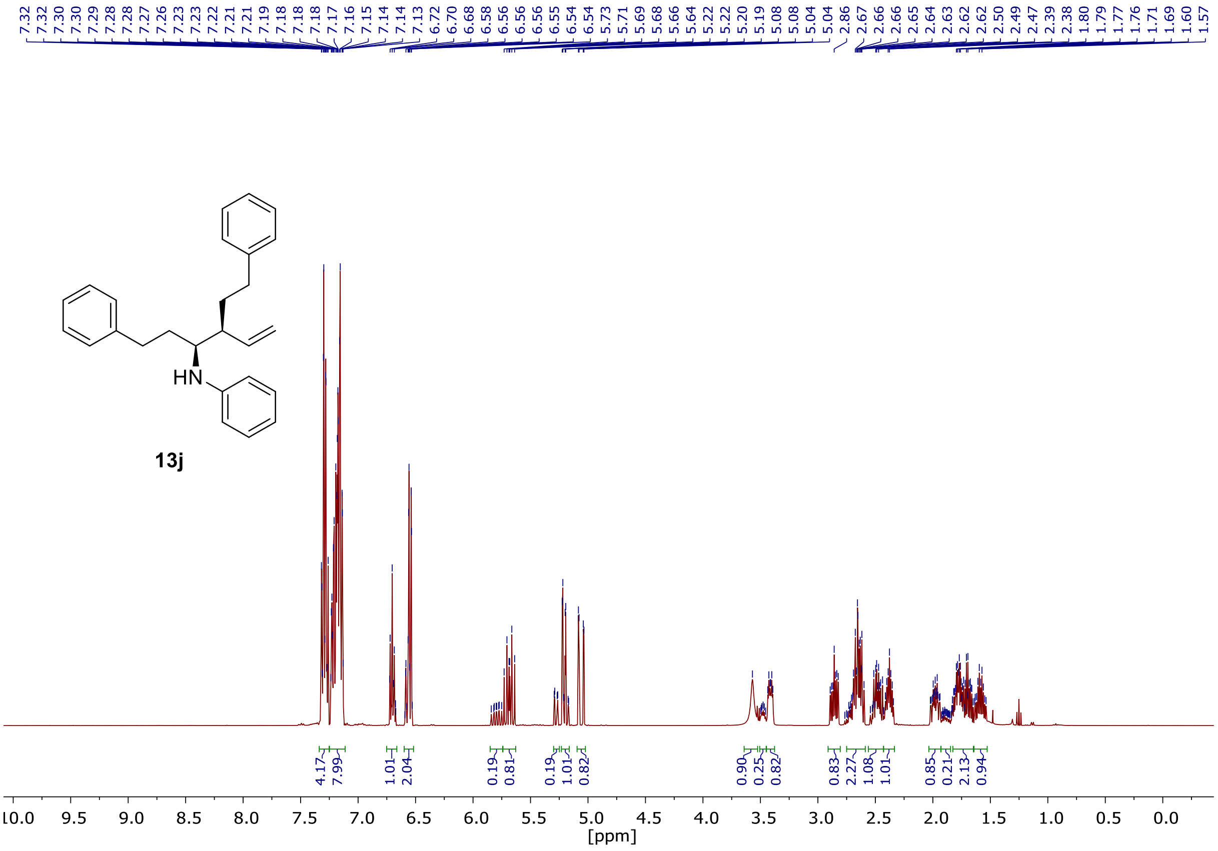
7.32 7.32 7.30 7.29 7.28 7.28 7.27 7.26 7.23 7.23 7.22 7.21 7.21 7.19 7.18 7.18 7.18 7.17 7.16 7.15 7.14 7.13 6.72 6.70 6.68 6.58 6.56 6.56 6.56 6.55 6.54 6.54 5.73 5.71 5.69 5.68 5.66 5.64 5.22 5.22 5.20 5.19 5.08 5.08 5.04 5.04 2.86 2.67 2.66 2.66 2.65 2.64 2.63 2.62 2.62 2.50 2.49 2.47 2.39 2.38 1.80 1.79 1.77 1.76 1.71 1.69 1.60 1.57
HN
13j
4.17 7.99 1.01 2.04 0.19 0.81 0.19 1.01 0.82 0.90 0.25 0.82 0.83 2.27 1.08 1.01 0.85 0.21 2.13 0.94
10.0 9.5 9.0 8.5 8.0 7.5 7.0 6.5 6.0 5.5 5.0 4.5 4.0 3.5 3.0 2.5 2.0 1.5 1.0 0.5 0.0
[ppm]

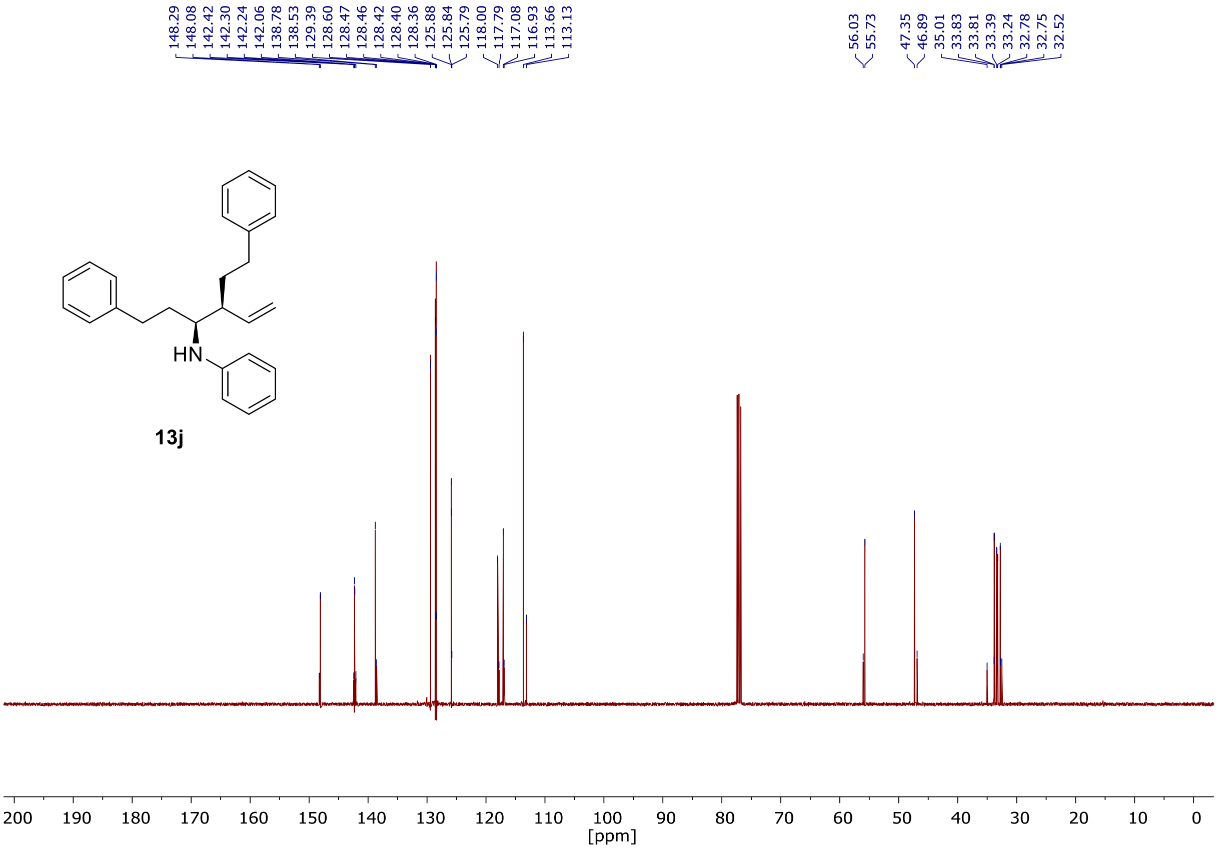
148.29 148.08 142.42 142.30 142.24 142.06 138.78 138.53 129.39 128.60 128.47 128.46 128.42 128.40 128.36 125.88 125.84 125.79 118.00 117.79 117.08 116.93 113.66 113.13
56.03 55.73 47.35 46.89 35.01 33.83 33.81 33.39 33.24 32.78 32.75 32.52
HN
13j
200 190 180 170 160 150 140 130 120 110 100 90 80 70 60 50 40 30 20 10 0
[ppm]

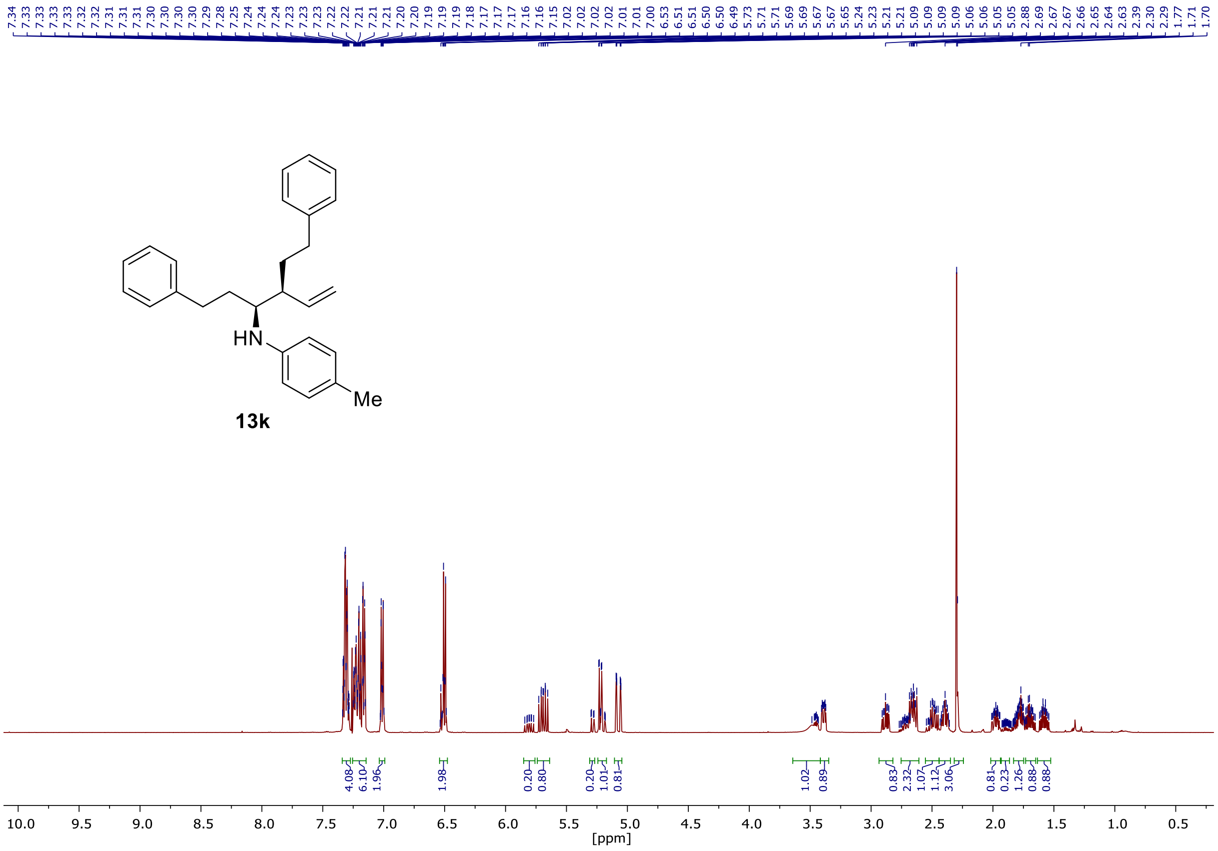
7.34 7.33 7.33 7.33 7.33 7.32 7.32 7.31 7.31 7.31 7.30 7.30 7.30 7.30 7.29 7.28 7.25 7.24 7.24 7.24 7.23 7.23 7.23 7.22 7.22 7.21 7.21 7.21 7.20 7.19 7.19 7.19 7.18 7.17 7.17 7.17 7.16 7.16 7.15 7.02 7.02 7.02 7.02 7.01 7.01 7.00 6.53 6.51 6.51 6.50 6.50 6.49 5.73 5.71 5.71 5.69 5.69 5.67 5.67 5.65 5.24 5.23 5.21 5.21 5.09 5.09 5.09 5.09 5.06 5.05 5.05 2.88 2.69 2.67 2.67 2.66 2.65 2.64 2.63 2.39 2.30 2.29 1.77 1.71 1.70
HN
Me
13k
4.08 6.10 1.96 1.98 0.20 0.80 0.20 1.01 0.81 1.02 0.89 0.83 2.32 1.07 1.12 3.06 0.81 0.23 1.26 0.88 0.88
10.0 9.5 9.0 8.5 8.0 7.5 7.0 6.5 6.0 5.5 5.0 4.5 4.0 3.5 3.0 2.5 2.0 1.5 1.0 0.5
[ppm]

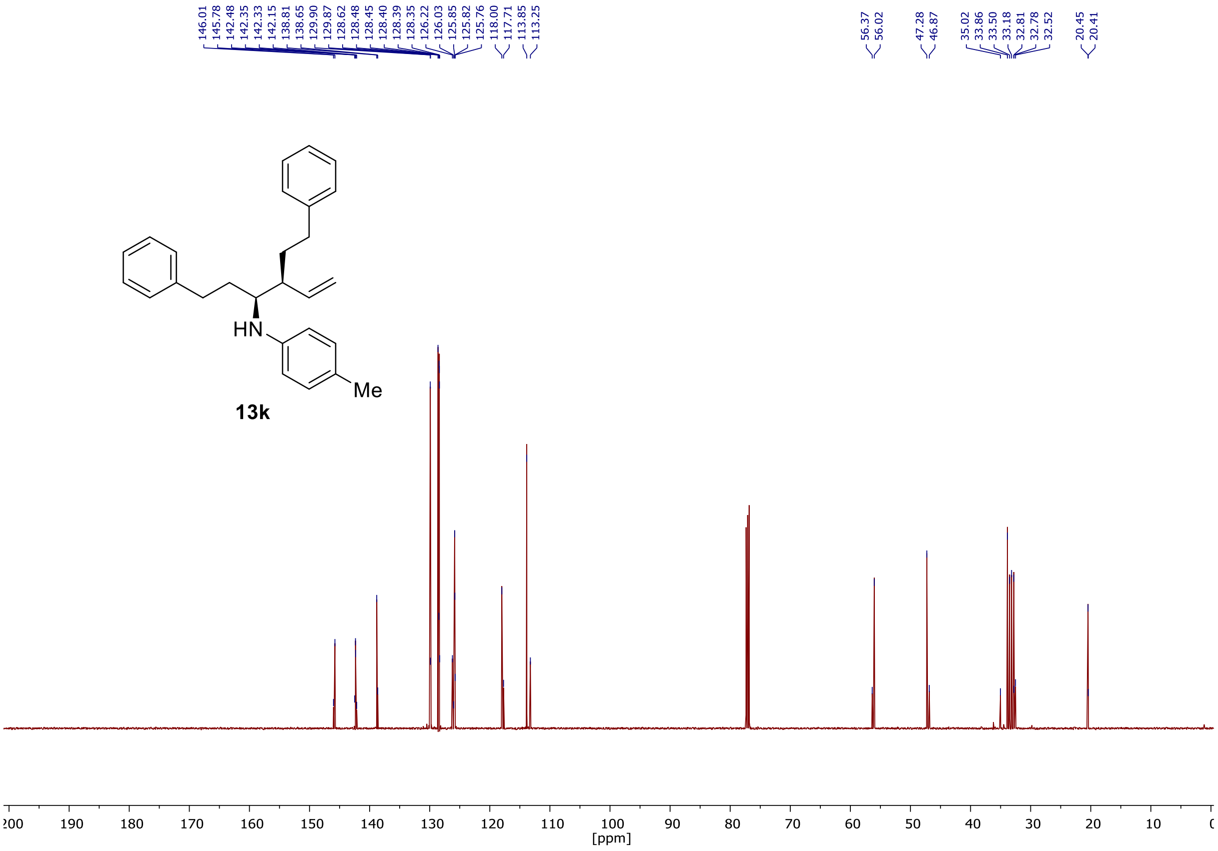
146.01 145.78 142.48 142.35 142.33 142.15 138.81 138.65 129.90 129.87 128.62 128.48 128.45 128.40 128.39 128.35 126.22 126.03 125.85 125.82 125.76 118.00 117.71 113.85 113.25
56.37 56.02 47.28 46.87 35.02 33.86 33.50 33.18 32.81 32.78 32.52 20.45 20.41
HN
Me
13k
200 190 180 170 160 150 140 130 120 110 100 90 80 70 60 50 40 30 20 10 0
[ppm]

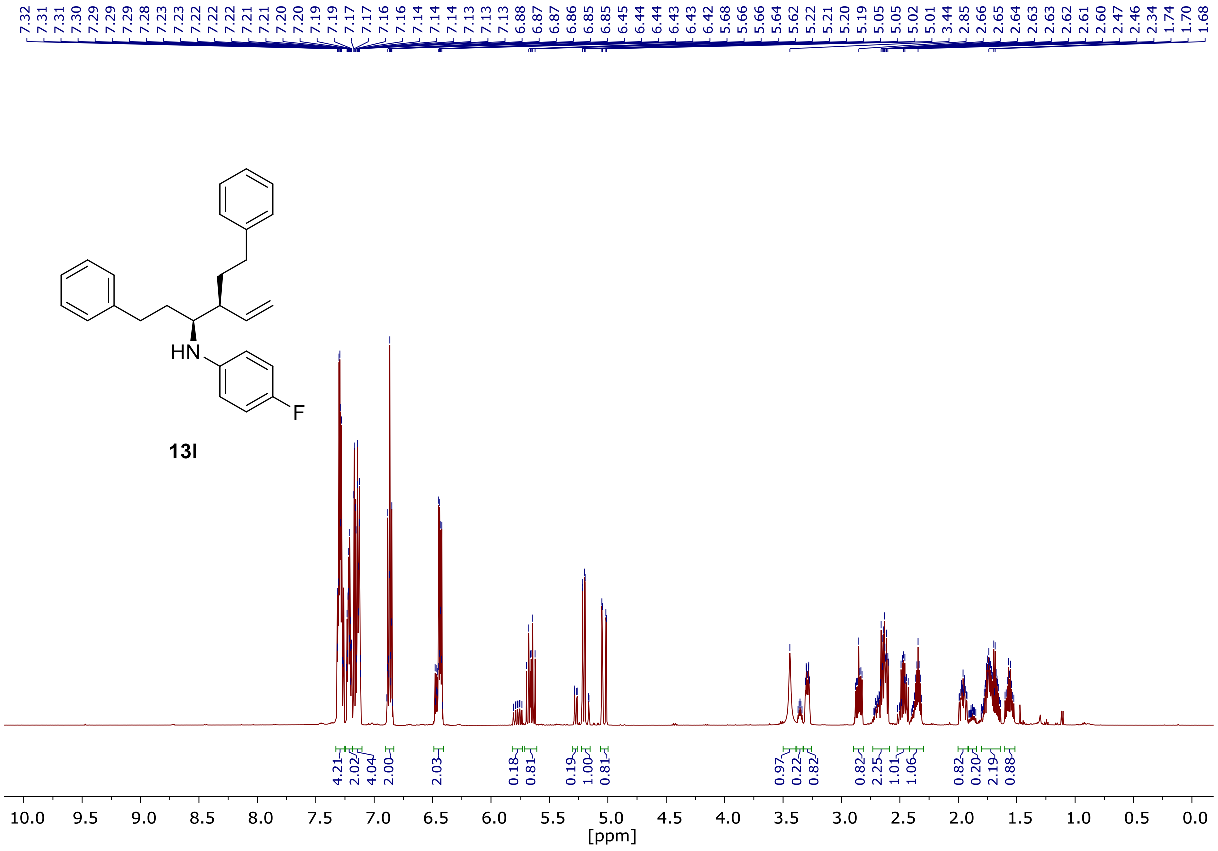
7.32 7.31 7.31 7.30 7.29 7.29 7.29 7.28 7.23 7.23 7.22 7.22 7.22 7.22 7.21 7.21 7.20 7.20 7.19 7.19 7.17 7.17 7.16 7.16 7.14 7.14 7.14 7.13 7.13 7.13 6.88 6.87 6.87 6.86 6.85 6.85 6.45 6.44 6.44 6.43 6.43 6.42 5.68 5.66 5.66 5.64 5.62 5.22 5.21 5.20 5.19 5.05 5.05 5.02 5.01 3.44 2.85 2.66 2.65 2.64 2.63 2.63 2.62 2.61 2.60 2.47 2.46 2.34 1.74 1.70 1.68
HN
F
13l
4.21 2.02 4.04 2.00 2.03 0.18 0.81 0.19 1.00 0.81 0.97 0.22 0.82 0.82 2.25 1.01 1.06 0.82 0.20 2.19 0.88
10.0 9.5 9.0 8.5 8.0 7.5 7.0 6.5 6.0 5.5 5.0 4.5 4.0 3.5 3.0 2.5 2.0 1.5 1.0 0.5 0.0
[ppm]

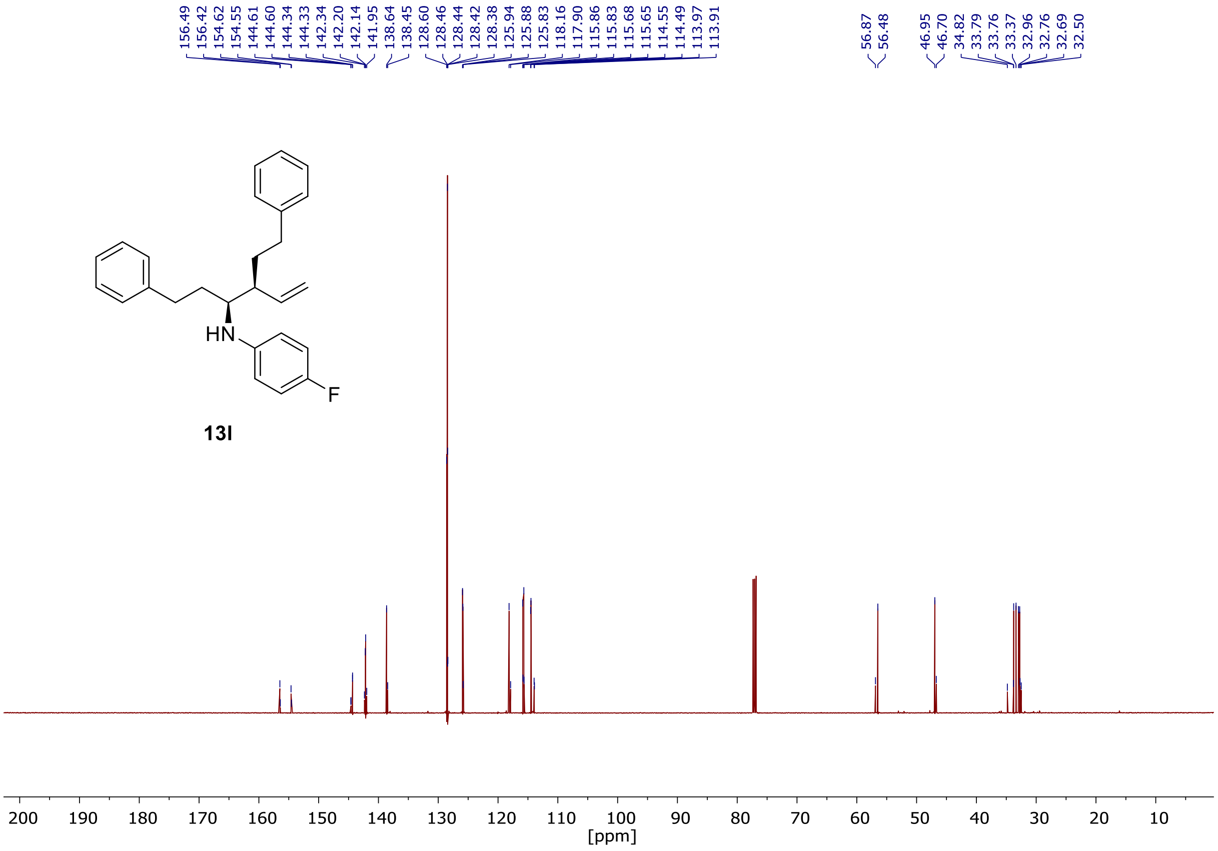
156.49 156.42 154.62 154.55 144.61 144.60 144.34 144.33 142.34 142.20 142.14 141.95 138.64 138.45 128.60 128.46 128.44 128.42 128.38 125.94 125.88 125.83 118.16 117.90 115.86 115.83 115.68 115.65 114.55 114.49 113.97 113.91
56.87 56.48
46.95 46.70 34.82 33.79 33.76 33.37 32.96 32.76 32.69 32.50
HN
F
13l
200 190 180 170 160 150 140 130 120 110 100 90 80 70 60 50 40 30 20 10
[ppm]

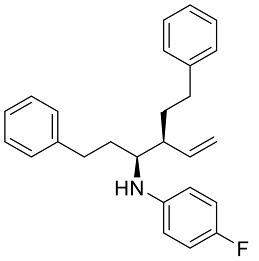
HN
F

13l

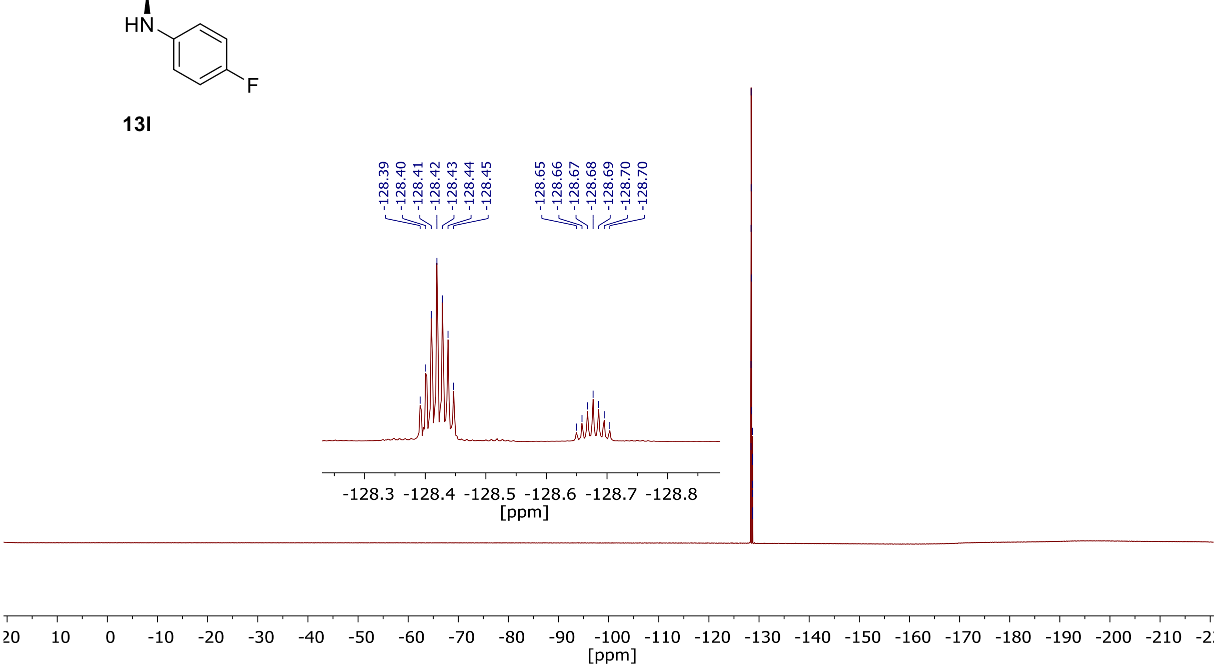
-128.39
-128.40
-128.41
-128.42
-128.43
-128.44
-128.45
-128.65
-128.66
-128.67
-128.68
-128.69
-128.70
-128.70
-128.3 -128.4 -128.5 -128.6 -128.7 -128.8
[ppm]
20 10 0 -10 -20 -30 -40 -50 -60 -70 -80 -90 -100 -110 -120 -130 -140 -150 -160 -170 -180 -190 -200 -210
[ppm]

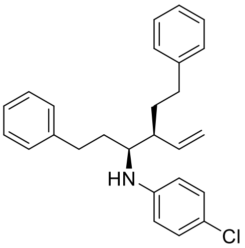
HN
Cl

13m

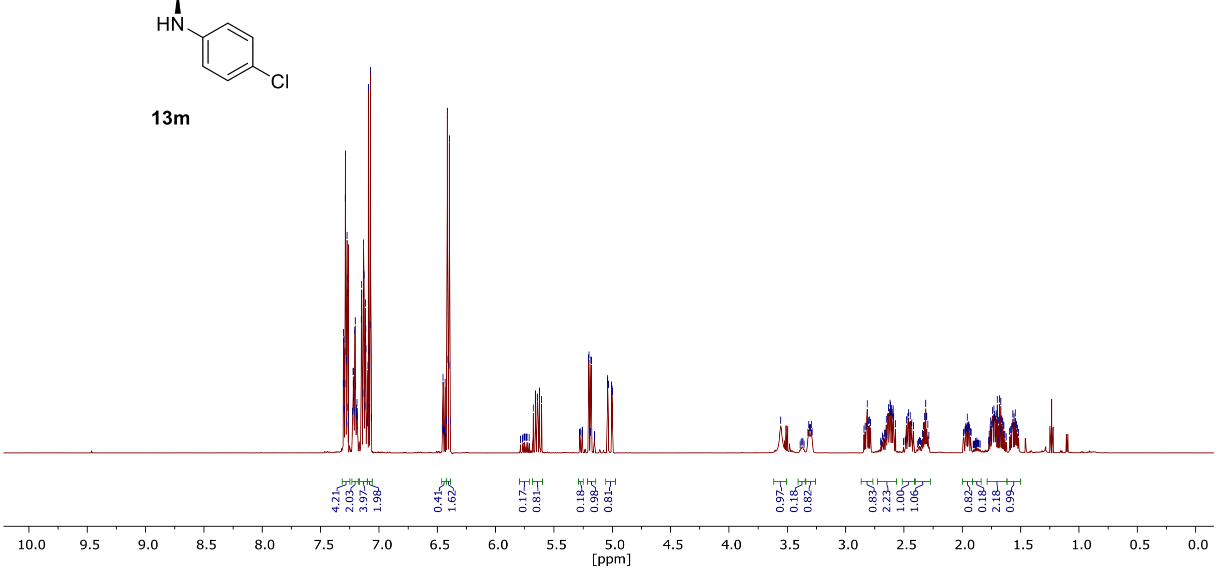
4.21
2.03
3.97
1.98
0.41
1.62
0.17
0.81
0.18
0.98
0.81
0.97
0.18
0.82
0.83
2.23
1.00
1.06
0.82
0.18
2.18
0.99
10.0 9.5 9.0 8.5 8.0 7.5 7.0 6.5 6.0 5.5 5.0 4.5 4.0 3.5 3.0 2.5 2.0 1.5 1.0 0.5 0.0
[ppm]

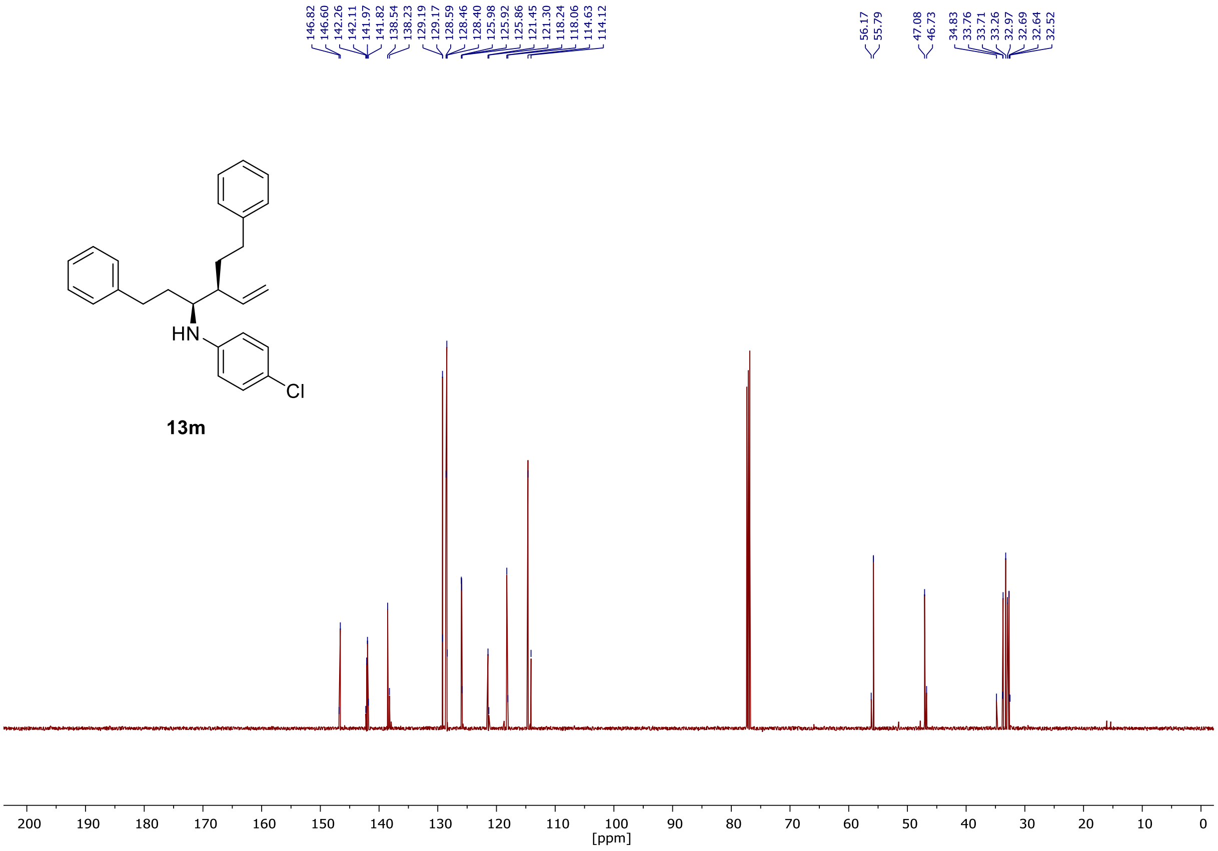
146.82
146.60
142.26
142.11
141.97
141.82
138.54
138.23
129.19
129.17
128.59
128.46
128.40
125.98
125.92
125.86
121.45
121.30
118.24
118.06
114.63
114.12
56.17
55.79
47.08
46.73
34.83
33.76
33.71
33.26
32.97
32.69
32.64
32.52
HN
Cl
13m
200 190 180 170 160 150 140 130 120 110 100 90 80 70 60 50 40 30 20 10 0
[ppm]

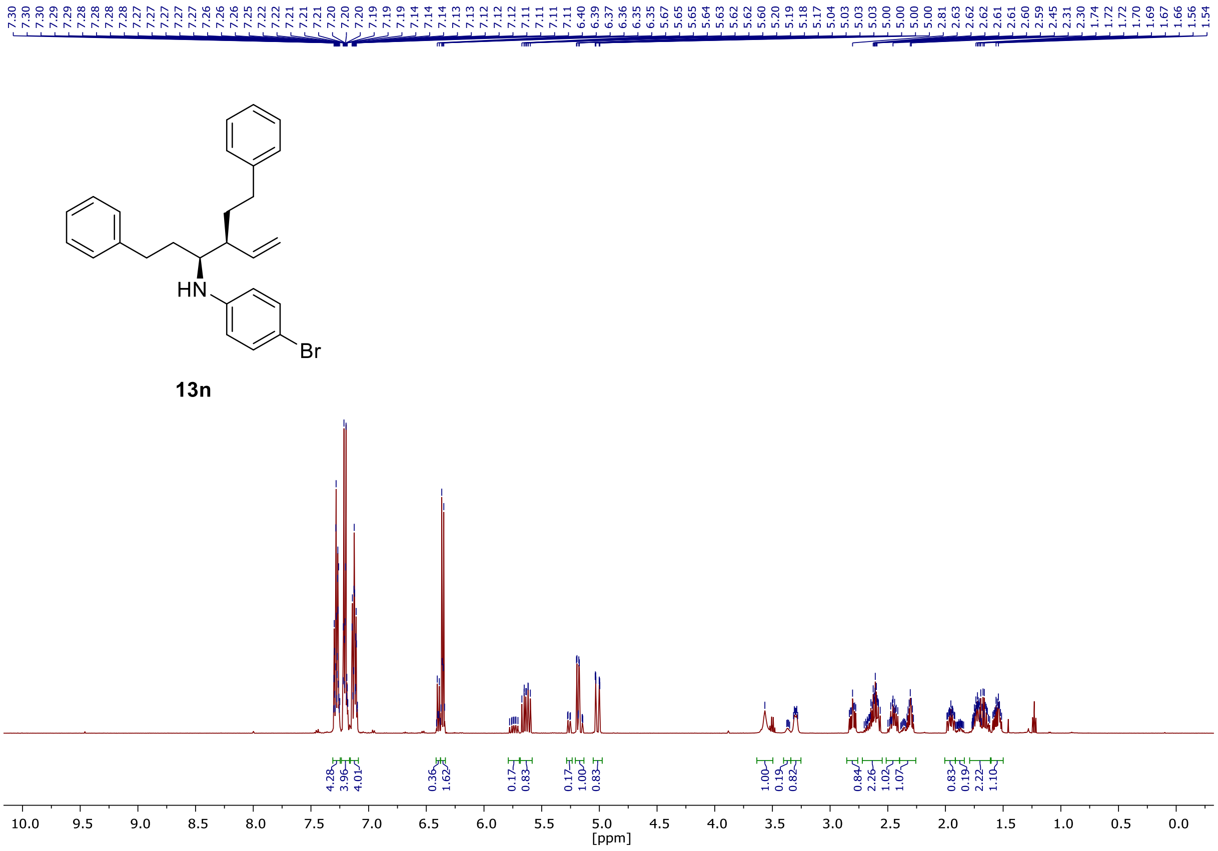
7.30 7.30 7.30 7.29 7.29 7.28 7.28 7.28 7.28 7.27 7.27 7.27 7.27 7.26 7.26 7.26 7.25 7.22 7.22 7.21 7.21 7.21 7.21 7.20 7.20 7.20 7.19 7.19 7.19 7.14 7.14 7.13 7.13 7.12 7.12 7.12 7.11 7.11 7.11 7.11 6.40 6.39 6.37 6.36 6.35 6.35 5.67 5.65 5.65 5.64 5.63 5.62 5.62 5.60 5.20 5.19 5.18 5.17 5.04 5.03 5.03 5.00 5.00 5.00 5.00 2.81 2.63 2.62 2.62 2.61 2.61 2.60 2.59 2.45 2.31 2.30 1.74 1.72 1.72 1.70 1.69 1.67 1.66 1.56 1.54
HN
Br
13n
4.28 3.96 4.01 0.36 1.62 0.17 0.83 0.17 1.00 0.83 1.00 0.19 0.82 0.84 2.26 1.02 1.07 0.83 0.19 2.22 1.10
10.0 9.5 9.0 8.5 8.0 7.5 7.0 6.5 6.0 5.5 5.0 4.5 4.0 3.5 3.0 2.5 2.0 1.5 1.0 0.5 0.0
[ppm]

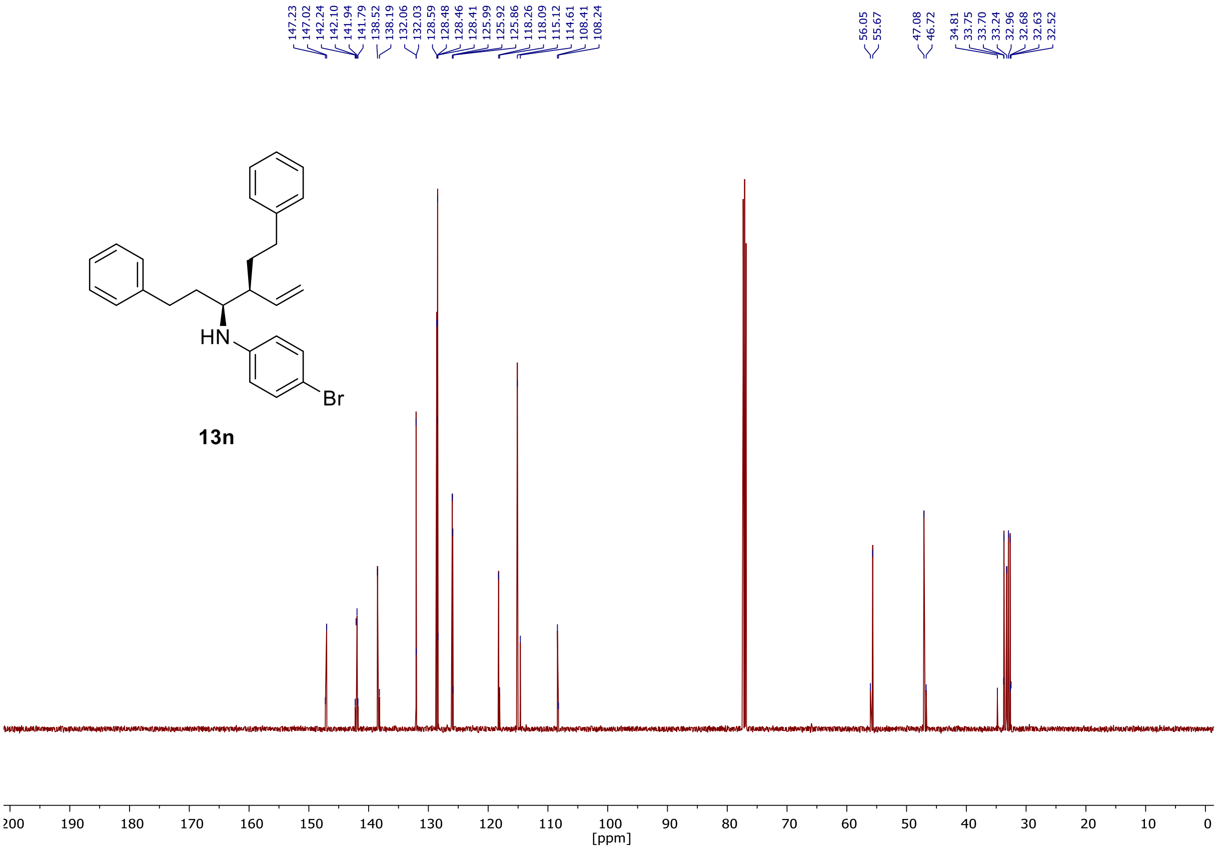
147.23
147.02
142.24
142.10
141.94
141.79
138.52
138.19
132.06
132.03
128.59
128.48
128.46
128.41
125.99
125.92
125.86
118.26
118.09
115.12
114.61
108.41
108.24
56.05
55.67
47.08
46.72
34.81
33.75
33.70
33.24
32.96
32.68
32.63
32.52
HN
Br
200 190 180 170 160 150 140 130 120 110 100 90 80 70 60 50 40 30 20 10 0
[ppm]

13n

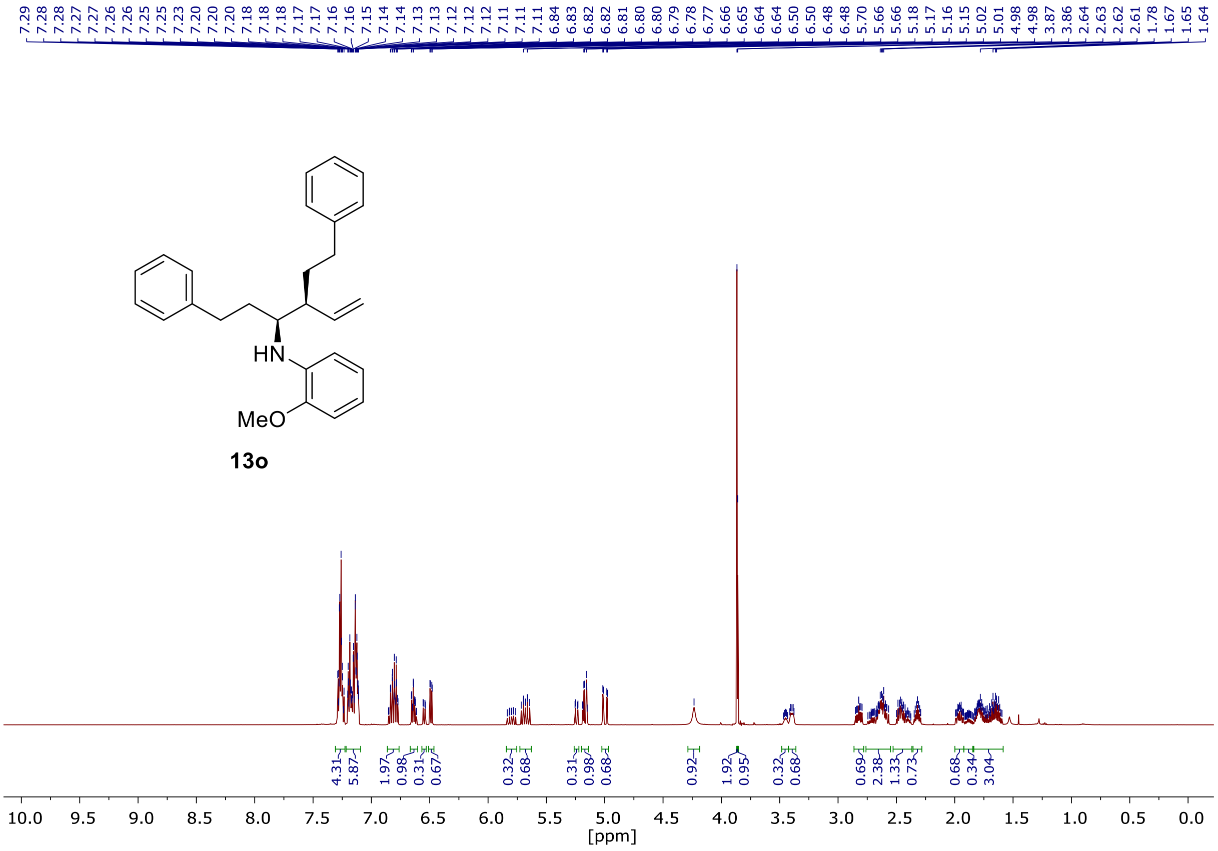
7.29
7.28
7.28
7.27
7.26
7.26
7.25
7.25
7.23
7.20
7.20
7.20
7.18
7.18
7.17
7.17
7.16
7.16
7.15
7.14
7.14
7.13
7.13
7.12
7.12
7.12
7.11
7.11
7.11
6.84
6.83
6.82
6.82
6.81
6.80
6.80
6.79
6.78
6.77
6.66
6.65
6.64
6.64
6.50
6.50
6.48
6.48
5.70
5.66
5.66
5.18
5.17
5.16
5.15
5.02
5.01
4.98
4.98
3.87
3.86
2.64
2.63
2.62
2.61
1.78
1.67
1.65
1.64
HN
MeO
4.31
5.87
1.97
0.98
0.31
0.67
0.32
0.68
0.31
0.98
0.68
0.92
1.92
0.95
0.32
0.68
0.69
2.38
1.33
0.73
0.68
0.34
3.04
10.0 9.5 9.0 8.5 8.0 7.5 7.0 6.5 6.0 5.5 5.0 4.5 4.0 3.5 3.0 2.5 2.0 1.5 1.0 0.5 0.0
[ppm]

13o

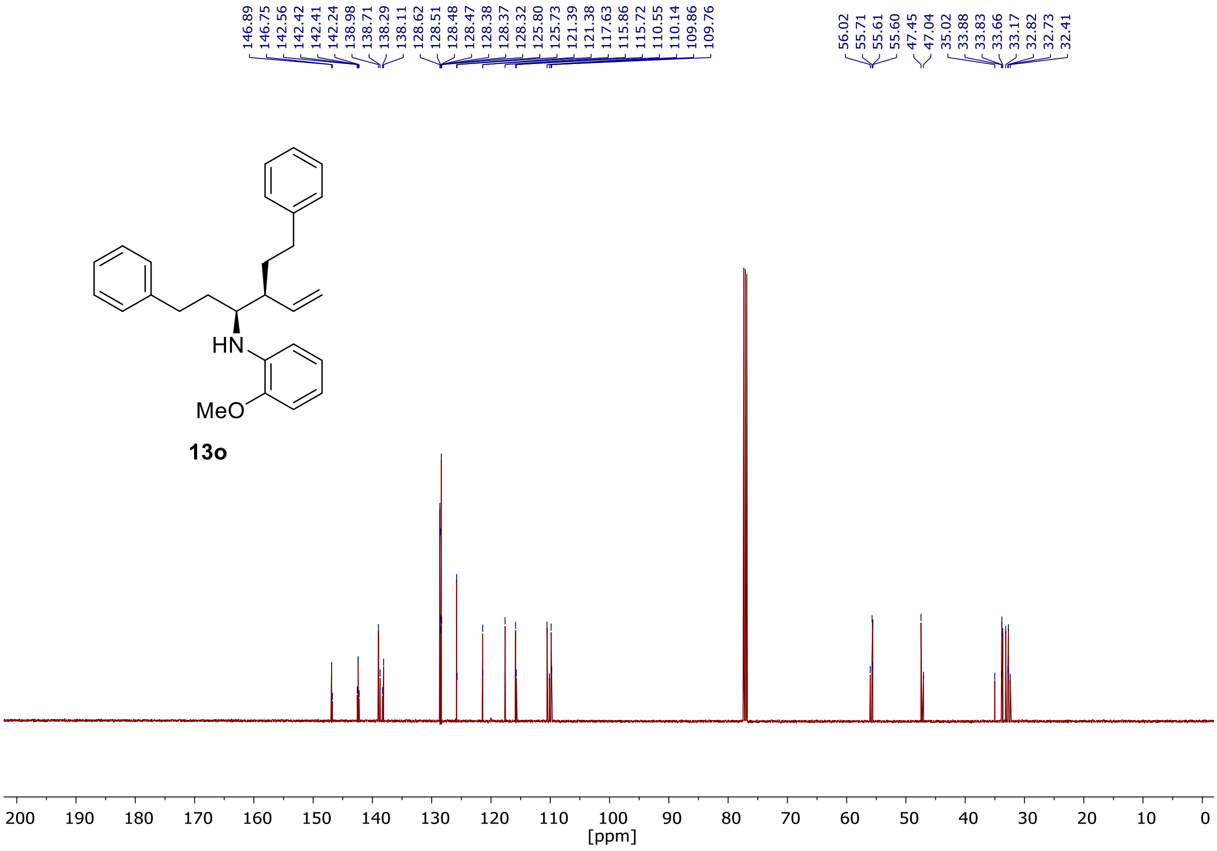
146.89
146.75
142.56
142.42
142.41
142.24
138.98
138.71
138.29
138.11
128.62
128.51
128.48
128.47
128.38
128.37
128.32
125.80
125.73
121.39
121.38
117.63
115.86
115.72
110.55
110.14
109.86
109.76
56.02
55.71
55.61
55.60
47.45
47.04
35.02
33.88
33.83
33.66
33.17
32.82
32.73
32.41
HN
MeO
200 190 180 170 160 150 140 130 120 110 100 90 80 70 60 50 40 30 20 10 0
[ppm]

13o

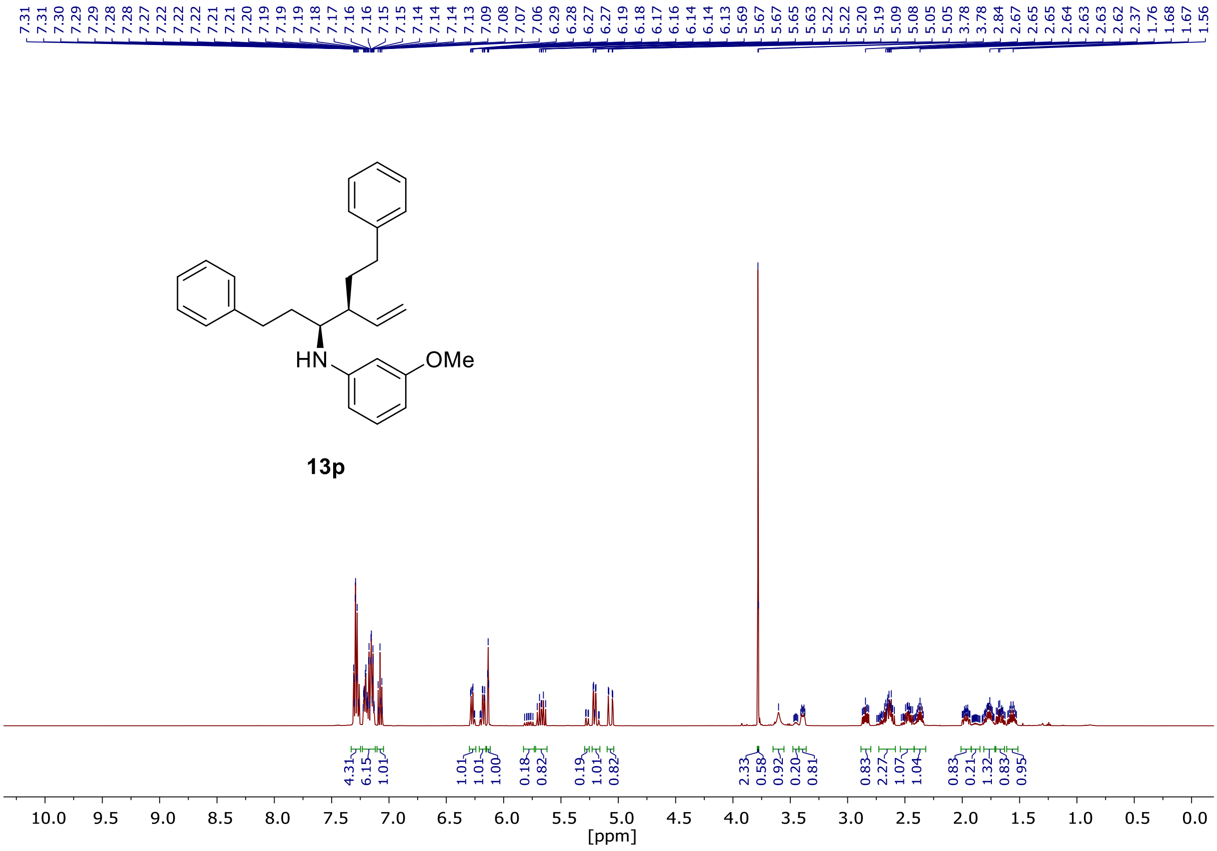
7.31
7.31
7.30
7.29
7.29
7.28
7.28
7.27
7.22
7.22
7.22
7.21
7.21
7.20
7.19
7.19
7.19
7.18
7.17
7.16
7.16
7.15
7.15
7.14
7.14
7.14
7.13
7.09
7.08
7.07
7.06
6.29
6.28
6.27
6.27
6.19
6.18
6.17
6.16
6.14
6.14
6.13
5.69
5.67
5.67
5.65
5.63
5.22
5.22
5.20
5.19
5.09
5.08
5.05
5.05
3.78
3.78
2.84
2.67
2.65
2.65
2.64
2.63
2.63
2.62
2.37
1.76
1.68
1.67
1.56
HN
OMe
4.31
6.15
1.01
1.01
1.01
1.00
0.18
0.82
0.19
1.01
0.82
2.33
0.58
0.92
0.20
0.81
0.83
2.27
1.07
1.04
0.83
0.21
1.32
0.83
0.95
10.0 9.5 9.0 8.5 8.0 7.5 7.0 6.5 6.0 5.5 5.0 4.5 4.0 3.5 3.0 2.5 2.0 1.5 1.0 0.5 0.0
[ppm]

13p

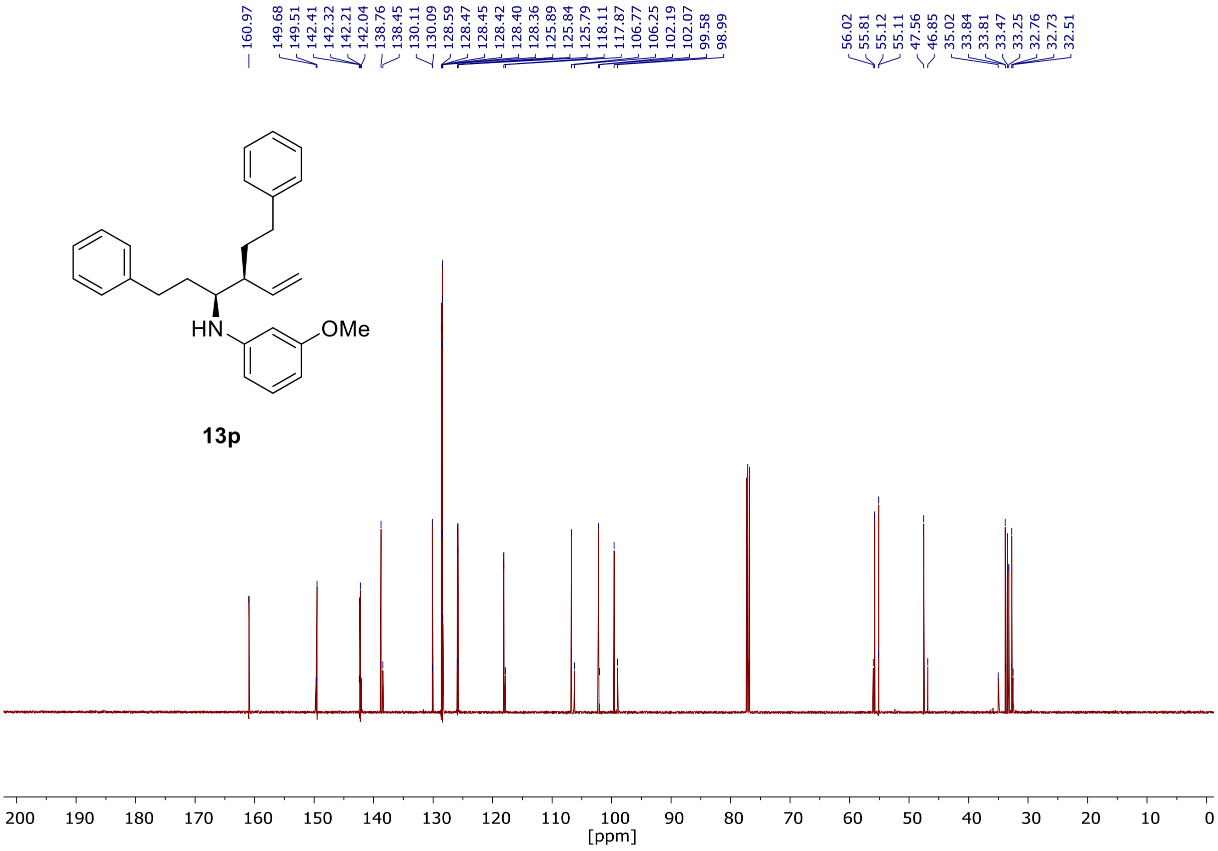

13p

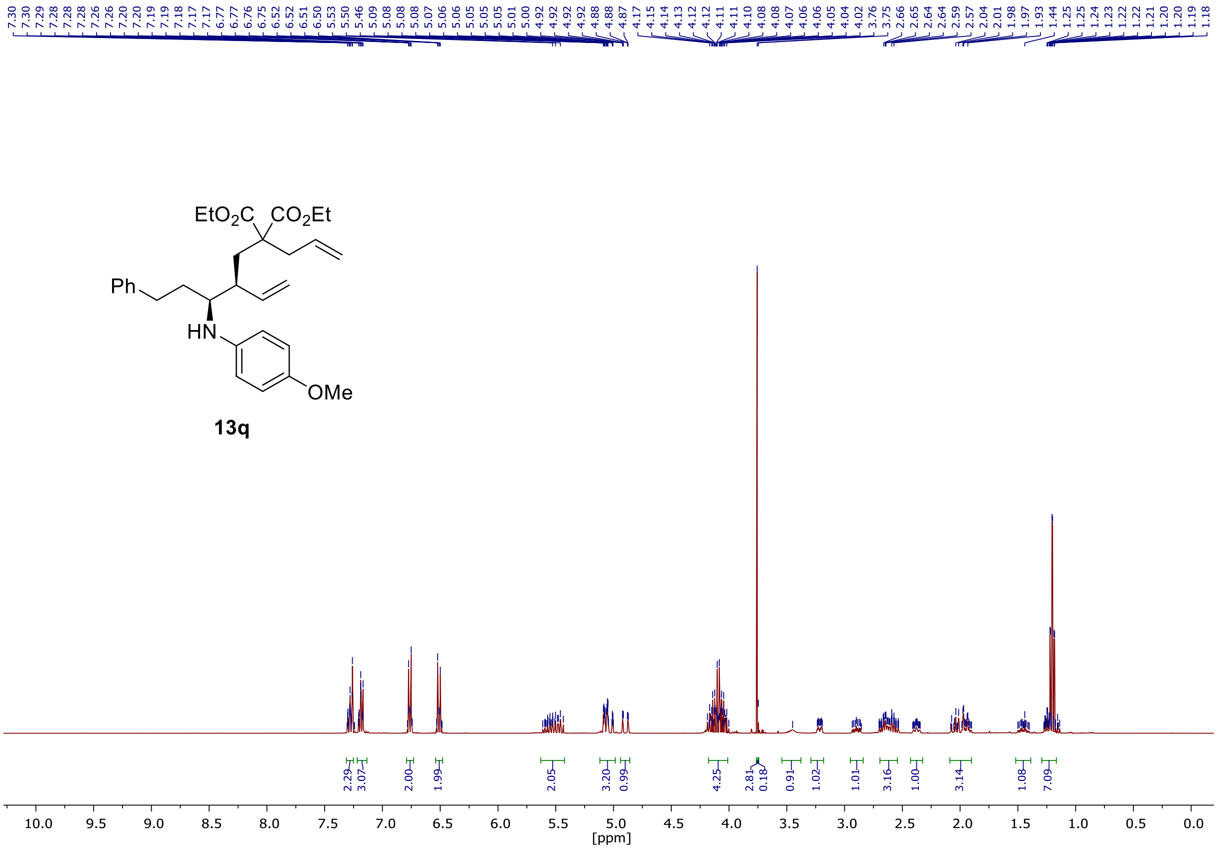

13q

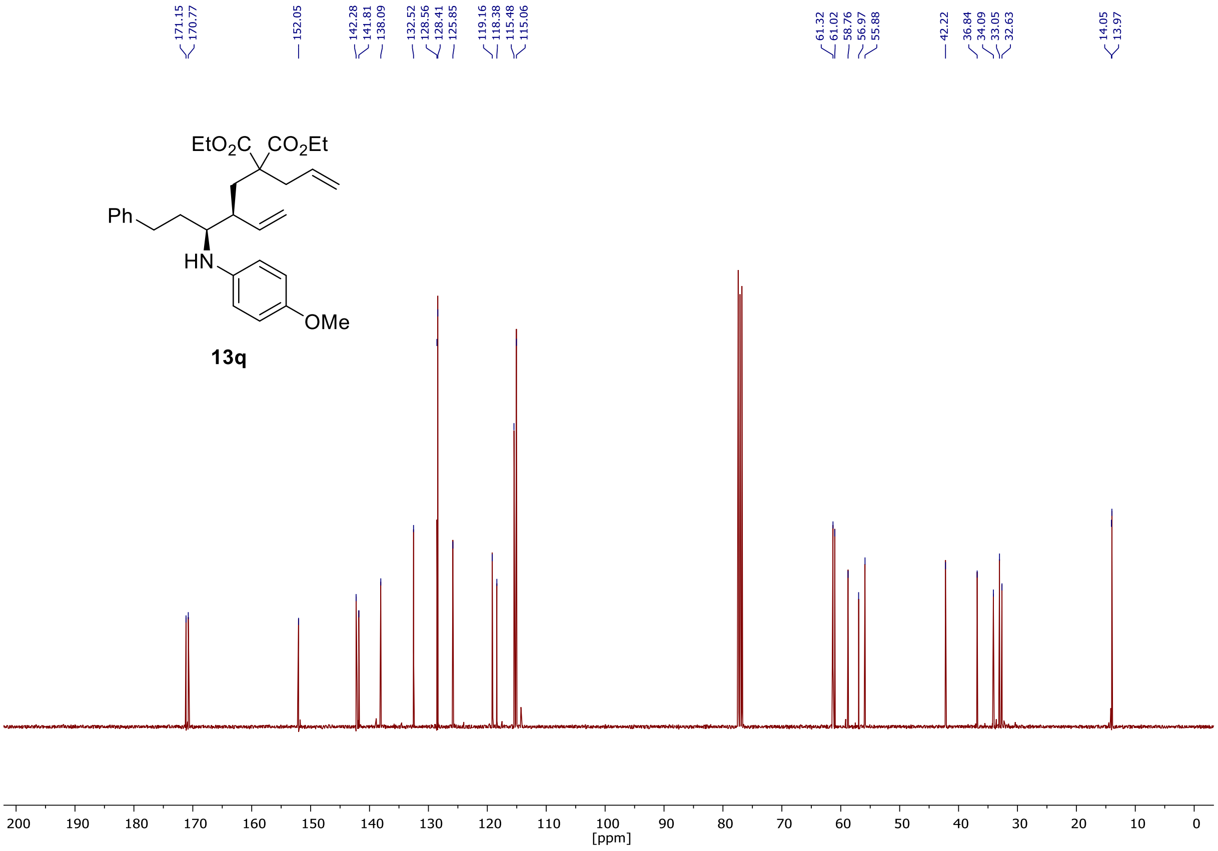
171.15
170.77
152.05
142.28
141.81
138.09
132.52
128.56
128.41
125.85
119.16
118.38
115.48
115.06
61.32
61.02
58.76
56.97
55.88
42.22
36.84
34.09
33.05
32.63
14.05
13.97
EtO2C
CO2Et
Ph
HN
OMe
200 190 180 170 160 150 140 130 120 110 100 90 80 70 60 50 40 30 20 10 0
[ppm]

13q

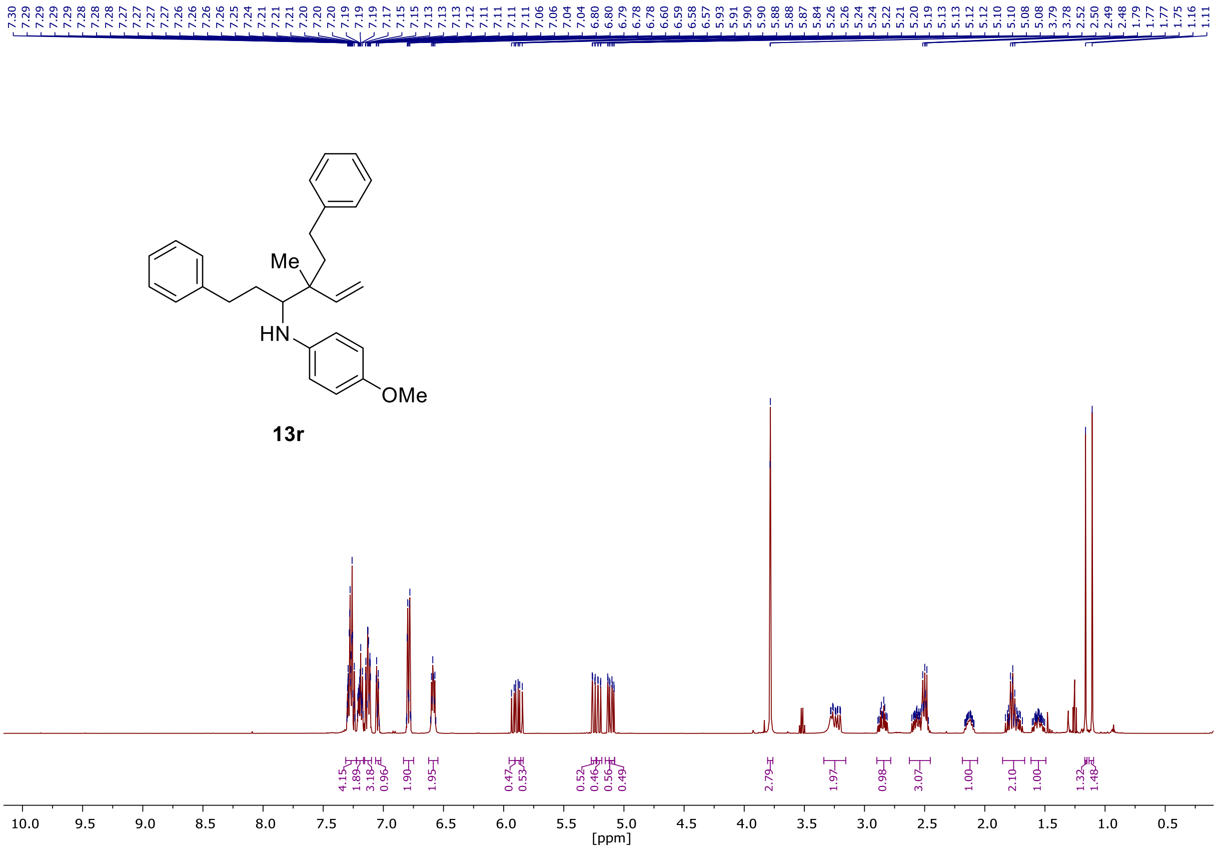
7.30 7.29 7.29 7.29 7.29 7.28 7.28 7.28 7.27 7.27 7.27 7.26 7.26 7.26 7.26 7.25 7.24 7.21 7.21 7.21 7.20 7.20 7.19 7.19 7.19 7.17 7.15 7.15 7.13 7.13 7.12 7.11 7.11 7.11 7.06 7.06 7.04 7.04 6.80 6.80 6.79 6.78 6.78 6.60 6.59 6.58 6.57 5.93 5.91 5.90 5.90 5.88 5.87 5.84 5.26 5.26 5.24 5.24 5.22 5.21 5.20 5.19 5.13 5.13 5.12 5.12 5.10 5.08 5.08 3.79 3.78 2.52 2.50 2.49 2.48 1.79 1.77 1.77 1.75 1.16 1.11
Me
HN
OMe
4.15
1.89
3.18
0.96
1.90
1.95
0.47
0.53
0.52
0.46
0.56
0.49
2.79
1.97
0.98
3.07
1.00
2.10
1.00
1.32
1.48
10.0 9.5 9.0 8.5 8.0 7.5 7.0 6.5 6.0 5.5 5.0 4.5 4.0 3.5 3.0 2.5 2.0 1.5 1.0 0.5
[ppm]

13r

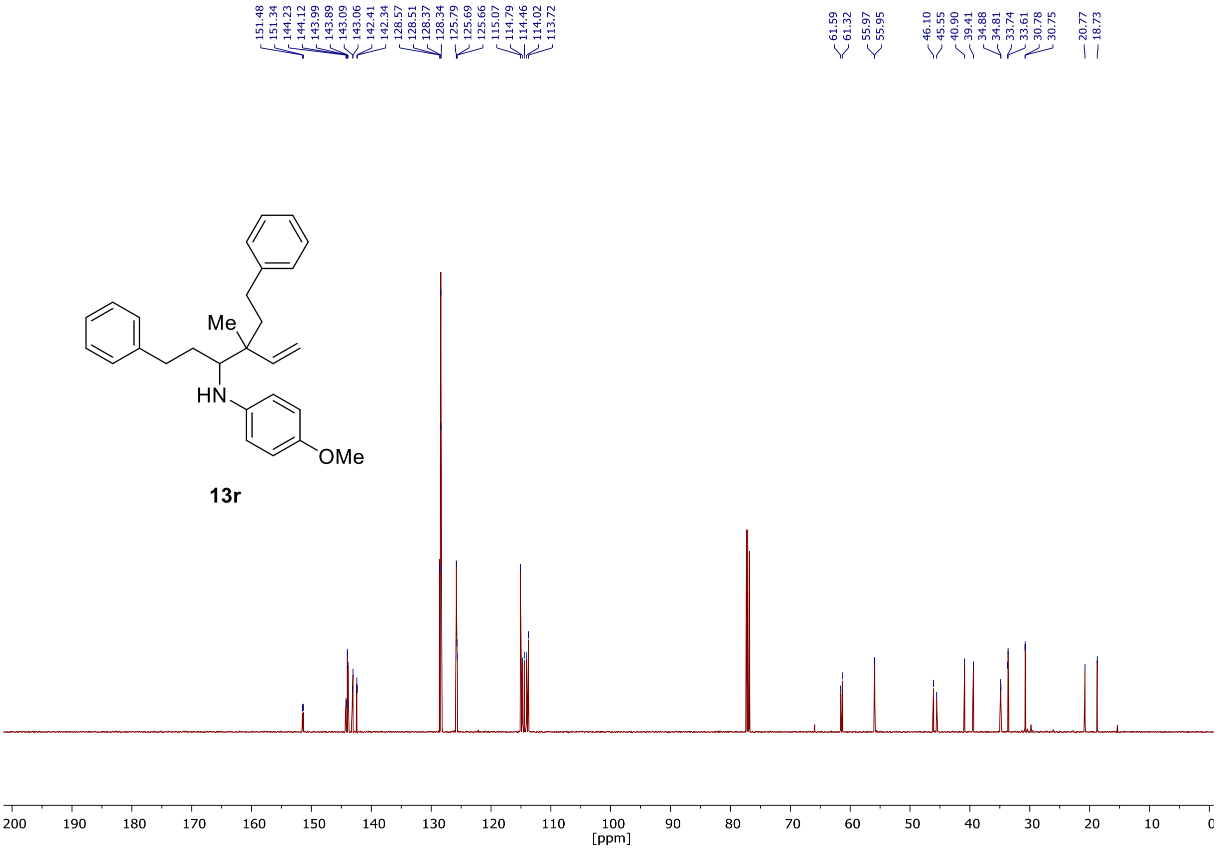
151.48
151.34
144.23
144.12
143.99
143.89
143.09
143.06
142.41
142.34
128.57
128.51
128.37
128.34
125.79
125.69
125.66
115.07
114.79
114.46
114.02
113.72
61.59
61.32
55.97
55.95
46.10
45.55
40.90
39.41
34.88
34.81
33.74
33.61
30.78
30.75
20.77
18.73
Me
HN
OMe
200 190 180 170 160 150 140 130 120 110 100 90 80 70 60 50 40 30 20 10 0
[ppm]

13r

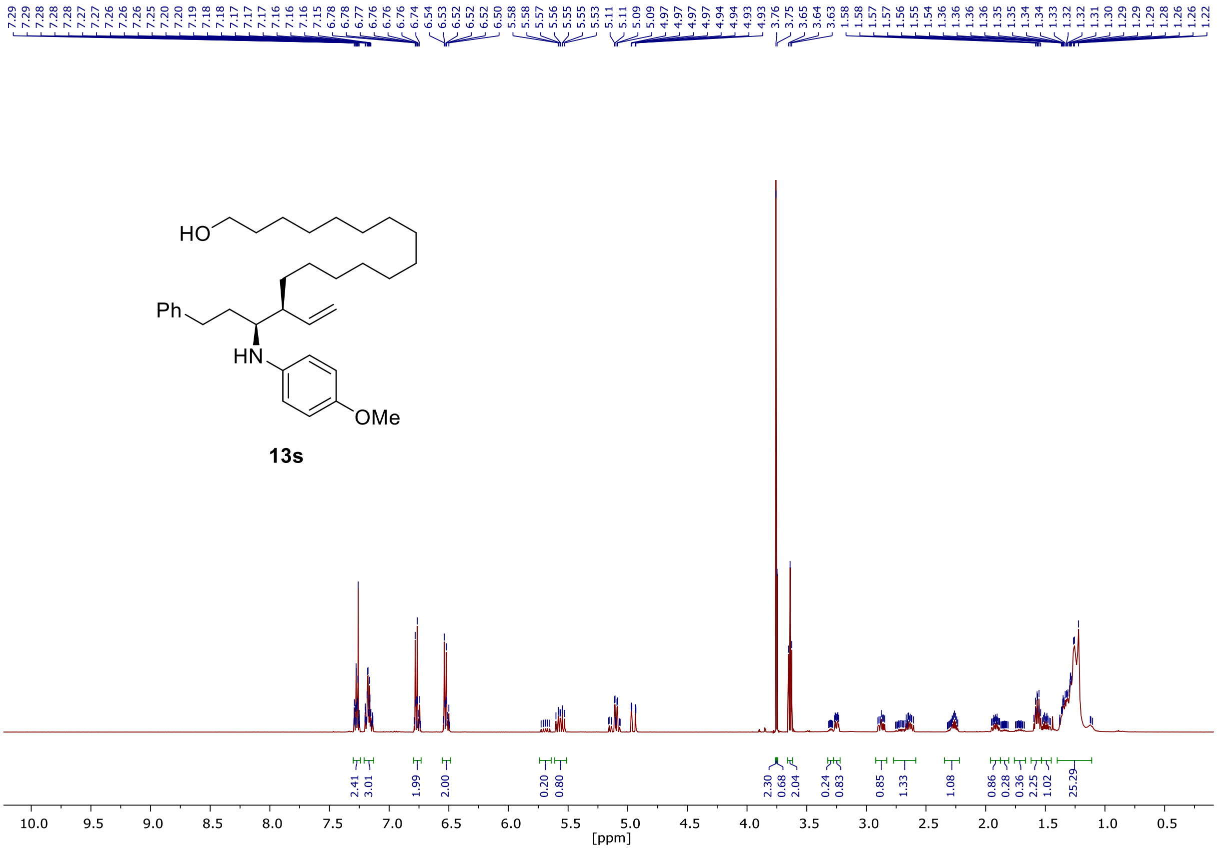
7.29
7.29
7.28
7.28
7.28
7.27
7.26
7.26
7.25
7.20
7.20
7.19
7.18
7.18
7.17
7.17
7.16
7.16
7.15
6.78
6.77
6.76
6.76
6.74
6.74
6.54
6.53
6.52
6.52
6.50
5.58
5.58
5.57
5.56
5.55
5.55
5.53
5.11
5.11
5.09
5.09
4.97
4.97
4.97
4.94
4.94
4.93
4.93
3.76
3.75
3.65
3.64
3.63
1.58
1.58
1.57
1.56
1.55
1.54
1.36
1.36
1.36
1.35
1.35
1.34
1.34
1.33
1.32
1.32
1.31
1.30
1.29
1.29
1.28
1.26
1.22
HO
Ph
HN
OMe
2.41
3.01
1.99
2.00
0.20
0.80
2.30
0.68
2.04
0.24
0.83
0.85
1.33
1.08
0.86
0.28
0.36
2.25
1.02
25.29
10.0 9.5 9.0 8.5 8.0 7.5 7.0 6.5 6.0 5.5 5.0 4.5 4.0 3.5 3.0 2.5 2.0 1.5 1.0 0.5
[ppm]

13s

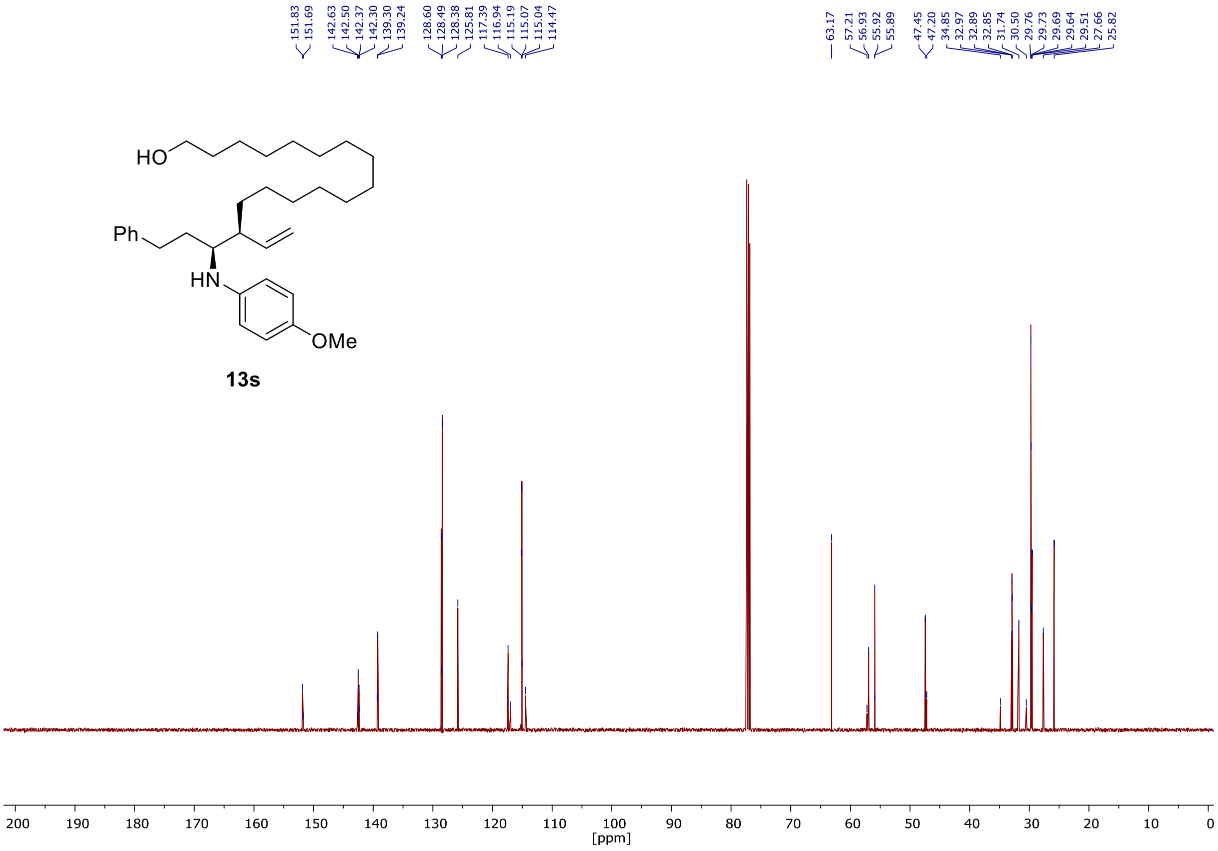
151.83
151.69
142.63
142.50
142.37
142.30
139.30
139.24
128.60
128.49
128.38
125.81
117.39
116.94
115.19
115.07
115.04
114.47
63.17
57.21
56.93
55.92
55.89
47.45
47.20
34.85
32.97
32.89
32.85
31.74
30.50
29.76
29.73
29.69
29.64
29.51
27.66
25.82
HO
Ph
HN
OMe
13s
200 190 180 170 160 150 140 130 120 110 100 90 80 70 60 50 40 30 20 10 0
[ppm]

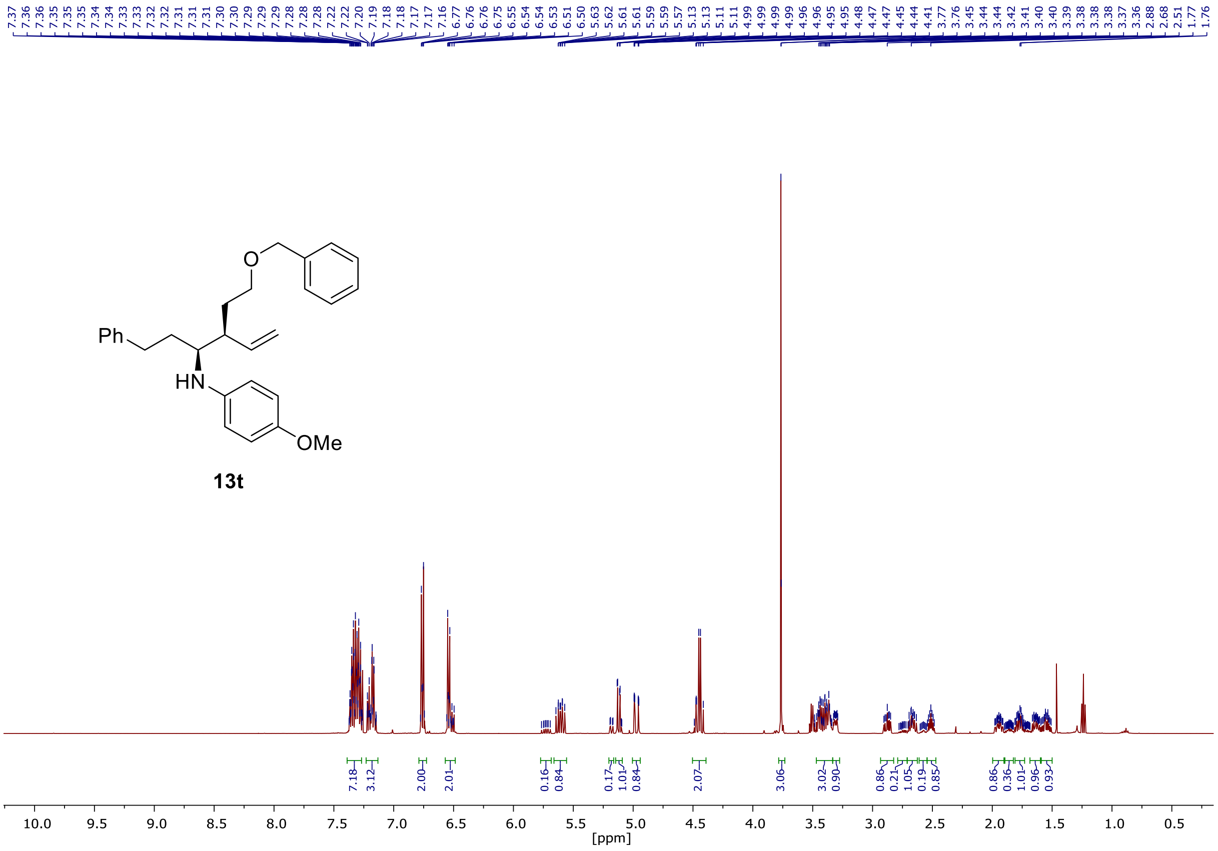
7.37 7.36 7.35 7.35 7.34 7.34 7.33 7.33 7.32 7.32 7.31 7.31 7.31 7.30 7.29 7.29 7.28 7.28 7.22 7.22 7.20 7.19 7.18 7.18 7.17 7.16 6.77 6.76 6.76 6.75 6.55 6.54 6.54 6.53 6.51 6.50 5.63 5.62 5.61 5.61 5.59 5.59 5.57 5.13 5.13 5.11 5.11 4.99 4.99 4.99 4.99 4.96 4.96 4.95 4.95 4.48 4.47 4.47 4.45 4.44 4.41 3.77 3.76 3.45 3.44 3.44 3.42 3.41 3.40 3.39 3.38 3.38 3.38 3.37 3.36 2.88 2.68 2.51 1.77 1.76
O
Ph
HN
OMe
13t
7.18 3.12 2.00 2.01 0.16 0.84 0.17 1.01 0.84 2.07 3.06 3.02 0.90 0.86 0.21 1.05 0.19 0.85 0.86 0.36 1.01 0.96 0.93
10.0 9.5 9.0 8.5 8.0 7.5 7.0 6.5 6.0 5.5 5.0 4.5 4.0 3.5 3.0 2.5 2.0 1.5 1.0 0.5
[ppm]

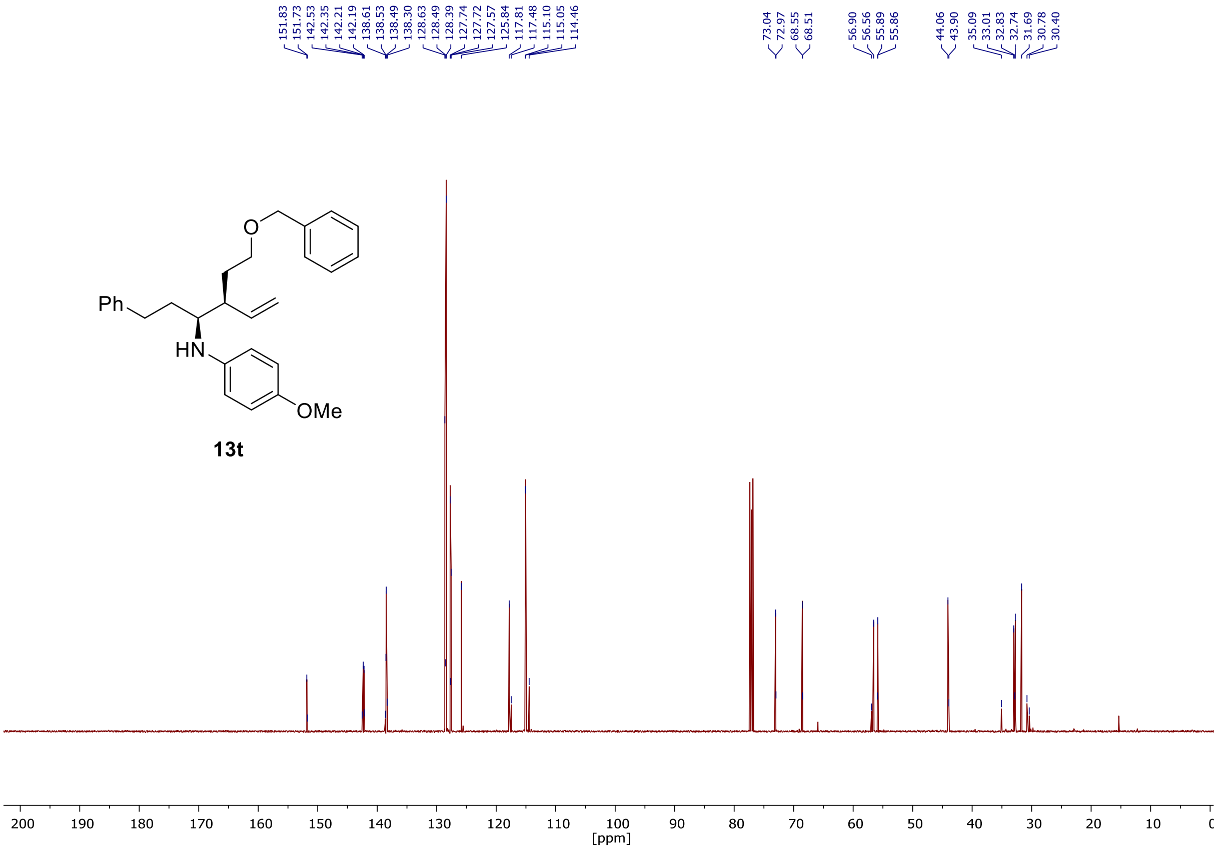
151.83 151.73 142.53 142.35 142.21 142.19 138.61 138.53 138.49 138.30 128.63 128.49 128.39 127.74 127.72 127.57 125.84 117.81 117.48 115.10 115.05 114.46
73.04 72.97 68.55 68.51
56.90 56.56 55.89 55.86
44.06 43.90 35.09 33.01 32.83 32.74 31.69 30.78 30.40
Ph
O
HN
OMe
13t
[ppm]

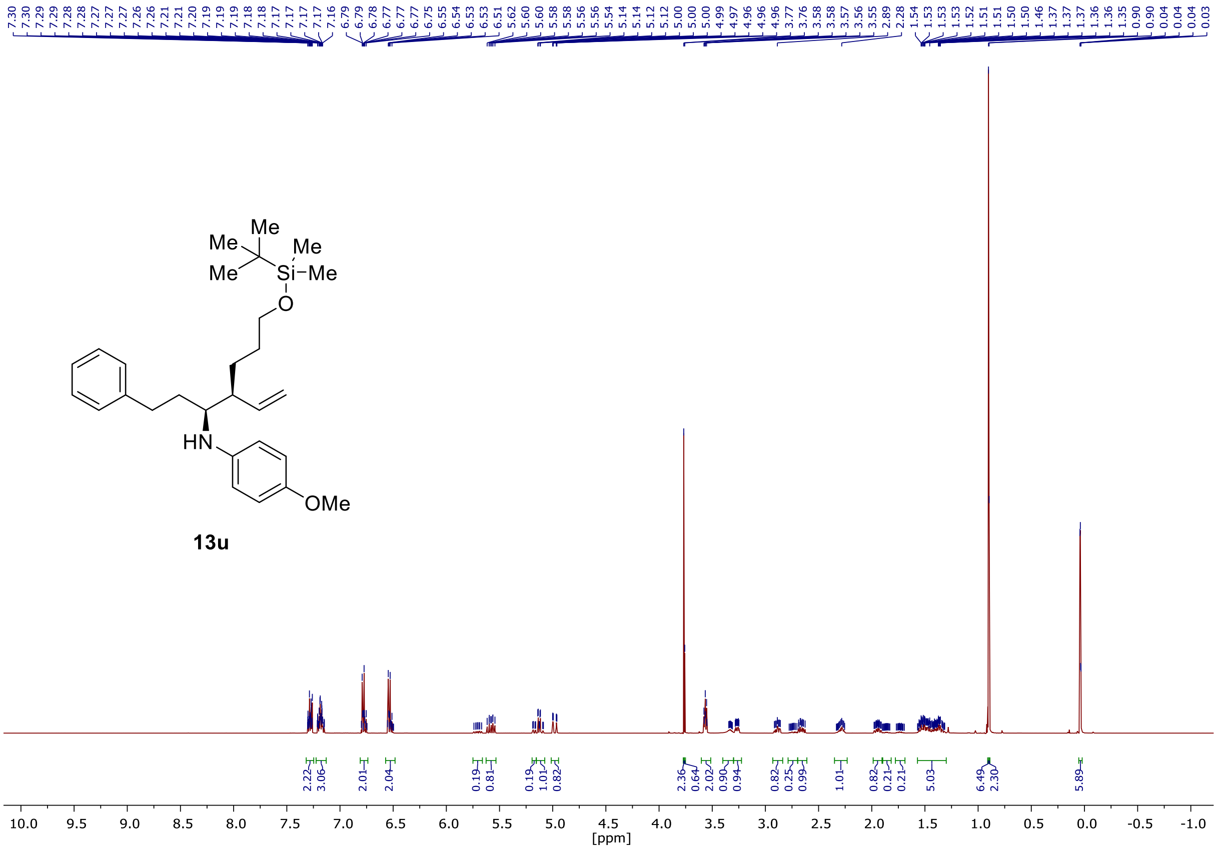
7.30 7.30 7.29 7.29 7.28 7.27 7.27 7.27 7.26 7.21 7.21 7.20 7.19 7.19 7.19 7.18 7.18 7.17 7.17 7.17 7.16 6.79 6.78 6.77 6.77 6.75 6.55 6.54 6.53 6.53 6.51 5.62 5.60 5.60 5.58 5.58 5.56 5.56 5.54 5.14 5.14 5.12 5.12 5.00 5.00 4.99 4.97 4.96 4.96 3.77 3.76 3.58 3.57 3.56 3.55 2.89 2.28 1.54 1.53 1.53 1.52 1.51 1.51 1.50 1.50 1.46 1.37 1.37 1.36 1.35 0.90 0.90 0.04 0.04 0.04 0.03
Me
Me
Me
Me
Si–Me
O
HN
OMe
13u
2.22 3.06 2.01 2.04 0.19 0.81 0.19 1.01 0.82 2.36 0.64 2.02 0.90 0.94 0.82 0.25 0.99 1.01 0.82 0.21 0.21 5.03 6.49 2.30 5.89
[ppm]

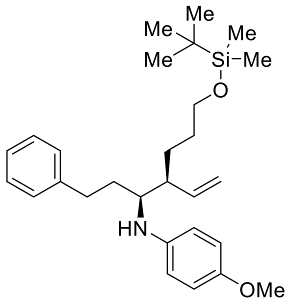
Me
Me
Me
Me
Si–Me
O
HN
OMe

13u

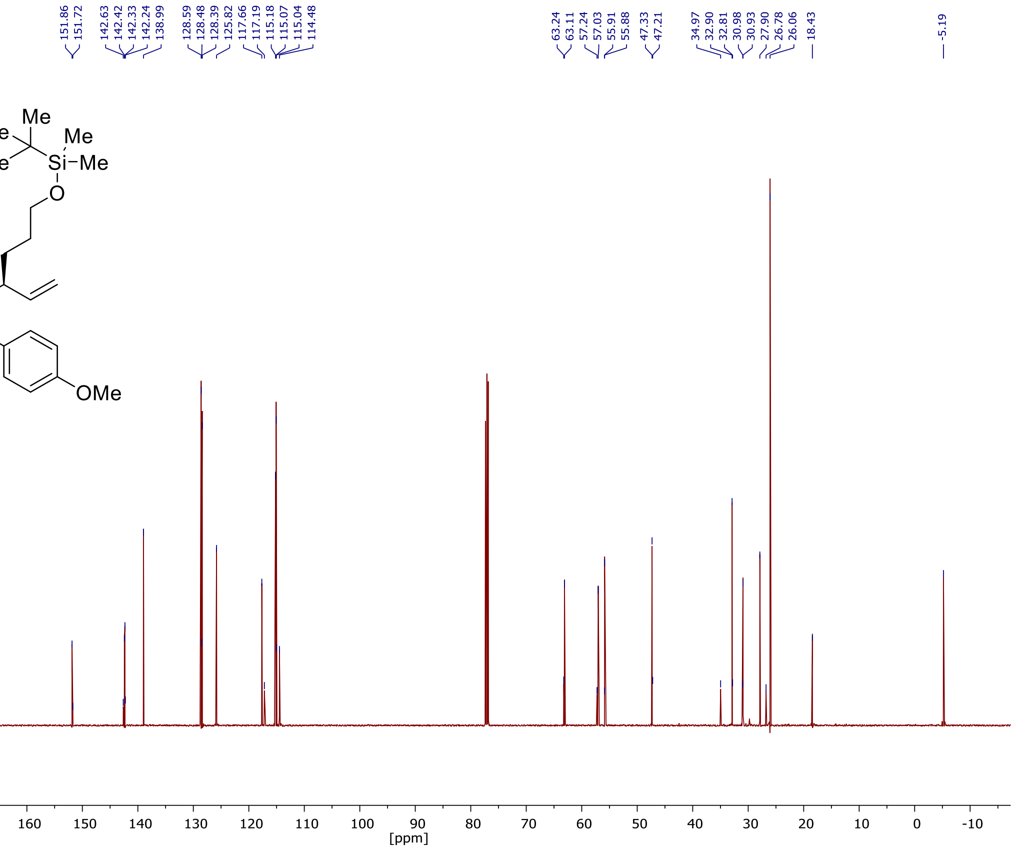
151.86
151.72
142.63
142.42
142.33
142.24
138.99
128.59
128.48
128.39
125.82
117.66
117.19
115.18
115.07
115.04
114.48
63.24
63.11
57.24
57.03
55.91
55.88
47.33
47.21
34.97
32.90
32.81
30.98
30.93
27.90
26.78
26.06
18.43
-5.19
200 190 180 170 160 150 140 130 120 110 100 90 80 70 60 50 40 30 20 10 0 -10
[ppm]

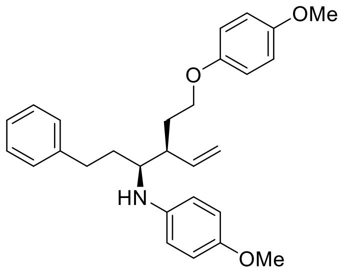
OMe
O
HN
OMe

13v

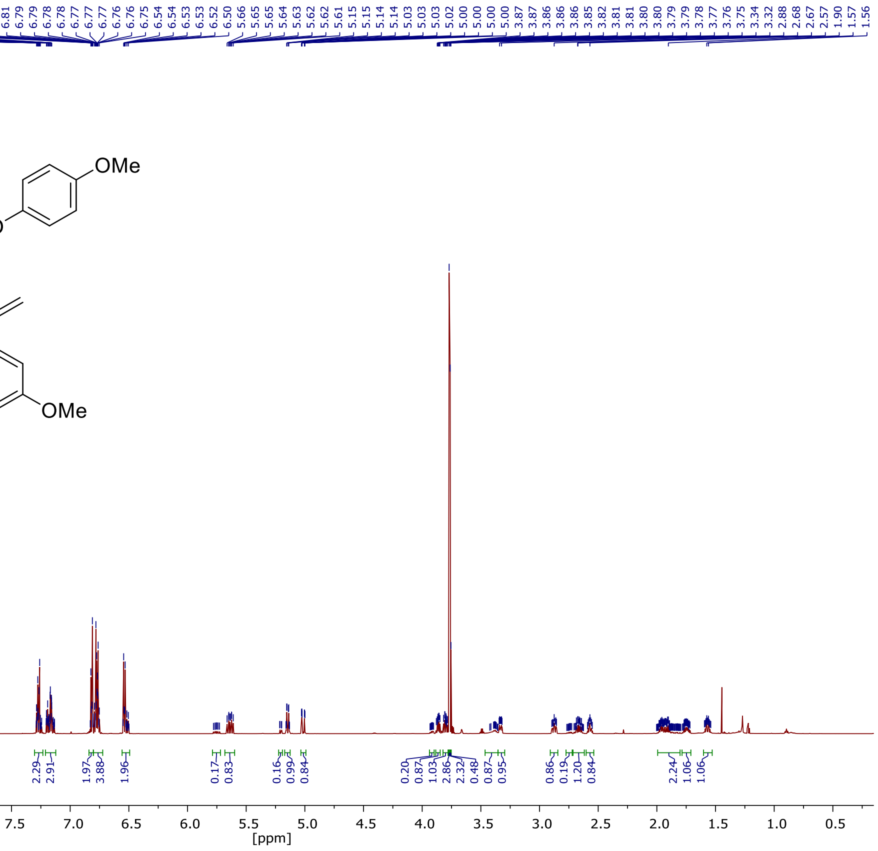
7.29 7.28 7.28 7.27 7.27 7.26 7.25 7.20 7.20 7.19 7.19 7.18 7.18 7.17 7.16 7.16 7.16 6.82 6.82 6.81 6.81 6.79 6.79 6.78 6.78 6.77 6.77 6.76 6.75 6.54 6.54 6.53 6.53 6.52 6.50 5.66 5.65 5.65 5.64 5.63 5.62 5.61 5.15 5.15 5.14 5.14 5.03 5.03 5.03 5.02 5.00 5.00 5.00 3.87 3.87 3.86 3.86 3.85 3.82 3.81 3.81 3.80 3.80 3.79 3.78 3.77 3.76 3.75 3.34 3.32 2.88 2.68 2.67 2.57 1.90 1.57 1.56
2.29 2.91 1.97 3.88 1.96 0.17 0.83 0.16 0.99 0.84 0.20 0.87 1.03 2.86 2.32 0.48 0.87 0.95 0.86 0.19 1.20 0.84 2.24 1.06 1.06
10.0 9.5 9.0 8.5 8.0 7.5 7.0 6.5 6.0 5.5 5.0 4.5 4.0 3.5 3.0 2.5 2.0 1.5 1.0 0.5
[ppm]

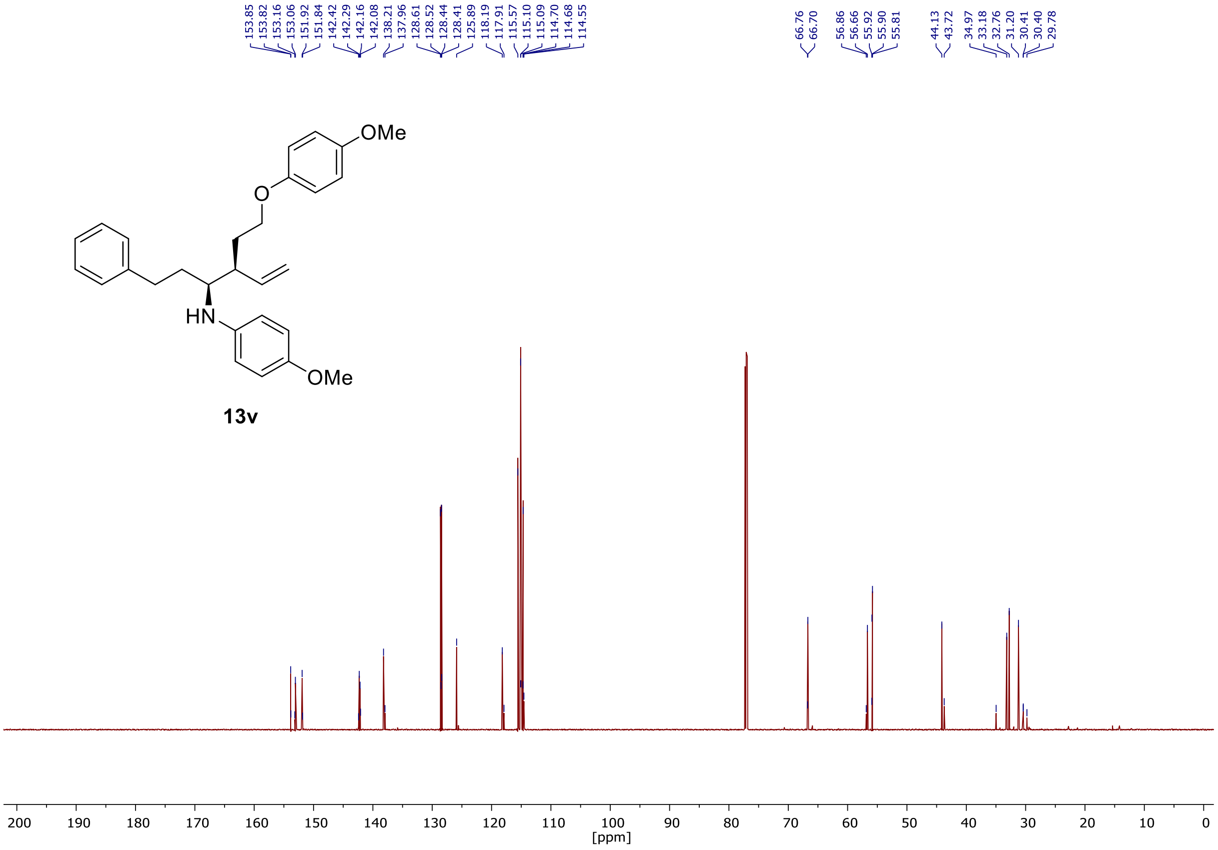
153.85
153.82
153.16
153.06
151.92
151.84
142.42
142.29
142.16
142.08
138.21
137.96
128.61
128.52
128.44
128.41
125.89
118.19
117.91
115.57
115.10
115.09
114.70
114.68
114.55
66.76
66.70
56.86
56.66
55.92
55.90
55.81
44.13
43.72
34.97
33.18
32.76
31.20
30.41
30.40
29.78
OMe
O
HN
OMe
[ppm]

13v

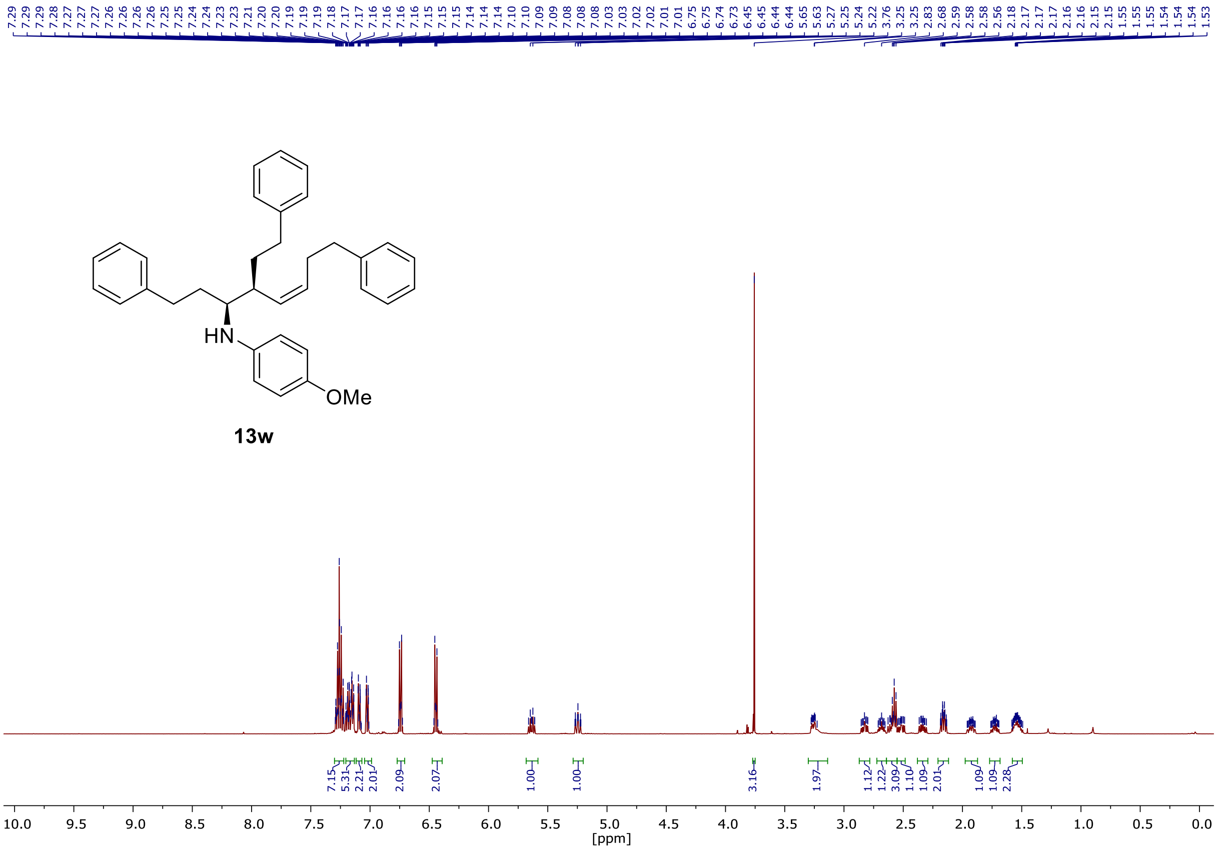
7.29
7.29
7.29
7.28
7.27
7.27
7.27
7.26
7.26
7.26
7.25
7.25
7.24
7.24
7.23
7.23
7.21
7.20
7.20
7.19
7.19
7.19
7.18
7.17
7.17
7.16
7.16
7.15
7.15
7.14
7.14
7.14
7.10
7.09
7.09
7.08
7.08
7.08
7.03
7.03
7.02
7.02
7.01
7.01
6.75
6.75
6.74
6.73
6.45
6.45
6.44
6.44
5.65
5.63
5.27
5.25
5.24
5.22
3.76
3.25
3.25
2.83
2.68
2.59
2.58
2.58
2.56
2.18
2.17
2.17
2.17
2.16
2.15
2.15
1.55
1.55
1.54
1.54
1.54
1.53
HN
OMe
7.15
5.31
2.21
2.01
2.09
2.07
1.00
1.00
3.16
1.97
1.12
1.22
3.09
1.10
1.09
2.01
1.09
1.09
2.28
[ppm]

13w

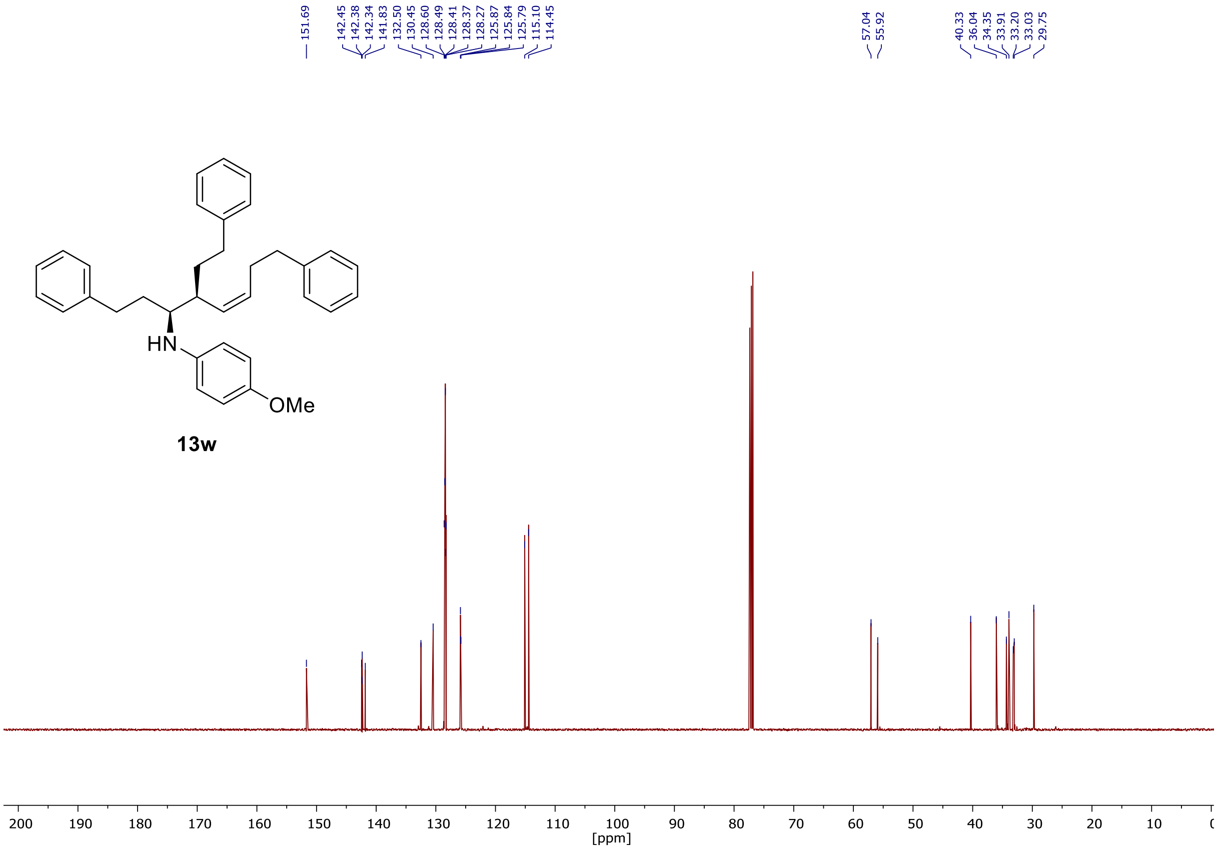
151.69
142.45
142.38
142.34
141.83
132.50
130.45
128.60
128.49
128.41
128.37
128.27
125.87
125.84
125.79
115.10
114.45
57.04
55.92
40.33
36.04
34.35
33.91
33.20
33.03
29.75
HN
OMe
13w
200 190 180 170 160 150 140 130 120 110 100 90 80 70 60 50 40 30 20 10
[ppm]

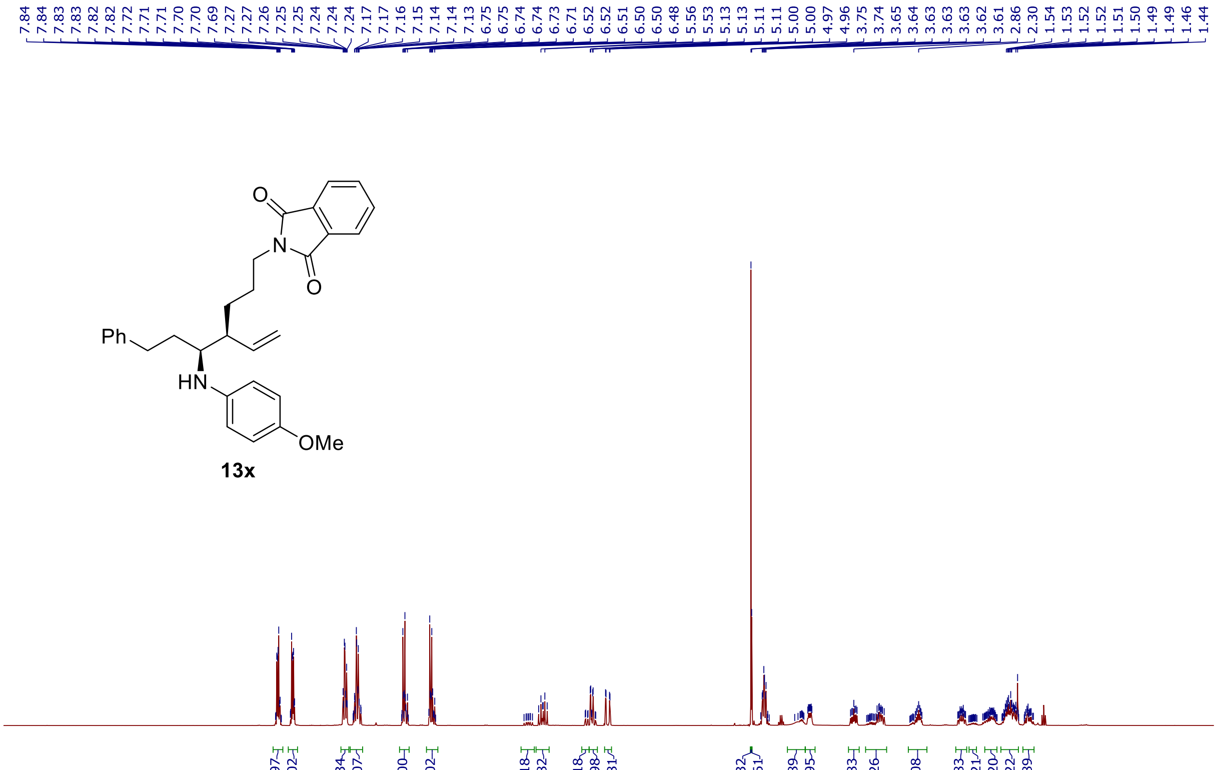
7.84 7.84 7.83 7.83 7.82 7.82 7.72 7.71 7.71 7.70 7.70 7.69 7.27 7.27 7.26 7.25 7.25 7.24 7.24 7.24 7.17 7.17 7.16 7.15 7.14 7.14 7.13 6.75 6.75 6.74 6.73 6.71 6.52 6.52 6.51 6.50 6.50 6.48 5.56 5.53 5.13 5.13 5.11 5.11 5.00 5.00 4.97 4.96 3.75 3.74 3.65 3.64 3.63 3.63 3.63 3.62 3.61 2.86 2.30 1.54 1.53 1.52 1.52 1.51 1.50 1.49 1.46 1.44
O
N
O
Ph
HN
OMe
13x
1.97 2.02 2.34 3.07 2.00 2.02 0.18 0.82 0.18 0.98 0.81 2.32 0.61 0.89 0.95 0.83 1.26 1.08 0.83 0.21 1.20 3.22 0.89
10.0 9.5 9.0 8.5 8.0 7.5 7.0 6.5 6.0 5.5 5.0 4.5 4.0 3.5 3.0 2.5 2.0 1.5 1.0 0.5 0.0
[ppm]

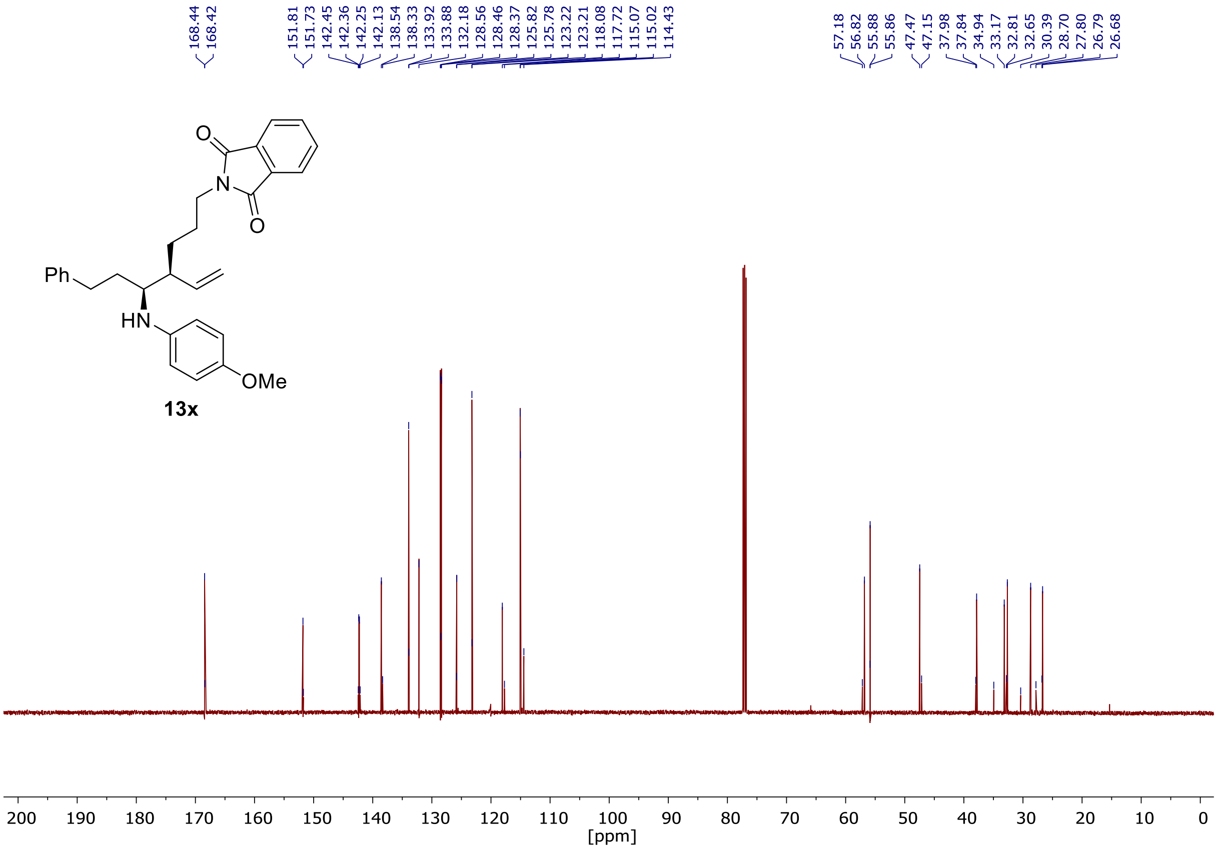
168.44
168.42
151.81
151.73
142.45
142.36
142.25
142.13
138.54
138.33
133.92
133.88
132.18
128.56
128.46
128.37
125.82
125.78
123.22
123.21
118.08
117.72
115.07
115.02
114.43
57.18
56.82
55.88
55.86
47.47
47.15
37.98
37.84
34.94
33.17
32.81
32.65
30.39
28.70
27.80
26.79
26.68
O
N
O
Ph
HN
OMe
13x
200 190 180 170 160 150 140 130 120 110 100 90 80 70 60 50 40 30 20 10 0
[ppm]

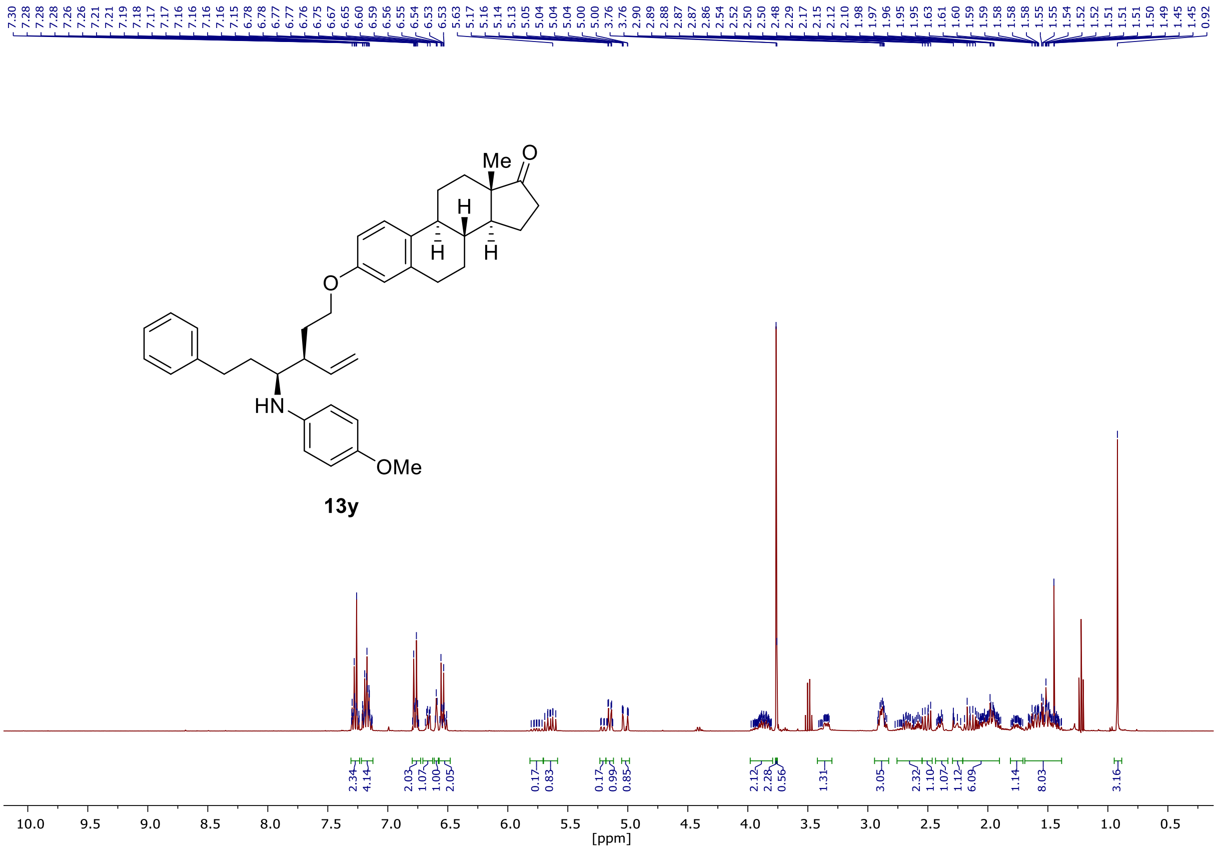
7.30 7.28 7.28 7.26 7.21 7.21 7.19 7.18 7.17 7.16 7.16 7.16 7.15 6.78 6.78 6.77 6.76 6.75 6.67 6.65 6.60 6.59 6.56 6.55 6.54 6.53 6.53 5.63 5.17 5.16 5.14 5.13 5.05 5.04 5.04 5.04 5.00 5.00 3.76 2.90 2.89 2.88 2.87 2.86 2.54 2.52 2.50 2.50 2.48 2.29 2.17 2.15 2.12 2.10 1.98 1.97 1.96 1.95 1.95 1.63 1.61 1.60 1.59 1.59 1.58 1.58 1.58 1.55 1.55 1.54 1.52 1.52 1.51 1.51 1.51 1.50 1.49 1.45 0.92
Me
O
H
H
H
O
HN
OMe
13y
2.34 4.14 2.03 1.07 1.00 2.05 0.17 0.83 0.17 0.99 0.85 2.12 2.28 0.56 1.31 3.05 2.32 1.10 1.07 1.12 6.09 1.14 8.03 3.16
10.0 9.5 9.0 8.5 8.0 7.5 7.0 6.5 6.0 5.5 5.0 4.5 4.0 3.5 3.0 2.5 2.0 1.5 1.0 0.5
[ppm]

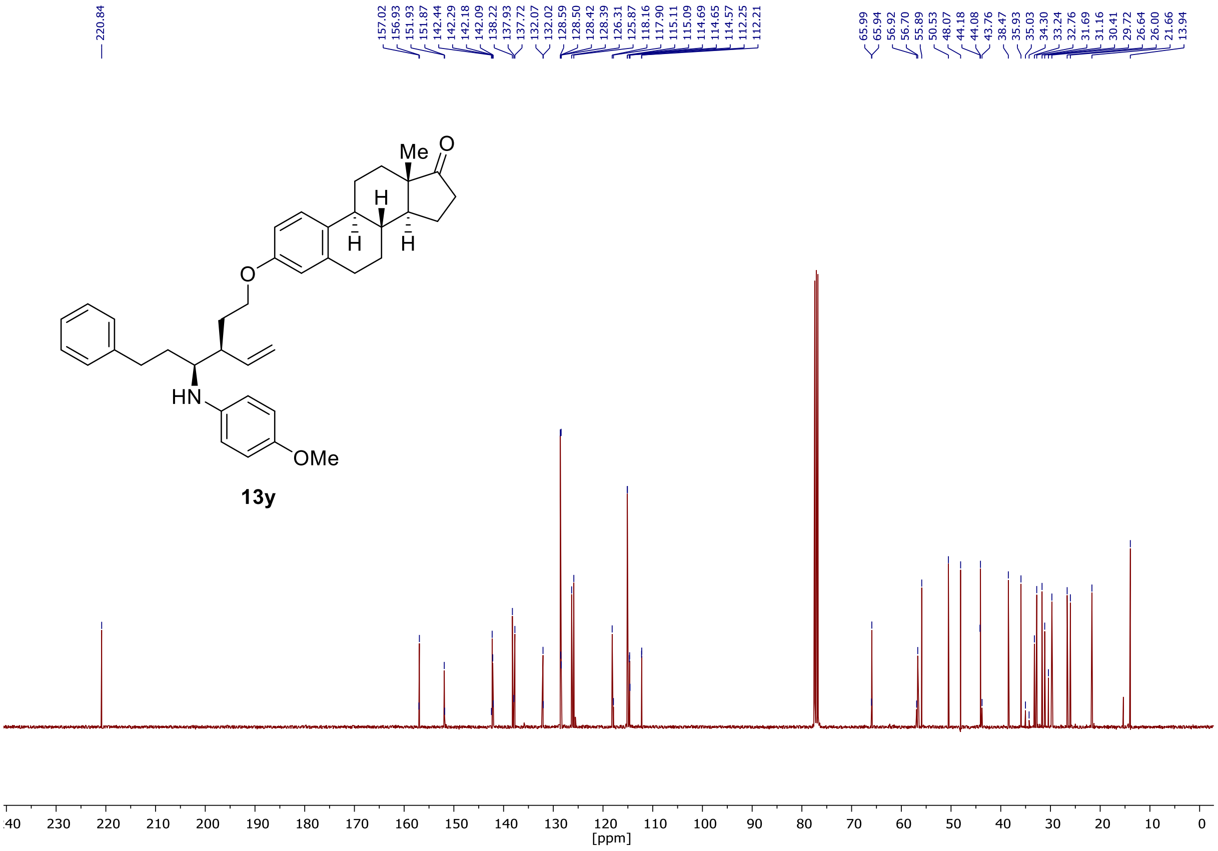
220.84
157.02 156.93 151.93 151.87 142.44 142.29 142.18 142.09 138.22 137.93 137.72 132.07 132.02 128.59 128.50 128.42 128.39 126.31 125.87 118.16 117.90 115.11 115.09 114.69 114.65 114.57 112.25 112.21
65.99 65.94 56.92 56.70 55.89 50.53 48.07 44.18 44.08 43.76 38.47 35.93 35.03 34.30 33.24 32.76 31.69 31.16 30.41 29.72 26.64 26.00 21.66 13.94
Me
O
H
H
H
HN
OMe
13y
[ppm]

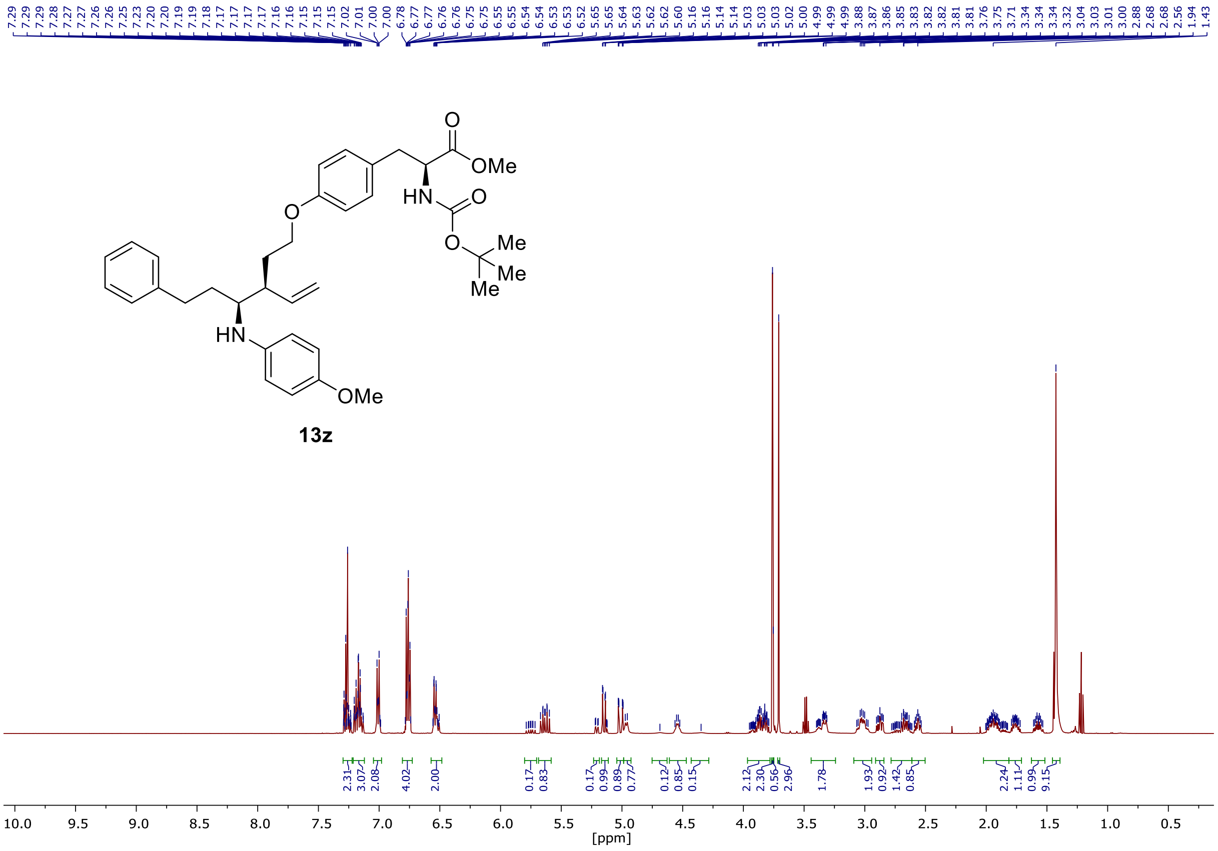
7.29 7.29 7.28 7.27 7.27 7.26 7.25 7.23 7.20 7.20 7.19 7.19 7.18 7.17 7.17 7.17 7.16 7.16 7.15 7.15 7.02 7.01 7.00 6.78 6.77 6.77 6.76 6.76 6.75 6.75 6.55 6.55 6.54 6.54 6.53 6.53 6.52 5.65 5.65 5.64 5.63 5.62 5.62 5.60 5.16 5.16 5.14 5.14 5.03 5.03 5.03 5.02 5.00 4.99 4.99 4.99 3.88 3.87 3.86 3.85 3.83 3.82 3.82 3.81 3.81 3.76 3.75 3.71 3.34 3.34 3.34 3.32 3.04 3.03 3.01 3.00 2.88 2.68 2.68 2.56 1.94 1.43
O
OMe
HN
O
O
Me
Me
Me
HN
OMe
13z
2.31 3.07 2.08 4.02 2.00 0.17 0.83 0.17 0.99 0.89 0.77 0.12 0.85 0.15 2.12 2.30 0.56 2.96 1.78 1.93 0.92 1.42 0.85 2.24 1.11 0.99 9.15
[ppm]

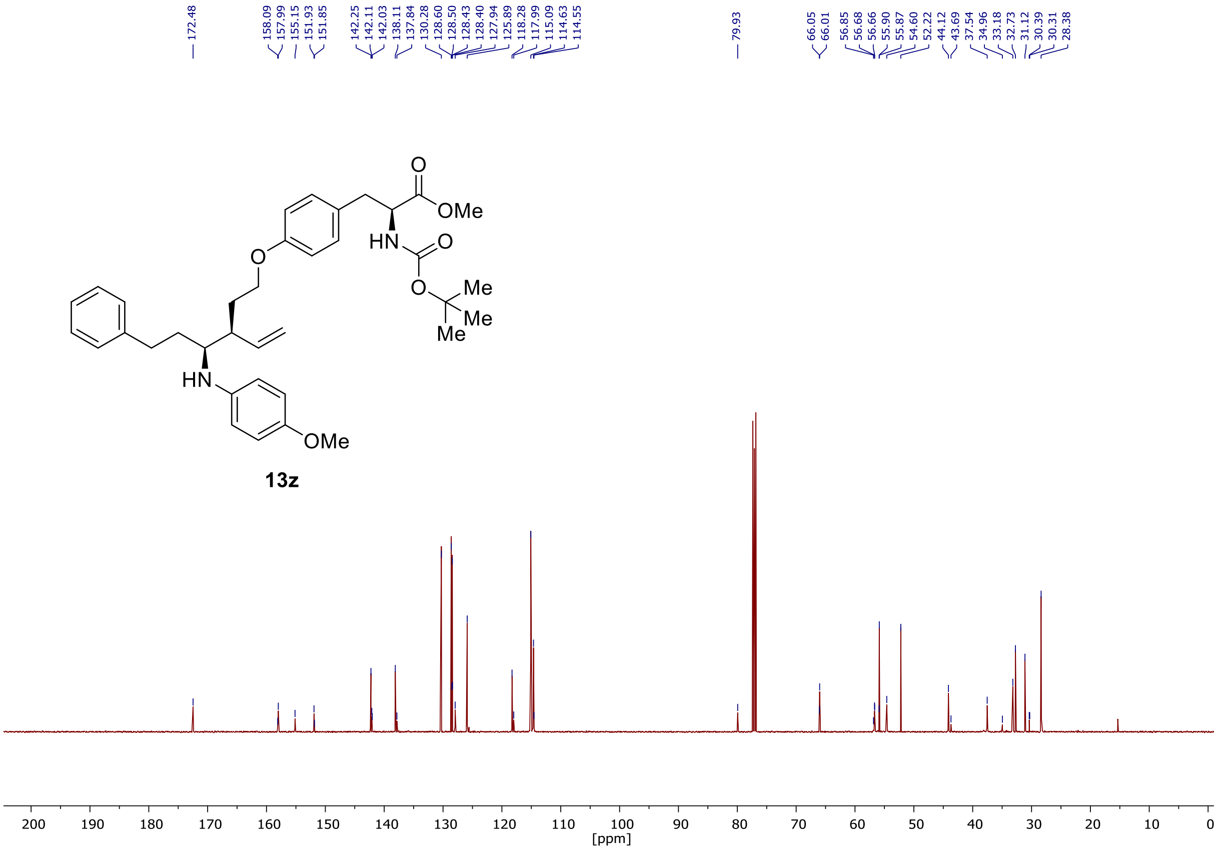
172.48
158.09 157.99 155.15 151.93 151.85
142.25 142.11 142.03 138.11 137.84 130.28 128.60 128.50 128.43 128.40 127.94 125.89 118.28 117.99 115.09 114.63 114.55
79.93
66.05 66.01 56.85 56.68 56.66 55.90 55.87 54.60 52.22 44.12 43.69 37.54 34.96 33.18 32.73 31.12 30.39 30.31 28.38
O
OMe
HN
O
O
Me
Me
Me
O
HN
OMe
13z
200 190 180 170 160 150 140 130 120 110 100 90 80 70 60 50 40 30 20 10 0
[ppm]

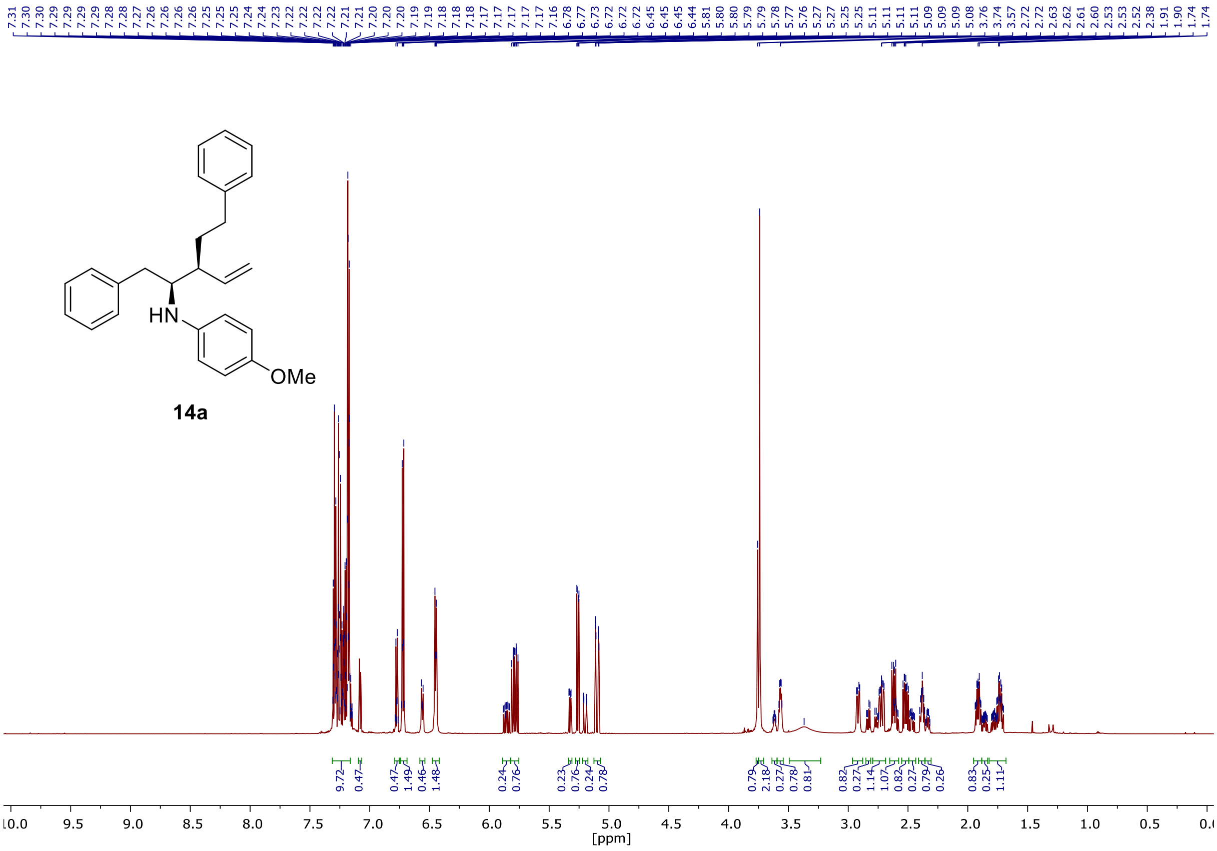
7.31 7.30 7.30 7.29 7.29 7.29 7.28 7.28 7.27 7.26 7.26 7.26 7.25 7.25 7.25 7.24 7.24 7.23 7.22 7.22 7.22 7.21 7.21 7.21 7.20 7.20 7.19 7.18 7.18 7.18 7.17 7.17 7.17 7.17 7.16 6.78 6.77 6.73 6.72 6.72 6.72 6.45 6.45 6.45 6.44 5.81 5.80 5.80 5.79 5.79 5.78 5.77 5.76 5.27 5.27 5.25 5.11 5.11 5.11 5.11 5.09 5.09 5.08 3.76 3.74 3.57 2.72 2.72 2.63 2.62 2.61 2.60 2.53 2.53 2.52 2.38 1.91 1.90 1.74 1.74
HN
OMe
14a
9.72 0.47 0.47 1.49 0.46 1.48 0.24 0.76 0.23 0.76 0.24 0.78 0.79 2.18 0.27 0.78 0.81 0.82 0.27 1.14 1.07 0.82 0.27 0.79 0.26 0.83 0.25 1.11
10.0 9.5 9.0 8.5 8.0 7.5 7.0 6.5 6.0 5.5 5.0 4.5 4.0 3.5 3.0 2.5 2.0 1.5 1.0 0.5 0.0
[ppm]

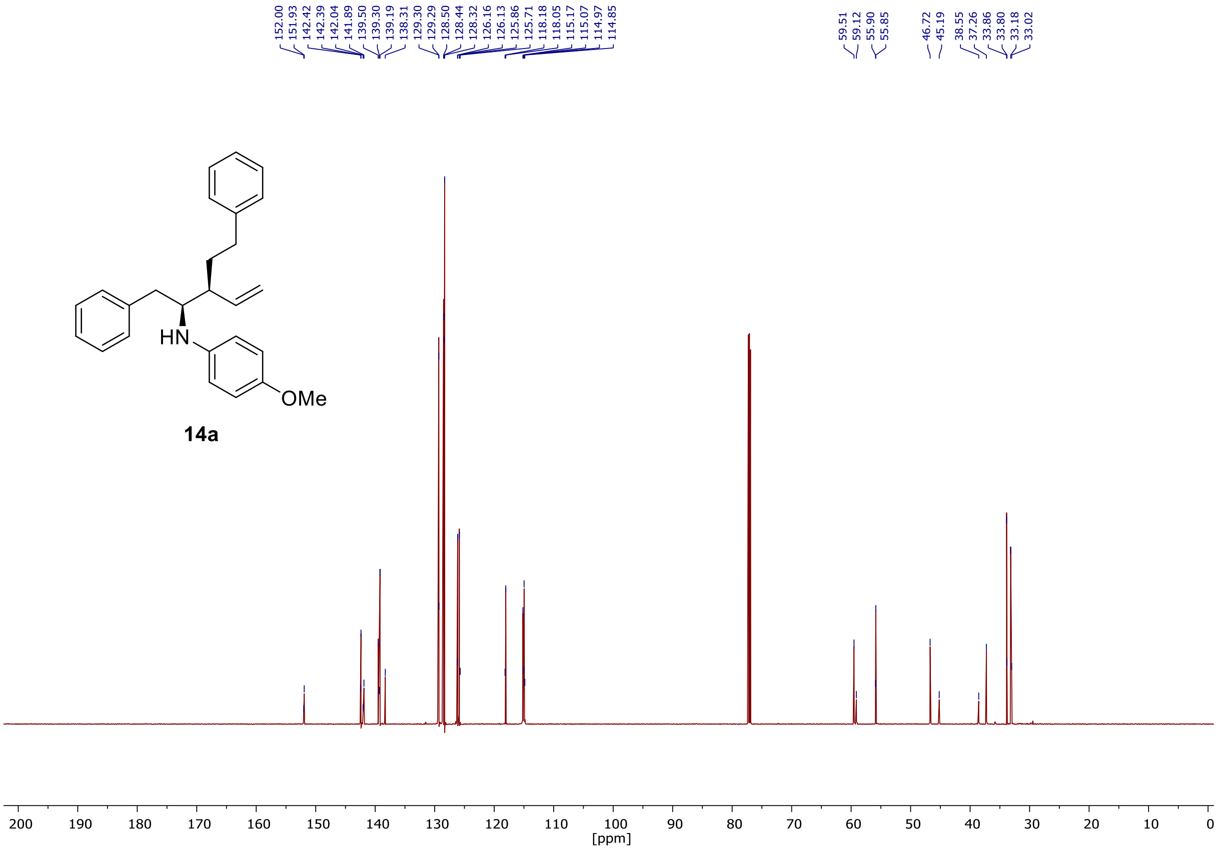
152.00 151.93 142.42 142.39 142.04 141.89 139.50 139.30 139.19 138.31 129.30 129.29 128.50 128.44 128.32 126.16 126.13 125.86 125.71 118.18 118.05 115.17 115.07 114.97 114.85
59.51 59.12 55.90 55.85
46.72 45.19 38.55 37.26 33.86 33.80 33.18 33.02
HN
OMe
14a
200 190 180 170 160 150 140 130 120 110 100 90 80 70 60 50 40 30 20 10 0
[ppm]

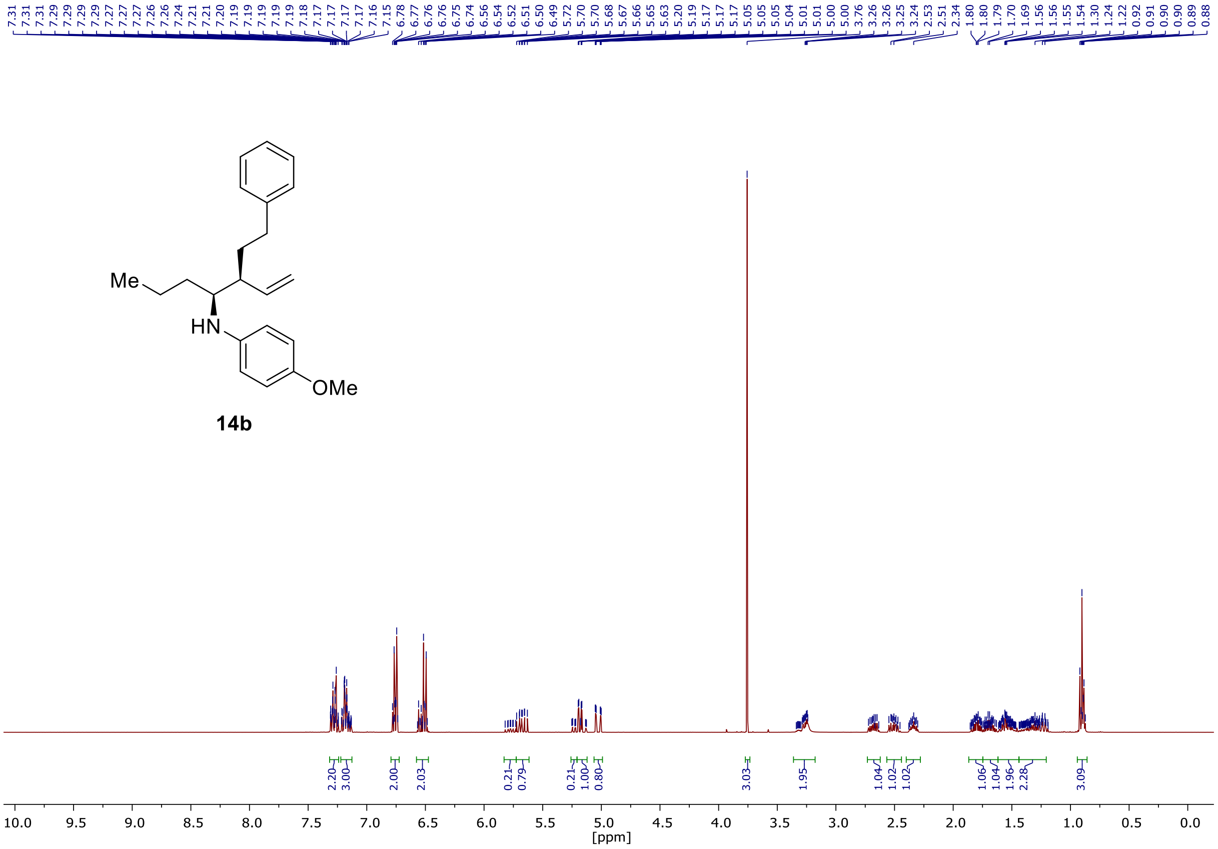
7.31 7.31 7.31 7.29 7.29 7.29 7.27 7.27 7.26 7.24 7.21 7.21 7.20 7.19 7.19 7.19 7.19 7.18 7.17 7.17 7.17 7.16 7.15 6.78 6.77 6.76 6.76 6.75 6.74 6.56 6.54 6.52 6.51 6.50 6.49 5.72 5.70 5.70 5.68 5.67 5.66 5.65 5.63 5.20 5.19 5.17 5.17 5.17 5.05 5.05 5.05 5.04 5.01 5.01 5.00 3.76 3.26 3.26 3.25 3.24 2.53 2.51 2.34 1.80 1.80 1.79 1.70 1.69 1.56 1.56 1.55 1.54 1.30 1.24 1.22 0.92 0.91 0.90 0.90 0.89 0.88
Me
HN
OMe
14b
2.20 3.00 2.00 2.03 0.21 0.79 0.21 1.00 0.80 3.03 1.95 1.04 1.02 1.02 1.06 1.04 1.96 2.28 3.09
10.0 9.5 9.0 8.5 8.0 7.5 7.0 6.5 6.0 5.5 5.0 4.5 4.0 3.5 3.0 2.5 2.0 1.5 1.0 0.5 0.0
[ppm]

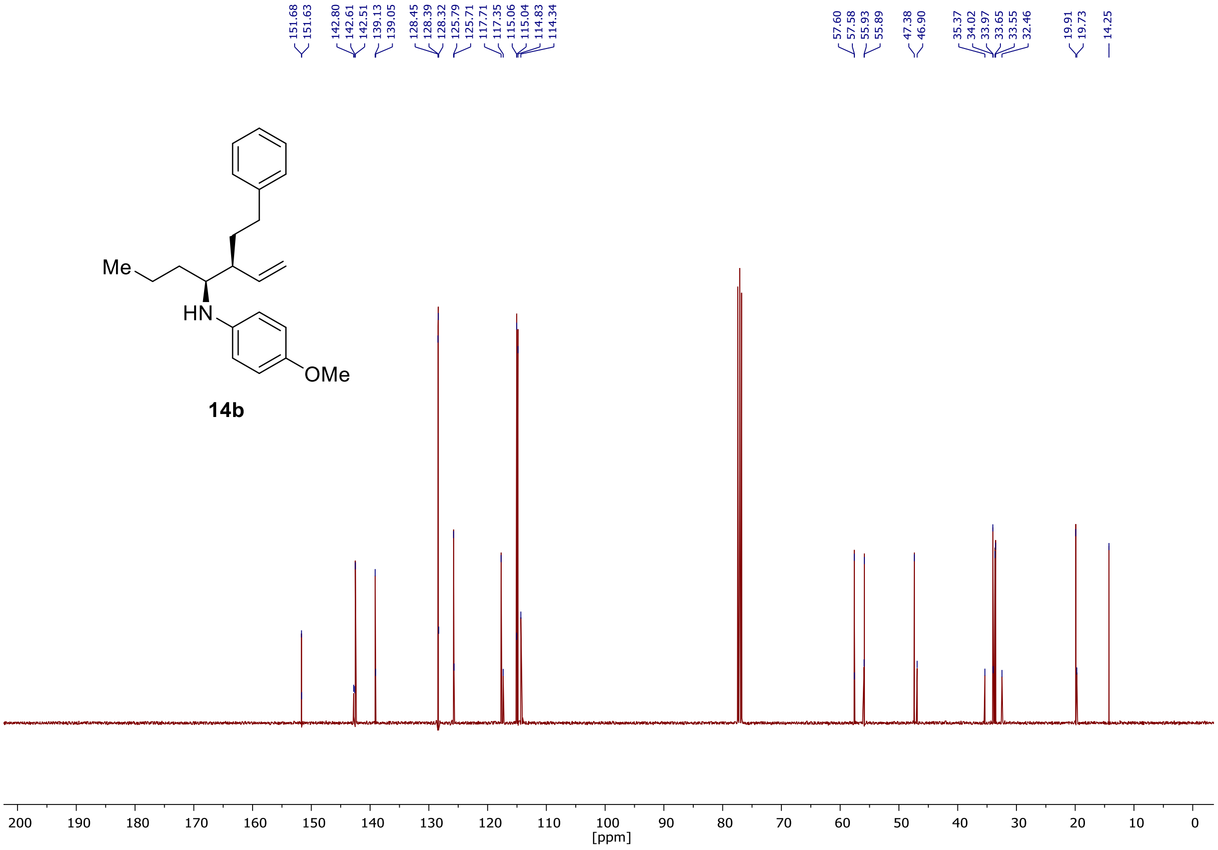
151.68 151.63 142.80 142.61 142.51 139.13 139.05 128.45 128.39 128.32 125.79 125.71 117.71 117.35 115.06 115.04 114.83 114.34
57.60 57.58 55.93 55.89 47.38 46.90 35.37 34.02 33.97 33.65 33.55 32.46 19.91 19.73 14.25
Me
HN
OMe
14b
200 190 180 170 160 150 140 130 120 110 100 90 80 70 60 50 40 30 20 10 0
[ppm]

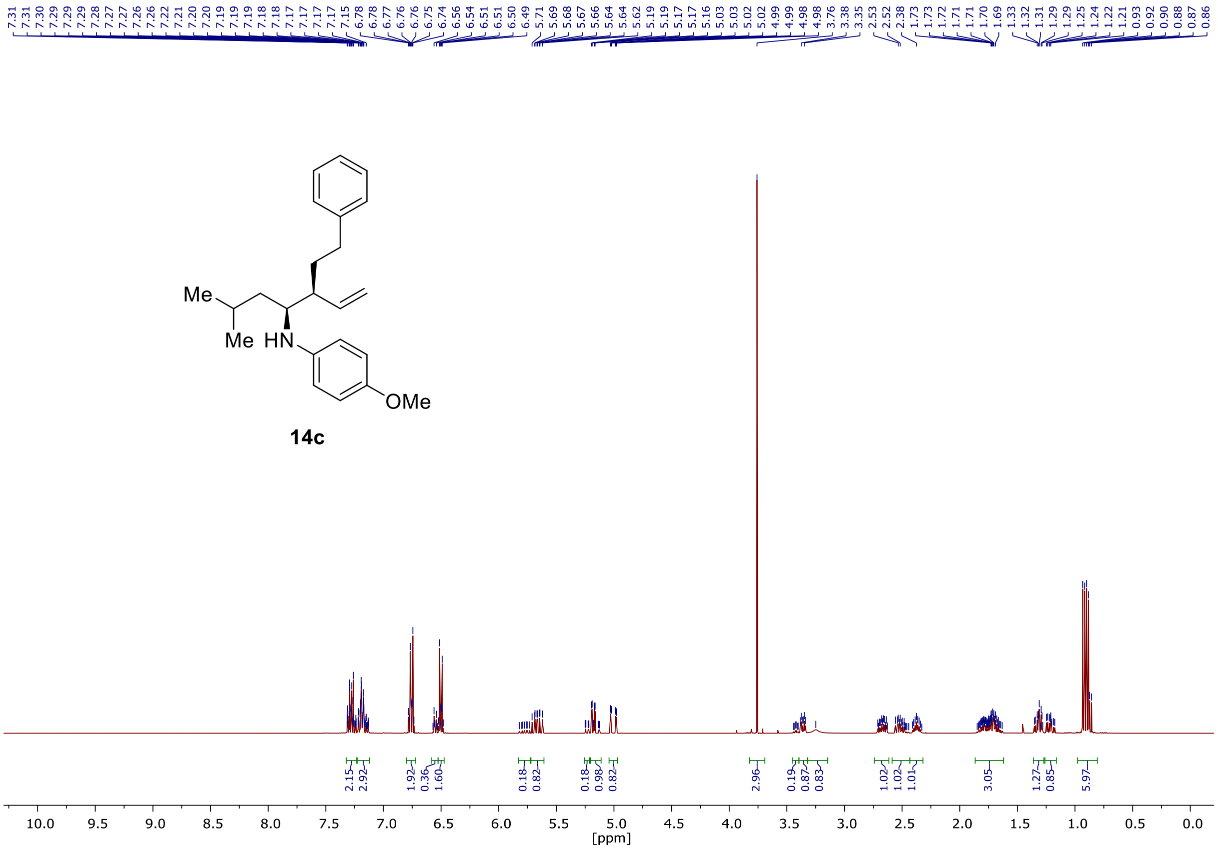
7.31 7.31 7.30 7.29 7.29 7.28 7.27 7.27 7.26 7.22 7.21 7.20 7.19 7.19 7.18 7.17 7.17 7.17 7.15 6.78 6.77 6.76 6.76 6.75 6.74 6.56 6.54 6.51 6.50 6.49 5.71 5.69 5.68 5.67 5.66 5.64 5.62 5.19 5.19 5.17 5.17 5.16 5.03 5.03 5.02 5.02 4.99 4.99 4.98 4.98 3.76 3.38 3.35 2.53 2.52 2.38 1.73 1.72 1.71 1.71 1.70 1.69 1.33 1.32 1.31 1.29 1.25 1.24 1.22 1.21 0.93 0.92 0.90 0.88 0.87 0.86
Me
Me HN
OMe
14c
2.15 2.92 1.92 0.36 1.60 0.18 0.82 0.18 0.98 0.82 2.96 0.19 0.87 0.83 1.02 1.02 1.01 3.05 1.27 0.85 5.97
10.0 9.5 9.0 8.5 8.0 7.5 7.0 6.5 6.0 5.5 5.0 4.5 4.0 3.5 3.0 2.5 2.0 1.5 1.0 0.5 0.0
[ppm]

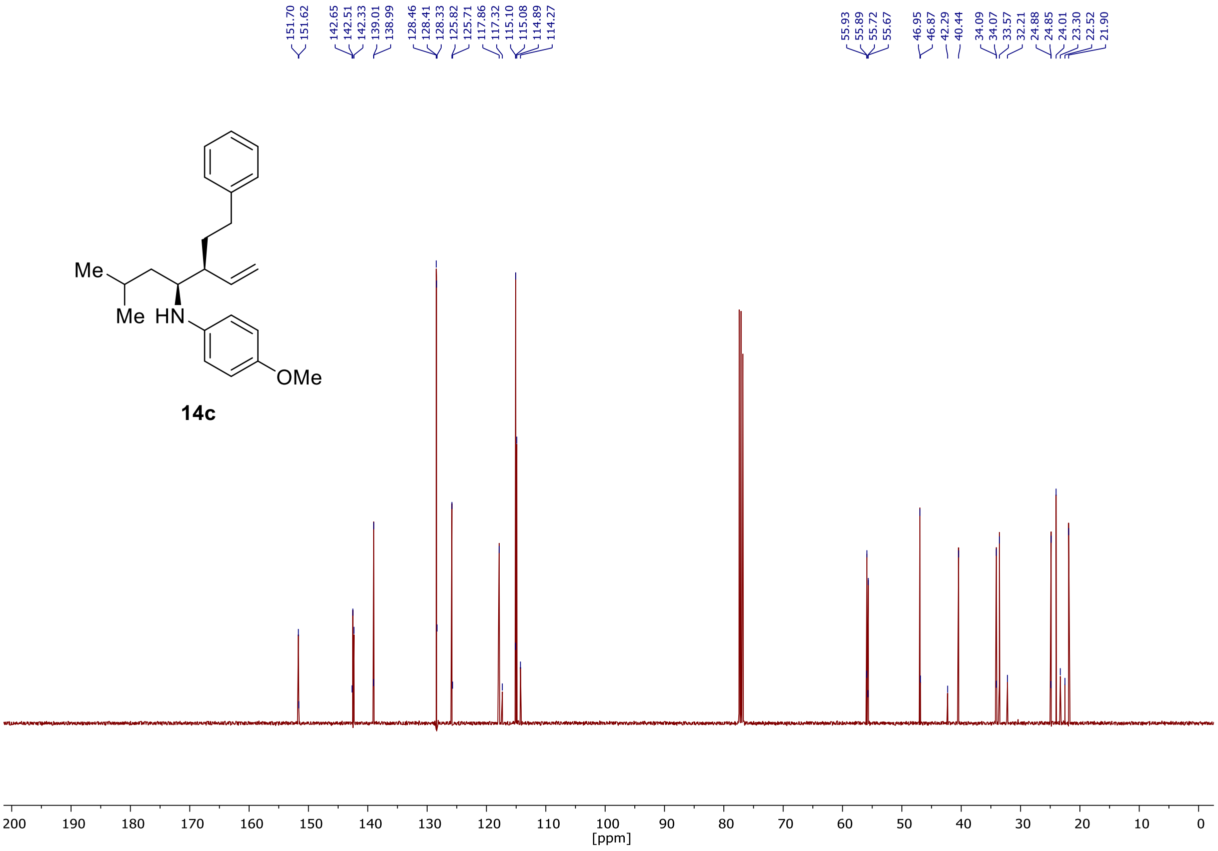
151.70
151.62
142.65
142.51
142.33
139.01
138.99
128.46
128.41
128.33
125.82
125.71
117.86
117.32
115.10
115.08
114.89
114.27
55.93
55.89
55.72
55.67
46.95
46.87
42.29
40.44
34.09
34.07
33.57
32.21
24.88
24.85
24.01
23.30
22.52
21.90
Me
Me HN
OMe
14c
200 190 180 170 160 150 140 130 120 110 100 90 80 70 60 50 40 30 20 10 0
[ppm]

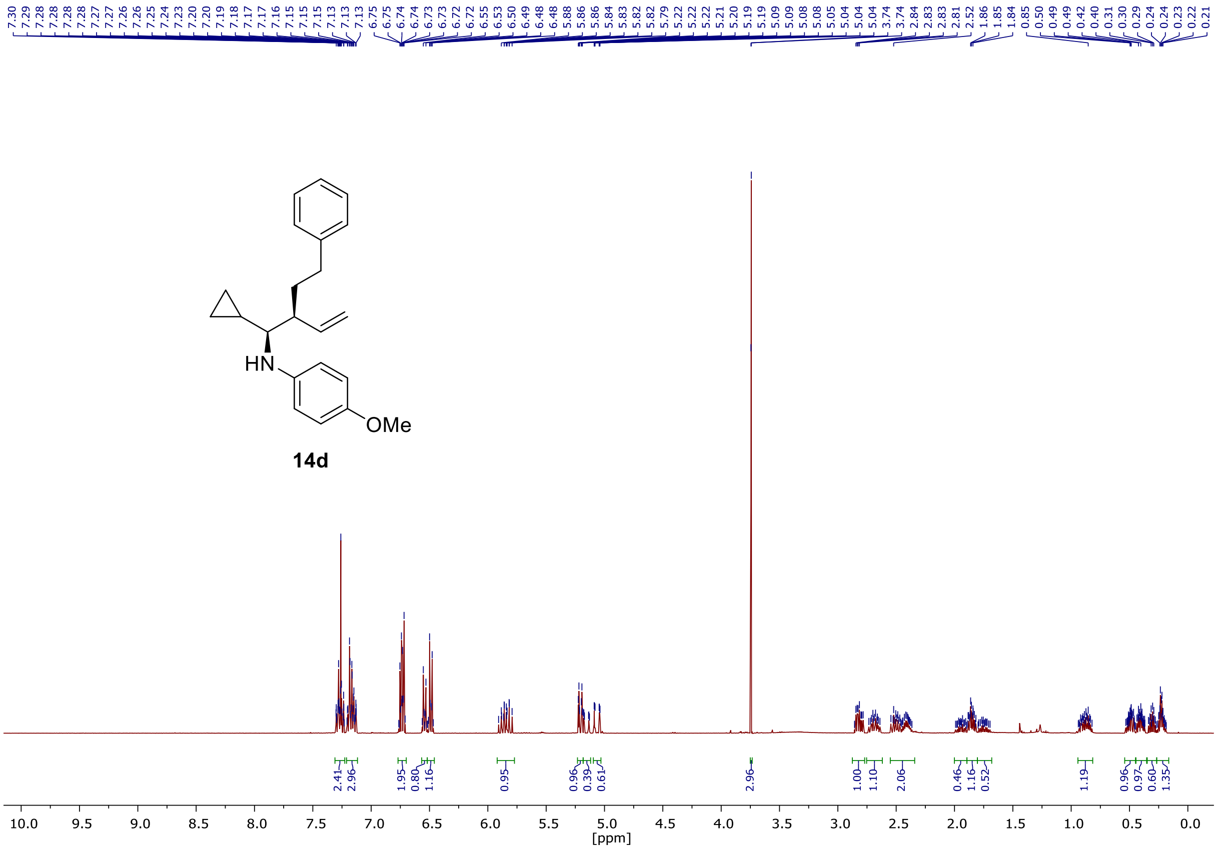
7.30 7.29 7.28 7.28 7.28 7.27 7.27 7.26 7.25 7.24 7.23 7.20 7.20 7.19 7.18 7.17 7.17 7.16 7.15 7.15 7.15 7.13 7.13 7.13 6.75 6.74 6.74 6.73 6.73 6.72 6.55 6.53 6.50 6.49 6.48 6.48 5.88 5.86 5.86 5.84 5.83 5.82 5.82 5.79 5.22 5.22 5.22 5.21 5.20 5.20 5.19 5.19 5.09 5.09 5.08 5.08 5.05 5.04 5.04 5.04 3.74 3.74 2.84 2.83 2.83 2.81 2.52 1.86 1.85 1.84 0.85 0.50 0.49 0.49 0.42 0.40 0.31 0.30 0.29 0.24 0.24 0.23 0.22 0.21
HN
OMe
14d
2.41 2.96 1.95 0.80 1.16 0.95 0.96 0.39 0.61 2.96 1.00 1.10 2.06 0.46 1.16 0.52 1.19 0.96 0.97 0.60 1.35
10.0 9.5 9.0 8.5 8.0 7.5 7.0 6.5 6.0 5.5 5.0 4.5 4.0 3.5 3.0 2.5 2.0 1.5 1.0 0.5 0.0
[ppm]

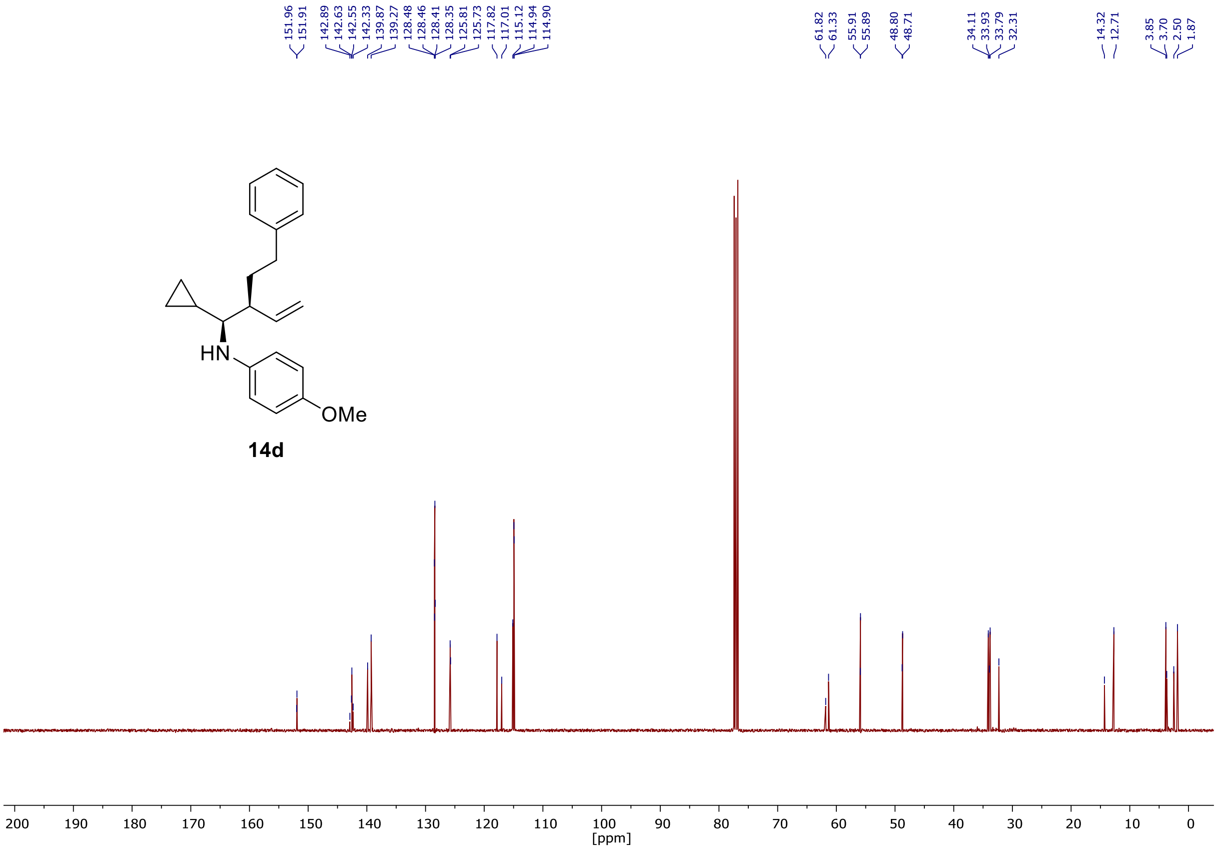
151.96 151.91 142.89 142.63 142.55 142.33 139.87 139.27 128.48 128.46 128.41 128.35 125.81 125.73 117.82 117.01 115.12 114.94 114.90
61.82 61.33 55.91 55.89 48.80 48.71 34.11 33.93 33.79 32.31 14.32 12.71 3.85 3.70 2.50 1.87
HN
OMe
[ppm]

14d

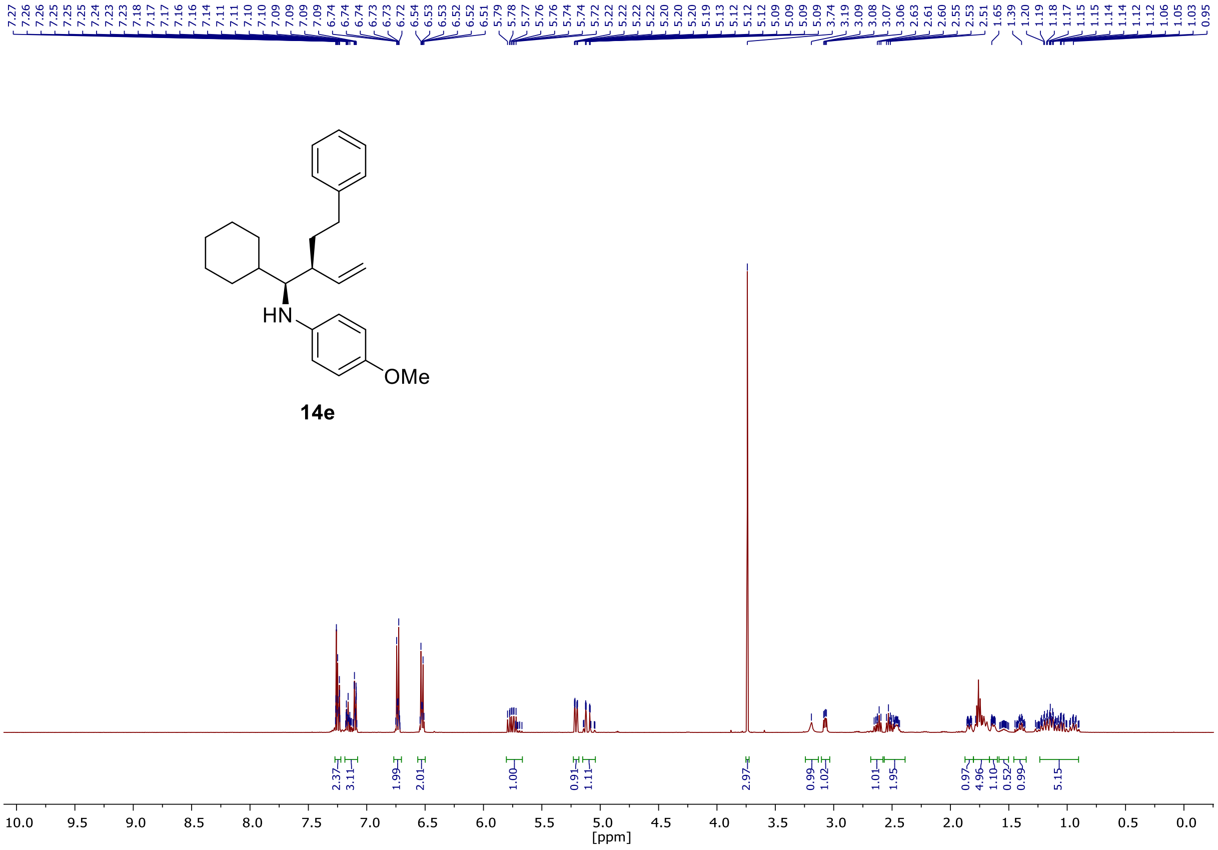
7.27 7.26 7.25 7.25 7.24 7.23 7.23 7.18 7.17 7.17 7.16 7.14 7.11 7.11 7.10 7.09 7.09 7.09 7.09 6.74 6.74 6.74 6.73 6.73 6.72 6.54 6.53 6.53 6.52 6.52 6.51 5.79 5.78 5.77 5.76 5.76 5.74 5.74 5.72 5.22 5.22 5.22 5.22 5.20 5.20 5.20 5.19 5.13 5.12 5.12 5.09 5.09 5.09 5.09 3.74 3.19 3.09 3.08 3.07 3.06 2.63 2.61 2.60 2.55 2.53 2.51 1.65 1.39 1.20 1.19 1.18 1.17 1.15 1.14 1.14 1.12 1.12 1.06 1.05 1.03 0.95
HN
OMe
2.37 3.11 1.99 2.01 1.00 0.91 1.11 2.97 0.99 1.02 1.01 1.95 0.97 4.96 1.10 0.52 0.99 5.15
[ppm]

14e

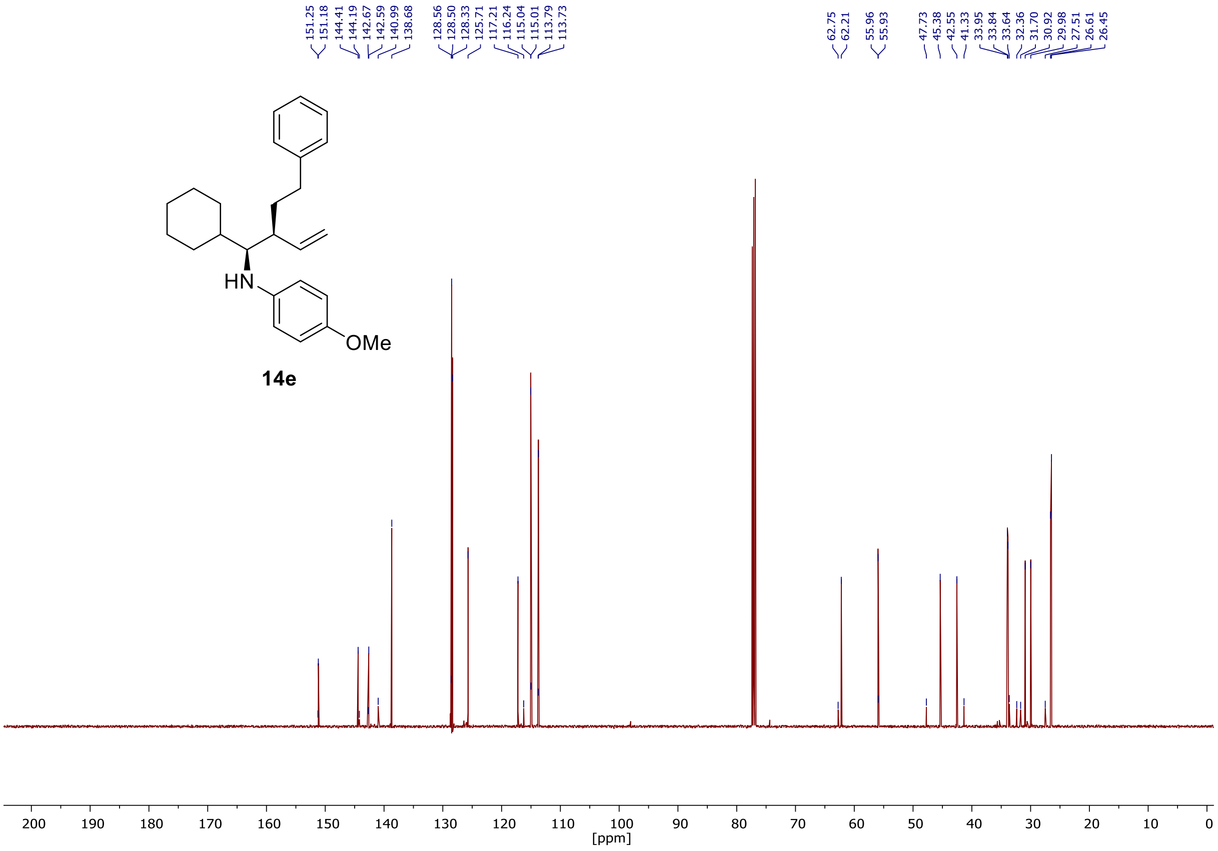
151.25
151.18
144.41
144.19
142.67
142.59
140.99
138.68
128.56
128.50
128.33
125.71
117.21
116.24
115.04
115.01
113.79
113.73
62.75
62.21
55.96
55.93
47.73
45.38
42.55
41.33
33.95
33.84
33.64
32.36
31.70
30.92
29.98
27.51
26.61
26.45
HN
OMe
14e
200 190 180 170 160 150 140 130 120 110 100 90 80 70 60 50 40 30 20 10 0
[ppm]

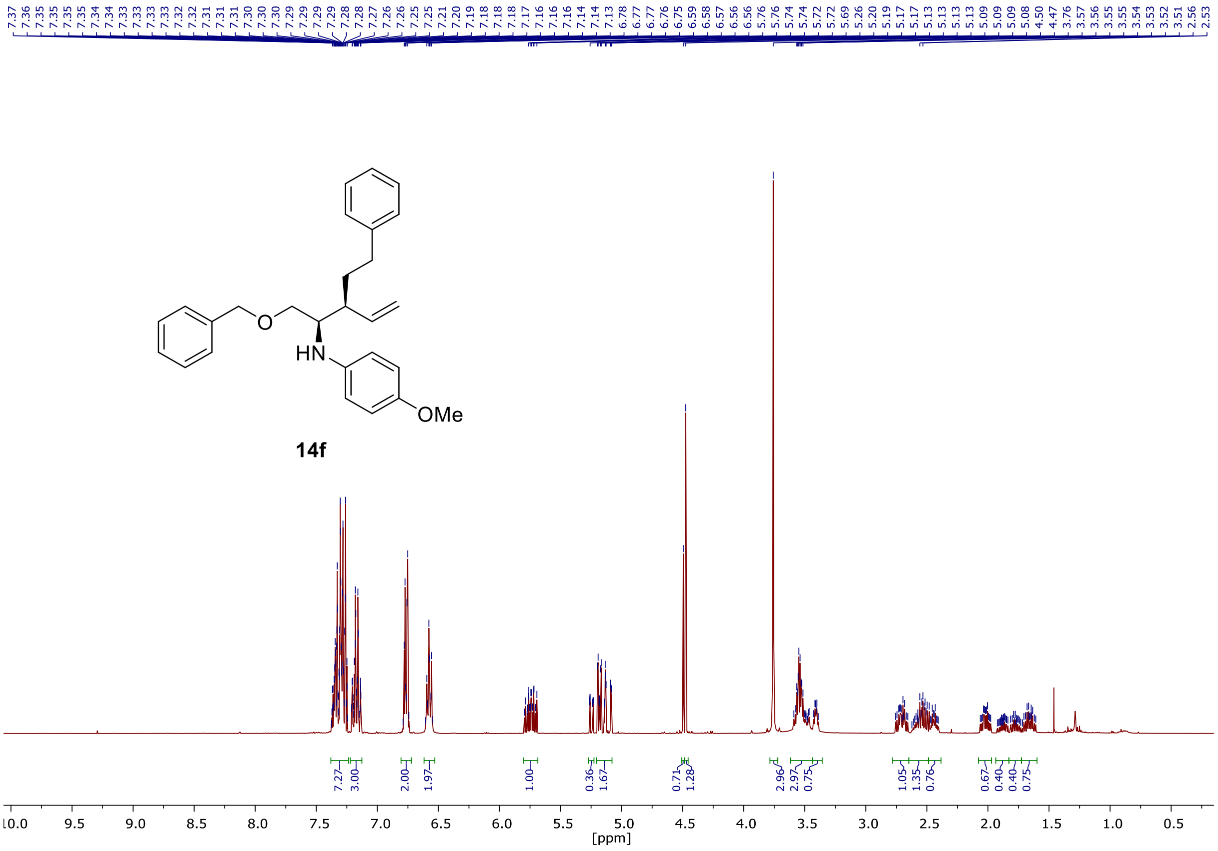
7.37 7.36 7.35 7.35 7.35 7.34 7.34 7.33 7.33 7.33 7.33 7.32 7.32 7.31 7.31 7.30 7.30 7.29 7.29 7.29 7.28 7.28 7.27 7.26 7.26 7.25 7.25 7.21 7.20 7.19 7.18 7.18 7.17 7.16 7.16 7.14 7.14 7.13 6.78 6.77 6.77 6.76 6.75 6.59 6.58 6.57 6.56 6.56 5.76 5.76 5.74 5.74 5.72 5.72 5.69 5.26 5.20 5.19 5.17 5.17 5.13 5.13 5.13 5.09 5.09 5.09 5.08 4.50 4.47 3.76 3.57 3.56 3.55 3.55 3.54 3.53 3.52 3.51 2.56 2.53
O
HN
OMe
14f
7.27 3.00 2.00 1.97 1.00 0.36 1.67 0.71 1.28 2.96 2.97 0.75 1.05 1.35 0.76 0.67 0.40 0.40 0.75
10.0 9.5 9.0 8.5 8.0 7.5 7.0 6.5 6.0 5.5 5.0 4.5 4.0 3.5 3.0 2.5 2.0 1.5 1.0 0.5
[ppm]

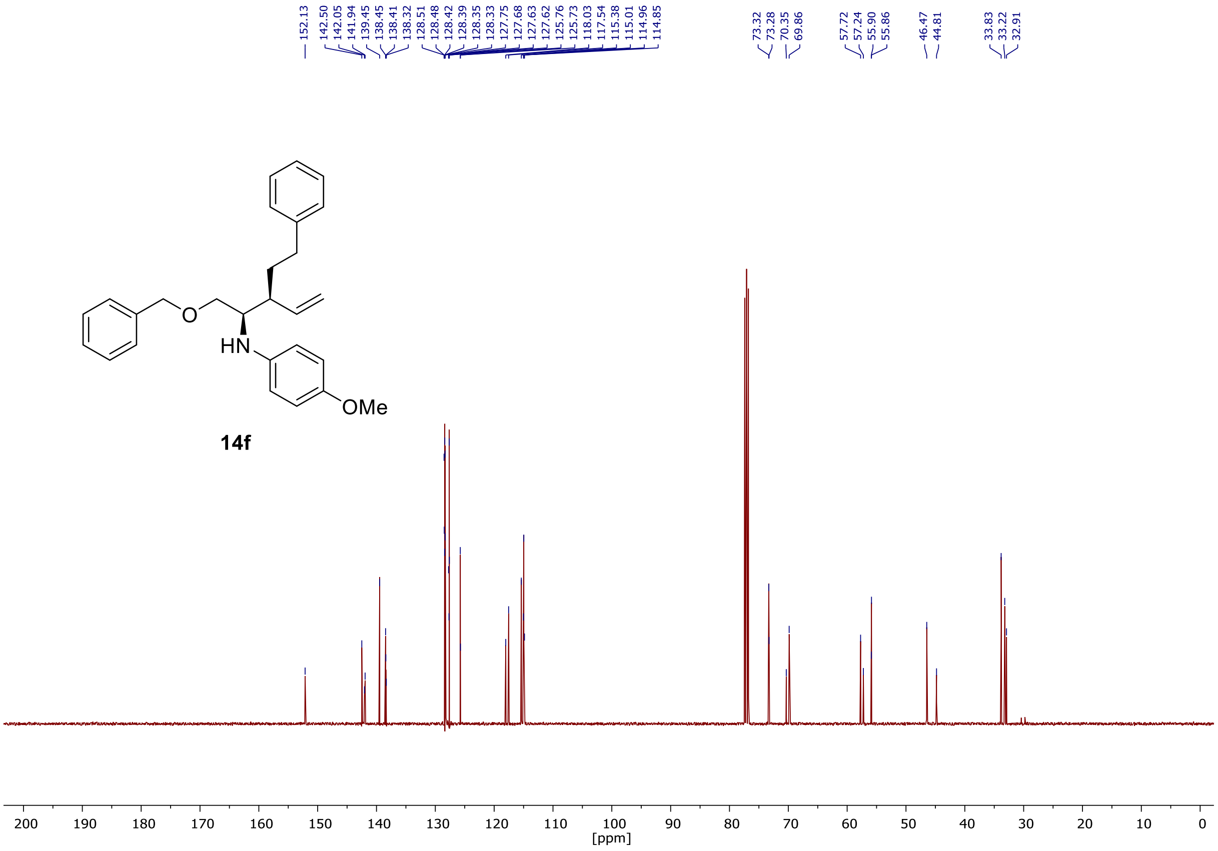
O
HN
OMe
14f
[ppm]

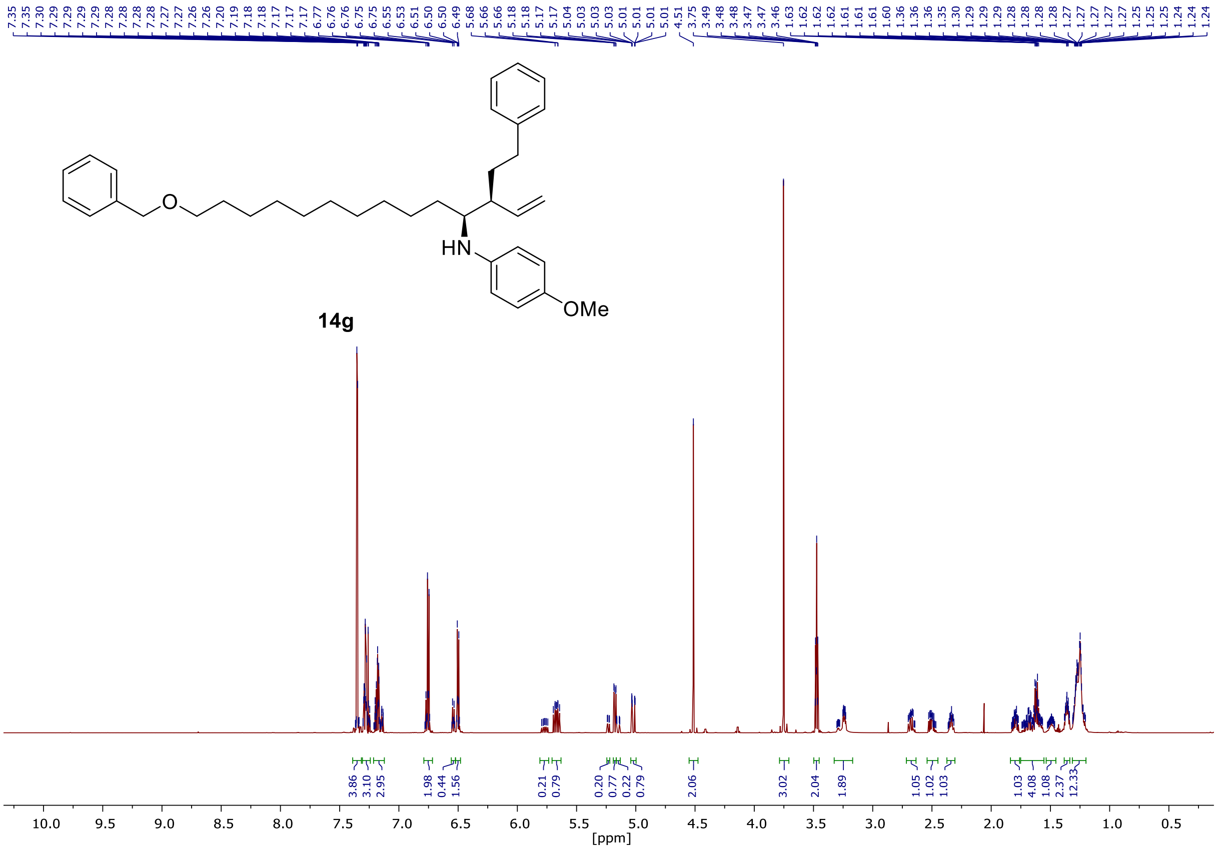
O
HN
OMe
14g
[ppm]

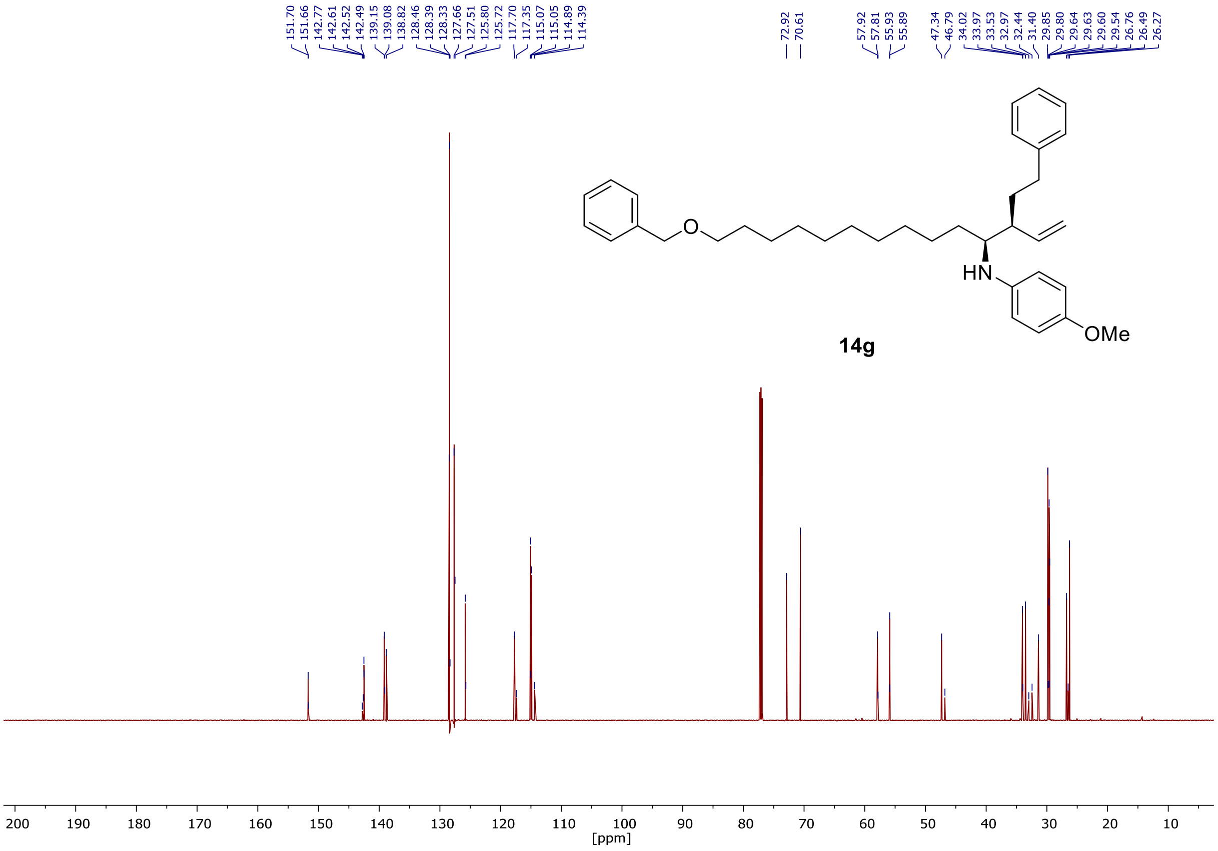
151.70
151.66
142.77
142.61
142.52
142.49
139.15
139.08
138.82
128.46
128.39
128.33
127.66
127.51
125.80
125.72
117.70
117.35
115.07
115.05
114.89
114.39
72.92
70.61
57.92
57.81
55.93
55.89
47.34
46.79
34.02
33.97
33.53
32.97
32.44
31.40
29.85
29.80
29.64
29.63
29.60
29.54
26.76
26.49
26.27
O
HN
OMe
14g
200 190 180 170 160 150 140 130 120 110 100 90 80 70 60 50 40 30 20 10
[ppm]

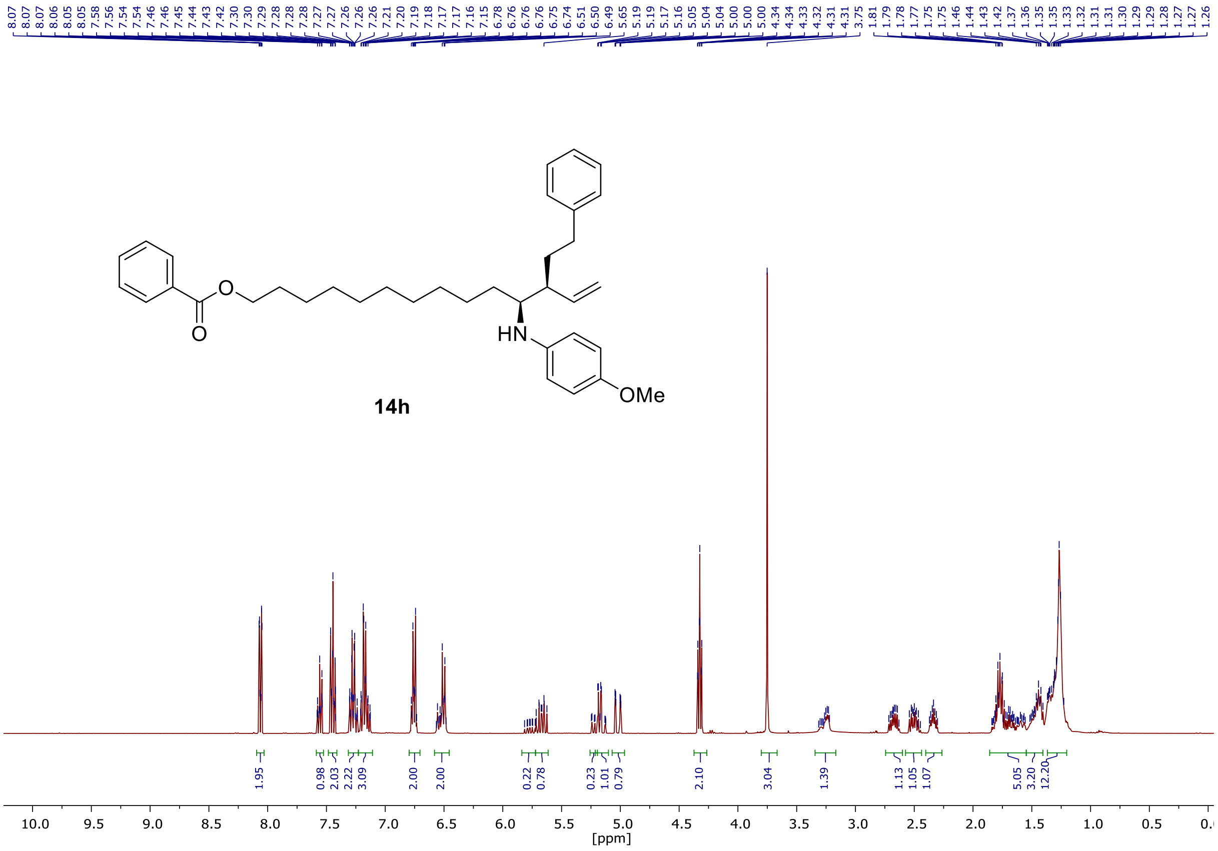
8.07
8.07
8.07
8.06
8.05
8.05
7.58
7.56
7.54
7.54
7.46
7.45
7.44
7.43
7.42
7.30
7.30
7.29
7.28
7.28
7.27
7.27
7.26
7.26
7.21
7.20
7.19
7.18
7.17
7.16
7.15
6.78
6.76
6.76
6.75
6.74
6.51
6.50
6.49
5.65
5.19
5.19
5.17
5.16
5.05
5.04
5.00
5.00
4.34
4.34
4.32
4.31
4.31
3.75
1.81
1.79
1.78
1.77
1.75
1.75
1.46
1.44
1.43
1.42
1.37
1.36
1.35
1.35
1.33
1.32
1.31
1.31
1.30
1.29
1.29
1.28
1.27
1.27
1.26
O
O
HN
OMe
14h
1.95
0.98
2.03
2.22
3.09
2.00
2.00
0.22
0.78
0.23
1.01
0.79
2.10
3.04
1.39
1.13
1.05
1.07
5.05
3.20
12.20
10.0 9.5 9.0 8.5 8.0 7.5 7.0 6.5 6.0 5.5 5.0 4.5 4.0 3.5 3.0 2.5 2.0 1.5 1.0 0.5
[ppm]

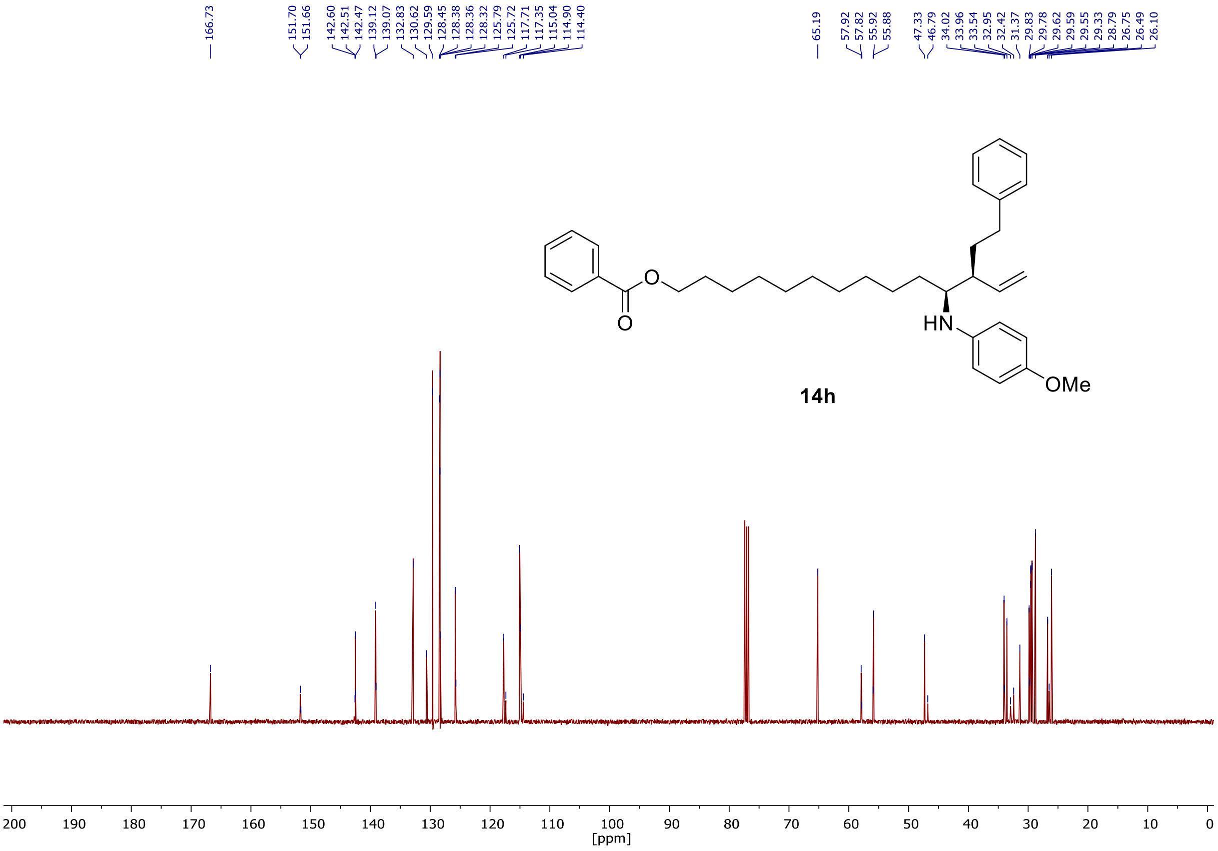
166.73
151.70 151.66
142.60 142.51 142.47 139.12 139.07 132.83 130.62 129.59 128.45 128.38 128.36 128.32 125.79 125.72 117.71 117.35 115.04 114.90 114.40
65.19
57.92 57.82 55.92 55.88
47.33 46.79 34.02 33.96 33.54 32.95 32.42 31.37 29.83 29.78 29.62 29.59 29.55 29.33 28.79 26.75 26.49 26.10
O
O
HN
OMe
14h
200 190 180 170 160 150 140 130 120 110 100 90 80 70 60 50 40 30 20 10 0
[ppm]

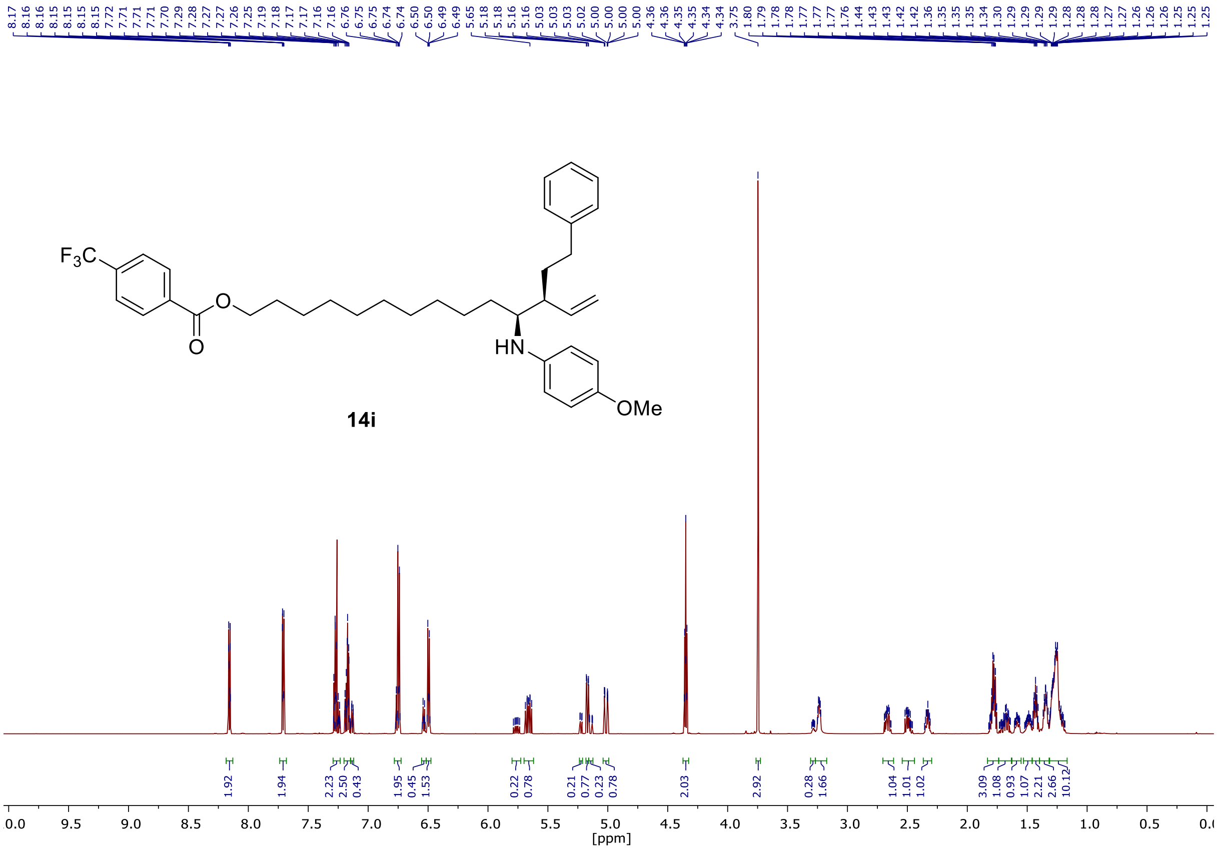
8.17 8.16 8.16 8.15 8.15 8.15 7.72 7.71 7.71 7.71 7.70 7.29 7.28 7.27 7.27 7.26 7.25 7.19 7.18 7.17 7.17 7.16 7.16 6.76 6.75 6.75 6.74 6.74 6.50 6.50 6.49 6.49 5.65 5.18 5.16 5.16 5.03 5.03 5.03 5.02 5.00 5.00 5.00 5.00 4.36 4.35 4.35 4.34 4.34 3.75 1.80 1.79 1.78 1.77 1.77 1.77 1.76 1.44 1.43 1.43 1.42 1.42 1.36 1.35 1.35 1.35 1.34 1.30 1.29 1.29 1.29 1.29 1.28 1.28 1.27 1.26 1.26 1.26 1.25 1.25 1.25
F_3C
O
O
HN
OMe
14i
1.92 1.94 2.23 2.50 0.43 1.95 0.45 1.53 0.22 0.78 0.21 0.77 0.23 0.78 2.03 2.92 0.28 1.66 1.04 1.01 1.02 3.09 1.08 0.93 1.07 2.21 2.66 10.12
0.0 9.5 9.0 8.5 8.0 7.5 7.0 6.5 6.0 5.5 5.0 4.5 4.0 3.5 3.0 2.5 2.0 1.5 1.0 0.5 0.0
[ppm]

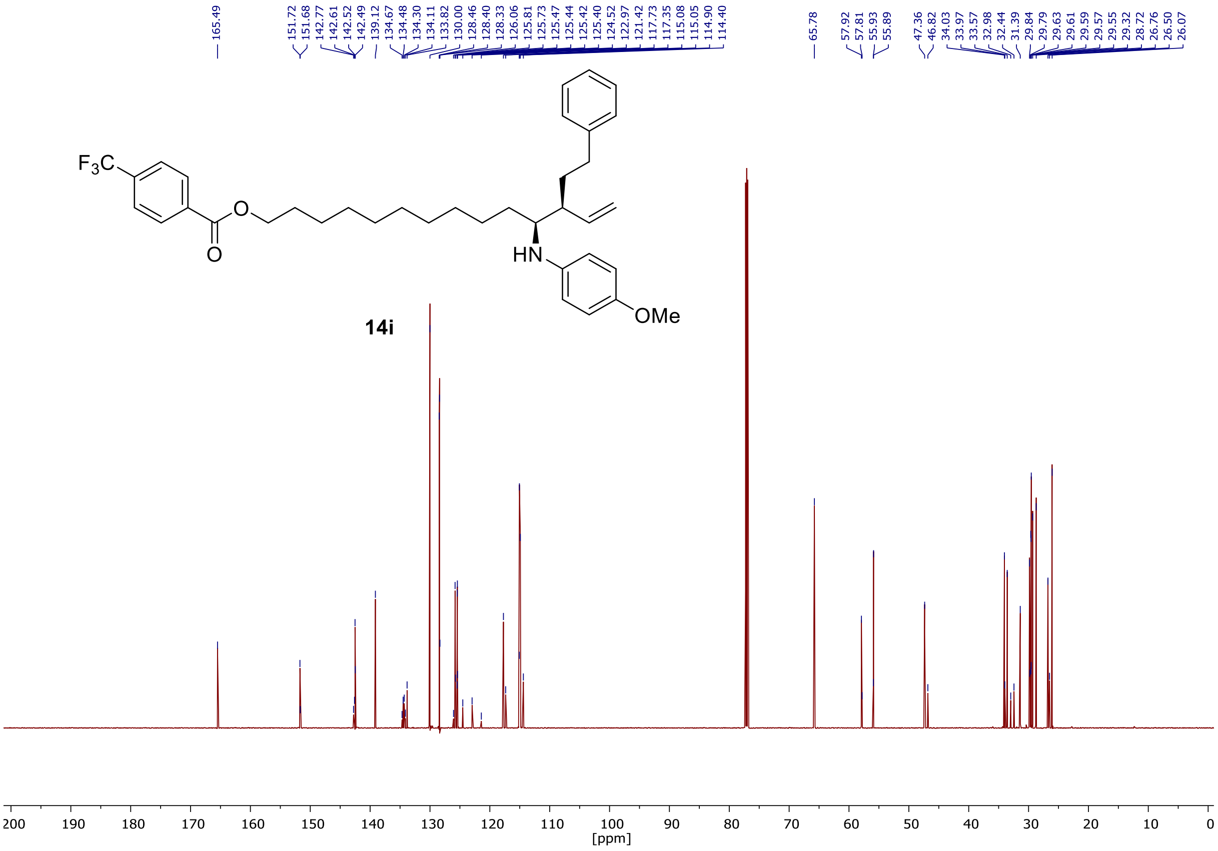
165.49
151.72
151.68
142.77
142.61
142.52
142.49
139.12
134.67
134.48
134.30
134.11
133.82
130.00
128.46
128.40
128.33
126.06
125.81
125.73
125.47
125.44
125.42
125.40
124.52
122.97
121.42
117.73
117.35
115.08
115.05
114.90
114.40
65.78
57.92
57.81
55.93
55.89
47.36
46.82
34.03
33.97
33.57
32.98
32.44
31.39
29.84
29.79
29.63
29.61
29.59
29.57
29.55
29.32
28.72
26.76
26.50
26.07
F3C
O
O
HN
OMe
14i
200 190 180 170 160 150 140 130 120 110 100 90 80 70 60 50 40 30 20 10 0
[ppm]

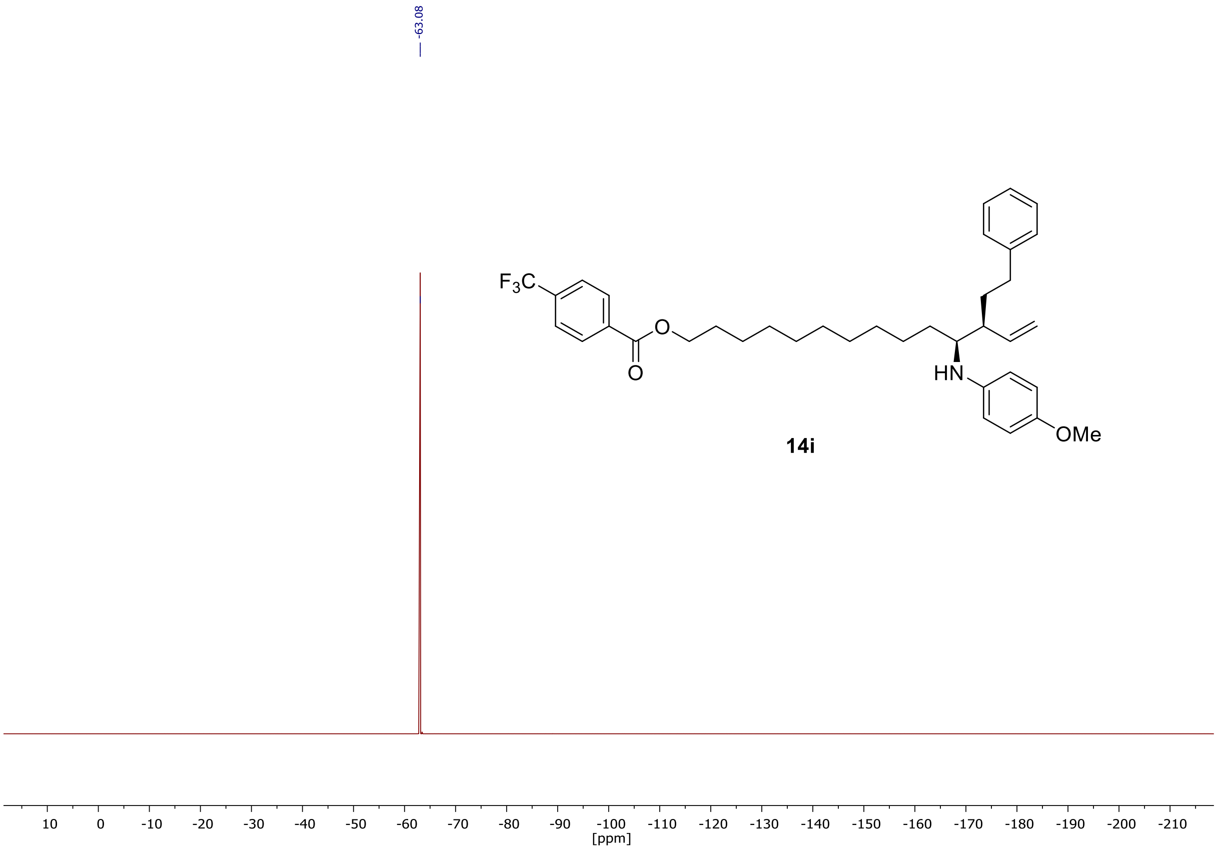
-63.08
F3C
O
O
HN
OMe
14i
10 0 -10 -20 -30 -40 -50 -60 -70 -80 -90 -100 -110 -120 -130 -140 -150 -160 -170 -180 -190 -200 -210
[ppm]

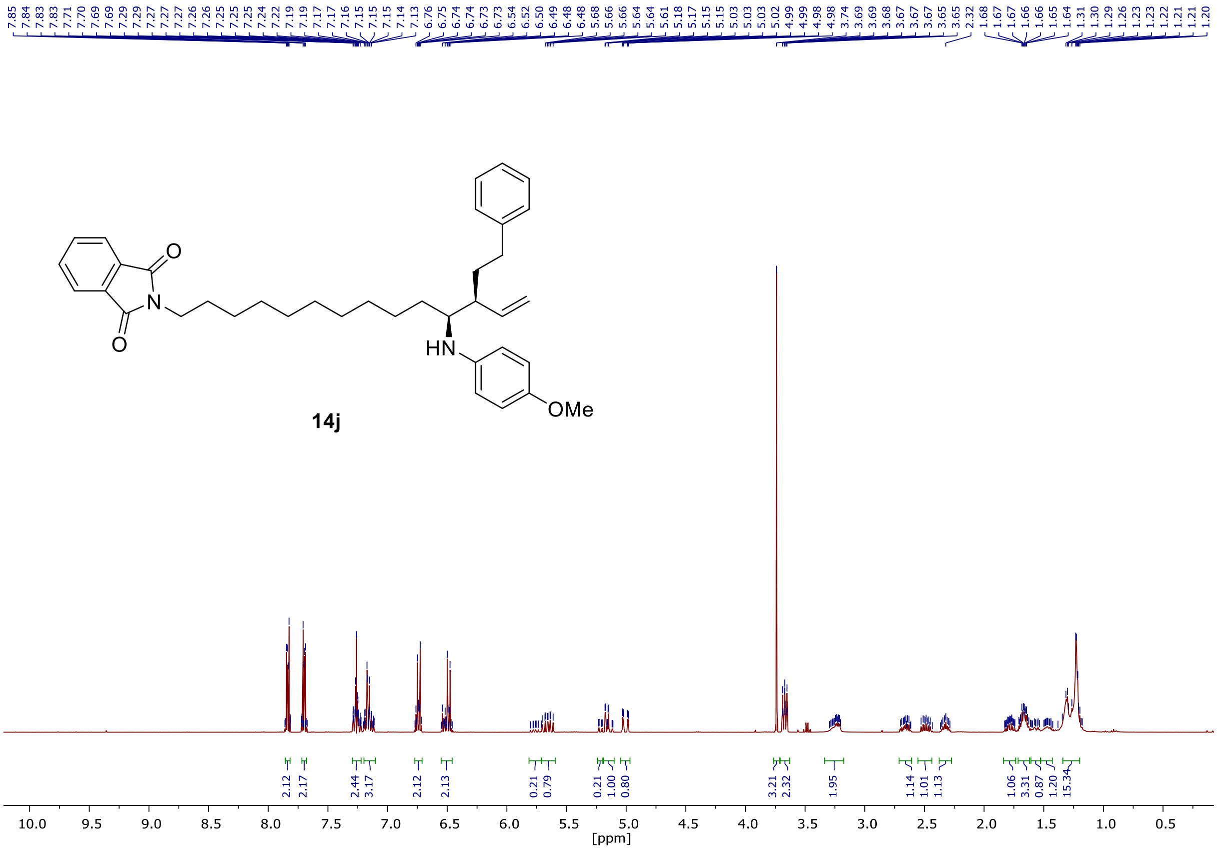
7.85 7.84 7.83 7.83 7.71 7.70 7.69 7.69 7.29 7.29 7.27 7.27 7.26 7.26 7.25 7.25 7.24 7.22 7.19 7.19 7.17 7.16 7.15 7.15 7.15 7.14 7.13 6.76 6.75 6.74 6.74 6.73 6.73 6.54 6.52 6.50 6.49 6.48 6.48 5.68 5.66 5.66 5.64 5.64 5.61 5.18 5.17 5.15 5.15 5.03 5.03 5.03 5.02 4.99 4.99 4.98 4.98 3.74 3.69 3.69 3.68 3.67 3.67 3.67 3.65 2.32 1.68 1.67 1.67 1.66 1.66 1.65 1.64 1.31 1.30 1.29 1.26 1.23 1.23 1.22 1.21 1.21 1.20
O
N
O
HN
OMe
14j
2.12 2.17 2.44 3.17 2.12 2.13 0.21 0.79 0.21 1.00 0.80 3.21 2.32 1.95 1.14 1.01 1.13 1.06 3.31 0.87 1.20 15.34
10.0 9.5 9.0 8.5 8.0 7.5 7.0 6.5 6.0 5.5 5.0 4.5 4.0 3.5 3.0 2.5 2.0 1.5 1.0 0.5
[ppm]

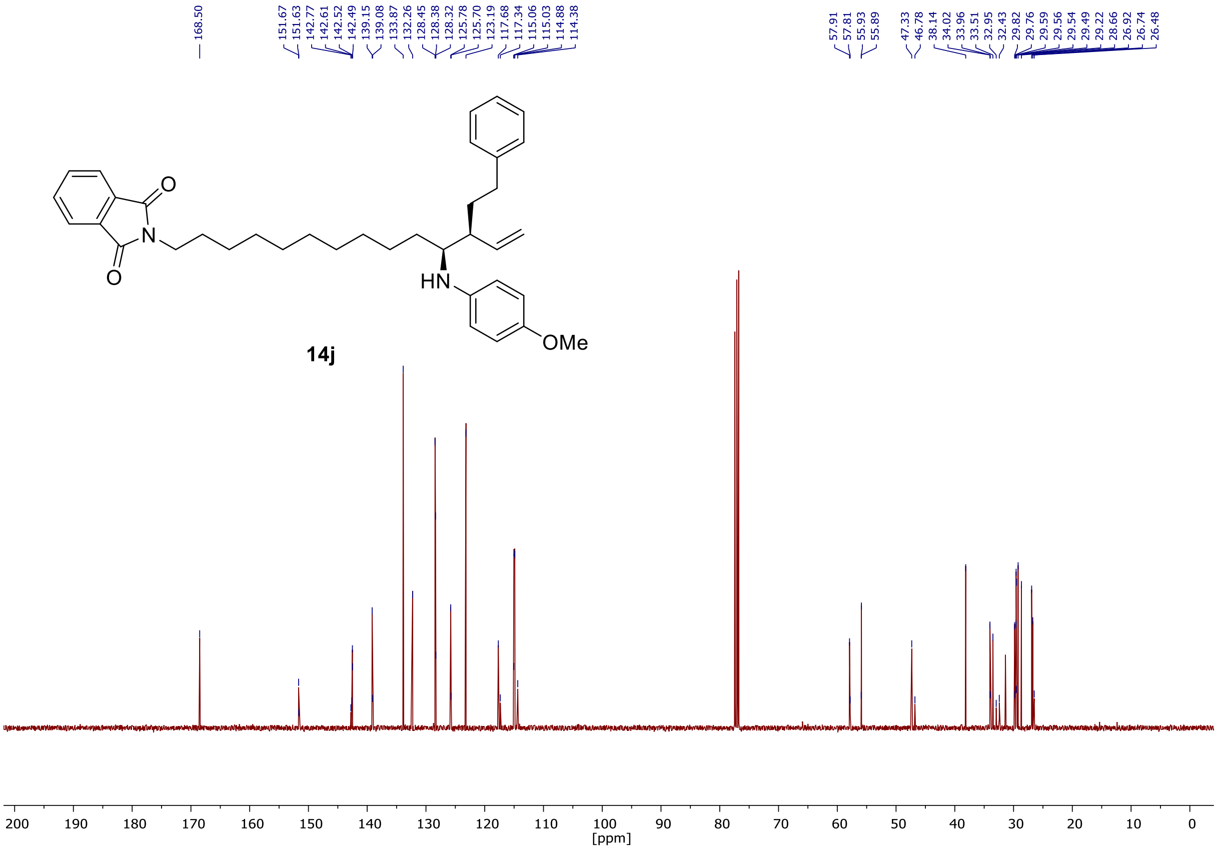
168.50
151.67 151.63 142.77 142.61 142.52 142.49 139.15 139.08 133.87 132.26 128.45 128.38 128.32 125.78 125.70 123.19 117.68 117.34 115.06 115.03 114.88 114.38
57.91 57.81 55.93 55.89 47.33 46.78 38.14 34.02 33.96 33.51 32.95 32.43 29.82 29.76 29.59 29.56 29.54 29.49 29.22 28.66 26.92 26.74 26.48
O
N
O
HN
OMe
14j
200 190 180 170 160 150 140 130 120 110 100 90 80 70 60 50 40 30 20 10 0
[ppm]

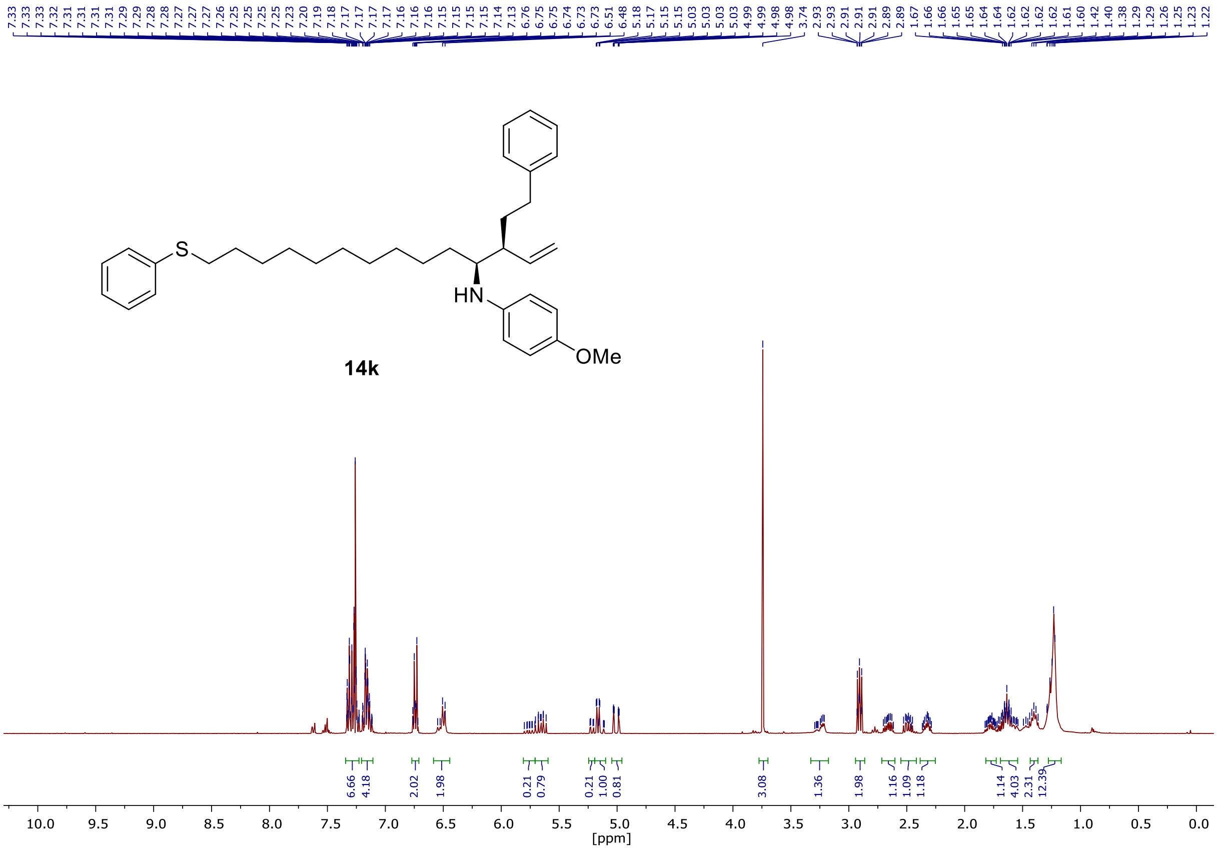
S
HN
OMe
14k
[ppm]

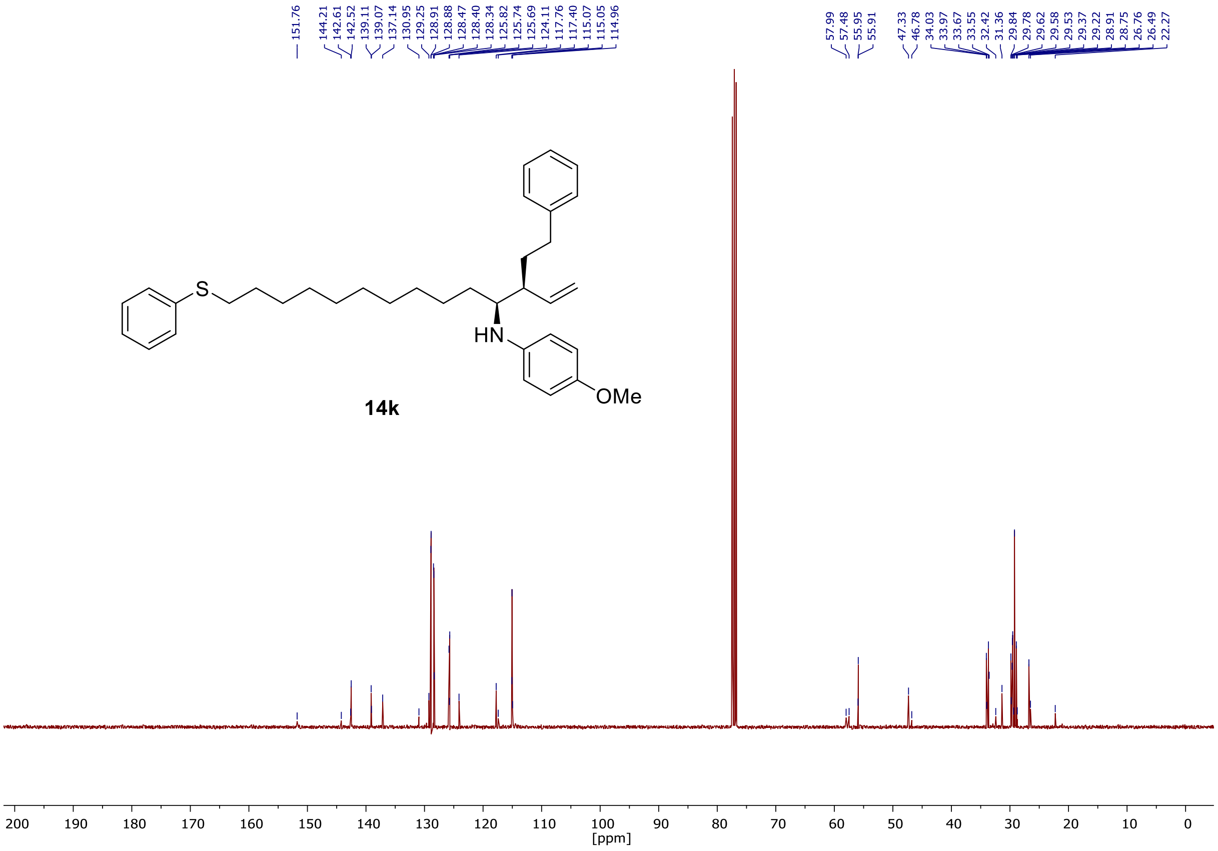
S
HN
OMe
14k
[ppm]

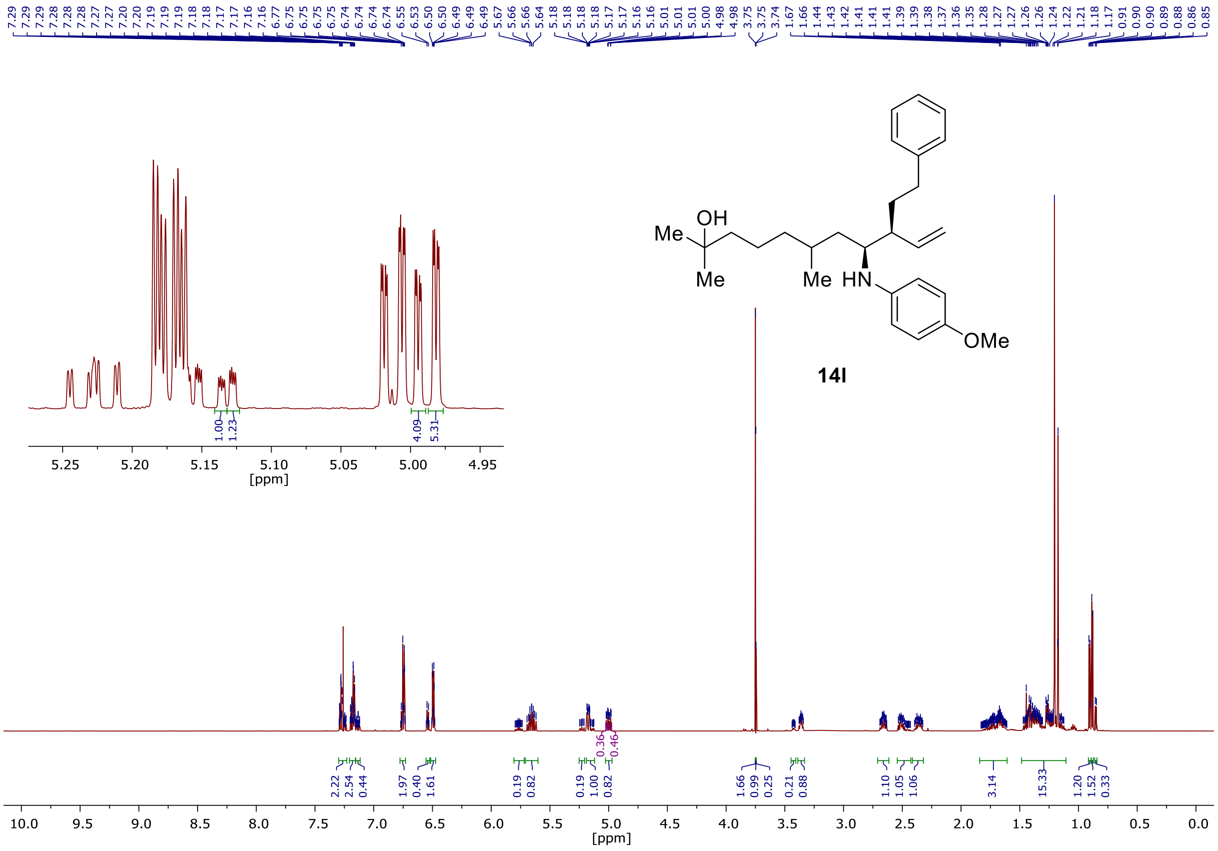

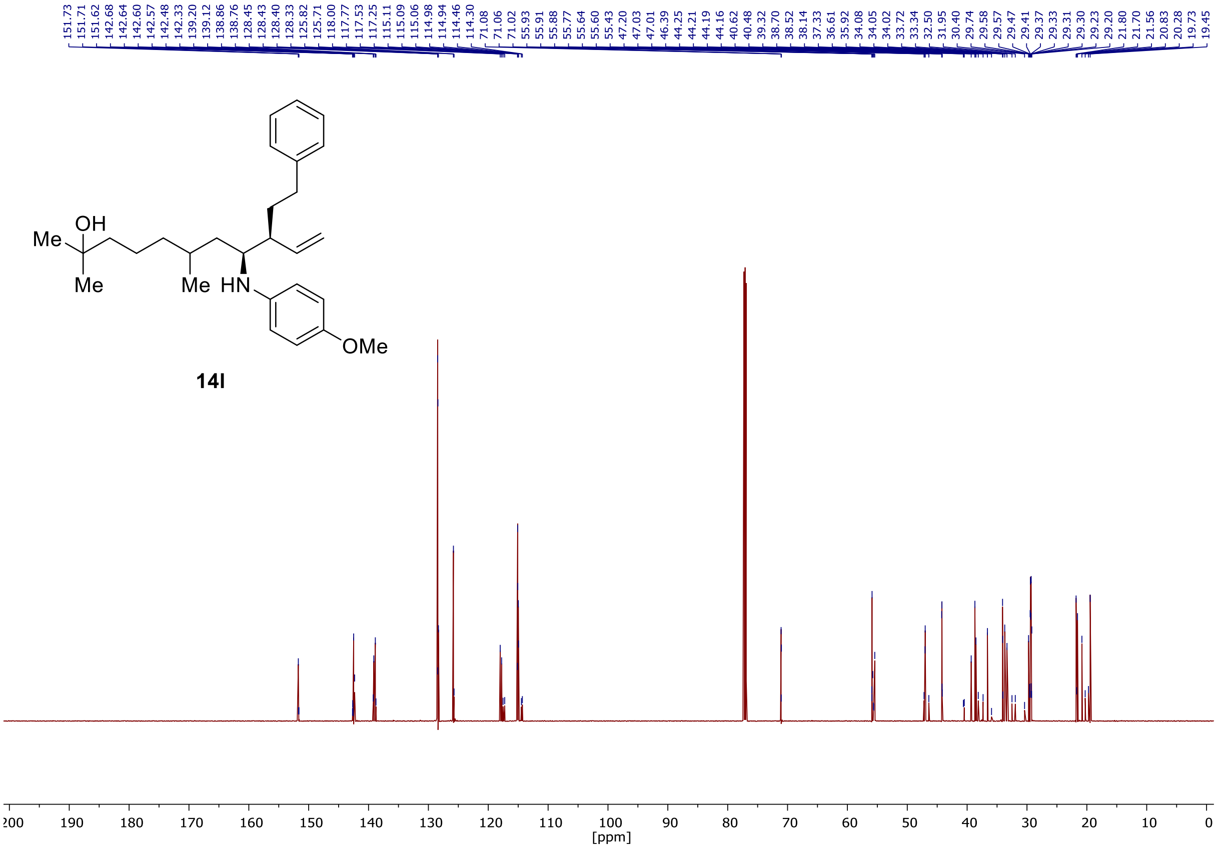

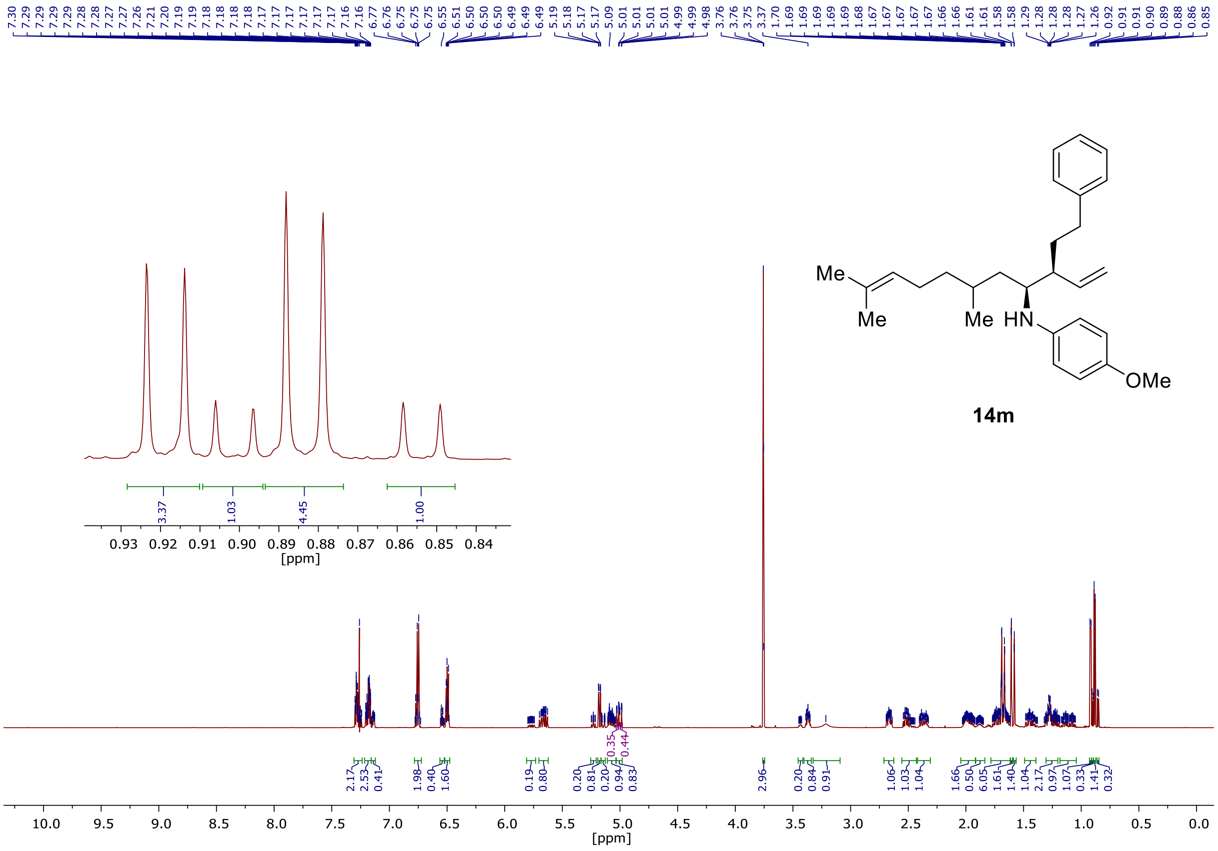
7.30 7.29 7.29 7.29 7.28 7.28 7.27 7.27 7.26 7.21 7.20 7.19 7.19 7.18 7.18 7.18 7.18 7.17 7.17 7.17 7.17 7.17 7.16 6.77 6.76 6.75 6.75 6.75 6.55 6.51 6.50 6.50 6.50 6.49 6.49 5.19 5.18 5.17 5.17 5.09 5.01 5.01 5.01 5.01 4.99 4.99 4.98 3.76 3.76 3.75 3.37 1.70 1.69 1.69 1.69 1.69 1.68 1.67 1.67 1.67 1.67 1.66 1.66 1.61 1.61 1.58 1.58 1.29 1.28 1.28 1.27 1.26 0.92 0.91 0.91 0.90 0.89 0.88 0.86 0.85
Me
Me
Me HN
OMe
14m
3.37
1.03
4.45
1.00
0.93 0.92 0.91 0.90 0.89 0.88 0.87 0.86 0.85 0.84
[ppm]
2.17 2.53 0.41 1.98 0.40 1.60 0.19 0.80 0.20 0.81 0.20 0.35 0.94 0.44 0.83 2.96 0.20 0.84 0.91 1.06 1.03 1.04 1.66 0.50 6.05 1.61 1.40 1.04 2.17 0.97 1.07 0.33 1.41 0.32
10.0 9.5 9.0 8.5 8.0 7.5 7.0 6.5 6.0 5.5 5.0 4.5 4.0 3.5 3.0 2.5 2.0 1.5 1.0 0.5 0.0
[ppm]

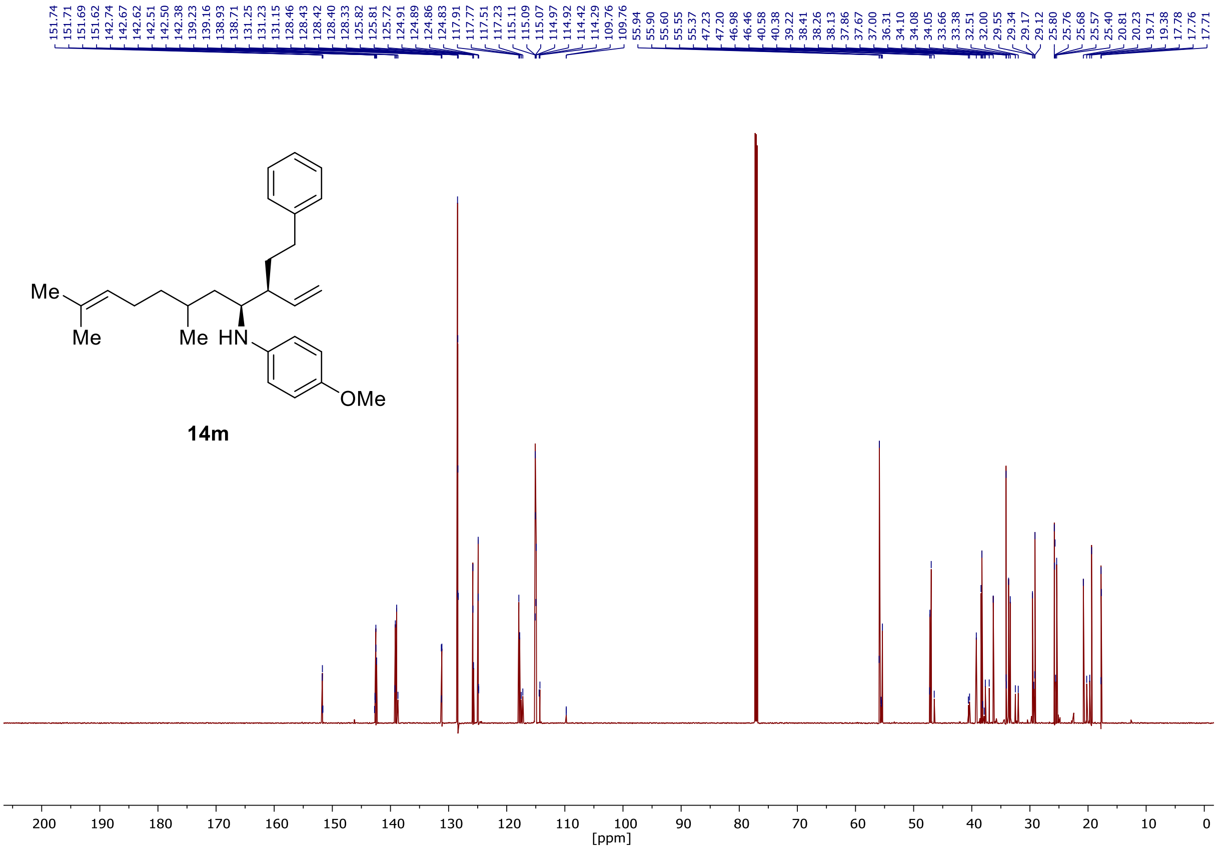
151.74 151.71 151.69 151.62 142.74 142.67 142.62 142.51 142.50 142.38 139.23 139.16 138.93 138.71 131.25 131.23 131.15 128.46 128.43 128.42 128.40 128.33 125.82 125.81 125.72 124.91 124.89 124.86 124.83 117.91 117.77 117.51 117.23 115.11 115.09 115.07 114.97 114.92 114.42 114.29 109.76 109.76 55.94 55.90 55.60 55.55 55.37 47.23 47.20 46.98 46.46 40.58 40.38 39.22 38.41 38.26 38.13 37.86 37.67 37.00 36.31 34.10 34.08 34.05 33.66 33.38 32.51 32.00 29.55 29.34 29.17 29.12 25.80 25.76 25.68 25.57 25.40 20.81 20.23 19.71 19.38 17.78 17.76 17.71
Me
Me
Me HN
OMe
14m
200 190 180 170 160 150 140 130 120 110 100 90 80 70 60 50 40 30 20 10 0
[ppm]

Mechanistic Experiments

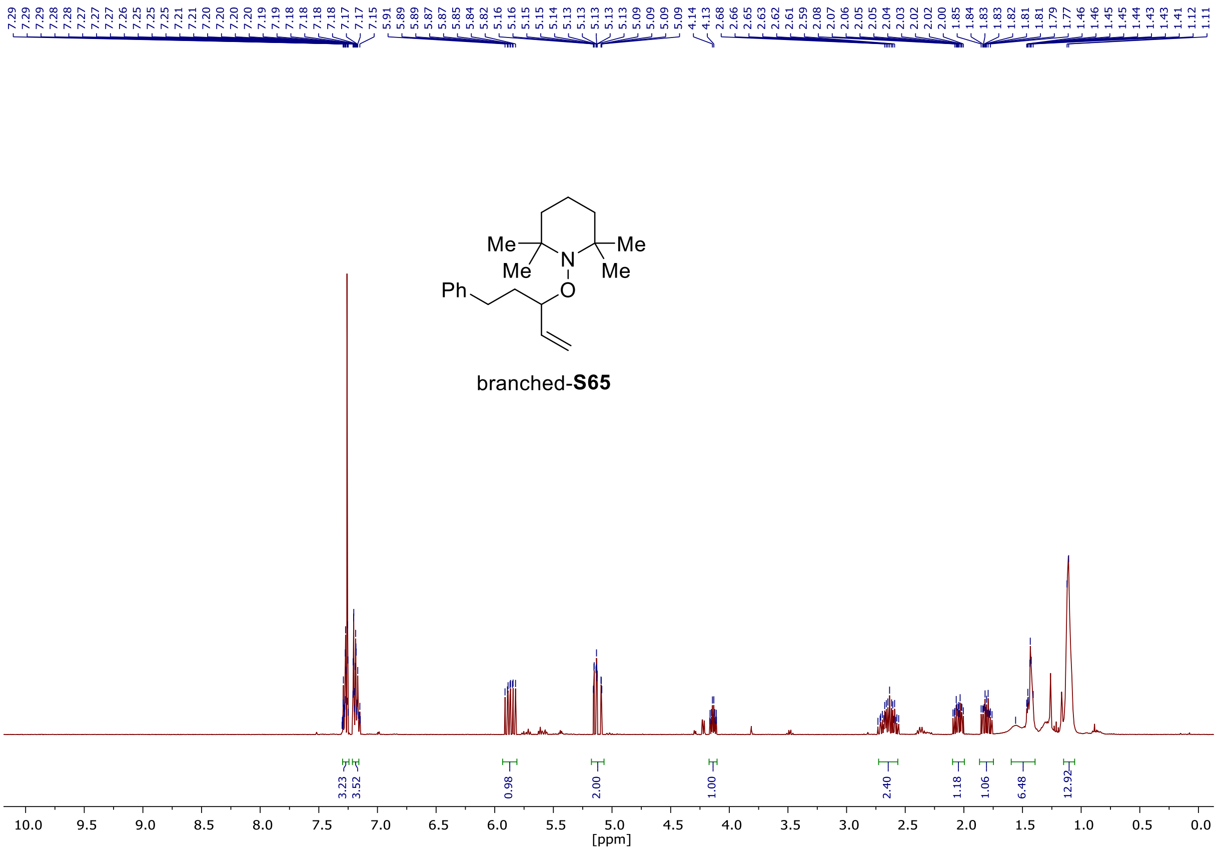

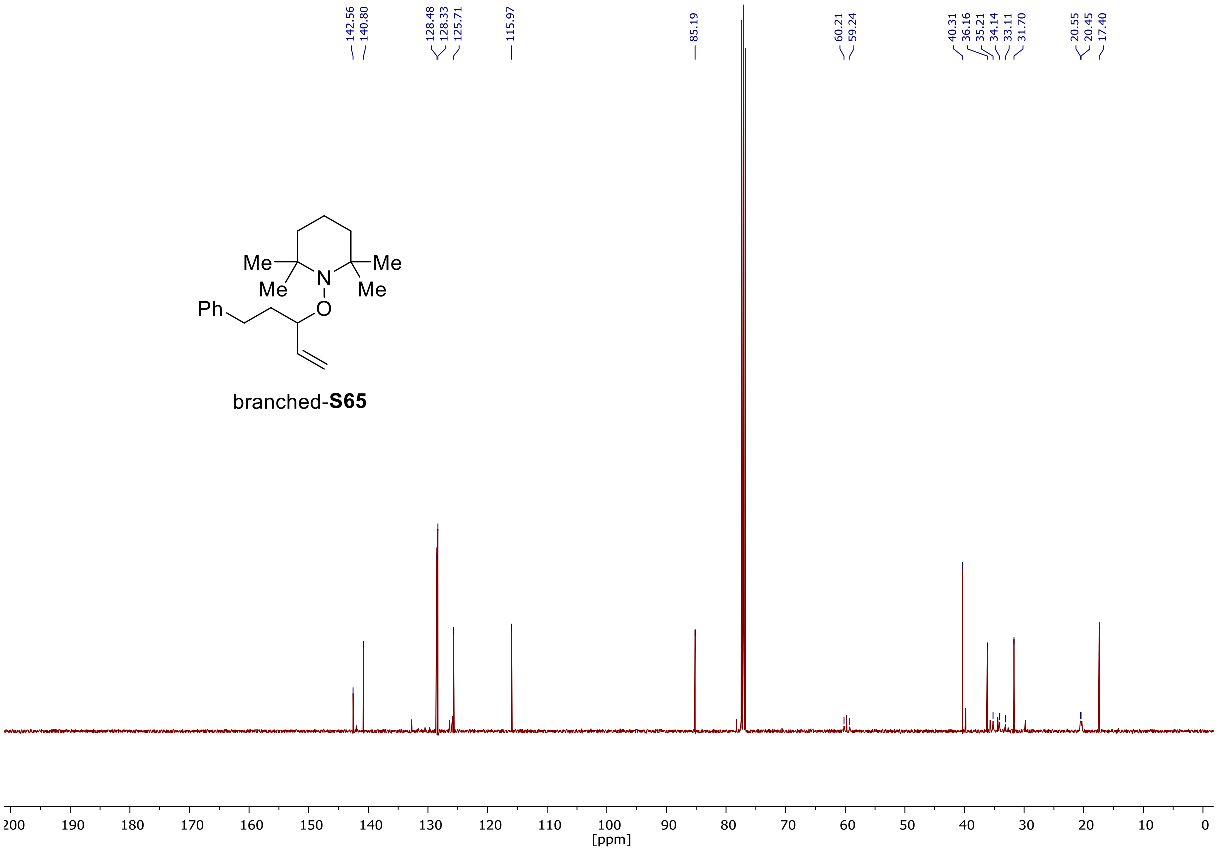

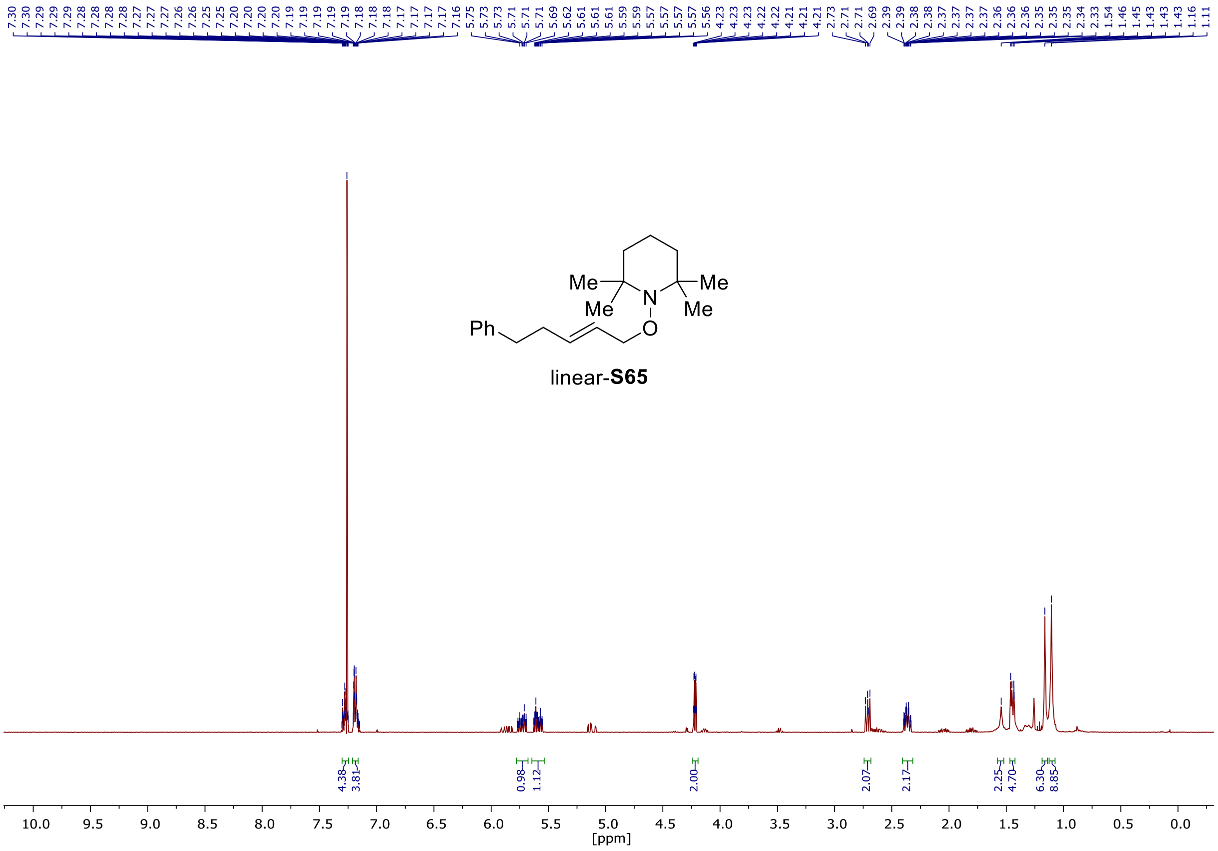
7.30 7.30 7.29 7.29 7.29 7.28 7.28 7.28 7.27 7.27 7.26 7.25 7.25 7.20 7.20 7.20 7.19 7.19 7.19 7.19 7.19 7.18 7.18 7.17 7.17 7.17 7.16 5.75 5.73 5.73 5.71 5.71 5.71 5.69 5.62 5.61 5.61 5.59 5.59 5.57 5.57 5.57 5.56 4.23 4.23 4.23 4.22 4.22 4.21 4.21 2.73 2.71 2.71 2.69 2.39 2.39 2.38 2.38 2.37 2.37 2.37 2.36 2.36 2.35 2.35 2.34 2.33 1.54 1.46 1.45 1.43 1.43 1.43 1.16 1.11
Me
Me
N
O
Me
Me
Ph
linear-**S65**
4.38
3.81
0.98
1.12
2.00
2.07
2.17
2.25
4.70
6.30
8.85
10.0 9.5 9.0 8.5 8.0 7.5 7.0 6.5 6.0 5.5 5.0 4.5 4.0 3.5 3.0 2.5 2.0 1.5 1.0 0.5 0.0
[ppm]

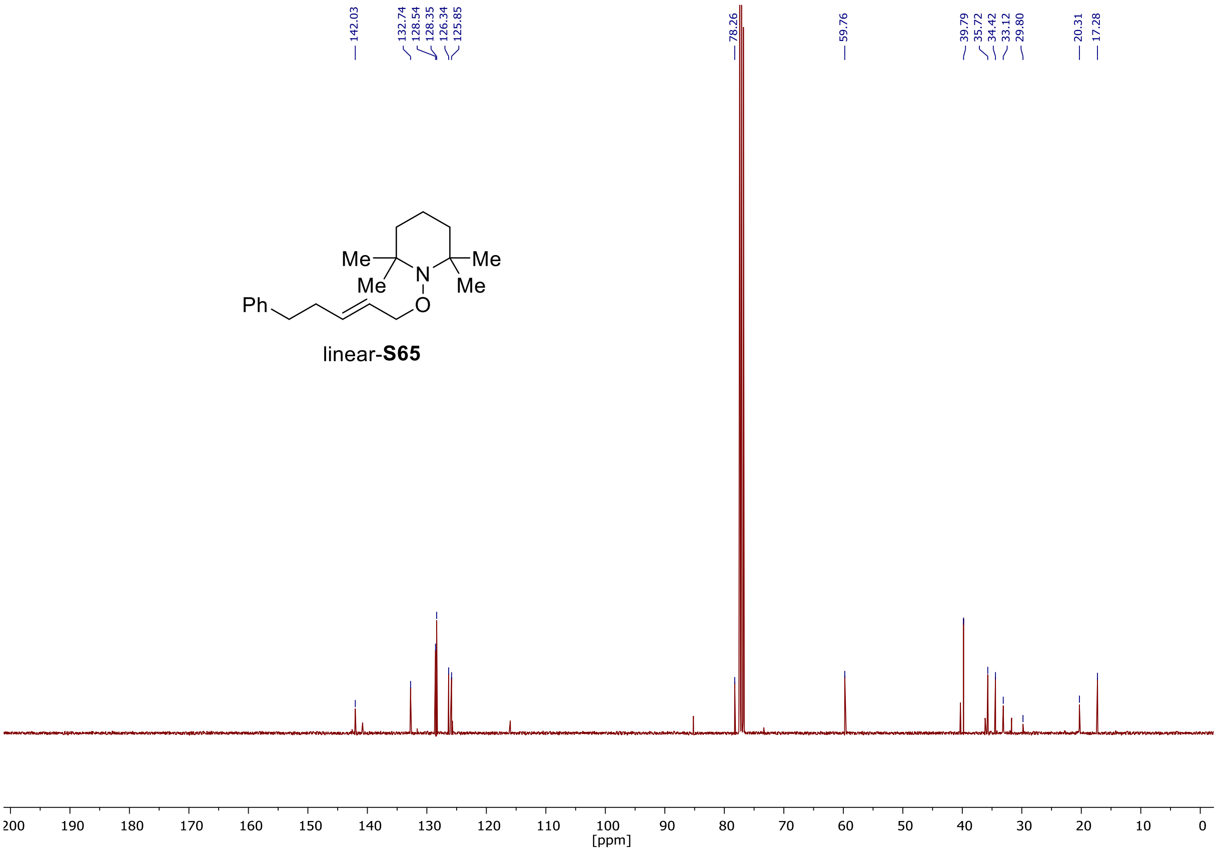
142.03
132.74 128.54 128.35 126.34 125.85
78.26
59.76
39.79 35.72 34.42 33.12 29.80
20.31 17.28
Me
Me
N
O
Me
Me
Ph
linear-**S65**
200 190 180 170 160 150 140 130 120 110 100 90 80 70 60 50 40 30 20 10 0
[ppm]

Follow-Up Chemistry

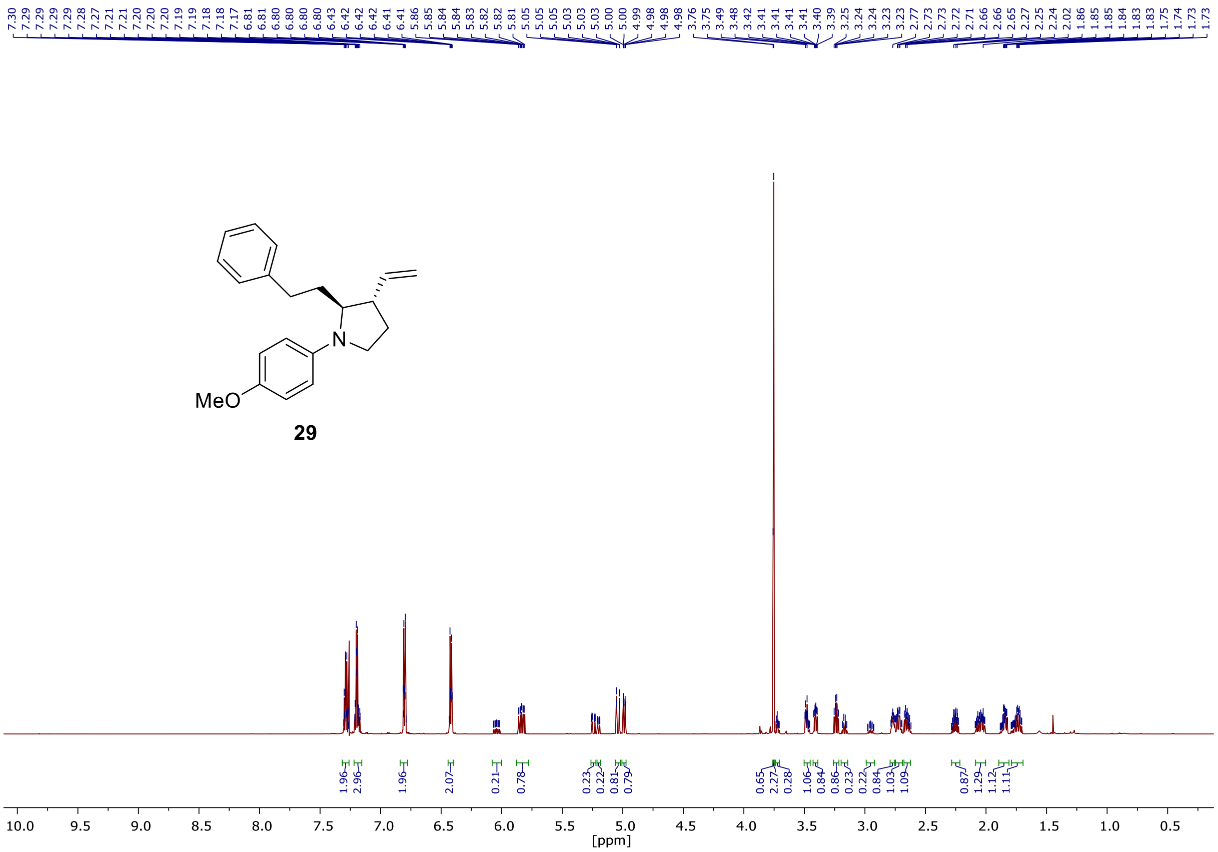

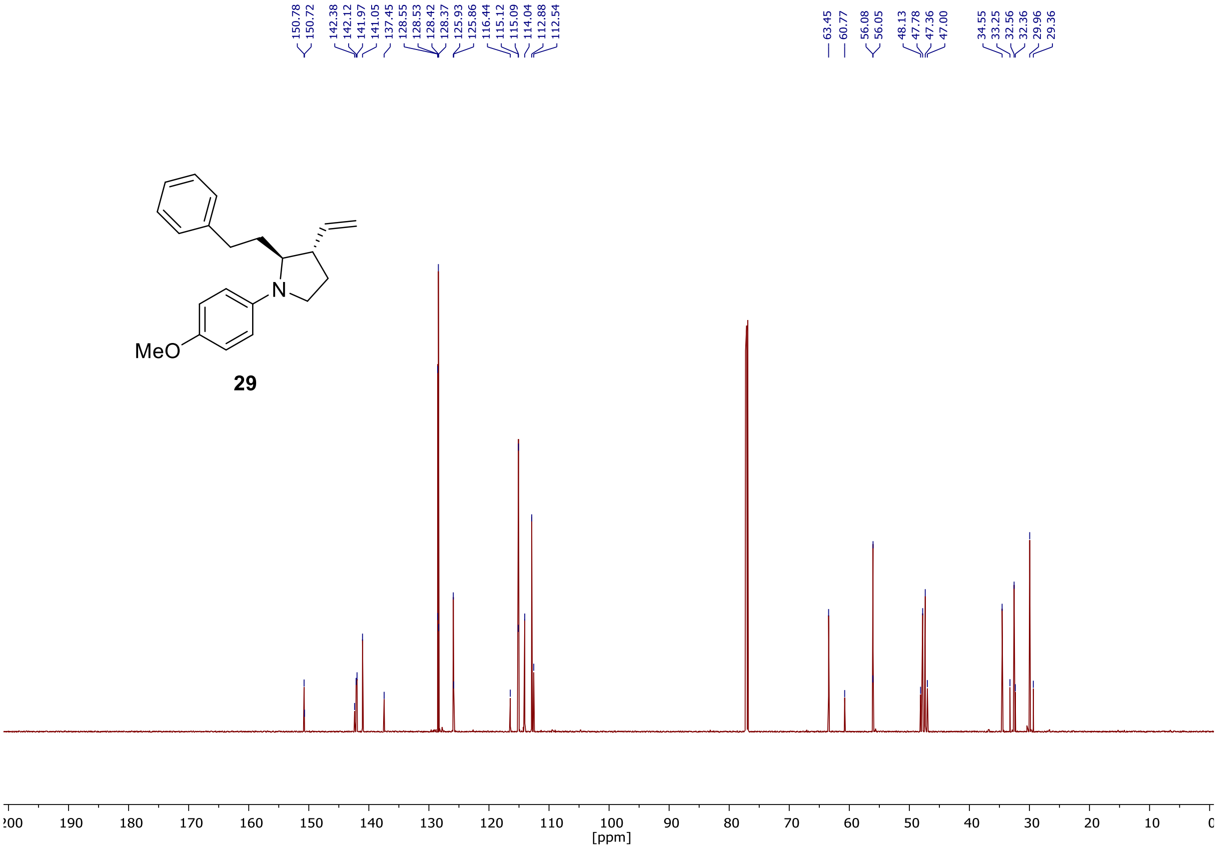

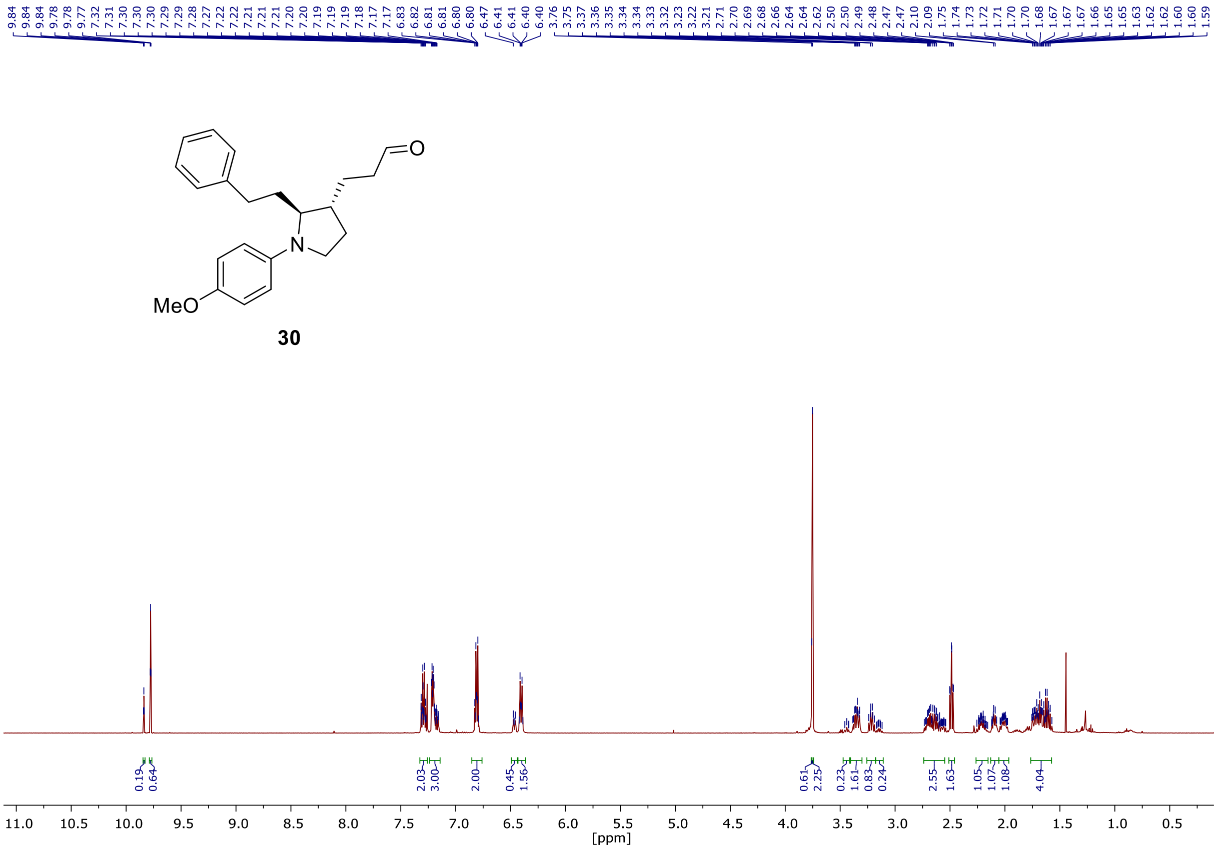
MeO
N
O
30
[ppm]

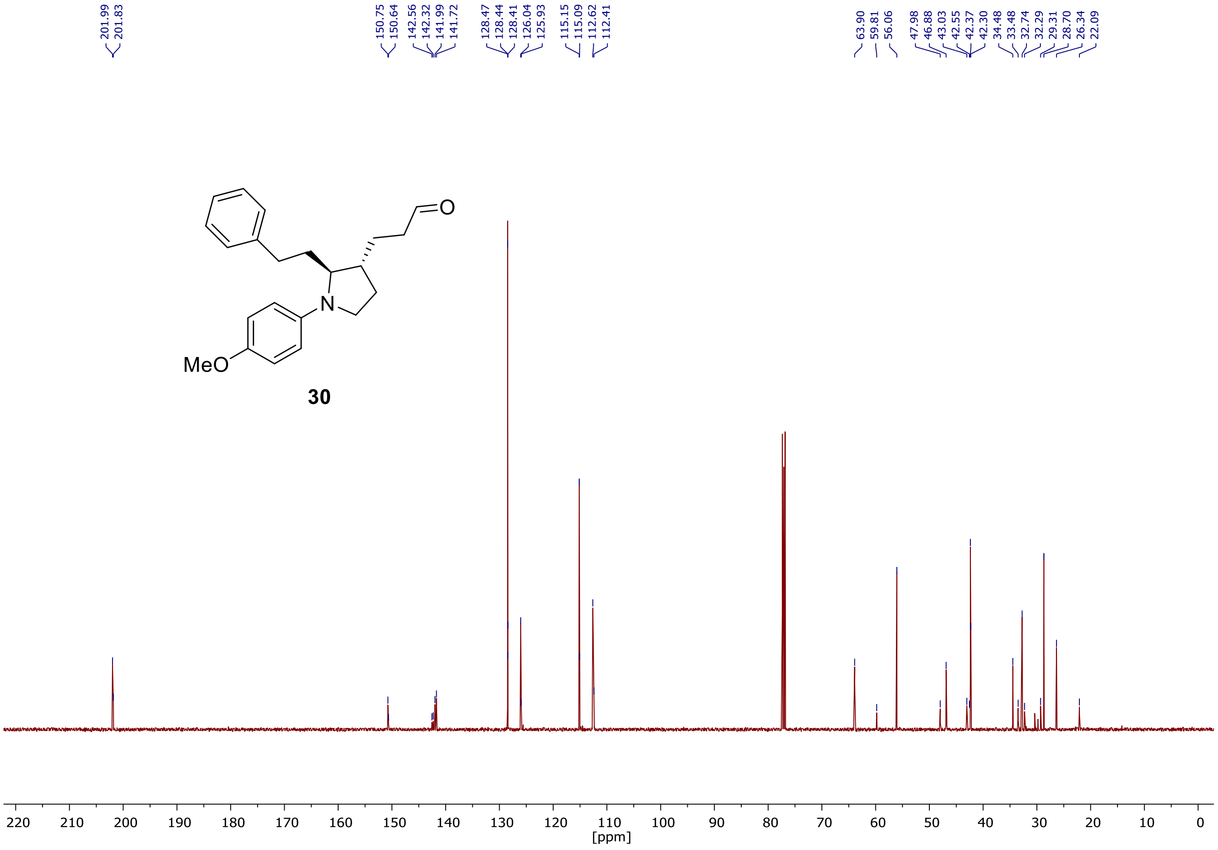
MeO
N
O
30
[ppm]

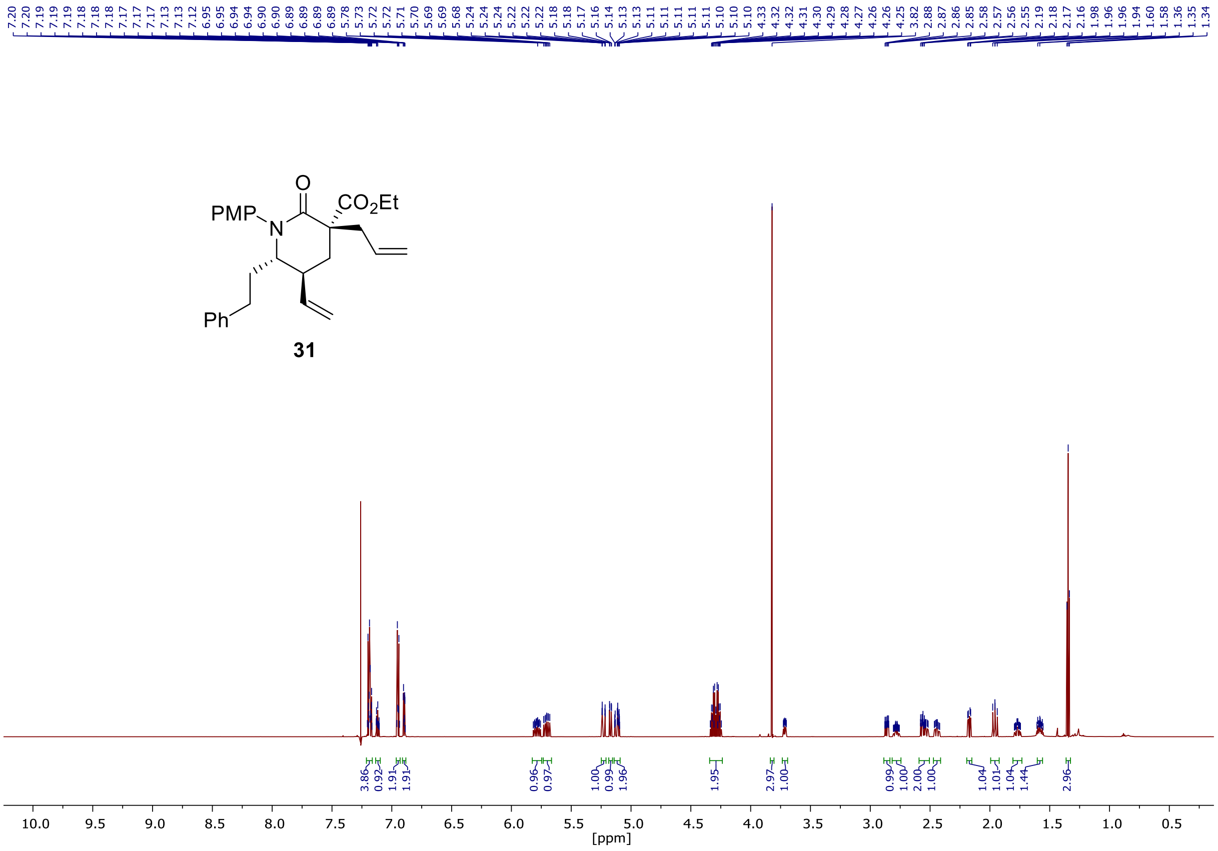
7.20 7.20 7.19 7.19 7.19 7.18 7.18 7.18 7.17 7.17 7.17 7.13 7.12 6.95 6.95 6.94 6.94 6.90 6.90 6.89 6.89 6.89 6.89 5.78 5.73 5.72 5.72 5.71 5.70 5.69 5.69 5.68 5.24 5.24 5.24 5.22 5.22 5.22 5.18 5.18 5.17 5.16 5.14 5.13 5.13 5.11 5.11 5.11 5.11 5.11 5.10 5.10 5.10 4.33 4.32 4.32 4.31 4.30 4.29 4.28 4.27 4.26 4.26 4.25 3.82 2.88 2.87 2.86 2.85 2.58 2.57 2.56 2.55 2.19 2.18 2.17 2.16 1.98 1.96 1.96 1.94 1.60 1.58 1.36 1.35 1.34
PMP
N
O
CO_2Et
Ph
31
3.86 0.92 1.91 1.91 0.96 0.97 1.00 0.99 1.96 1.95 2.97 1.00 0.99 1.00 2.00 1.00 1.04 1.01 1.04 1.44 2.96
10.0 9.5 9.0 8.5 8.0 7.5 7.0 6.5 6.0 5.5 5.0 4.5 4.0 3.5 3.0 2.5 2.0 1.5 1.0 0.5
[ppm]

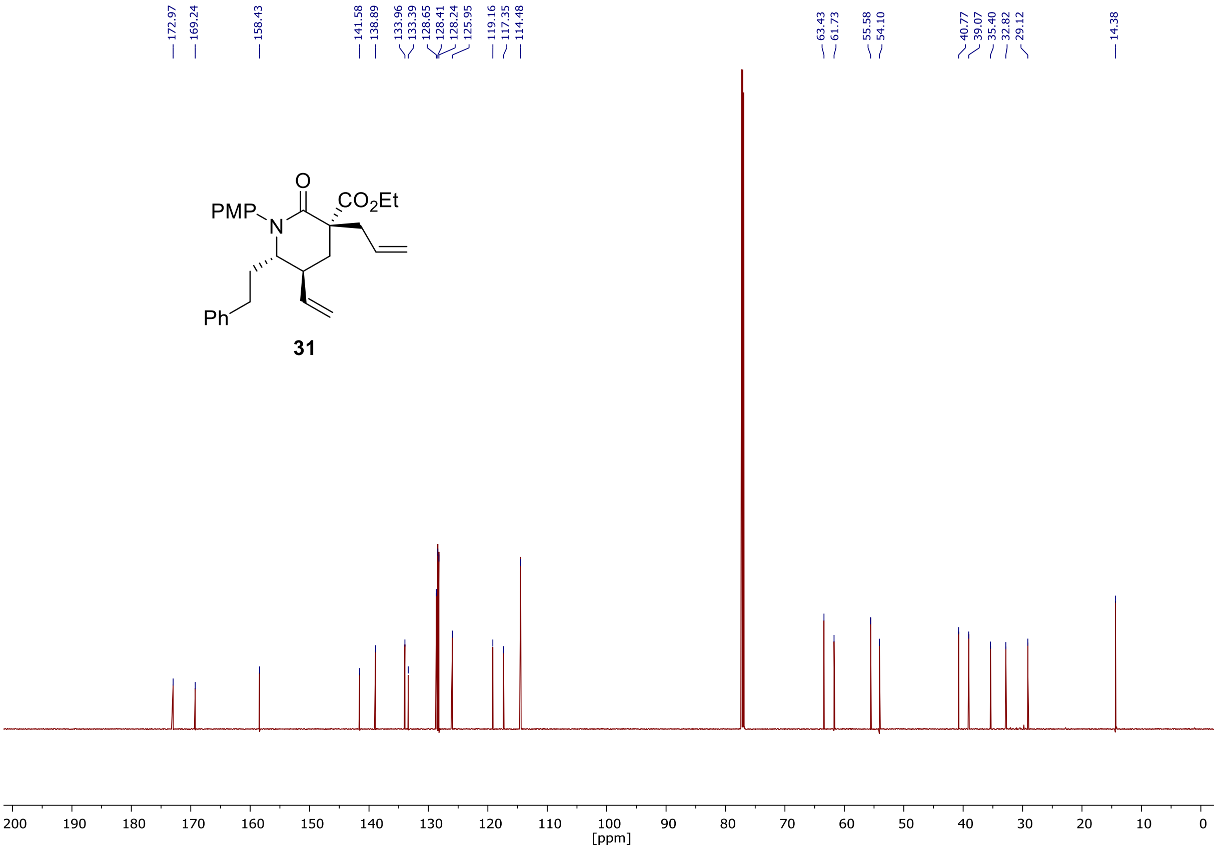
172.97 169.24 158.43 141.58 138.89 133.96 133.39 128.65 128.41 128.24 125.95 119.16 117.35 114.48 63.43 61.73 55.58 54.10 40.77 39.07 35.40 32.82 29.12 14.38
PMP
N
O
CO_2Et
Ph
31
200 190 180 170 160 150 140 130 120 110 100 90 80 70 60 50 40 30 20 10 0
[ppm]

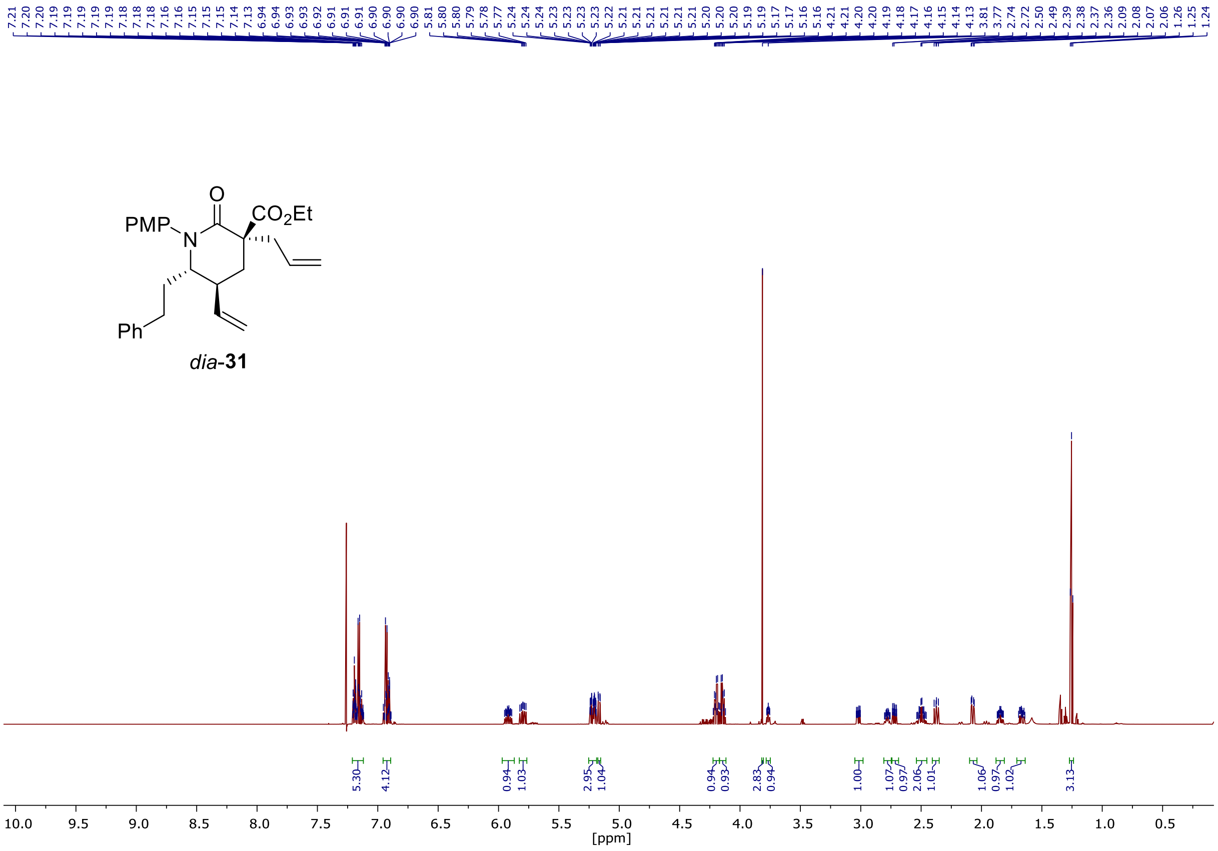
7.21 7.20 7.20 7.19 7.19 7.19 7.19 7.18 7.18 7.16 7.16 7.15 7.15 7.14 7.13 6.94 6.94 6.93 6.93 6.92 6.91 6.91 6.91 6.90 6.90 6.90 5.81 5.80 5.80 5.79 5.78 5.77 5.24 5.24 5.24 5.23 5.23 5.23 5.22 5.21 5.21 5.21 5.21 5.21 5.20 5.20 5.20 5.19 5.19 5.17 5.17 5.16 5.16 4.21 4.21 4.20 4.20 4.19 4.18 4.17 4.16 4.15 4.14 4.13 3.81 3.77 2.74 2.72 2.72 2.50 2.49 2.39 2.38 2.37 2.36 2.09 2.08 2.07 2.06 1.26 1.25 1.24
PMP
N
O
CO2Et
Ph
dia-31
5.30
4.12
0.94
1.03
2.95
1.04
0.94
0.93
2.83
0.94
1.00
1.07
0.97
2.06
1.01
1.06
0.97
1.02
3.13
[ppm]

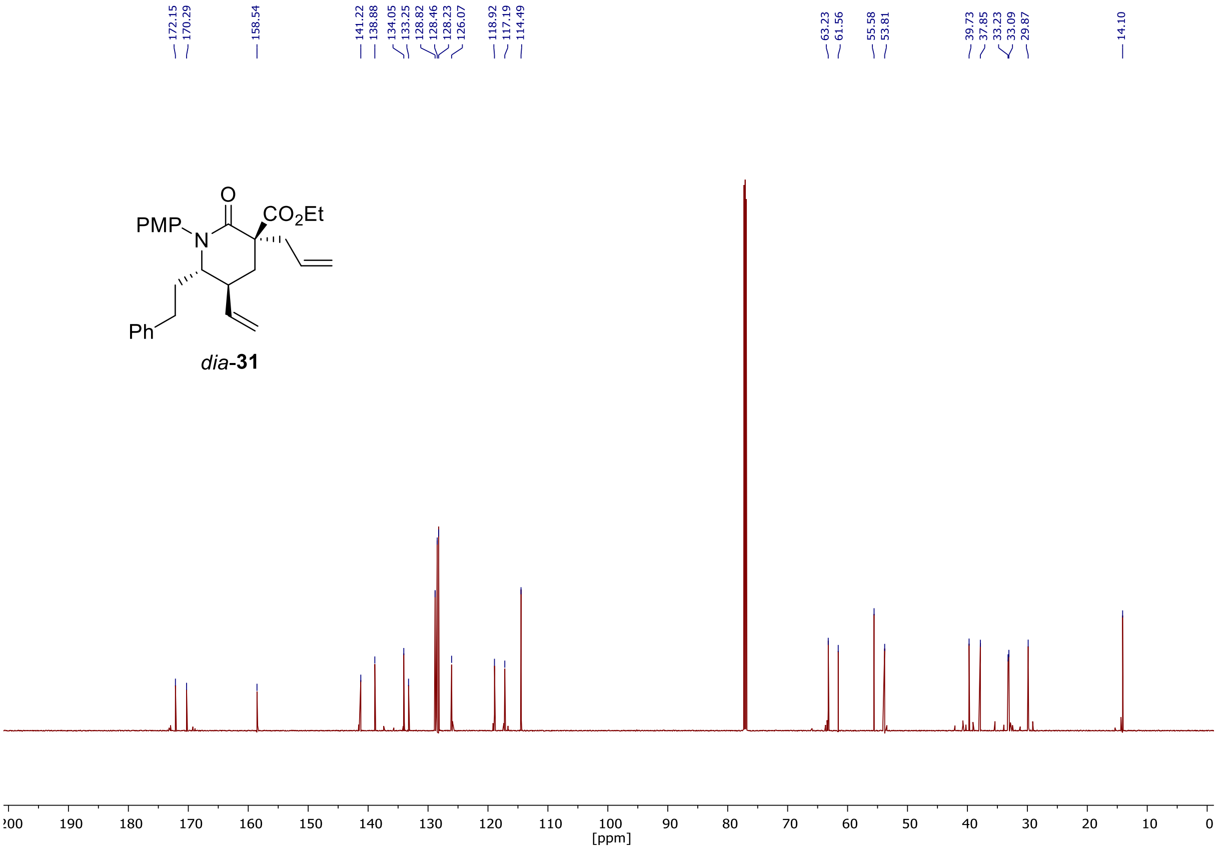
172.15 170.29 158.54 141.22 138.88 134.05 133.25 128.82 128.46 128.23 126.07 118.92 117.19 114.49 63.23 61.56 55.58 53.81 39.73 37.85 33.23 33.09 29.87 14.10
PMP
N
O
CO2Et
Ph
dia-31
[ppm]

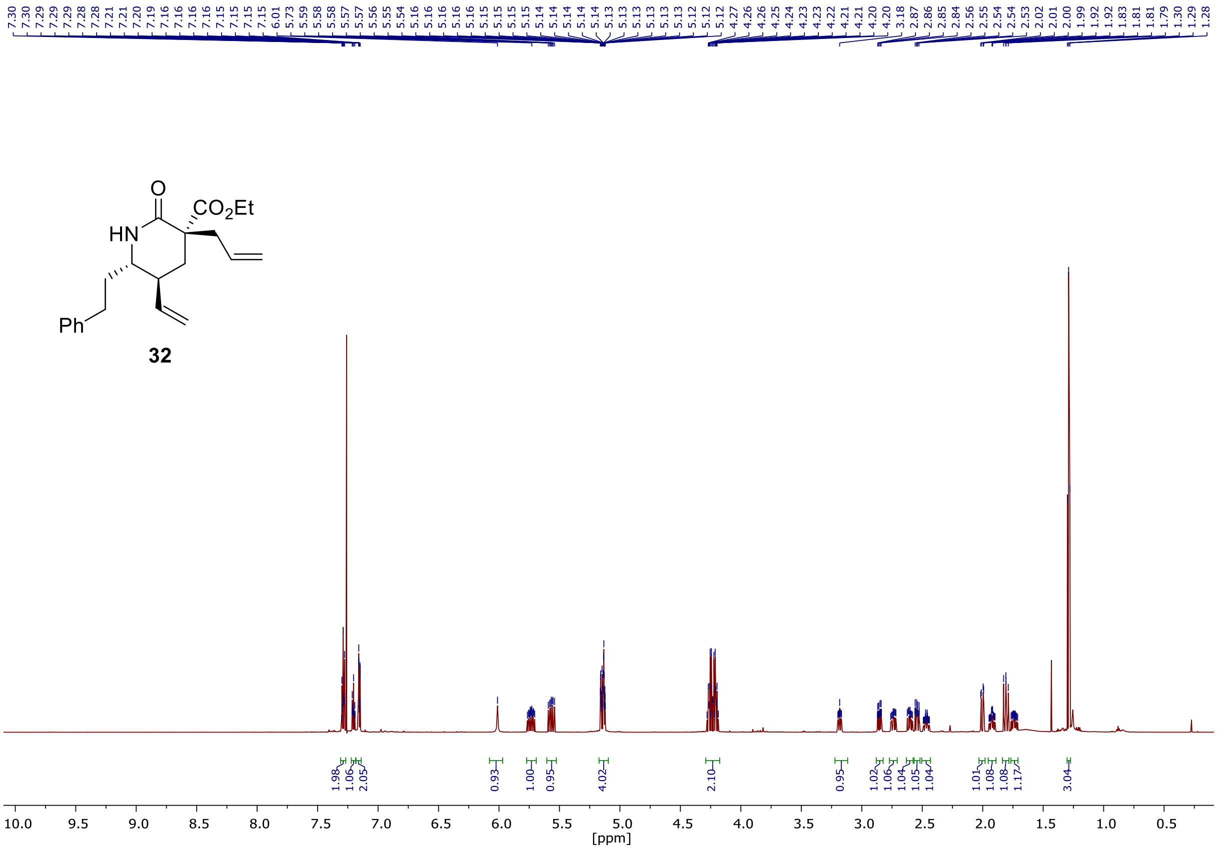
7.30
7.30
7.29
7.29
7.29
7.28
7.28
7.21
7.21
7.20
7.19
7.16
7.16
7.16
7.15
7.15
7.15
7.15
6.01
5.73
5.59
5.58
5.58
5.57
5.57
5.56
5.55
5.54
5.16
5.16
5.16
5.16
5.16
5.15
5.15
5.15
5.15
5.14
5.14
5.14
5.14
5.13
5.13
5.13
5.13
5.13
5.13
5.12
5.12
5.12
4.27
4.26
4.25
4.25
4.24
4.23
4.23
4.22
4.21
4.21
4.20
4.20
3.18
2.87
2.86
2.85
2.84
2.56
2.55
2.54
2.54
2.53
2.02
2.01
2.00
1.99
1.92
1.92
1.83
1.81
1.81
1.79
1.30
1.29
1.28
O
HN
CO2Et
Ph
32
1.98
1.06
2.05
0.93
1.00
0.95
4.02
2.10
0.95
1.02
1.06
1.04
1.05
1.04
1.01
1.08
1.08
1.17
3.04
10.0 9.5 9.0 8.5 8.0 7.5 7.0 6.5 6.0 5.5 5.0 4.5 4.0 3.5 3.0 2.5 2.0 1.5 1.0 0.5
[ppm]

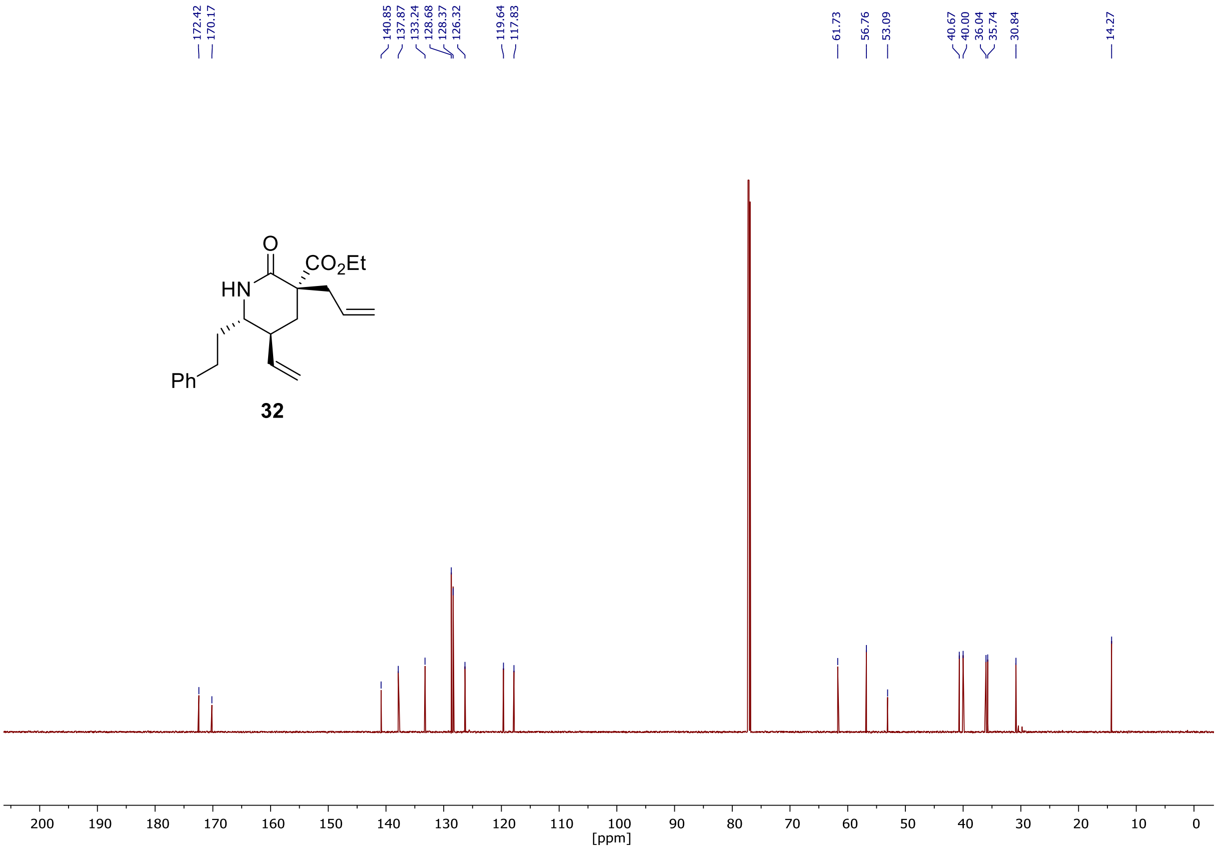
172.42
170.17
140.85
137.87
133.24
128.68
128.37
126.32
119.64
117.83
61.73
56.76
53.09
40.67
40.00
36.04
35.74
30.84
14.27
O
HN
CO2Et
Ph
32
200 190 180 170 160 150 140 130 120 110 100 90 80 70 60 50 40 30 20 10 0
[ppm]

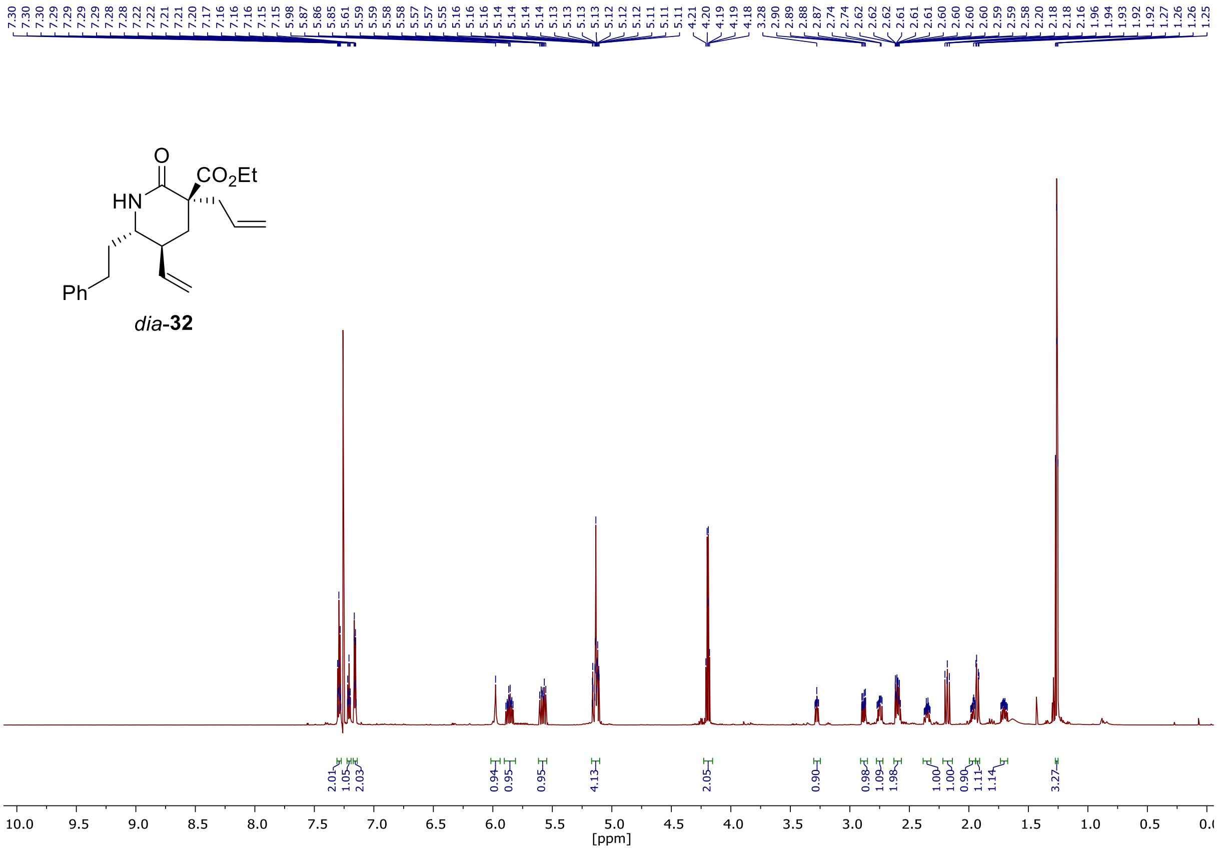
7.30 7.30 7.30 7.29 7.29 7.29 7.29 7.28 7.28 7.22 7.22 7.21 7.21 7.20 7.17 7.16 7.16 7.16 7.15 7.15 5.98 5.87 5.86 5.85 5.61 5.59 5.59 5.58 5.58 5.57 5.57 5.55 5.16 5.16 5.16 5.14 5.14 5.14 5.14 5.13 5.13 5.13 5.13 5.13 5.12 5.12 5.12 5.11 5.11 5.11 4.21 4.20 4.19 4.19 4.18 3.28 2.90 2.89 2.88 2.87 2.74 2.74 2.62 2.62 2.62 2.61 2.61 2.61 2.60 2.60 2.60 2.60 2.59 2.58 2.20 2.18 2.18 2.16 1.96 1.94 1.93 1.92 1.92 1.27 1.26 1.25
O
CO2Et
HN
Ph
dia-32
2.01
1.05
2.03
0.94
0.95
0.95
4.13
2.05
0.90
0.98
1.09
1.98
1.00
1.00
0.90
1.11
1.14
3.27
[ppm]

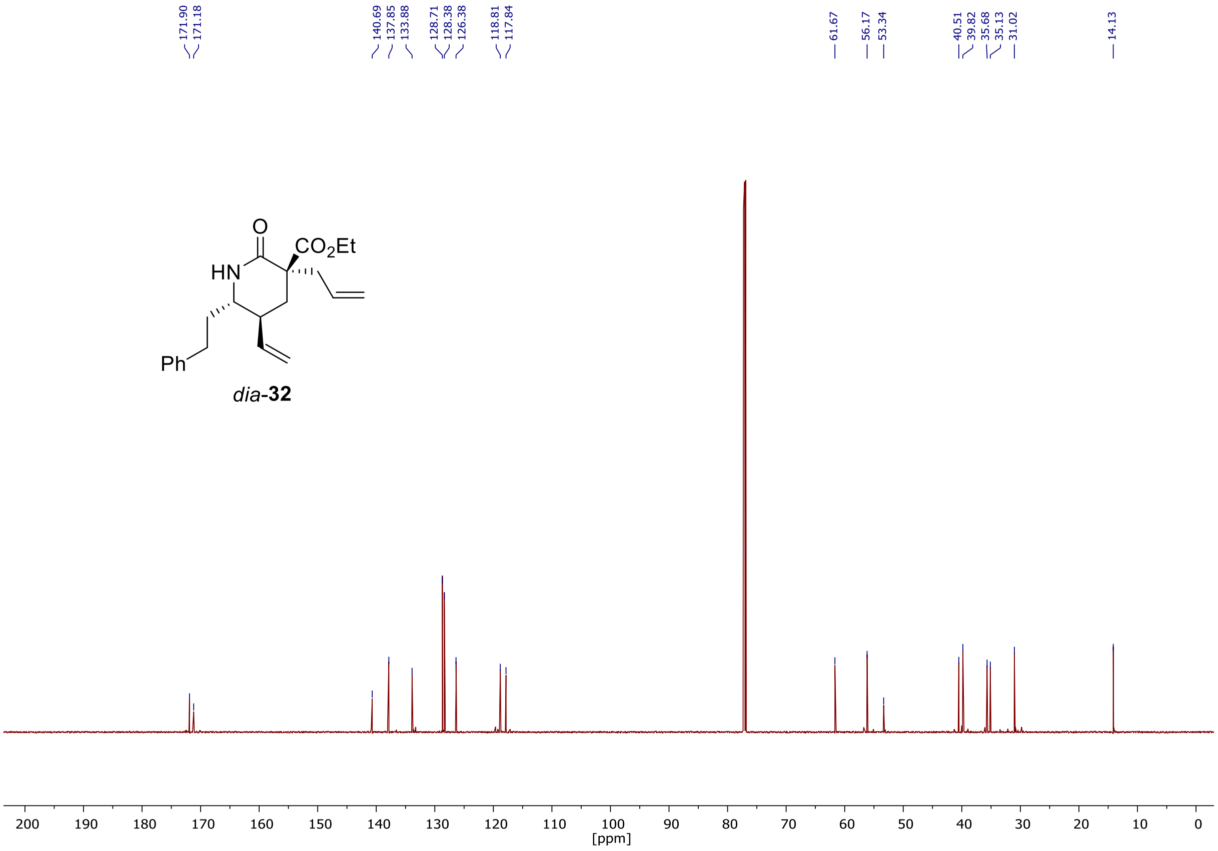
171.90 171.18 140.69 137.85 133.88 128.71 128.38 126.38 118.81 117.84 61.67 56.17 53.34 40.51 39.82 35.68 35.13 31.02 14.13
O
CO2Et
HN
Ph
dia-32
[ppm]

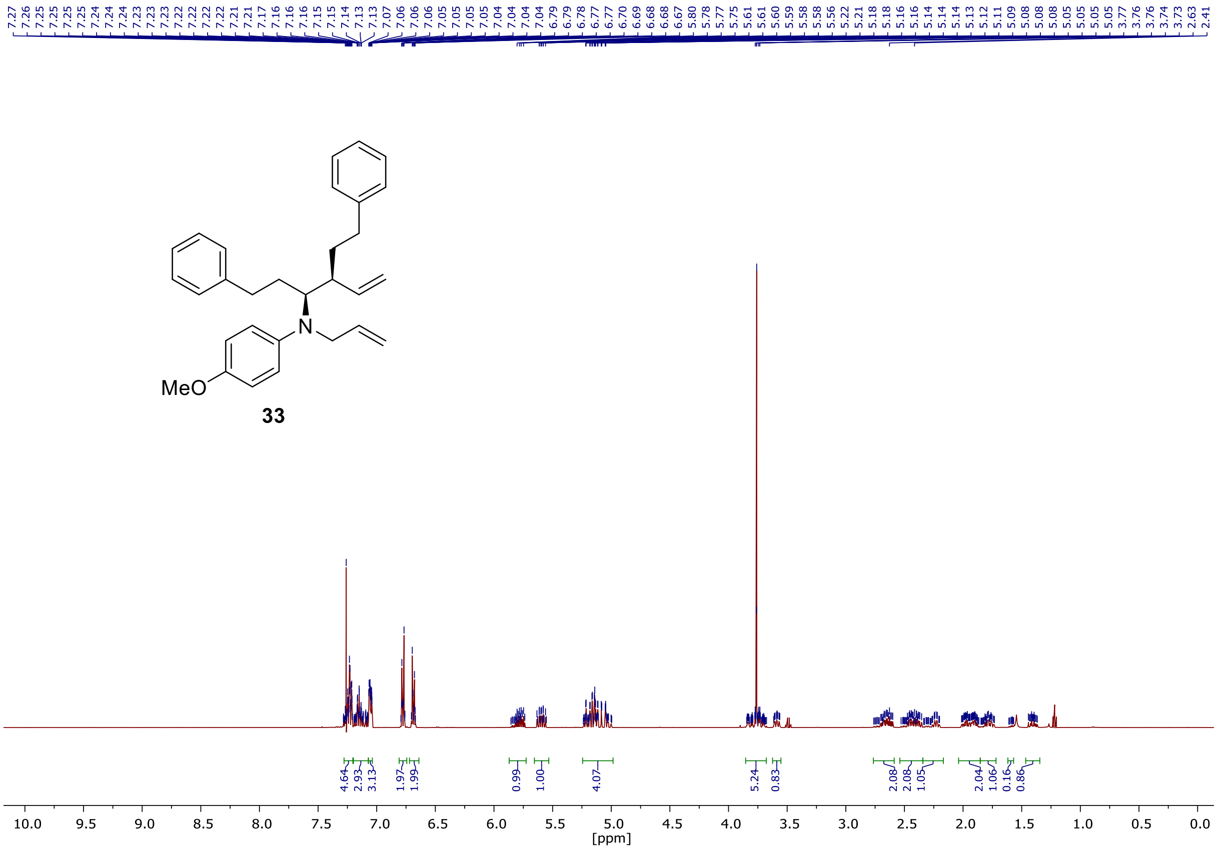
7.27 7.26 7.25 7.25 7.25 7.24 7.24 7.24 7.23 7.23 7.22 7.22 7.22 7.22 7.21 7.17 7.16 7.16 7.16 7.15 7.15 7.14 7.13 7.13 7.07 7.06 7.06 7.05 7.05 7.05 7.05 7.04 7.04 7.04 7.04 6.79 6.79 6.78 6.77 6.77 6.70 6.69 6.68 6.68 6.67 5.80 5.78 5.77 5.75 5.61 5.61 5.60 5.59 5.58 5.58 5.56 5.22 5.21 5.18 5.18 5.16 5.16 5.14 5.14 5.13 5.12 5.11 5.09 5.08 5.08 5.08 5.05 5.05 5.05 5.05 3.77 3.76 3.74 3.73 2.63 2.41
MeO
N
33
4.64 2.93 3.13 1.97 1.99 0.99 1.00 4.07 5.24 0.83 2.08 2.08 1.05 2.04 1.06 0.16 0.86
10.0 9.5 9.0 8.5 8.0 7.5 7.0 6.5 6.0 5.5 5.0 4.5 4.0 3.5 3.0 2.5 2.0 1.5 1.0 0.5 0.0
[ppm]

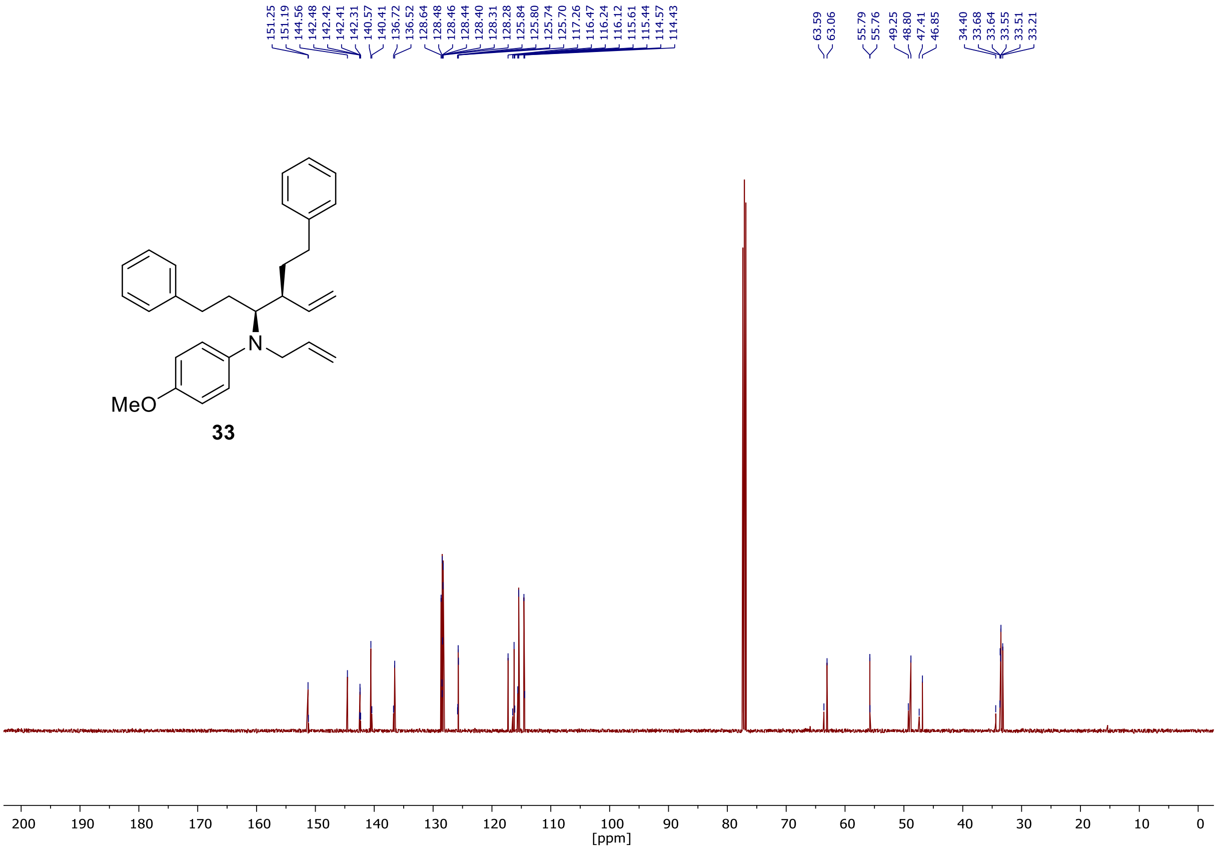
151.25 151.19 144.56 142.48 142.42 142.41 142.31 140.57 140.41 136.72 136.52 128.64 128.48 128.46 128.44 128.40 128.31 128.28 125.84 125.80 125.74 125.70 117.26 116.47 116.24 116.12 115.61 115.44 114.57 114.43
63.59 63.06 55.79 55.76 49.25 48.80 47.41 46.85 34.40 33.68 33.64 33.55 33.51 33.21
MeO
N
33
200 190 180 170 160 150 140 130 120 110 100 90 80 70 60 50 40 30 20 10 0
[ppm]

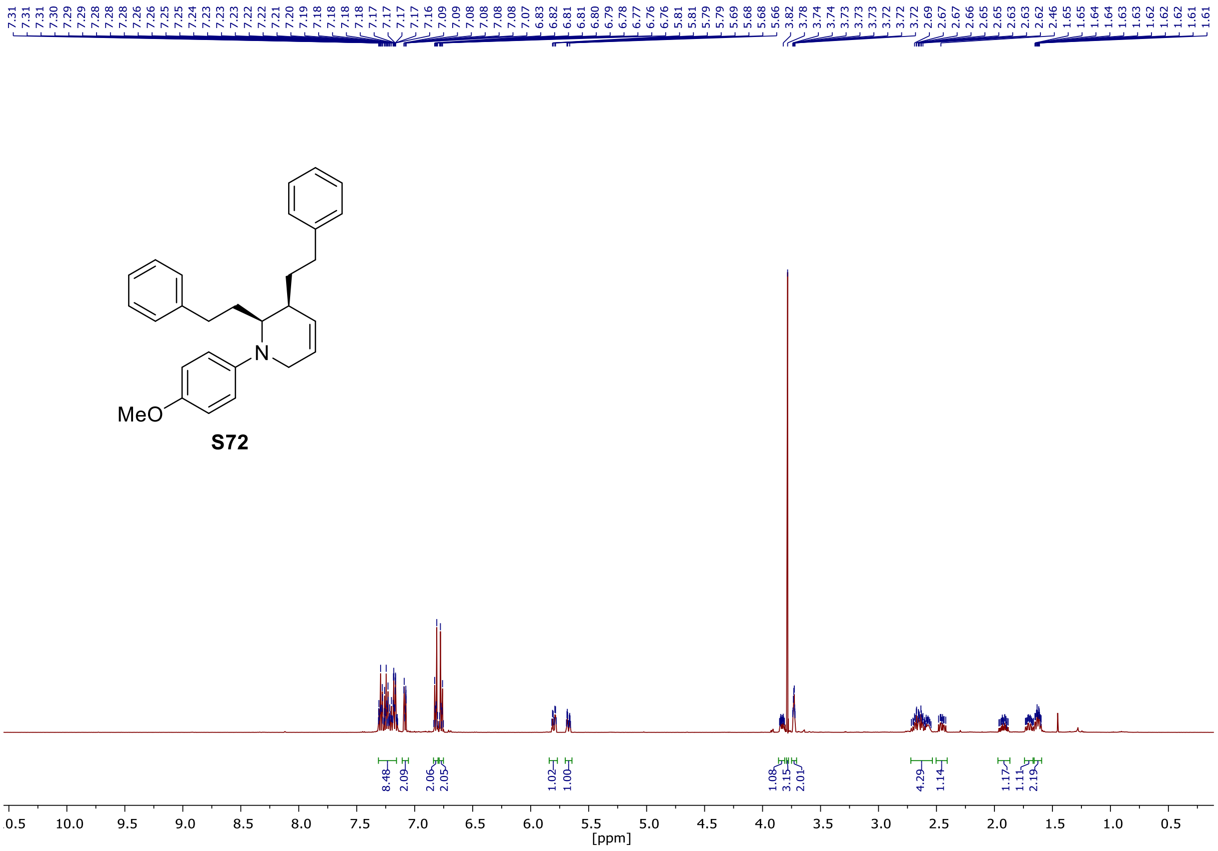
7.31 7.31 7.30 7.29 7.29 7.28 7.28 7.28 7.26 7.25 7.25 7.24 7.23 7.23 7.23 7.22 7.22 7.21 7.20 7.19 7.18 7.18 7.18 7.18 7.17 7.17 7.17 7.16 7.09 7.09 7.08 7.08 7.08 7.07 6.83 6.82 6.81 6.81 6.80 6.79 6.78 6.77 6.76 6.76 5.81 5.81 5.79 5.69 5.68 5.68 5.66 3.82 3.78 3.74 3.74 3.73 3.73 3.73 3.72 3.72 3.72 2.69 2.67 2.66 2.65 2.65 2.63 2.63 2.62 2.46 1.65 1.65 1.64 1.64 1.63 1.63 1.62 1.62 1.61 1.61
MeO
N
S72
8.48 2.09 2.06 2.05 1.02 1.00 1.08 3.15 2.01 4.29 1.14 1.17 1.11 2.19
[ppm]

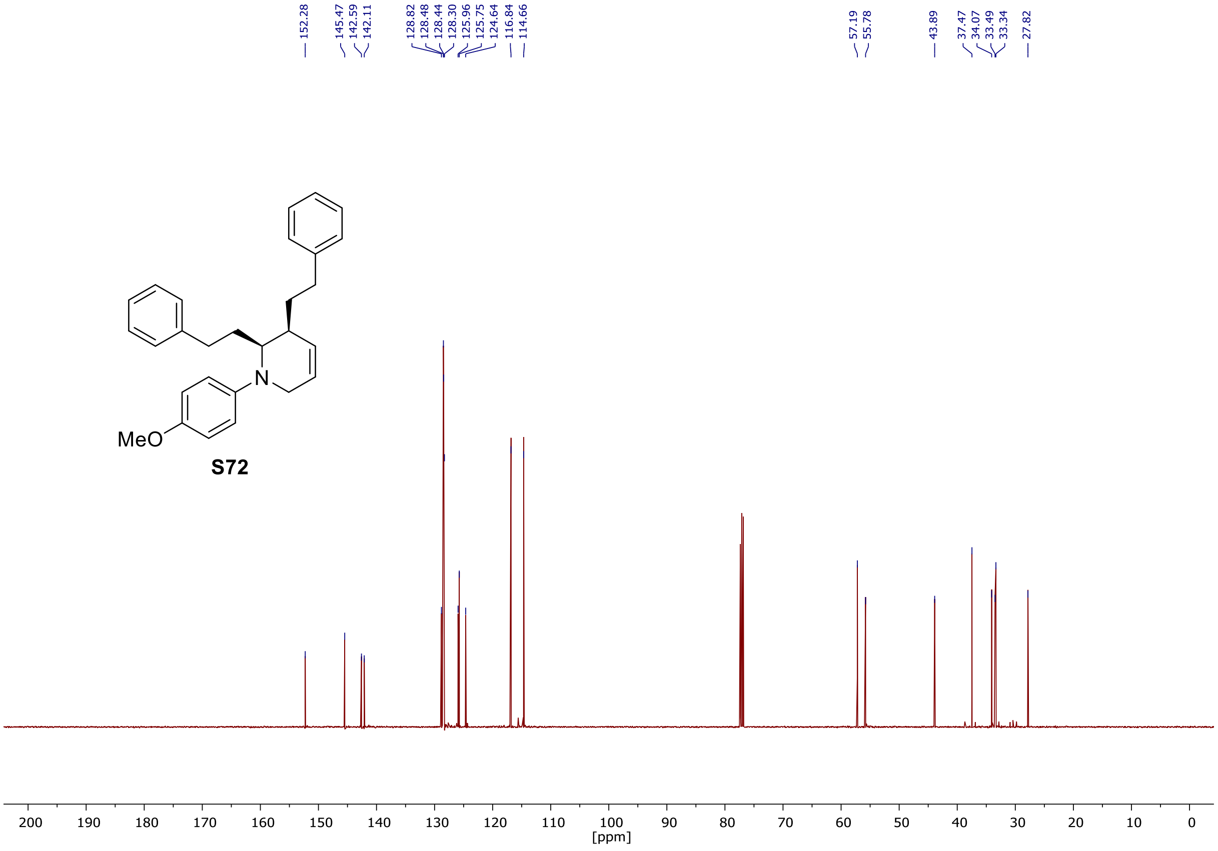
152.28 145.47 142.59 142.11 128.82 128.48 128.44 128.30 125.96 125.75 124.64 116.84 114.66 57.19 55.78 43.89 37.47 34.07 33.49 33.34 27.82
MeO
N
S72
[ppm]

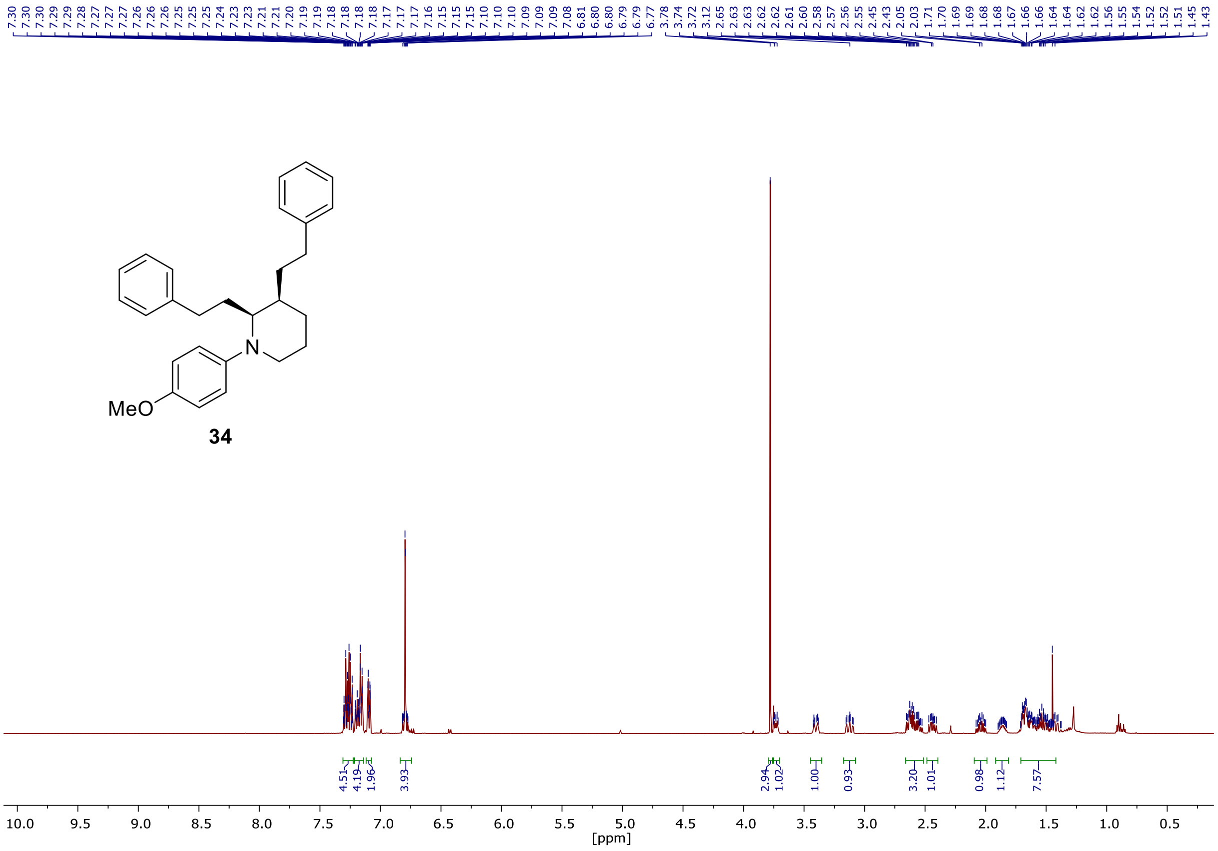
7.30 7.30 7.30 7.29 7.29 7.28 7.27 7.27 7.27 7.26 7.26 7.25 7.25 7.25 7.24 7.23 7.23 7.21 7.21 7.20 7.19 7.19 7.18 7.18 7.18 7.18 7.17 7.17 7.16 7.15 7.15 7.15 7.10 7.10 7.10 7.09 7.09 7.09 7.08 6.81 6.80 6.80 6.79 6.79 6.77 3.78 3.74 3.72 3.12 2.65 2.63 2.63 2.62 2.62 2.61 2.60 2.58 2.57 2.56 2.55 2.45 2.43 2.05 2.03 1.71 1.70 1.69 1.69 1.68 1.68 1.67 1.66 1.66 1.64 1.64 1.62 1.62 1.56 1.55 1.54 1.52 1.52 1.51 1.45 1.43
N
MeO
34
4.51 4.19 1.96 3.93 2.94 1.02 1.00 0.93 3.20 1.01 0.98 1.12 7.57
10.0 9.5 9.0 8.5 8.0 7.5 7.0 6.5 6.0 5.5 5.0 4.5 4.0 3.5 3.0 2.5 2.0 1.5 1.0 0.5
[ppm]

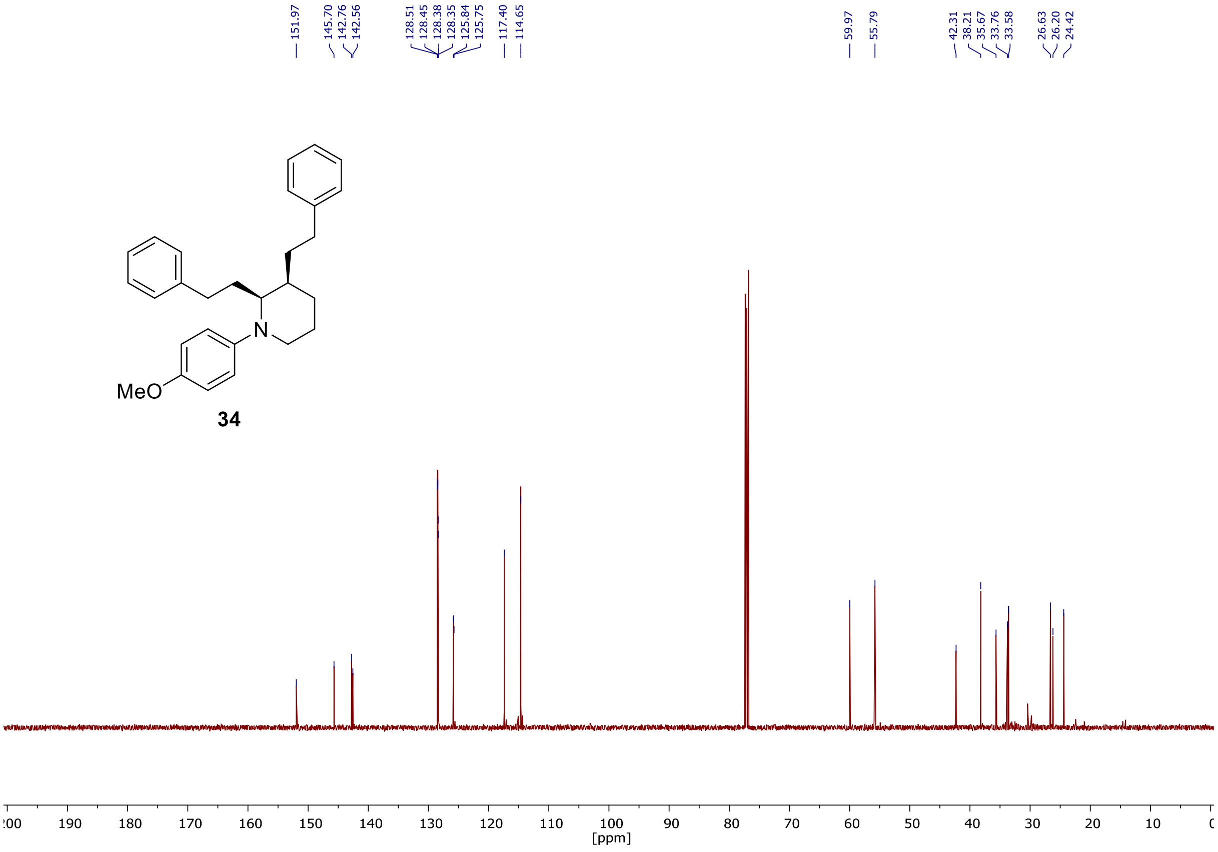
151.97 145.70 142.76 142.56 128.51 128.45 128.38 128.35 125.84 125.75 117.40 114.65 59.97 55.79 42.31 38.21 35.67 33.76 33.58 26.63 26.20 24.42
N
MeO
34
190 180 170 160 150 140 130 120 110 100 90 80 70 60 50 40 30 20 10
[ppm]

DFT Calculations

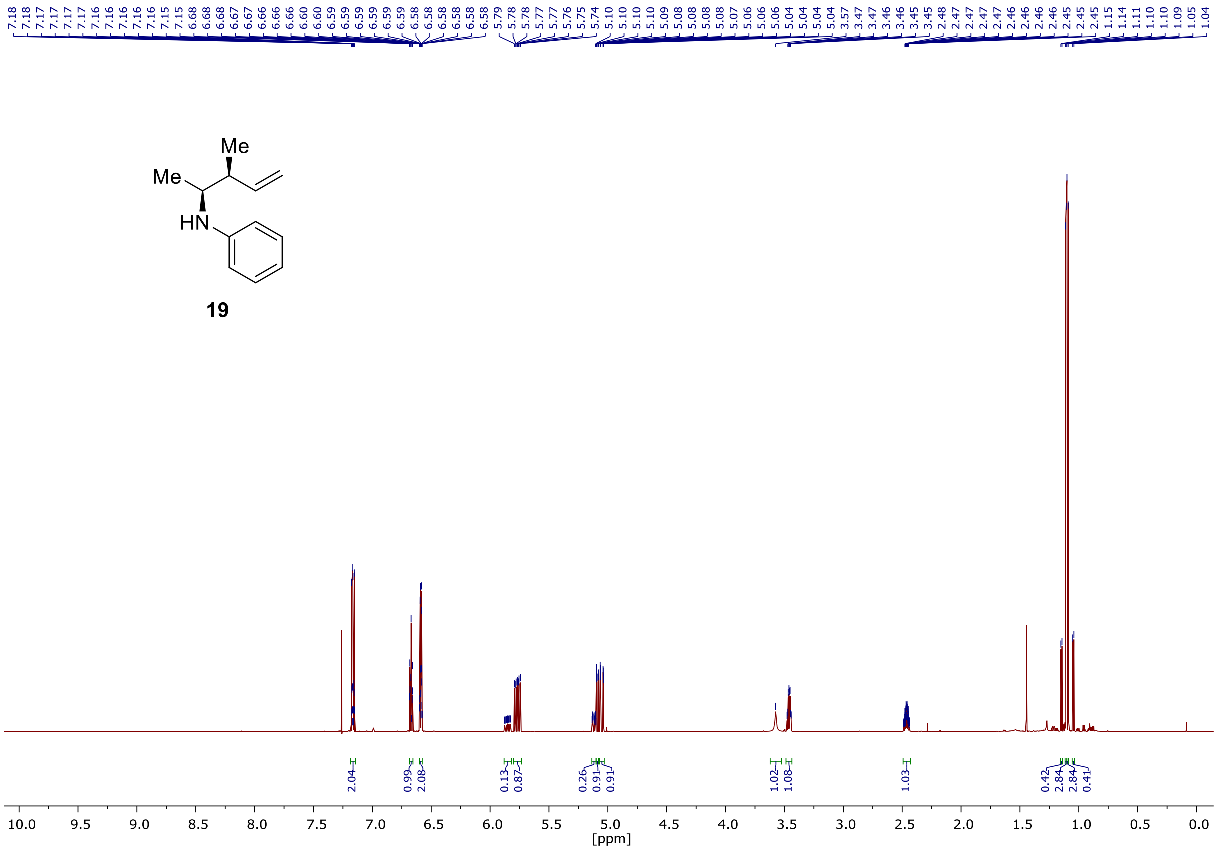

19

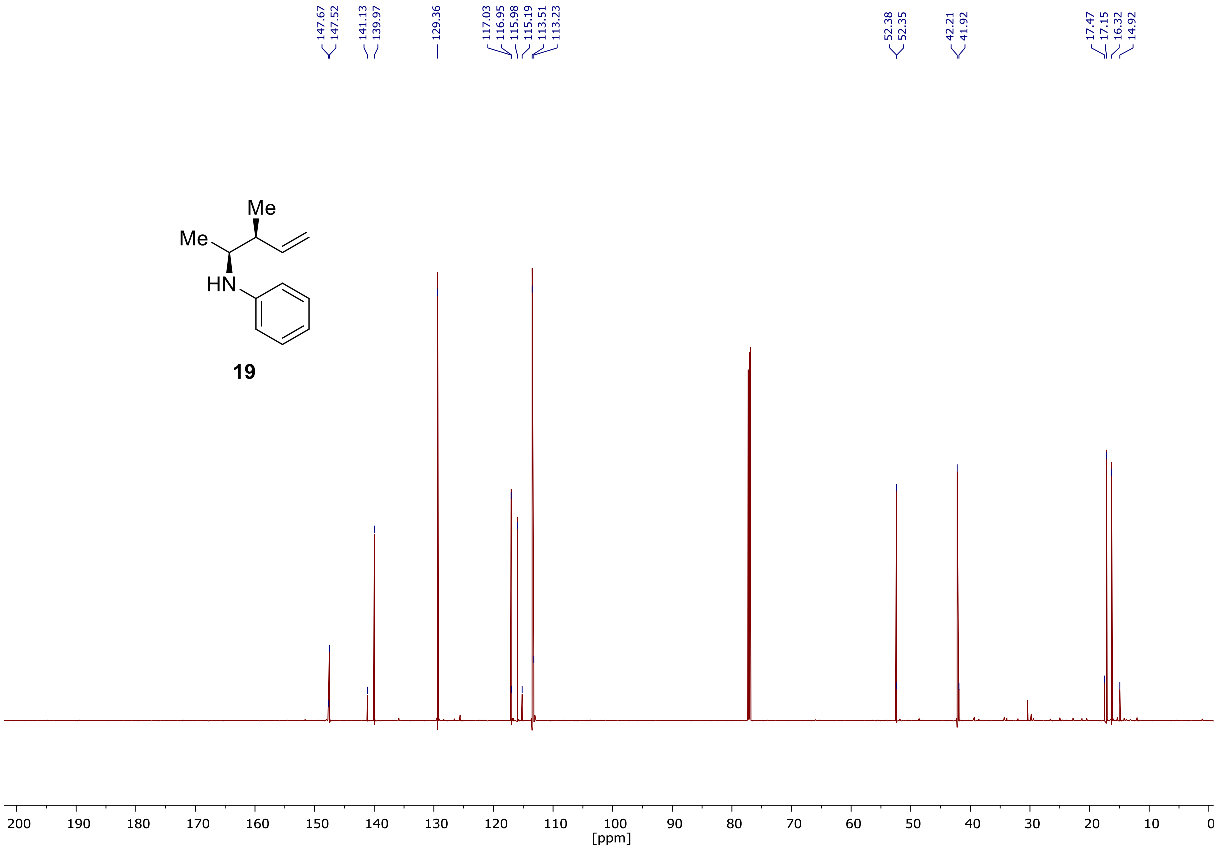

19
